# Comprehensive characterization of flavonoid derivatives in young leaves of core-collected soybean (*Glycine max* L.) cultivars based on high-resolution mass spectrometry

**DOI:** 10.1038/s41598-022-18226-4

**Published:** 2022-08-29

**Authors:** Suji Lee, Heon-Woong Kim, So-Jeong Lee, Ryeong Ha Kwon, Hyemin Na, Ju Hyung Kim, Yu-Mi Choi, Hyemyeong Yoon, Yong-Suk Kim, Chi-Do Wee, Seon Mi Yoo, Sang Hoon Lee

**Affiliations:** 1 Department of Agro-Food Resources, National Institute of Agricultural Sciences, Rural Development Administration, Wanju, 55365 Republic of Korea; 2 National Agrobiodiversity Center, National Institute of Agricultural Sciences, Rural Development Administration, Jeonju, 54874 Republic of Korea; 3 Department of Food Science and Technology, Jeonbuk National University, Jeonju, 54896 Republic of Korea

**Keywords:** Biochemistry, Plant sciences

## Abstract

Most previous studies have been focused on isoflavone profile with biological activities from soybean seed and its related products. However, in the present study, eighty-three flavonoid derivatives (55 flavonols, 9 flavones and 19 isoflavones) were comprehensively identified and quantified from young leaves of 21 core-collected soybean cultivars based on ultra-performance liquid chromatography-diode array detector with quadrupole time of flight/mass spectrometry (UPLC-DAD-QToF/MS). Among total flavonoids from soybean leaves (SLs), the abundant flavonols (83.6%) were primarily composed of di- and tri- glycosides combined to the aglycones (K, kaempferol; Q, quercetin; I, isorhamnetin). Particularly, K-rich SLs (yellow coated seed), Nongrim 51 (breeding line) and YJ208-1 (landrace) contained mainly kaempferol 3-*O*-(2″-*O*-glucosyl-6″-*O*-rhamnosyl)galactoside and 3-*O*-(2″,6″-di-*O*-rhamnosyl)galactoside, and were expected to be superior cultivars by their higher flavonoids. Besides, the new tri-I-glycosides (soyanins I–V) were presented as predominant components in Junyeorikong (landrace, black). Thus, this study suggest that the SLs can be considered as valuable edible resources due to their rich flavonoids. Also, these detailed profiles will support breeding of superior varieties with excellent biological activities as well as relationship with seed anthocyanins production, and contribute to perform metabolomics approach to investigate the changes of SLs flavonols during the leaf growth and fermentation in further research.

## Introduction

Flavonoids are widely distributed as glycosidic form by their group (isoflavone, flavonol, flavone, flavanone, anthocyanin, etc*.*) in most edible plants (vegetables, fruits and seed crops) and have been reported to help in prevention of human diseases such as inflammation, cancer, diabetes, obesity and neurodegeneration^1,2^.

Soybeans (*Glycine max* L.) are isoflavone rich source, and one of the most important crops due to their essential nutrients and biological effects through dietary soy foods (*e.g.* soup, tofu, soy sauce, soymilk)^3,4^. Most previous studies have been focused on the isoflavone profile and its health benefits of soybean seeds^4^. On the other hand, soybean leaves (**SL**s) are considered as potential by-products by their abundant flavonols^5,6^, and consumed as the traditional fermented foods (*Jangajji* and *Kimchi*) using young leaf in Korea^7^.

The **SL**s flavonoid studies have been performed from their extracts based on mass (MS) and nuclear magnetic resonance (NMR) spectroscopies in relation to the potential effect on diabetes^8,9^, lowering cholestetol^10,11^, atherosclerosis^12^ and vascular disease^13^. It was reported that the **SL**s from black and yellow-coated seed collected from Korea were characterized by primarily containing quercetin / isorhamnetin and kaempferol glycosides (**QG**s / **IG**s and **KG**s) depending on their seed coat color, respectively, and both extracts indicated the excellent effects on suppression of hepatic steatosis and promotion of insulin secretion, which are associated with high-fat-diet (HFD) induced obesity and diabetes^14–16^. Besides, from a **KG**s-rich fraction of Japanese unripe cultivar, ‘Jindai’ (**SL**s), kaempferol 3-*O*-(2″-*O*-glucosyl-6″-*O*-rhamnosyl)galactoside and kaempferol 3-*O*-(2″-*O*-glucosyl-6″-*O*-rhamnosyl)glucoside were found to be the predominant **tri**-glycosides to play an important role in reducing blood glucose of diabetic mice^9,17^. Another kaempferol 3-*O*-(2,6-di-*O*-rhamnosyl)galactoside purified from the Jindai cultivar was also determined to have potent antioxidant and hepatoprotective activities^18^.

A total of thirteen flavonol glycosides composed of **QG**s (3 **tri**- and 2 **di**-), **KG**s (4 **tri**- and 2 **di**-), and **IG**s (2 **di**-) were confirmed from the **SL**s of eight Japanese cultivars by MS and NMR elucidation, and among them, quercetin 3-*O*-(4″,6″-di-*O*-rhamnosyl)galactoside ranked the highest proportion as a new **tri**-glycoside in cultivar, ‘Clark’^19,20^. The flavonoid derivatives (12 **QG**s, 7 **KG**s and 5 isoflavones) whose glycosylated type and position are still unclear, were distributed differently in their contents (mg/100 g, dry weight) by Italian cultivars (Emiliana, Kure and Elvir) and plant parts (seeds, leaves, stems, pods and roots), particularly, the flavonols (487.3–1586.8) were much higher than isoflavones (91.3–124.3) in young leaves^6^. Moreover, from Chinese cultivar, its flavonols (**KG**s) were present only in the **SL**s, and contained about six times higher than seed isoflavones^5^.

Although twenty-eight flavonols (14 **KG**s, 10 **QG**s and 4 **IG**s), nine flavones and sixteen isoflavones were identified through previous **SL**s studies, in the **IG**s group^14,16,19^, only four **di**-glycosides (3-*O*-rutinoside, 3-*O*-robinobioside, 3-*O*-(2″-*O*-glucosyl)galactoside and 3-*O*-(2″-*O*-rhamnosyl)galactoside, based on isorhamnetin) were detected at low level from the **SL**s of black coated cultivars. Recently, two **tri**-**IG**s were characterized as isorhamnetin 3-*O*-rhamnosylrhamnosylglucoside and 3-*O*-rhamnosylrhamnosylgalactoside from the leaves of wild Taiwanese *G. max* subsp. *formosana*, but their glycosylated positions have not been determined^21^. Until now, in only few cultivars, most **SL**s flavonoids have been identified mainly using NMR-based techniques, and their detailed quantifications are also limited. Therefore, after the selection of representative soybean cultivars in which the genetic diversity is sufficiently considered, it is required to perform comprehensive structural interpretation based on MS fragmentations of **SL**s flavonoids from these samples.

In this study, based on MS and NMR analytical data reported, a LC–MS library was precisely constructed to carry out comprehensive flavonoids profiling from young leaves of 21 core-collected soybean cultivars. Through the integrated application of LC–MS library and UPLC-DAD-QToF/MS analysis, it was purposed to rapidly identify and quantify numerous flavonoid derivatives including novel **tri**-**IG**s found from the **SL**s. Ultimately, these detailed profiles will support breeding superior varieties which is expected to have excellent biological activities, and this study suggest that the **SL**s can be considered as a valuable edible resource due to their abundant flavonoids.

## Results and discussion

### Identification of 83 flavonoid derivatives in soybean leaves

**Figure 1:**
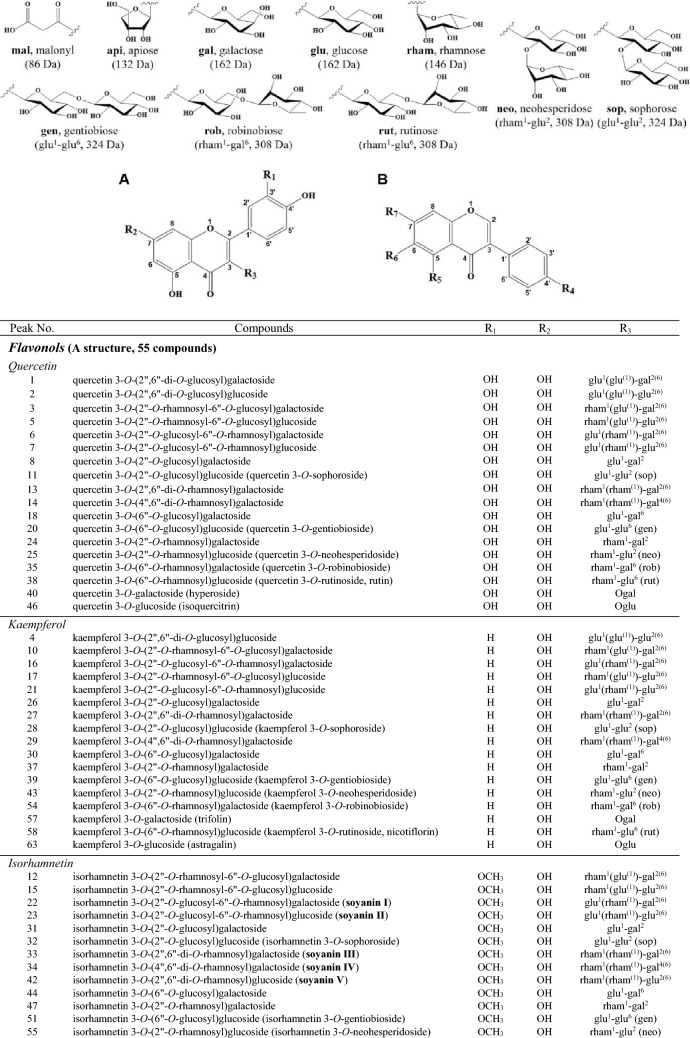

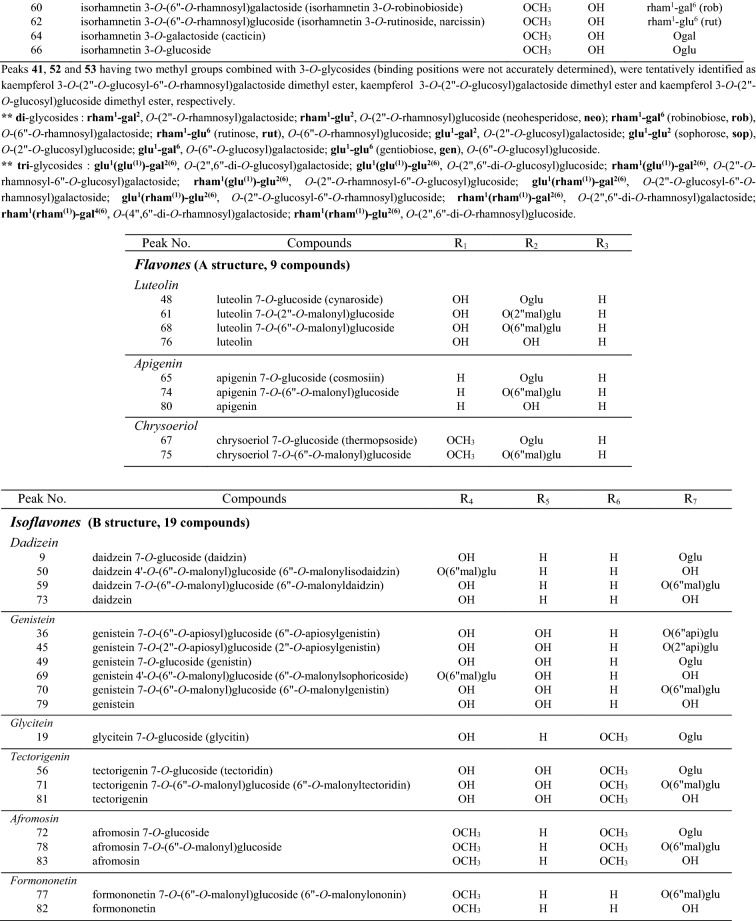
Chemical structures of eighty-three flavonoid derivatives (**A**, 55 flavonols and 9 flavones; **B**, 19 isoflavones) presented from young leaves of 21 soybean cultivars. mal, malonyl; api, apiose; gal, galactose; glu, glucose; rham, rhamnose; gen, gentiobiose; rob, robinobiose; rut, rutinose; neo, neohesperidose; sop, sophorose.

A total of eighty-three flavonoid derivatives consisting of **flavonol (55)**, **flavone (9)** and **isoflavone (19)** derivatives according to basic structures presented in Fig. 1A,B were tentatively identified from young leaves of soybean cultivars by comparing retention time, UV spectra, MS fragmentation using previously constructed NMR and LC–MS library (Table S1) and UPLC-DAD-QToF/MS analysis. These numerous flavonoid derivatives (flavonol-flavone and isoflavone, wavelengths at 350 and 254 nm, respectively) are presented with excellent separation in UPLC-DAD chromatograms of Fig. S1, and detailed with their compound name and MS characteristics by corresponding peak number in Table 1. The positive ionized fragmentation used in this study makes it easy to check the parent ion through adductive sodium (Na^+^, 23 Da), potassium (K^+^, 39 Da) and hydrogen (H^+^, 1 Da) ions as well as the specified glycosidic (*e.g.* glucosyl, glucose—H_2_O) loss from flavonoid structure, when compared with previous negative ionized studies^22,23^.

**Table 1:** Characterization of eighty-three flavonoid derivatives from young leaves of 21 soybean cultivars using UPLC-DAD-QToF/MS.

Peak No	Individual flavonoids	Abbreviation	RT (min)	Molecular Formula	ESI(+)-QToF/MS (experimental ions, *m/z*)		
					[M + H]^+^	error (ppm)	Fragmentation
1^a^	quercetin 3-*O*-(2″,6″-di-*O*-glucosyl)galactoside	Q 3-*O*-(2″,6″-di-*O*-glu)gal	9.03	C_33_H_40_O_22_	789.2092	1.0	827[M + K]^+^, 811[M + Na]^+^, 789[M + H]^+^, 627[M + H-glu]^+^, 465[M + H-2glu]^+^,303[M + H-2glu-gal]^+^
2^a^	quercetin 3-*O*-(2″,6″-di-*O*-glucosyl)glucoside	Q 3-*O*-(2″,6″-di-*O*-glu)glu	9.06	C_33_H_40_O_22_	789.2099	1.9	827[M + K]^+^, 811[M + Na]^+^, 789[M + H]^+^, 627[M + H-glu]^+^, 465[M + H-2glu]^+^,303[M + H-3glu]^+^
3^a^	quercetin 3-*O*-(2″-*O*-rhamnosyl-6″-*O*-glucosyl)galactoside	Q 3-(2″-*O*-rham-6″-*O*-glu)gal	10.02	C_33_H_40_O_21_	773.2138	0.4	811[M + K]^+^, 795[M + Na]^+^, 773[M + H]^+^, 627[M + H-rham]^+^, 611[M + H-glu]^+^, 465[M + H-rham-glu]^+^, 303[M + H-rham-glu-gal]^+^
4^a^	kaempferol 3-*O*-(2″,6″-di-*O*-glucosyl)glucoside	K 3-*O*-(2″,6″-di-*O*-glu)glu	10.38	C_33_H_40_O_21_	773.2139	0.5	811[M + K]^+^, 795[M + Na]^+^, 773[M + H]^+^, 611[M + H-glu]^+^, 449[M + H-2glu]^+^,287[M + H-3glu]^+^
5^a^	quercetin 3-*O*-(2″-*O*-rhamnosyl-6″-*O*-glucosyl)glucoside	Q 3-*O*-(2″-*O*-rham-6″-*O*-glu)glu	10.41	C_33_H_40_O_21_	773.2139	0.5	811[M + K]^+^, 795[M + Na]^+^, 773[M + H]^+^, 627[M + H-rham]^+^, 611[M + H-glu]^+^, 465[M + H-rham-glu]^+^, 303[M + H-rham-2glu]^+^
6	quercetin 3-*O*-(2″-*O*-glucosyl-6″-*O*-rhamnosyl)galactoside	Q 3-*O*-(2″-*O*-glu-6″-*O*-rham)gal	10.43	C_33_H_40_O_21_	773.2138	0.4	811[M + K]^+^, 795[M + Na]^+^, 773[M + H]^+^, 627[M + H-rham]^+^, 611[M + H-glu]^+^, 465[M + H-rham-glu]^+^, 303[M + H-rham-glu-gal]^+^
7	quercetin 3-*O*-(2″-*O*-glucosyl-6″-*O*-rhamnosyl)glucoside	Q 3-*O*-(2″-*O*-glu-6″-*O*-rham)glu	10.65	C_33_H_40_O_21_	773.2136	0.2	811[M + K]^+^, 795[M + Na]^+^, 773[M + H]^+^, 627[M + H-rham]^+^, 611[M + H-glu]^+^, 465[M + H-rham-glu]^+^, 303[M + H-rham-2glu]^+^
8	quercetin 3-*O*-(2″-*O*-glucosyl)galactoside	Q 3-*O*-(2″-*O*-glu)gal	11.16	C_27_H_30_O_17_	627.1548	−1.2	665[M + K]^+^, 649[M + Na]^+^, 627[M + H]^+^, 465[M + H-glu]^+^, 303[M + H-glu-gal]^+^
9^c^	daidzein 7-*O*-glucoside (daidzin)	D 7-*O*-glu	11.25	C_21_H_20_O_9_	417.1181	0.2	455[M + K]^+^, 439[M + Na]^+^,417[M + H]^+^, 255[M + H-glu]^+^
10	kaempferol 3-*O*-(2″-*O*-rhamnosyl-6″-*O*-glucosyl)galactoside	K 3-*O*-(2″-*O*-rham-6″-*O*-glu)gal	11.35	C_33_H_40_O_20_	757.2194	0.7	795[M + K]^+^, 779[M + Na]^+^, 757[M + H]^+^, 611[M + H-rham]^+^, 595[M + H-glu]^+^, 449[M + H-rham-glu]^+^, 287[M + H-rham-glu-gal]^+^
11^c^	quercetin 3-*O*-(2″-*O*-glucosyl)glucoside (quercetin 3-*O*-sophoroside)	Q 3-*O*-(2″-*O*-glu)glu	11.39	C_27_H_30_O_17_	627.1545	−1.7	665[M + K]^+^, 649[M + Na]^+^, 627[M + H]^+^, 465[M + H-glu]^+^, 303[M + H-2glu]^+^
12^a^	isorhamnetin 3-*O*-(2″-*O*-rhamnosyl-6″-*O*-glucosyl)galactoside	I 3-*O*-(2″-*O*-rham-6″-*O*-glu)gal	11.54	C_34_H_42_O_21_	787.2293	0.2	825[M + K]^+^, 809[M + Na]^+^, 787[M + H]^+^, 641[M + H-rham]^+^, 625[M + H-glu]^+^, 479[M + H-rham-glu]^+^, 317[M + H-rham-glu-gal]^+^, 302[M + H-rham-glu-gal-CH_3_]^+^
13^a^	quercetin 3-*O*-(2″,6″-di-*O*-rhamnosyl)galactoside	Q 3-*O*-(2″,6″-di-*O*-rham)gal	11.55	C_33_H_40_O_20_	757.2190	0.6	795[M + K]^+^, 779[M + Na]^+^, 757[M + H]^+^, 611[M + H-rham]^+^, 465[M + H-2rham]^+^, 303[M + H-2rham-gal]^+^
14	quercetin 3-*O*-(4″,6″-di-*O*-rhamnosyl)galactoside	Q 3-*O*-(4″,6″-di-*O*-rham)gal	11.71	C_33_H_40_O_20_	757.2189	0.4	795[M + K]^+^, 779[M + Na]^+^, 757[M + H]^+^, 611[M + H-rham]^+^, 465[M + H-2rham]^+^, 303[M + H-2rham-gal]^+^
15^a^	isorhamnetin 3-*O*-(2″-*O*-rhamnosyl-6″-*O*-glucosyl)glucoside	I 3-*O*-(2″-*O*-rham-6″-*O*-glu)glu	11.79	C_34_H_42_O_21_	787.2289	−0.3	825[M + K]^+^, 809[M + Na]^+^, 787[M + H]^+^, 641[M + H-rham]^+^, 625[M + H-glu]^+^, 479[M + H-rham-glu]^+^, 317[M + H-rham-2glu]^+^, 302[M + H-rham-2glu-CH_3_]^+^
16	kaempferol 3-*O*-(2″-*O*-glucosyl-6″-*O*-rhamnosyl)galactoside	K 3-*O*-(2″-*O*-glu-6″-*O*-rham)gal	11.83	C_33_H_40_O_20_	757.2159	−3.5	795[M + K]^+^, 779[M + Na]^+^, 757[M + H]^+^, 611[M + H-rham]^+^, 595[M + H-glu]^+^, 449[M + H-rham-glu]^+^, 287[M + H-rham-glu-gal]^+^
17^a^	kaempferol 3-*O*-(2″-*O*-rhamnosyl-6″-*O*-glucosyl)glucoside	K 3-*O*-(2″-*O*-rham-6″-*O*-glu)glu	11.84	C_33_H_40_O_20_	757.2189	0.4	795[M + K]^+^, 779[M + Na]^+^, 757[M + H]^+^, 611[M + H-rham]^+^, 595[M + H-glu]^+^, 449[M + H-rham-glu]^+^, 287[M + H-rham-2glu]^+^
18^a^	quercetin 3-*O*-(6″-*O*-glucosyl)galactoside	Q 3-*O*-(6″-*O*-glu)gal	11.99	C_27_H_30_O_17_	627.1559	0.5	665[M + K]^+^, 649[M + Na]^+^, 627[M + H]^+^, 465[M + H-glu]^+^, 303[M + H-glu-gal]^+^
19^c^	glycitein 7-*O*-glucoside (glycitin)	G_y_ 7-*O*-glu	12.07	C_22_H_22_O_10_	447.1286	0.1	485[M + K]^+^, 469[M + Na]^+^,447[M + H]^+^, 285[M + H-glu]^+^_,_ 270[M + H-glu-CH_3_]^+^
20^a,c^	quercetin 3-*O*-(6″-*O*-glucosyl)glucoside (quercetin 3-*O*-gentiobioside)	Q 3-*O*-(6″-*O*-glu)glu	12.15	C_27_H_30_O_17_	627.1550	−0.9	665[M + K]^+^, 649[M + Na]^+^, 627[M + H]^+^, 465[M + H-glu]^+^, 303[M + H-2glu]^+^
21	kaempferol 3-*O*-(2″-*O*-glucosyl-6″-*O*-rhamnosyl)glucoside	K 3-*O*-(2″-*O*-glu-6″-*O*-rham)glu	12.16	C_33_H_40_O_20_	757.2160	−3.4	795[M + K]^+^, 779[M + Na]^+^, 757[M + H]^+^, 611[M + H-rham]^+^, 595[M + H-glu]^+^, 449[M + H-rham-glu]^+^, 287[M + H-rham-2glu]^+^
22^a,b^	isorhamnetin 3-*O*-(2″-*O*-glucosyl-6″-*O*-rhamnosyl)galactoside (**soyanin I**)	I 3-*O*-(2″-*O*-glu-6″-rham)gal	12.36	C_34_H_42_O_21_	787.2283	−1.1	825[M + K]^+^, 809[M + Na]^+^, 787[M + H]^+^, 641[M + H-rham]^+^, 625[M + H-glu]^+^, 479[M + H-rham-glu]^+^, 317[M + H-rham-glu-gal]^+^
23^a,b^	isorhamnetin 3-*O*-(2″-*O*-glucosyl-6″-*O*-rhamnosyl)glucoside (**soyanin II**)	I 3-*O*-(2″-*O*-glu-6″-*O*-rham)glu	12.36	C_34_H_42_O_21_	787.2281	−1.3	825[M + K]^+^, 809[M + Na]^+^, 787[M + H]^+^, 641[M + H-rham]^+^, 625[M + H-glu]^+^, 479[M + H-rham-glu]^+^, 317[M + H-rham-2glu]^+^
24	quercetin 3-*O*-(2″-*O*-rhamnosyl)galactoside	Q 3-*O*-(2″-*O*-rham)gal	12.40	C_27_H_30_O_16_	611.1613	1.0	649[M + K]^+^, 633[M + Na]^+^, 611[M + H]^+^, 465[M + H-rham]^+^, 303[M + H-rham-gal]^+^
25^a^	quercetin 3-*O*-(2″-*O*-rhamnosyl)glucoside (quercetin 3-*O*-neohesperidoside)	Q 3-*O*-(2″-*O*-rham)glu	12.68	C_27_H_30_O_16_	611.1612	0.9	649[M + K]^+^, 633[M + Na]^+^, 611[M + H]^+^, 465[M + H-rham]^+^, 303[M + H-rham-glu]^+^
26	kaempferol 3-*O*-(2″-*O*-glucosyl)galactoside	K 3-*O*-(2″-*O*-glu)gal	12.75	C_27_H_30_O_16_	611.1602	−0.8	649[M + K]^+^, 633[M + Na]^+^, 611[M + H]^+^, 449[M + H-glu]^+^, 287[M + H-glu-gal]^+^
27	kaempferol 3-*O*-(2″,6″-di-*O*-rhamnosyl)galactoside	K 3-*O*-(2″,6″-di-*O*-rham)gal	12.80	C_33_H_40_O_19_	741.2240	0.5	779[M + K]^+^, 763[M + Na]^+^, 741[M + H]^+^, 595[M + H-rham]^+^, 449[M + H-2rham]^+^, 287[M + H-2rham-gal]^+^
28	kaempferol 3-*O*-(2″-*O*-glucosyl)glucoside (kaempferol 3-*O*-sophoroside)	K 3-*O*-(2″-*O*-glu)glu	12.99	C_27_H_30_O_16_	611.1602	−0.8	649[M + K]^+^, 633[M + Na]^+^, 611[M + H]^+^, 449[M + H-glu]^+^, 287[M + H-2glu]^+^
29	kaempferol 3-*O*-(4″,6″-di-*O*-rhamnosyl)galactoside	K 3-*O*-(4″,6″-di-*O*-rham)gal	13.11	C_33_H_40_O_19_	741.2259	0.3	779[M + K]^+^, 763[M + Na]^+^, 741[M + H]^+^, 595[M + H-rham]^+^, 449[M + H-2rham]^+^, 287[M + H-2rham-gal]^+^
30^a^	kaempferol 3-*O*-(6″-*O*-glucosyl)galactoside	K 3-*O*-(6″-*O*-glu)gal	13.27	C_27_H_30_O_16_	611.1610	0.6	649[M + K]^+^, 633[M + Na]^+^, 611[M + H]^+^, 449[M + H-glu]^+^, 325[glu + gal + H]^+^, 287[M + H-glu-gal]^+^
31	isorhamnetin 3-*O*-(2″-*O*-glucosyl)galactoside	I 3-*O*-(2″-*O*-glu)gal	13.30	C_28_H_32_O_17_	641.1705	−1.1	679[M + K]^+^, 663[M + Na]^+^, 641[M + H]^+^, 479[M + H-glu]^+^, 317[M + H-glu-gal]^+^
32^a^	isorhamnetin 3-*O*-(2″-*O*-glucosyl)glucoside (isorhamnetin 3-*O*-sophoroside)	I 3-*O*-(2″-*O*-glu)glu	13.30	C_28_H_32_O_17_	641.1705	−0.7	679[M + K]^+^, 663[M + Na]^+^, 641[M + H]^+^, 479[M + H-glu]^+^, 317[M + H-2glu]^+^
33^a,b^	isorhamnetin 3-*O*-(2″,6″-di-*O*-rhamnosyl)galactoside (**soyanin III**)	I 3-*O*-(2″,6″-di-*O*-rham)gal	13.37	C_34_H_42_O_20_	771.2345	0.4	809[M + K]^+^, 793[M + Na]^+^, 771[M + H]^+^, 625[M + H-rham]^+^, 479[M + H-2rham]^+^, 317[M + H-2rham-gal]^+^
34^a,b^	isorhamnetin 3-*O*-(4″,6″-di-*O*-rhamnosyl)galactoside (**soyanin IV**)	I 3-*O*-(4″,6″-di-*O*-rham)gal	13.59	C_34_H_42_O_20_	771.2342	0.0	809[M + K]^+^, 793[M + Na]^+^, 771[M + H]^+^, 625[M + H-rham]^+^, 479[M + H-2rham]^+^, 317[M + H-2rham-gal]^+^
35	quercetin 3-*O*-(6″-*O*-rhamnosyl)galactoside (quercetin 3-*O*-robinobioside)	Q 3-*O*-(6″-*O*-rham)gal	13.59	C_27_H_30_O_16_	611.1611	0.7	649[M + K]^+^, 633[M + Na]^+^, 611[M + H]^+^, 465[M + H-rham]^+^, 303[M + H-rham-gal]^+^
36^a^	genistein 7-*O*-(6″-*O*-apiosyl)glucoside (6″-*O*-apiosylgenistin)	G_n_ 7-*O*-(6″-*O*-api)glu	13.69	C_26_H_28_O_14_	565.1555	0.6	603[M + K]^+^, 587[M + Na]^+^, 565[M + H]^+^, 433[M + H-api]^+^, 271[M + H-api-glu]^+^
37	kaempferol 3-*O*-(2″-*O*-rhamnosyl)galactoside	K 3-*O*-(2″-*O*-rham)gal	13.83	C_27_H_30_O_15_	595.1658	0.1	633[M + K]^+^, 617[M + Na]^+^, 595[M + H]^+^, 449[M + H-rham]^+^, 287[M + H-rham-gal]^+^
38^c^	quercetin 3-*O*-(6″-*O*-rhamnosyl)glucoside (quercetin 3-*O*-rutinoside, rutin)	Q 3-*O*-(6″-*O*-rham)glu	13.95	C_27_H_30_O_16_	611.1603	−0.6	649[M + K]^+^, 633[M + Na]^+^, 611[M + H]^+^, 465[M + H-rham]^+^, 303[M + H-rham-glu]^+^
39^c^	kaempferol 3-*O*-(6″-*O*-glucosyl)glucoside (kaempferol 3-*O*-gentiobioside)	K 3-*O*-(6″-*O*-glu)glu	14.01	C_27_H_30_O_16_	611.1607	0.1	649[M + K]^+^, 633[M + Na]^+^, 611[M + H]^+^, 449[M + H-glu]^+^, 325[2glul + H]^+^, 287[M + H-2glu]^+^
40^c^	quercetin 3-*O*-galactoside (hyperoside)	Q 3-*O*-gal	14.04	C_21_H_20_O_12_	465.1032	1.0	503[M + K]^+^, 487[M + Na]^+^, 465[M + H]^+^, 303[M + H-gal]^+^
41^a^	kaempferol 3-*O*-(2″-*O*-glucosyl-6″-*O*-rhamnosyl)galactoside dimethyl ester	K 3-*O*-(2″-*O*-glu-6″-*O*-rham)gal DME	14.06	C_35_H_44_O_20_	785.2136	−46.2	823[M + K]^+^, 807[M + Na]^+^, 785[M + H]^+^, 639[M + H-rham]^+^, 287[M + H-rham-glu-gal-2CH_3_]^+^
42^a,b^	isorhamnetin 3-*O*-(2″,6″-di-*O*-rhamnosyl)glucoside (**soyanin V**)	I 3-*O*-(2″,6″-di-*O*-rham)glu	14.14	C_34_H_42_O_20_	771.2350	1.0	809[M + K]^+^, 793[M + Na]^+^, 771[M + H]^+^, 625[M + H-rham]^+^, 479[M + H-2rham]^+^, 317[M + H-2rham-glu]^+^
43^a^	kaempferol 3-*O*-(2″-*O*-rhamnosyl)glucoside (kaempferol 3-*O*-neohesperidoside)	K 3-*O*-(2″-*O*-rham)glu	14.22	C_27_H_30_O_15_	595.1657	−0.1	633[M + K]^+^, 617[M + Na]^+^, 595[M + H]^+^, 449[M + H-rham]^+^, 287[M + H-rham-glu]^+^
44^a^	isorhamnetin 3-*O*-(6″-*O*-glucosyl)galactoside	I 3-*O*-(6″-*O*-glu)gal	14.27	C_28_H_32_O_17_	641.1712	0.0	679[M + K]^+^, 663[M + Na]^+^, 641[M + H]^+^, 479[M + H-glu]^+^, 317[M + H-glu-gal]^+^ 302[M + H-glu-gal-CH_3_]^+^
45^a^	genistein 7-*O*-(2″-*O*-apiosyl)glucoside (2″-*O*-apiosylgenistin)	G_n_ 7-*O*-(2″-*O*-api)glu	14.37	C_26_H_28_O_14_	565.1553	0.2	603[M + K]^+^, 587[M + Na]^+^, 565[M + H]^+^, 433[M + H-api]^+^, 271[M + H-api-glu]^+^
46^c^	quercetin 3-*O*-glucoside (isoquercitrin)	Q 3-*O*-glu	14.46	C_21_H_20_O_12_	465.1032	1.0	503[M + K]^+^, 487[M + Na]^+^, 465[M + H]^+^, 303[M + H-glu]^+^
47	isorhamnetin 3-*O*-(2″-*O*-rhamnosyl)galactoside	I 3-*O*-(2″-*O*-rham)gal	14.46	C_28_H_32_O_16_	625.1769	0.9	663[M + K]^+^, 647[M + Na]^+^, 625[M + H]^+^, 479[M + H-rham]^+^, 317[M + H-rham-gal]^+^
48^c^	luteolin 7-*O*-glucoside (cynaroside)	L 7-*O*-glu	14.48	C_21_H_20_O_11_	449.1080	0.4	449[M + H]^+^, 287[M + H-glu]^+^
49^c^	genistein 7-*O*-glucoside (genistin)	G_n_ 7-*O*-glu	14.63	C_21_H_20_O_10_	433.1128	−0.3	471[M + K]^+^, 455[M + Na]^+^, 433[M + H]^+^, 271[M + H-glu]^+^
50^a^	daidzein 4'-*O*-(6″-*O*-malonyl)glucoside	D 4'-*O*-(6″-*O*-mal)glu	14.66	C_24_H_22_O_12_	503.1183	−0.2	541[M + K]^+^, 525[M + Na]^+^, 503[M + H]^+^, 255[M + H-mal-glu]^+^
51^a^	isorhamnetin 3-*O*-(6″-*O*-glucosyl)glucoside (isorhamnetin 3-*O*-gentiobioside)	I 3-*O*-(6″-*O*-glu)glu	14.67	C_28_H_32_O_17_	641.1711	−0.2	679[M + K]^+^, 663[M + Na]^+^, 641[M + H]^+^, 479[M + H-glu]^+^, 317[M + H-2glu]^+^ 302[M + H-2glu-CH_3_]^+^
52^a^	kaempferol 3-*O*-(2″-*O*-glucosyl)galactoside dimethyl ester	K 3-*O*-(2″-*O*-glu)gal DME	14.90	C_29_H_34_O_16_	639.1558	−56.6	677[M + K]^+^, 661[M + Na]^+^, 639[M + H]^+^, 287[M + H-glu-gal-2CH_3_]^+^
53^a^	kaempferol 3-*O*-(2″-*O*-glucosyl)glucoside dimethyl ester	K 3-*O*-(2″-*O*-glu)glu DME	15.03	C_29_H_34_O_16_	639.1559	−56.4	677[M + K]^+^, 661[M + Na]^+^, 639[M + H]^+^, 287[M + H-2glu-2CH_3_]^+^
54	kaempferol 3-*O*-(6″-*O*-rhamnosyl)galactoside (kaempferol 3-*O*-robinobioside, biorobin)	K 3-*O*-(6″-*O*-rham)gal	15.03	C_27_H_30_O_15_	595.1658	0.1	633[M + K]^+^, 617[M + Na]^+^, 595[M + H]^+^, 449[M + H-rham]^+^, 287[M + H-rham-gal]^+^
55^a,c^	isorhamnetin 3-*O*-(2″-*O*-rhamnosyl)glucoside (isorhamnetin 3-*O*-neohesperidoside, calendoflavoside)	I 3-*O*-(2″-*O*-rham)glu	15.13	C_28_H_32_O_16_	625.1767	0.6	663[M + K]^+^, 647[M + Na]^+^, 625[M + H]^+^, 479[M + H-rham]^+^, 317[M + H-rham-glu]^+^
56^a^	tectorigenin 7-*O*-glucoside (tectoridin)	T 7-*O*-glu	15.37	C_22_H_22_O_11_	463.1239	0.9	501[M + K]^+^, 485[M + Na]^+^, 463[M + H]^+^, 301[M + H-glu]^+^, 286[M + H-glu-CH_3_]^+^
57^a,c^	kaempferol 3-*O*-galactoside (trifolin)	K 3-*O*-gal	15.67	C_21_H_20_O_11_	449.1083	1.0	487[M + K]^+^, 471[M + Na]^+^, 449[M + H]^+^, 287[M + H-gal]^+^
58^c^	kaempferol 3-*O*-(6″-*O*-rhamnosyl)glucoside (kaempferol 3-*O*-rutinoside, nicotiflorin)	K 3-*O*-(6″-*O*-rham)glu	15.95	C_27_H_30_O_15_	595.1657	−0.1	633[M + K]^+^, 617[M + Na]^+^, 595[M + H]^+^, 449[M + H-rham]^+^, 287[M + H-rham-glu]^+^
59^c^	daidzein 7-*O*-(6″-*O*-malonyl)glucoside (6″-*O*-malonyldaidzin)	D 7-*O*-(6″-*O*-mal)glu	16.09	C_24_H_22_O_12_	503.1185	0.2	541[M + K]^+^, 525[M + Na]^+^, 503[M + H]^+^, 255[M + H-mal-glu]^+^
60	isorhamnetin 3-*O*-(6″-*O*-rhamnosyl)galactoside (isorhamnetin 3-*O*-robinobioside)	I 3-*O*-(6″-*O*-rham)gal	16.12	C_28_H_32_O_16_	625.1764	0.1	663[M + K]^+^, 647[M + Na]^+^, 625[M + H]^+^, 479[M + H-rham]^+^, 317[M + H-rham-gal]^+^
61	luteolin 7-*O*-(2″-*O*-malonyl)glucoside	L 7-*O*-(2″-*O*-mal)glu	16.24	C_24_H_22_O_14_	535.1089	1.3	573[M + K]^+^, 557[M + Na]^+^, 535[M + H]^+^, 287[M + H-mal-glu]^+^
62^c^	isorhamnetin 3-*O*-(6″-*O*-rhamnosyl)glucoside (isorhamnetin 3-*O*-rutinoside, narcissin)	I 3-*O*-(6″-*O*-rham)glu	16.48	C_28_H_32_O_16_	625.1761	−0.3	663[M + K]^+^, 647[M + Na]^+^, 625[M + H]^+^, 479[M + H-rham]^+^, 317[M + H-rham-glu]^+^
63^c^	kaempferol 3-*O*-glucoside (astragalin)	K 3-*O*-glu	16.49	C_21_H_20_O_11_	449.1084	1.2	487[M + K]^+^, 471[M + Na]^+^, 449[M + H]^+^, 287[M + H-glu]^+^
64^a,c^	isorhamnetin 3-*O*-galactoside (cacticin)	I 3-*O*-gal	16.60	C_22_H_22_O_12_	479.1191	1.5	517[M + K]^+^, 501[M + Na]^+^, 479[M + H]^+^, 317[M + H-gal]^+^
65^c^	apigenin 7-*O*-glucoside (cosmosiin)	A_p_ 7-*O*-glu	16.95	C_21_H_20_O_10_	433.1133	0.9	455[M + Na]^+^, 433[M + H]^+^, 271[M + H-glu]^+^
66^a,c^	isorhamnetin 3-*O*-glucoside	I 3-*O*-glu	17.05	C_22_H_22_O_12_	479.1188	0.8	517[M + K]^+^, 501[M + Na]^+^, 479[M + H]^+^, 317[M + H-glu]^+^
67	chrysoeriol 7-*O*-glucoside (thermopsoside)	C 7-*O*-glu	17.70	C_22_H_22_O_11_	463.1237	0.5	485[M + Na]^+^, 463[M + H]^+^, 301[M + H-glu]^+^, 286[M + H-glu-CH_3_]^+^
68^a^	luteolin 7-*O*-(6″-*O*-malonyl)glucoside	L 7-*O*-(6″-*O*-mal)glu	17.98	C_24_H_22_O_14_	535.1084	0.3	557[M + Na]^+^, 535[M + H]^+^, 287[M + H-mal-glu]^+^
69^a^	genistein 4'-*O*-(6″-*O*-malonyl)glucoside	G_n_ 4'-*O*-(6″-*O*-mal)glu	18.12	C_24_H_22_O_13_	519.1136	0.5	557[M + K]^+^, 541[M + Na]^+^, 519[M + H]^+^, 271[M + H-mal-glu]^+^
70^c^	genistein 7-*O*-(6″-*O*-malonyl)glucoside (6″-*O*-malonylgenistin)	G_n_ -7-*O*-(6″-*O*-mal)glu	19.16	C_24_H_22_O_13_	519.1130	−0.6	557[M + K]^+^, 541[M + Na]^+^, 519[M + H]^+^, 271[M + H-mal-glu]^+^
71^a^	tectorigenin 7-*O*-(6″-*O*-malonyl)glucoside (6″-*O*-malonyltectoridin)	T 7-*O*-(6″-*O*-mal)glu	19.71	C_25_H_24_O_14_	549.1237	−0.3	587[M + K]^+^, 571[M + Na]^+^, 549[M + H]^+^, 301[M + H-mal-glu]^+^_,_ 286[M + H-glu-CH_3_]^+^
72	afromosin 7-*O*-glucoside	A_f_ 7-*O*-glu	19.71	C_23_H_24_O_10_	461.1445	0.6	499[M + K]^+^, 483[M + Na]^+^, 461[M + H]^+^, 299[M + H-glu]^+^, 284[M + H-glu-CH_3_]^+^
73^c^	daidzein	D	20.17	C_15_H_10_O_4_	255.0653	0.4	277[M + Na]^+^, 255[M + H]^+^
74^a^	apigenin 7-*O*-(6″-*O*-malonyl)glucoside	A_p_ 7-*O*-(6″-*O*-mal)glu	20.50	C_24_H_22_O_13_	519.1129	−0.8	557[M + K]^+^, 541[M + Na]^+^, 519[M + H]^+^, 271[M + H-mal-glu]^+^
75^a^	chrysoeriol 7-*O*-(6″-*O*-malonyl)glucoside	C 7-*O*-(6″-*O*-mal)glu	21.00	C_25_H_24_O_14_	549.1239	0.0	571[M + Na]^+^, 549[M + H]^+^, 301[M + H-mal-glu]^+^
76^c^	luteolin	L	22.02	C_15_H_10_O_6_	287.0550	0.0	287[M + H]^+^
77	formononetin 7-*O*-(6″-*O*-malonyl)glucoside (6″-*O*-malonylononin)	F 7-*O*-(6″-*O*-mal)glu	22.68	C_25_H_24_O_12_	517.1344	0.7	555[M + K]^+^, 539[M + Na]^+^, 517[M + H]^+^, 269[M + H-mal-glu]^+^, 254[M + H-mal-glu-CH_3_]^+^
78^a^	afromosin 7-*O*-(6″-*O*-malonyl)glucoside	A_f_ 7-*O*-(6″-*O*-mal)glu	22.74	C_26_H_26_O_13_	547.1448	0.3	585[M + K]^+^, 569[M + Na]^+^, 547[M + H]^+^, 299[M + H-mal-glu]^+^, 284[M + H-mal-glu-CH_3_]^+^
79^c^	genistein	G_n_	23.54	C_15_H_10_O_5_	271.0601	0.0	271[M + H]^+^
80^c^	apigenin	A_p_	23.68	C_15_H_10_O_5_	271.0600	−0.4	271[M + H]^+^
81^a^	tectorigenin	T	23.82	C_16_H_12_O_6_	301.0710	1.1	323[M + Na]^+^, 301[M + H]^+^, 286[M + H-CH_3_]^+^
82^c^	formononetin	F	24.99	C_16_H_12_O_4_	269.0809	0.2	291[M + Na]^+^, 269[M + H]^+^, 254[M + H-CH_3_]^+^
83	afromosin	A_f_	25.22	C_17_H_14_O_5_	299.0914	0.0	337[M + K]^+^, 321[M + Na]^+^, 299[M + H]^+^, 284[M + H-CH_3_]^+^

All samples analyzed in positive ESI-ionization mode (*m/z* [M + H]^+^) of ToF–MS; [M + Na]^+^ and [M + K]^+^ adduct ions presented. Each peak was tentatively determined by comparing elution order, MS fragmentation and NMR confirmation presented in constructed library.

RT = retention time; DME = dimethyl ester; A_f_ = afromosin; A_p_ = apigenin; C = chrysoeriol; D = daidzein; F = formononetin; G_n_ = genistein; G_y_ = glycitein; I = isorhamnetin; K = kaempferol; L = luteolin; Q = quercetin; T = tectorigenin; api = apiose (132 Da); gal = galactose (162 Da); glu = glucose (162 Da); rham = rhamnose (146 Da); mal = malonyl (86 Da).

a, new flavonoid in soybean leaves; b, newly named; c, further confirmed in comparison with authentic standards.

### Flavonol derivatives (55)

A total of fifty-five flavonol glycosides were mainly composed of **di**-groups [**rham**^**1**^**-gal**^**2**^, **rham**^**1**^**-glu**^**2**^ (neohesperidose, **neo**), **rham**^**1**^**-gal**^**6**^ (robinobiose, **rob**), **rham**^**1**^**-glu**^**6**^ (rutinose, **rut**): 308 Da] [**glu**^**1**^**-gal**^**2**^, **glu**^**1**^**-glu**^**2**^ (sophorose, **sop**), **glu**^**1**^**-gal**^**6**^, **glu**^**1**^**-glu**^**6**^ (gentiobiose, **gen**): 324 Da] and **tri**-groups [**glu**^**1**^**(glu**^**(1)**^**)-gal**^**2(6)**^, **glu**^**1**^**(glu**^**(1)**^**)-glu**^**2(6)**^: 486 Da] [**rham**^**1**^**(glu**^**(1)**^**)-gal**^**2(6)**^, **rham**^**1**^**(glu**^**(1)**^**)-glu**^**2(6)**^, **glu**^**1**^**(rham**^**(1)**^**)-gal**^**2(6)**^, **glu**^**1**^**(rham**^**(1)**^**)-glu**^**2(6)**^: 470 Da] [**rham**^**1**^**(rham**^**(1)**^**)-gal**^**2(6)**^, **rham**^**1**^**(rham**^**(1)**^**)-gal**^**4(6)**^, **rham**^**1**^**(rham**^**(1)**^**)-glu**^**2(6)**^: 454 Da] combined to the 3-OH of kaempferol (**K**, *m/z* 287), quercetin (**Q**, *m/z* 303) and isorhamnetin (**I**, *m/z* 317) (Fig. 1A and Table 1).

The structural profile of twenty-three flavonol **tri**-glycosides (peaks **1–7**, **10**, **12–17**, **21–23**, **27**, **29**, **33**, **34**, **41** and **42**) include the pattern of 26 **di**-glycosides (peaks **8**, **11**, **18**, **20**, **24–26**, **28**, **30–32**, **35**, **37–39**, **43**, **44**, **47**, **51–55**, **58**, **60** and **62**). Six **di**-glycosides ([M + H]^+^, *m/z* 627, 611, 641, based on **Q**, **K** and **I**, respectively) of **glu**^**1**^**-gal**^**2**^ (peaks **8**, **26** and **31**) and **glu**^**1**^**-glu**^**2**^ (**sop**, peaks **11**, **28** and **32**), which were predominant components from some cultivars (**SL**s 7, 10–12, 15 and 21) (Supplementary Fig. S1 and Table 1), presented the fragmentation of [M + H-glu]^+^ and [M + H-glu-gal]^+^ / [M + H-2glu]^+^. In particular, ‘Q 3-*O*-(2″-*O*-glu)**gal**’ (peak **8**) and ‘Q 3-*O*-(2″-*O*-glu)**glu**’ (Q 3-*O*-sop, peak **11**) were consistent with previous reports^14,16^ following the elution order of **gal** (Rt = 11.16 min) > **glu** (Rt = 11.39 min) confirmed after NMR elucidation (Supplementary Fig. S1 and Table S1), and closely related to corresponding **tri**-glycosides (peaks **1**, **2**, **6** and **7**). Peaks **26**, **28** and **31** also determined through interpretation of previous LC–MS and NMR results^14–17,24^, and furthermore, peak **32** was tentatively identified as ‘I 3-*O*-(2″-*O*-glu)glu’ (I 3-*O*-sop) on the basis of above mentioned identical information and reported for the first time from the **SL**s. Peaks **1** and **2** (*m/z* 789[M + H]^+^, 811[M + Na]^+^, 827[M + K]^+^, based on **Q**) corresponding to **glu**^**1**^**(glu**^**(1)**^**)-gal**^**2(6)**^ and **glu**^**1**^**(glu**^**(1)**^**)-glu**^**2(6)**^, respectively, were tentatively identified as ‘Q 3-*O*-(2″,6″-di-*O*-glu)gal’ and ‘Q 3-*O*-(2″,6″-di-*O*-glu)glu’ with fragment ions of *m/z* 627[M + H-glu]^+^, 465[M + H-2glu]^+^ and 303[M + H-2glu-gal]^+^ / [M + H-3glu]^+^. Also, peak **4** (*m/z* 773[M + H]^+^) was found to be ‘K 3-*O*-(2″,6″-di-*O*-glu)glu’ including structure of primary ‘K 3-*O*-(2″-*O*-glu)glu’ (K 3-*O*-sop, peak **28**) and showed similar fragment patterns with peaks **1** and **2**. Three **tri**-glycosides (peaks **1**, **2** and **4**) were reported for the first time in this source.

Peaks **6** and **7** (*m/z* 773[M + H]^+^, based on **Q**) corresponding to above **glu**^**1**^**(rham**^**(1)**^**)-gal**^**2(6)**^ and **glu**^**1**^**(rham**^**(1)**^**)-glu**^**2(6)**^, respectively, were tentatively identified as ‘Q 3-*O*-(2″-*O*-glu-6″-*O*-rham)gal’ and ‘Q 3-*O*-(2″-*O*-glu-6″-*O*-rham)glu’ with fragment ions of *m/z* 627[M + H-rham]^+^, 611[M + H-glu]^+^, 465[M + H-rham-glu]^+^, and 303[M + H-rham-glu-gal]^+^ / [M + H-rham-2glu]^+^, which were predominant components from black coated cultivars (**SL**s 8, 12, 17 and 19) including Cheongja 2 (**SL**3) (Tables 1 and 2). Likewise, ‘K 3-*O*-(2″-*O*-glu-6″-*O*-rham)gal’ (peak **16**) and ‘K 3-*O*-(2″-*O*-glu-6″-*O*-rham)glu’ (peak **21**) with *m/z* 757[M + H]^+^ were highly contained in similar cultivars (**SL**s 6, 9 and 14) with Daewon kong (**SL**2, yellow) and consistent with deglycosidic patterns of peaks **6** and **7**^14–17,20,23,25^. Especially, two **di**-glycosides related to peaks **16** and **21**, ‘K 3-*O*-(2″-*O*-glu)gal’ (peak **26**) and ‘K 3-*O*-(2″-*O*-glu)glu’ (K 3-*O*-sop, peak **28**) were only detected with large amount in **SL**s 7 and 21^14,15,17,24^. Additionally, new **tri**-glycosides, peaks **22** and **23** (*m/z* 787[M + H]^+^, based on **I**) were identified as ‘I 3-*O*-(2″-*O*-glu-6″-*O*-rham)gal’ (named as **soyanin I**) and ‘I 3-*O*-(2″-*O*-glu-6″-*O*-rham)glu’ (named as **soyanin II**) in mainly **SL**s 13 and 19, respectively.

Twelve glycosides of **glu**^**1**^**-gal**^**6**^ (peaks **18**, **30** and **44**) / **glu**^**1**^**-glu**^**6**^ (**gen**, peaks **20**, **39** and **51**) and **rham**^**1**^**(glu**^**(1)**^**)-gal**^**2(6)**^ (peaks **3**, **10** and **12**) / **rham**^**1**^**(glu**^**(1)**^**)-glu**^**2(6)**^ (peaks **5**, **17** and **15**) were closely related to each other and identified simultaneously in **SL**4 unlike other cultivars (Supplementary Fig. S1). Among them, most glycosides were confirmed as new compounds except for K 3-*O*-(2″-*O*-rham-6″-*O*-glu)gal (peak **10**) and K 3-*O*-(6″-*O*-glu)glu (K 3-*O*-gen, peak **39**)^5,14,23,25^. In special, ‘I 3-*O*-(2″-*O*-rham-6″-*O*-glu)gal’ (peak **12**) and ‘I 3-*O*-(2″-*O*-rham-6″-*O*-glu)glu’ (peak **15**) with *m/z* 787[M + H]^+^ were tentatively determined as new **tri**-**IG**s through mass fragmented interpretation.

Twelve **di**-glycosides of **rham**^**1**^**-gal**^**6**^ (**rob**, peaks **35**, **54** and **60**)^5,14,19,21,23,25^ / **rham**^**1**^**-glu**^**6**^ (**rut**, peaks **38**, **58** and **62**)^5,14,19,21^ and **rham**^**1**^**-gal**^**2**^ (peaks **24**, **37** and **47**)^14,16^ / **rham**^**1**^**-glu**^**2**^ (**neo**, peaks **25**, **43** and **55**) were fragmented from the parent ions ([M + H]^+^, *m/z* 611, 595, 625, based on **Q**, **K** and **I**, respectively) to [M + H-rham]^+^ and [M + H-rham-gal]^+^ / [M + H-rham-glu]^+^. Six glycosides belonging to **rham**^**1**^**-gal**^**6**^ and **rham**^**1**^**-glu**^**6**^ described above were evenly distributed in **SL**s 1 (Shinpaldalkong2ho), 4 and 16, while, ‘K 3-*O*-(6″-*O*-rham)gal’ (K 3-*O*-rob, **biorobin**, peak **54**) and ‘K 3-*O*-(6″-*O*-rham)glu’ (K 3-*O*-rut, **nicotiflorin**, peak **58**) were only detected with large amount in **SL**5 (Supplementary Fig. S1). ‘K 3-*O*-(2″-*O*-rham)gal’ (peak **37**) and ‘I 3-*O*-(2″-*O*-rham)gal’ (peak **47**) of **rham**^**1**^**-gal**^**2**^ were confirmed as major constituents, but new glycosides (peaks **25**, **43** and **55**) of **rham**^**1**^**-glu**^**2**^ (**neo**) slightly contained in **SL**18.

Six **tri**-glycosides ([M + H]^+^, *m/z* 757, 741**,** 771, based on **Q**, **K** and **I**, respectively), **rham**^**1**^**(rham**^**(1)**^**)-gal**^**2(6)**^ (peaks **13, 27** and **33**) and **rham**^**1**^**(rham**^**(1)**^**)-gal**^**4(6)**^ (peaks **14**, **29** and **34**) composed of **rham**^**1**^**-gal**^**6**^ (peaks **35**, **54** and **60**) and **rham**^**1**^**-gal**^**2**^ (peaks **24**, **37** and **47**) were fragmented with [M + H-rham]^+^, [M + H-2rham]^+^ and [M + H-2rham-gal]^+^. As major compound from mainly **SL**5, it was reported that ‘K 3-*O*-(2″,6″-di-*O*-rham)gal’ (peak **27**)^5,14–17,23,25^ have significant antioxidant and hepatoprotective activities against carbon tetrachloride-induced liver injury in mice^20^. Especially, peak **42** of **rham**^**1**^**(rham**^**(1)**^**)-glu**^**2(6)**^ with peaks **13**, **33**, and **34** were newly identified from **SL**s 1, 3, 4, 8, 12, 13, 16, 17, 19 and 20, among them, ‘I 3-*O*-(2″,6″-di-*O*-rham)gal’ (peak **33**) largely found as well as closely related to peak **27** presented as major **tri**-**KG**s in Korean representative variety, Shinpaldalkong2ho (**SL**1). Furthermore, peak **33**, ‘I 3-*O*-(4″,6″-di-*O*-rham)gal’ (peak **34**) and ‘I 3-*O*-(2″,6″-di-*O*-rham)glu’ (peak **42**) were termed as **soyanin**s **III, IV** and **V**, respectively (Fig. 3 and Supplementary Fig. S2). Recently, two **tri**-**IG**s (isorhamnetin 3-*O*-rhamnosylrhamnosylglucoside and 3-*O*-rhamnosylrhamnosylgalactoside) were partially characterized by LC–MS, UV spectra and hydrolysis from the leaves of wild Taiwanese *G. max* subsp. *formosana*, but their glycosylated positions have not been determined^21^.

### Flavone (9) and isoflavone (19) derivatives

Among nine flavone derivatives identified as minor compounds, seven glycosides (peaks **48**, **61**, **65**, **67**, **68**, **74** and **75**) were described with combination to the 7-OH of luteolin (**L**, *m/z* 287), apigenin (**A**_**P**_, *m/z* 271) and chrysoeriol (**C**, *m/z* 301) (Fig. 1A and Table 1). Four glycosides (peaks **61**/**68**, **74** and **75**; [M + H]^+^, *m/z* 535, 519, 549, based on **L**, **A**_**p**_ and **C**, respectively) were malonylated with L 7-*O*-glu (**cynaroside**, peak **48**), A_p_ 7-*O*-glu (**cosmosiin**, peak **65**) and C 7-*O*-glu (**thermopsoside**, peak **67**) corresponding to structures confirmed by comparing authentic standards and previous reports^14,21,23,26^. These new malonylated (**mal**) glycosides were tentatively identified as ‘L 7-*O*-(2″-*O*-mal)glu’ (peak **61**), ‘L 7-*O*-(6″-*O*-mal)glu’ (peak **68**), ‘A_p_ 7-*O*-(6″-*O*-mal)glu’ (peak **74**) and ‘C 7-*O*-(6″-*O*-mal)glu’ (peak **75**) with key fragment of [M + H-mal-glu]^+^. Peak **68** was found to be consistent with that isolated from Korean lettuce samples^27^.

From nineteen isoflavone derivatives (5 aglycones and 14 glycosides), the glycosides were presented as structures in which glucose (162 Da; peaks **9**, **19**, **49**, **56** and **72**), malonylglucose (**mal-glu**, 248 Da; peaks **50**, **59**, **69**–**71**, **77** and **78**) and apiosylglucose (**api-glu**, 294 Da; peaks **36** and **45**) combined to the 7-OH or 4'-OH of daidzein (**D**, *m/z* 255; peak **73**), genistein (**G**_**n**_, *m/z* 271; peak **79**), glycitein (**G**_**y**_, *m/z* 285), formononetin (**F**, *m/z* 269; peak **82**), afromosin (**A**_**f**_, *m/z* 299; peak **83**) and tectorigenin (**T**, *m/z* 301; peak **81**) (Fig. 1B and Table 1). Among them, eleven isoflavones^28–30^ corresponding to **aglycones** (peaks **73** and **79**), **7-*O*-glu** (peaks **9**, **19** and **49**), **7-*O*-(6**″**-*O*-mal)glu** (peaks **59** and **70**), **4'-*O*-(6**″**-*O*-mal)glu** (peaks **50** and **69**), **7-*O*-(6**″**-*O*-api)glu** (peak **36**) and **7-*O*-(2**″**-*O*-api)glu** (peak **45**) have already been reported from seeds of soybean cultivars used in the present study. Particularly, peaks **50** and **69** were newly reported as ‘D 4'-*O*-(6″-*O*-mal)glu’ (6″-*O*-malonylisodaidzin; *m/z* 503[M + H]^+^, 255[M + H-mal-glu]^+^) and ‘G_n_ 4'-*O*-(6″-*O*-mal)glu’ (6″-*O*-malonylsophoricoside; *m/z* 519[M + H]^+^, 271[M + H-mal-glu]^+^)^30^ from the **SL**s, respectively, and provided similar fragmentation with the peaks **59** (6″-*O*-malonyldaidzin) and **70** (6″-*O*-malonylgenistin) well-known from soybean seeds and leaves (Supplementary Fig. S1, Supplementary Table S1 and Table 1)^16,23,25,31–35^. Additional peaks **36** and **45** were tentatively identified as new **di**-glycosides of ‘G_n_ 7-*O*-(6″-*O*-api)glu’ (6″-*O*-apiosylgenistin) and ‘G_n_ 7-*O*-(2″-*O*-api)glu’ (2″-*O*-apiosylgenistin) with same *m/z* 565[M + H]^+^, 433[M + H-api]^+^ and 271[M + H-api-glu]^+^, and have also not been reported in the **SL**s yet.

Eight methoxy-isoflavones of **7-*O*-glu** (peaks **56** and **72**) and **7-*O*-(6**″**-*O*-mal)glu** (peaks **71**, **77** and **78**) based on **F**, **A**_**f**_ and **T** ([M + H]^+^, *m/z* 269, 299, 301; peaks **82**, **83** and **81**, respectively) were interestingly developed during the **SL**s growth, and their aglycones indicated certain fragment ion related to methyl (CH_3_, 15 Da) loss in MS positive ionization. Peaks **77** (6″-*O*-malonylononin), **82** (**F**) and **83** (**A**_**f**_) have been studied from the **SL**s by NMR and MS^14,36,37^, while two glycosides close to peaks **72** and **78** were characterized as afromosin *O*-glucoside and *O*-malonylglucoside whose malonylated and glycosylated positions are not determined^38^. Nevertheless, peaks **72** and **78** could be suggested as ‘A_f_ 7-*O*-glu’ (*m/z* 461[M + H]^+^, 299[M + H-glu]^+^, 284[M + H-glu-CH_3_]^+^) and ‘A_f_ 7-*O*-(6″-*O*-mal)glu’ (*m/z* 547[M + H]^+^, 299[M + H-mal-glu]^+^, 284[M + H-mal-glu-CH_3_]^+^) considering isoflavone profiles (elution order, UV spectra and QToF-MS data) presented in roots of *Medicago truncatula*^39^. Besides, peaks **56**, **71** and **81** newly generated from the **SL**s were tentatively identified as ‘T 7-*O*-glu’ (*m/z* 463[M + H]^+^, 301[M + H-glu]^+^, 286[M + H-glu-CH_3_]^+^), ‘T 7-*O*-(6″-*O*-mal)glu’ (*m/z* 549[M + H]^+^, 301[M + H-mal-glu]^+^, 286[M + H-mal-glu-CH_3_]^+^) and ‘**T**’ (*m/z* 301[M + H]^+^, 286[M + H-CH_3_]^+^), respectively, depending on reports of *Stellaria* species belong to the Caryophyllaceae^40^, and necessary to confirm through further NMR studies.

### Quantification of 83 flavonoid derivatives in soybean leaves

**Figure 2:**
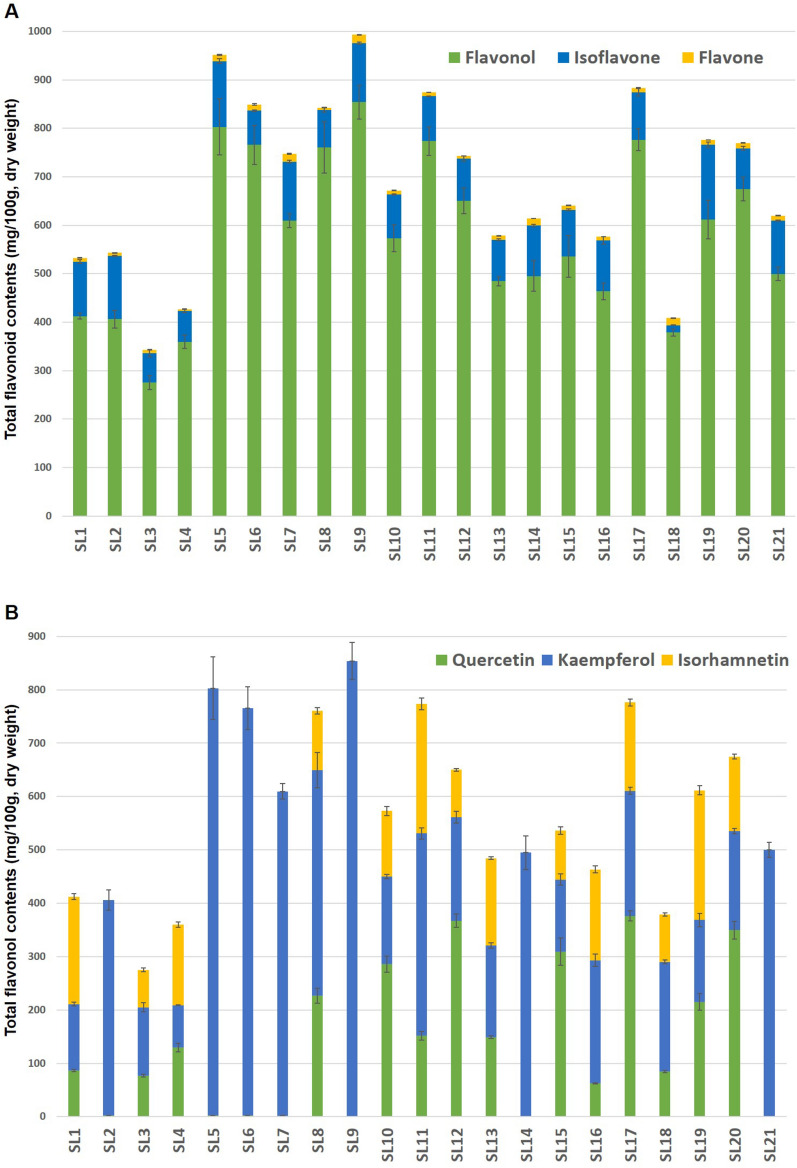

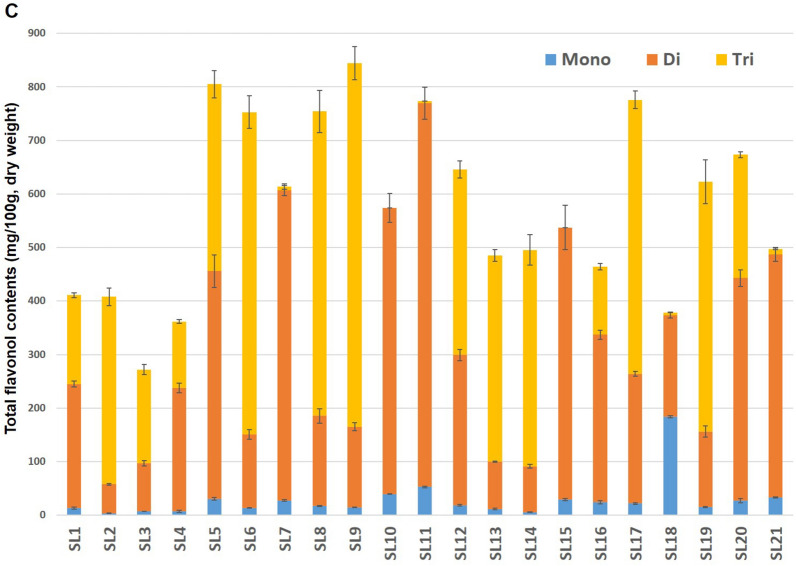
Comparison in total contents (mg/100 g, dry weight) according to flavonoid (**A**) types (flavonol, flavone and isoflavone) as well as flavonol (**B**) aglycones (quercetin, kaempferol and isorhamnetin) and (**C**) glycosides (mono-, di- and tri-) in young leaves of 21 soybean cultivars.

The contents of eighty-three flavonoid derivatives are summarized according to their aglycones and glycosides in Table 2. The total content (mg/100 g, dry weight) of these derivatives ranged from 342.5 to 992.7 (average 684.9) in young leaves of 21 soybean cultivars, and detailed as flavonols (275.1–854.0), flavones (3.6–17.3) and isoflavones (61.2–154.0) (Fig. 2A). These results (mainly flavonols, 83.6%) are consistent with previous reports that the leaf-flavonols (487.3–2280.0) were much higher than seed-isoflavones (240.2–445.2) as well as leaf-isoflavones (91.3–124.3)^5,6,28^. As presented in Fig. 2B,C, the abundant flavonols contained primarily as **di**- (50.4%) and **tri**- (44.0%) glycosidic forms from the **SL**s were distributed in the order of **K** (79.7–853.5, 57.5%), **Q** (1.6–376.3, 23.9%) and **I** (70.0–243.2, 18.6%) according to their aglycone types, and had different predominant aglycones under affected by the cultivar’s characteristics. Among eleven cultivars belonging to **K**-rich **SL**s (with yellow-coated seeds), the **SL**s 2, 5, 6, 7, 9, 14 and 21 were composed of about 100% **KG**s^14,15^. In particular, the **SL**s 7 (Kongnamulkong, Korean landrace for bean sprouts) and 21 (Himeyudaga, Japanese breeding line) showed the largest proportion of **di**-glycosides (94.7 and 91.3%), while the **SL**s 6 (Kongnamulkong, Korean landrace for bean sprouts) and 9 (Nongrim 51, Japanese breeding line) were expected to be superior cultivars due to their higher total flavonols (**TF**s; 765.6 and 854.0) with **tri**-glycosides levels (79.8 and 80.1%), respectively. In addition, the **Q**-rich **SL**17 (CS 02,028, Korean landrace) with **QG**s and **TF**s (48.5% and 776.3) possessed much higher **tri**-glycosides (65.3%) compared to the **SL**s 10 (49.9 and 93.1) and 15 (57.7 and 94.6) with **QG**s (%) and **di**-glycosides (%), respectively. Interestingly, despite the low **TF**s (359.6) of **SL**4 (PI 90,763, Chinese landrace), its flavonol profile mostly composed of new glycosides such as **rham**^**1**^**(glu**^**(1)**^**)-gal**^**2(6)**^ (peaks **3**, **10** and **12**) and

**Table 2:** Contents of flavonoid derivatives according to the aglycones and glycosides in young leaves of 21 soybean cultivars (mg/100 g, dry weight).

Glycosides	Peak No	Shinpaldal2ho (SL1)	Daewon kong (SL2)	Cheongja 2 (SL3)	SL4	SL5	SL6	SL7	SL8	SL9	SL10	SL11	SL12	SL13	SL14	SL15	SL16	SL17	SL18	SL19	SL20	SL21
***Flavonols (55)***																						
***Quercetin derivatives (18)***																						
Mono	40	ND	ND	1.1 ± 0.1	1.5 ± 0.1	ND	ND	ND	1.1 ± 0.3	ND	7.2 ± 0.2	4.8 ± 0.7	4.6 ± 0.6	1.2 ± 0.3	ND	7.1 ± 0.6	0.8 ± 0.3	6.3 ± 0.8	13.0 ± 1.1	0.8 ± 0.4	6.0 ± 0.0	ND
	46	2.0 ± 0.3	ND	1.4 ± 0.3	1.8 ± 0.1	ND	ND	ND	3.7 ± 0.2	ND	13.0 ± 0.2	8.4 ± 0.4	6.8 ± 0.4	2.1 ± 0.2	ND	11.5 ± 1.0	2.1 ± 0.4	6.1 ± 0.5	42.0 ± 1.1	3.9 ± 0.9	7.6 ± 0.0	ND
Di	8	1.4 ± 0.3	ND	10.0 ± 0.2	ND	0.4 ± 0.1	0.6 ± 0.0	1.1 ± 0.3	10.6 ± 0.3	ND	106.9 ± 6.4	58.1 ± 2.5	32.1 ± 0.6	4.7 ± 0.6	ND	113.9 ± 11.3	2.4 ± 0.4	12.2 ± 0.4	2.3 ± 0.5	8.1 ± 0.5	2.1 ± 0.1	ND
	11	1.0 ± 0.1	ND	10.3 ± 0.2	ND	ND	0.3 ± 0.0	0.6 ± 0.1	11.6 ± 0.6	ND	146.1 ± 8.2	72.7 ± 5.9	48.2 ± 0.4	3.5 ± 0.2	ND	164.9 ± 12.1	2.4 ± 0.6	13.3 ± 0.4	4.3 ± 0.6	7.2 ± 0.6	2.3 ± 0.3	ND
	18^a^	ND	ND	ND	11.2 ± 0.2	ND	ND	ND	ND	ND	ND	ND	ND	ND	ND	ND	ND	ND	ND	ND	ND	ND
	20^a^	ND	ND	ND	27.2 ± 0.2	ND	ND	ND	ND	ND	ND	ND	ND	ND	ND	ND	ND	ND	1.1 ± 0.1	ND	ND	ND
	24	ND	ND	ND	ND	ND	ND	ND	ND	ND	11.5 ± 0.8	7.2 ± 0.6	12.5 ± 2.9	ND	ND	10.5 ± 1.2	ND	ND	17.1 ± 0.6	ND	ND	ND
	25^a^	ND	ND	ND	ND	ND	ND	ND	ND	ND	1.4 ± 0.2	ND	ND	8.9 ± 0.7	ND	1.5 ± 0.3	ND	ND	4.7 ± 0.5	ND	ND	ND
	35	18.6 ± 1.1	ND	2.8 ± 0.3	16.2 ± 3.2	ND	ND	ND	10.1 ± 1.1	ND	ND	ND	19.6 ± 1.4	9.7 ± 0.4	ND	ND	15.3 ± 1.5	29.9 ± 0.3	ND	12.6 ± 1.8	61.9 ± 4.8	ND
	38	41.5 ± 1.7	ND	3.6 ± 0.3	36.8 ± 1.2	0.7 ± 0.1	ND	ND	18.0 ± 1.2	ND	ND	ND	45.3 ± 0.7	ND	ND	ND	28.3 ± 0.7	65.6 ± 1.4	ND	23.2 ± 0.6	176.7 ± 9.1	ND
Tri	1^a^	ND	ND	ND	2.1 ± 0.5	ND	ND	ND	ND	ND	ND	ND	ND	ND	ND	ND	ND	ND	ND	ND	ND	ND
	2^a^	ND	ND	ND	2.1 ± 0.9	ND	ND	ND	ND	ND	ND	ND	ND	ND	ND	ND	ND	ND	ND	ND	ND	ND
	3^a^	ND	ND	ND	13.3 ± 0.3	ND	ND	ND	ND	ND	ND	ND	ND	ND	ND	ND	ND	ND	ND	ND	ND	ND
	5^a^	ND	ND	ND	1.4 ± 0.3	ND	ND	ND	ND	ND	ND	ND	ND	ND	ND	ND	ND	ND	ND	ND	ND	ND
	6	2.0 ± 0.3	1.3 ± 0.0	22.3 ± 1.2	ND	0.6 ± 0.1	0.6 ± 0.1	ND	69.9 ± 5.6	0.5 ± 0.1	ND	ND	76.3 ± 4.6	56.5 ± 1.7	ND	ND	1.5 ± 0.2	85.8 ± 3.7	ND	59.4 ± 7.2	4.0 ± 0.0	ND
	7	1.0 ± 0.2	1.5 ± 0.1	21.3 ± 0.7	1.1 ± 0.1	0.7 ± 0.1	0.5 ± 0.2	ND	86.0 ± 6.1	ND	ND	ND	101.8 ± 5.7	46.3 ± 1.5	ND	ND	1.0 ± 0.2	113.8 ± 4.6	ND	80.6 ± 7.0	5.3 ± 0.2	ND
	13^a^	17.4 ± 0.3	ND	4.0 ± 0.1	13.8 ± 0.2	ND	ND	ND	15.1 ± 1.0	ND	ND	ND	18.3 ± 0.4	15.1 ± 0.6	ND	ND	8.5 ± 0.5	41.0 ± 0.7	ND	18.1 ± 1.3	78.2 ± 0.4	ND
	14	1.2 ± 0.1	ND	ND	0.9 ± 0.0	ND	ND	ND	0.8 ± 0.1	ND	ND	ND	1.6 ± 0.1	0.7 ± 0.1	ND	ND	ND	2.4 ± 0.2	ND	1.3 ± 0.1	5.4 ± 0.3	ND
**Subtotal**		**86.2 ± 2.2**	**2.8 ± 0.1**	**76.6 ± 2.6**	**129.3 ± 8.1**	**2.3 ± 0.1**	**2.0 ± 0.3**	**1.6 ± 0.4**	**226.9 ± 14.2**	**0.5 ± 0.1**	**286.0 ± 15.2**	**151.3 ± 7.9**	**367.2 ± 12.8**	**148.7 ± 2.4**	**ND**	**309.4 ± 25.6**	**62.2 ± 0.9**	**376.3 ± 9.7**	**84.5 ± 2.1**	**215.2 ± 15.2**	**349.6 ± 16.3**	**ND**
***Kaempferol derivatives (20)***																						
Mono	57^a^	1.4 ± 0.1	2.1 ± 0.1	1.5 ± 0.1	0.5 ± 0.1	15.1 ± 1.2	7.9 ± 0.2	15.8 ± 0.7	4.8 ± 0.2	8.4 ± 0.4	8.3 ± 0.4	14.8 ± 0.7	2.1 ± 0.0	2.4 ± 0.0	3.4 ± 0.2	3.9 ± 0.4	3.8 ± 0.1	2.5 ± 0.1	34.4 ± 0.4	1.5 ± 0.1	2.5 ± 0.3	20.7 ± 0.8
	63	1.5 ± 0.3	1.4 ± 0.0	1.1 ± 0.1	1.0 ± 0.2	15.4 ± 1.2	5.5 ± 0.4	11.8 ± 1.0	4.0 ± 0.6	6.2 ± 0.4	3.2 ± 0.1	8.3 ± 0.2	2.3 ± 0.5	0.8 ± 0.1	2.3 ± 0.1	2.2 ± 0.2	5.0 ± 1.2	2.5 ± 0.6	65.8 ± 0.3	1.3 ± 0.4	2.8 ± 0.6	12.4 ± 0.6
Di	26	1.3 ± 0.3	12.9 ± 0.3	17.4 ± 0.1	ND	ND	49.7 ± 0.2	303.6 ± 9.8	31.2 ± 6.0	56.5 ± 2.5	69.8 ± 2.5	179.0 ± 5.0	21.9 ± 0.6	6.7 ± 0.5	26.8 ± 3.6	61.1 ± 6.5	3.7 ± 0.9	7.3 ± 1.3	7.2 ± 0.3	7.6 ± 0.8	9.1 ± 0.4	258.0 ± 11.5
	28	ND	5.4 ± 0.2	12.1 ± 0.7	ND	ND	24.7 ± 0.4	218.8 ± 6.5	17.7 ± 1.0	31.1 ± 0.5	55.6 ± 2.9	126.4 ± 4.3	18.0 ± 0.7	2.3 ± 0.1	15.8 ± 0.1	54.2 ± 3.6	4.6 ± 1.0	4.7 ± 0.2	6.8 ± 0.5	3.5 ± 0.3	2.0 ± 0.2	192.5 ± 8.6
	30^a^	ND	ND	ND	9.1 ± 0.5	ND	ND	ND	ND	ND	ND	ND	ND	ND	ND	ND	ND	ND	0.4 ± 0.2	ND	ND	ND
	37	0.5 ± 0.1	0.5 ± 0.0	1.4 ± 0.1	ND	5.0 ± 0.2	2.8 ± 0.1	45.0 ± 2.7	1.9 ± 0.0	4.4 ± 0.2	24.8 ± 1.0	42.6 ± 1.1	8.7 ± 0.3	0.9 ± 0.1	2.0 ± 0.1	11.3 ± 0.9	1.4 ± 0.2	0.7 ± 0.1	64.7 ± 2.7	0.6 ± 0.1	0.4 ± 0.0	1.7 ± 0.1
	39	ND	ND	ND	11.4 ± 1.1	0.8 ± 0.1	ND	ND	ND	ND	ND	ND	ND	ND	ND	ND	ND	ND	ND	ND	ND	ND
	43^a^	ND	ND	ND	ND	1.0 ± 0.2	0.5 ± 0.1	6.4 ± 0.3	ND	0.8 ± 0.2	1.7 ± 0.1	5.7 ± 0.6	2.3 ± 0.1	ND	0.4 ± 0.1	1.3 ± 0.1	ND	ND	23.2 ± 2.0	ND	ND	ND
	54	31.8 ± 0.8	18.5 ± 0.9	5.3 ± 0.1	13.2 ± 0.5	171.4 ± 13.3	31.5 ± 1.4	ND	22.2 ± 1.0	29.5 ± 1.5	ND	ND	16.7 ± 0.3	12.8 ± 0.4	20.0 ± 0.7	ND	71.2 ± 2.7	28.8 ± 0.8	ND	9.6 ± 0.6	41.0 ± 1.9	ND
	58	41.3 ± 2.6	16.8 ± 0.6	4.8 ± 0.2	16.9 ± 0.9	245.6 ± 17.6	31.1 ± 1.0	ND	19.2 ± 1.4	32.1 ± 1.2	ND	ND	17.0 ± 0.8	6.8 ± 0.2	19.7 ± 0.5	ND	73.9 ± 4.3	33.7 ± 1.3	ND	9.6 ± 0.4	62.7 ± 0.9	ND
	52^a^	ND	ND	0.2 ± 0.0	ND	ND	ND	1.3 ± 0.5	ND	ND	0.6 ± 0.1	ND	ND	ND	ND	0.6 ± 0.0	ND	ND	ND	ND	ND	2.6 ± 0.6
	53^a^	ND	ND	ND	ND	ND	ND	0.7 ± 0.2	ND	ND	0.2 ± 0.1	ND	ND	ND	ND	0.4 ± 0.0	ND	ND	ND	ND	ND	1.6 ± 0.4
Tri	4^a^	ND	ND	ND	2.4 ± 0.4	ND	0.7 ± 0.0	3.6 ± 0.2	ND	1.5 ± 0.1	ND	2.9 ± 0.3	ND	ND	ND	ND	ND	ND	ND	ND	ND	10.2 ± 1.5
	10	ND	ND	ND	9.9 ± 0.5	0.6 ± 0.0	ND	1.0 ± 0.1	ND	ND	ND	ND	ND	ND	ND	ND	ND	ND	3.0 ± 0.3	ND	ND	ND
	16	2.4 ± 0.4	175.9 ± 9.0	44.5 ± 2.4	ND	7.0 ± 0.2	303.5 ± 5.8	ND	151.1 ± 5.5	298.6 ± 15.9	ND	ND	46.2 ± 1.7	65.7 ± 3.3	191.5 ± 13.5	ND	42.2 ± 0.2	57.4 ± 1.9	ND	52.0 ± 4.9	4.8 ± 0.2	ND
	17^a^	ND	ND	ND	1.2 ± 0.2	ND	ND	ND	ND	ND	ND	ND	ND	ND	ND	ND	ND	ND	ND	ND	ND	ND
	21	1.2 ± 0.2	116.7 ± 7.7	28.8 ± 0.9	ND	3.5 ± 0.3	198.8 ± 9.3	ND	104.0 ± 4.8	226.3 ± 12.3	ND	ND	38.4 ± 0.8	32.6 ± 0.7	141.5 ± 6.3	ND	1.6 ± 0.1	44.0 ± 0.5	ND	40.6 ± 2.9	3.6 ± 0.3	ND
	27	39.7 ± 2.2	49.3 ± 2.2	10.3 ± 0.2	12.9 ± 0.6	307.2 ± 24.6	99.6 ± 1.3	ND	60.9 ± 2.3	146.6 ± 4.6	ND	ND	18.7 ± 1.5	39.9 ± 0.9	64.7 ± 10.0	ND	57.3 ± 4.6	49.4 ± 2.6	ND	25.6 ± 2.7	51.3 ± 2.0	ND
	29	4.0 ± 0.2	3.0 ± 0.2	0.8 ± 0.2	1.3 ± 0.1	28.2 ± 1.1	5.9 ± 0.1	ND	2.7 ± 0.3	9.0 ± 0.6	ND	ND	1.8 ± 0.5	1.3 ± 0.2	4.4 ± 0.3	ND	4.0 ± 0.1	3.0 ± 0.2	ND	1.4 ± 0.3	5.4 ± 0.1	ND
	41^a^	ND	0.9 ± 0.4	0.3 ± 0.0	ND	ND	1.4 ± 0.6	ND	2.7 ± 0.1	2.6 ± 0.5	ND	ND	ND	ND	2.4 ± 0.1	ND	ND	ND	ND	ND	ND	ND
**Subtotal**		**125.1 ± 3.7**	**403.5 ± 19.0**	**128.4 ± 8.3**	**79.7 ± 0.6**	**800.7 ± 58.2**	**763.7 ± 40.0**	**607.9 ± 14.8**	**422.4 ± 33.3**	**853.5 ± 34.7**	**164.1 ± 4.3**	**379.5 ± 10.4**	**194.0 ± 11.2**	**172.1 ± 4.6**	**495.0 ± 31.5**	**135.0 ± 10.3**	**230.8 ± 11.5**	**234.1 ± 6.4**	**205.6 ± 3.7**	**153.4 ± 12.4**	**185.6 ± 4.7**	**499.7 ± 13.9**
***Isorhamnetin derivatives (17)***																						
Mono	64^a^	6.4 ± 1.4	ND	0.9 ± 0.2	1.5 ± 0.2	ND	ND	ND	2.0 ± 0.3	ND	3.4 ± 0.1	7.5 ± 0.1	1.7 ± 0.1	2.5 ± 0.8	ND	1.8 ± 0.1	7.0 ± 2.6	2.9 ± 0.4	13.3 ± 1.7	3.0 ± 0.5	5.1 ± 1.1	ND
	66^a^	2.8 ± 0.1	ND	1.2 ± 0.2	1.1 ± 0.0	ND	ND	ND	1.4 ± 0.1	ND	4.4 ± 0.4	8.8 ± 0.6	1.2 ± 0.1	2.0 ± 0.2	ND	2.5 ± 0.0	5.0 ± 0.0	2.2 ± 0.3	15.5 ± 0.7	3.1 ± 0.4	2.8 ± 0.3	ND
Di	31	ND	ND	8.5 ± 0.2	ND	ND	ND	ND	8.6 ± 0.7	ND	45.8 ± 4.1	103.8 ± 4.9	8.0 ± 1.0	6.0 ± 0.2	ND	33.4 ± 1.7	1.6 ± 0.0	7.0 ± 1.0	1.2 ± 0.3	13.2 ± 1.5	ND	ND
	32^a^	ND	ND	5.5 ± 0.2	ND	ND	ND	ND	4.3 ± 0.7	ND	40.5 ± 3.1	65.3 ± 3.3	6.1 ± 0.4	1.9 ± 0.3	ND	33.1 ± 5.0	0.6 ± 0.0	4.4 ± 0.1	ND	5.4 ± 0.4	ND	ND
	44^a^	ND	ND	ND	13.0 ± 0.6	ND	ND	ND	ND	ND	ND	ND	ND	ND	ND	ND	ND	ND	ND	ND	ND	ND
	47	1.8 ± 0.1	ND	3.3 ± 0.3	ND	ND	ND	ND	2.6 ± 0.3	ND	27.7 ± 1.7	54.6 ± 3.6	12.4 ± 0.6	1.4 ± 0.2	ND	20.9 ± 1.7	3.0 ± 0.6	3.0 ± 0.5	54.9 ± 1.9	5.3 ± 1.3	2.4 ± 0.1	ND
	51^a^	ND	ND	ND	22.4 ± 1.1	ND	ND	ND	ND	ND	ND	ND	ND	ND	ND	ND	ND	ND	ND	ND	ND	ND
	55^a^	ND	ND	ND	ND	ND	ND	ND	ND	ND	1.1 ± 0.1	1.6 ± 0.0	ND	ND	ND	ND	ND	ND	1.5 ± 0.0	ND	ND	ND
	60	44.3 ± 0.8	ND	3.9 ± 0.1	23.0 ± 0.7	ND	ND	ND	6.7 ± 0.3	ND	ND	ND	6.9 ± 0.0	13.6 ± 0.4	ND	ND	47.8 ± 1.5	15.3 ± 0.1	ND	18.4 ± 1.4	24.7 ± 0.2	ND
	62	48.8 ± 0.1	ND	2.6 ± 0.2	29.4 ± 1.8	ND	ND	ND	5.1 ± 0.2	ND	ND	ND	7.0 ± 0.2	8.7 ± 0.1	ND	ND	56.6 ± 1.1	17.2 ± 0.7	ND	16.1 ± 1.0	32.3 ± 0.4	ND
Tri	12^a^	ND	ND	ND	4.8 ± 0.1	ND	ND	ND	ND	ND	ND	ND	ND	ND	ND	ND	ND	ND	1.1 ± 0.4	ND	ND	ND
	15^a^	ND	ND	ND	17.9 ± 0.5	ND	ND	ND	ND	ND	ND	1.2 ± 0.2	ND	ND	ND	ND	ND	ND	1.1 ± 0.1	ND	ND	ND
	22^a,b^	1.2 ± 0.0	ND	10.2 ± 1.0	ND	ND	ND	ND	25.5 ± 2.6	ND	ND	ND	16.8 ± 0.5	21.6 ± 0.6	ND	ND	0.3 ± 0.1	26.2 ± 0.6	ND	14.7 ± 3.8	1.1 ± 0.1	ND
	23^a,b^	ND	ND	22.2 ± 0.6	ND	ND	ND	ND	29.3 ± 0.8	ND	ND	ND	13.1 ± 1.2	66.3 ± 3.4	ND	ND	1.1 ± 0.3	36.4 ± 1.8	ND	70.5 ± 2.5	2.0 ± 0.3	ND
	33^a,b^	31.8 ± 0.3	ND	3.8 ± 0.2	12.8 ± 0.7	ND	ND	ND	6.4 ± 0.0	ND	ND	ND	6.3 ± 0.4	8.5 ± 0.2	ND	ND	15.5 ± 0.7	16.7 ± 1.5	ND	19.1 ± 2.1	23.9 ± 0.4	ND
	34^a,b^	60.5 ± 2.2	ND	7.4 ± 0.3	23.5 ± 1.3	ND	ND	ND	18.0 ± 0.9	ND	ND	ND	8.6 ± 0.7	28.7 ± 0.5	ND	ND	28.8 ± 1.1	31.3 ± 1.7	ND	45.9 ± 2.5	41.0 ± 1.6	ND
	42^a,b^	3.6 ± 0.6	ND	0.6 ± 0.1	1.2 ± 0.4	ND	ND	ND	1.2 ± 0.1	ND	ND	ND	0.7 ± 0.1	2.5 ± 0.5	ND	ND	3.3 ± 0.8	3.3 ± 0.2	ND	1.5 ± 0.0	4.2 ± 0.5	ND
**Subtotal**		**201.3 ± 5.4**	**ND**	**70.0 ± 3.3**	**150.6 ± 5.2**	**ND**	**ND**	**ND**	**111.0 ± 5.8**	**ND**	**122.8 ± 8.7**	**242.7 ± 11.1**	**88.7 ± 2.8**	**163.6 ± 2.9**	**ND**	**91.7 ± 7.3**	**170.6 ± 6.2**	**165.9 ± 6.5**	**88.7 ± 3.2**	**243.2 ± 8.1**	**139.5 ± 4.7**	**ND**
**Flavonols**		**412.5 ± 5.9**	**406.2 ± 18.0**	**275.1 ± 14.1**	**359.6 ± 13.7**	**803.0 ± 58.2**	**765.6 ± 39.9**	**609.5 ± 14.5**	**760.3 ± 53.3**	**854.0 ± 34.8**	**572.9 ± 27.8**	**773.5 ± 29.3**	**649.9 ± 26.4**	**484.5 ± 9.6**	**495.0 ± 31.5**	**536.1 ± 43.1**	**463.6 ± 17.3**	**776.3 ± 22.3**	**378.8 ± 7.3**	**611.8 ± 39.4**	**674.8 ± 24.2**	**499.7 ± 13.9**
***Flavones (9)***																						
***Luteolin derivatives (4)***																						
	76	2.0 ± 0.2	0.2 ± 0.0	2.5 ± 0.1	1.1 ± 0.1	ND	ND	ND	2.3 ± 0.2	ND	4.3 ± 0.7	3.8 ± 0.3	2.1 ± 0.1	4.9 ± 0.4	ND	2.8 ± 0.1	2.0 ± 0.2	3.0 ± 0.2	5.3 ± 0.5	2.5 ± 0.1	4.7 ± 0.4	ND
	48	0.7 ± 0.0	ND	ND	ND	ND	ND	ND	0.7 ± 0.3	ND	ND	ND	ND	ND	ND	ND	0.4 ± 0.0	0.3 ± 0.1	ND	1.1 ± 0.0	0.6 ± 0.1	ND
	61	ND	ND	ND	ND	0.6 ± 0.3	ND	ND	ND	ND	0.5 ± 0.1	ND	ND	ND	ND	0.5 ± 0.1	ND	ND	1.2 ± 0.3	ND	ND	ND
	68^a^	0.9 ± 0.2	ND	0.6 ± 0.1	ND	ND	ND	ND	ND	ND	ND	1.3 ± 0.5	0.7 ± 0.0	ND	ND	1.6 ± 0.2	0.5 ± 0.0	1.0 ± 0.1	1.7 ± 0.3	1.6 ± 0.1	1.3 ± 0.1	ND
**Subtotal**		**3.6 ± 0.2**	**0.2 ± 0.0**	**3.1 ± 0.2**	**1.1 ± 0.1**	**0.6 ± 0.3**	**ND**	**ND**	**3.0 ± 0.4**	**ND**	**4.8 ± 0.8**	**5.1 ± 0.3**	**2.8 ± 0.1**	**4.9 ± 0.4**	**ND**	**4.9 ± 0.1**	**2.9 ± 0.1**	**4.4 ± 0.3**	**8.2 ± 0.9**	**5.3 ± 0.3**	**6.6 ± 0.5**	**ND**
***Apigenin derivatives (3)***																						
	80	0.7 ± 0.1	2.2 ± 0.1	1.1 ± 0.0	0.7 ± 0.1	7.6 ± 0.7	8.3 ± 0.5	8.1 ± 0.8	0.8 ± 0.0	12.1 ± 1.0	1.1 ± 0.0	1.4 ± 0.1	0.7 ± 0.1	0.8 ± 0.0	4.3 ± 0.2	0.7 ± 0.1	1.6 ± 0.1	1.0 ± 0.1	1.5 ± 0.1	1.0 ± 0.1	1.3 ± 0.1	5.8 ± 0.5
	65	ND	0.6 ± 0.0	ND	ND	1.6 ± 0.1	2.7 ± 0.2	3.6 ± 0.1	ND	2.7 ± 0.1	ND	ND	ND	ND	2.6 ± 0.1	ND	ND	ND	ND	ND	ND	1.6 ± 0.0
	74^a^	0.4 ± 0.1	3.0 ± 0.1	ND	ND	3.0 ± 0.3	1.9 ± 0.1	4.3 ± 0.4	ND	2.5 ± 0.1	ND	ND	0.2 ± 0.0	ND	7.2 ± 0.2	ND	0.8 ± 0.1	0.5 ± 0.0	1.2 ± 0.1	0.8 ± 0.2	0.9 ± 0.0	3.4 ± 0.1
**Subtotal**		**1.1 ± 0.2**	**5.8 ± 0.2**	**1.1 ± 0.0**	**0.7 ± 0.1**	**12.2 ± 1.0**	**12.9 ± 1.6**	**16.0 ± 1.2**	**0.8 ± 0.0**	**17.3 ± 0.9**	**1.1 ± 0.0**	**1.4 ± 0.1**	**0.9 ± 0.1**	**0.8 ± 0.0**	**14.2 ± 0.2**	**0.7 ± 0.1**	**2.4 ± 0.3**	**1.5 ± 0.1**	**2.6 ± 0.2**	**1.7 ± 0.2**	**2.2 ± 0.1**	**10.7 ± 0.5**
***Chrysoeriol derivatives (2)***																						
	67	1.2 ± 0.0	ND	0.5 ± 0.1	1.1 ± 0.1	ND	ND	ND	ND	ND	0.8 ± 0.0	0.6 ± 0.1	ND	0.9 ± 0.1	ND	1.0 ± 0.1	0.9 ± 0.0	0.6 ± 0.1	1.0 ± 0.1	1.2 ± 0.2	0.9 ± 0.1	ND
	75^a^	1.9 ± 0.1	ND	1.6 ± 0.1	0.8 ± 0.1	ND	ND	ND	1.1 ± 0.1	ND	1.4 ± 0.1	1.2 ± 0.0	1.1 ± 0.1	1.4 ± 0.1	ND	2.4 ± 0.2	1.4 ± 0.1	1.6 ± 0.0	2.6 ± 0.1	2.0 ± 0.1	1.4 ± 0.0	ND
**Subtotal**		**3.1 ± 0.1**	**ND**	**2.1 ± 0.2**	**1.9 ± 0.2**	**ND**	**ND**	**ND**	**1.1 ± 0.1**	**ND**	**2.2 ± 0.1**	**1.8 ± 0.1**	**1.1 ± 0.1**	**2.3 ± 0.1**	**ND**	**3.4 ± 0.3**	**2.3 ± 0.1**	**2.2 ± 0.4**	**3.6 ± 0.1**	**3.2 ± 0.2**	**2.3 ± 0.1**	**ND**
**Flavones**		**7.9 ± 0.3**	**3.6 ± 0.2**	**6.3 ± 0.1**	**3.6 ± 0.2**	**12.9 ± 1.0**	**12.9 ± 1.6**	**16.0 ± 1.2**	**4.9 ± 0.3**	**17.3 ± 0.9**	**8.0 ± 0.8**	**8.3 ± 0.3**	**4.8 ± 0.2**	**8.1 ± 0.2**	**14.2 ± 0.2**	**9.0 ± 0.5**	**7.6 ± 0.3**	**8.1 ± 0.8**	**14.4 ± 0.7**	**10.3 ± 0.4**	**11.0 ± 1.0**	**10.7 ± 0.5**
***Isoflavones (19)***																						
***Daidzein derivatives (4)***																						
	73	1.1 ± 0.2	6.9 ± 0.3	0.6 ± 0.3	0.4 ± 0.1	0.5 ± 0.0	0.5 ± 0.1	0.9 ± 0.1	0.6 ± 0.0	1.0 ± 0.1	0.5 ± 0.1	1.0 ± 0.1	2.3 ± 0.2	1.1 ± 0.1	0.6 ± 0.0	ND	1.6 ± 0.2	0.8 ± 0.0	0.7 ± 0.0	2.2 ± 0.7	0.5 ± 0.2	1.1 ± 0.3
	9	ND	2.5 ± 0.5	1.0 ± 0.1	0.6 ± 0.3	1.5 ± 0.4	1.1 ± 0.1	1.1 ± 0.1	1.0 ± 0.1	1.9 ± 0.2	5.8 ± 1.6	5.0 ± 0.3	1.2 ± 0.5	1.0 ± 0.3	0.9 ± 0.1	0.6 ± 0.1	1.3 ± 0.1	0.7 ± 0.1	0.4 ± 0.2	1.2 ± 0.2	0.6 ± 0.1	ND
	50^a^	ND	ND	ND	ND	ND	ND	ND	ND	ND	ND	0.3 ± 0.1	ND	ND	ND	ND	ND	ND	ND	ND	ND	ND
	59	4.6 ± 0.3	11.0 ± 0.3	1.1 ± 0.0	1.2 ± 0.7	9.1 ± 0.6	2.2 ± 0.9	3.7 ± 0.1	2.0 ± 0.1	4.5 ± 1.4	2.6 ± 0.3	9.5 ± 0.6	3.7 ± 0.4	2.5 ± 0.3	2.7 ± 0.1	2.1 ± 0.2	6.6 ± 0.8	3.5 ± 0.1	4.5 ± 0.1	4.0 ± 0.4	2.5 ± 0.5	2.4 ± 0.2
**Subtotal**		**5.7 ± 0.1**	**20.5 ± 1.0**	**2.7 ± 0.5**	**2.1 ± 0.7**	**11.2 ± 0.4**	**3.8 ± 0.7**	**5.8 ± 0.2**	**3.6 ± 0.0**	**7.4 ± 1.1**	**8.9 ± 2.0**	**15.8 ± 0.8**	**7.3 ± 0.3**	**4.6 ± 1.7**	**4.2 ± 1.2**	**2.7 ± 0.3**	**9.5 ± 0.4**	**4.9 ± 0.4**	**5.6 ± 0.4**	**7.4 ± 0.5**	**3.6 ± 0.8**	**3.5 ± 0.3**
***Glycitein derivatives (1)***																						
	19	1.4 ± 0.3	ND	ND	ND	ND	ND	ND	ND	ND	ND	ND	ND	ND	ND	ND	ND	ND	ND	ND	ND	ND
**Subtotal**		**1.4 ± 0.3**	**ND**	**ND**	**ND**	**ND**	**ND**	**ND**	**ND**	**ND**	**ND**	**ND**	**ND**	**ND**	**ND**	**ND**	**ND**	**ND**	**ND**	**ND**	**ND**	**ND**
***Genistein derivatives (6)***																						
	79	4.7 ± 0.3	10.5 ± 0.1	4.7 ± 0.2	3.7 ± 0.2	4.1 ± 0.5	3.7 ± 0.1	4.9 ± 0.7	2.3 ± 0.2	4.2 ± 0.3	2.3 ± 0.4	1.0 ± 0.1	5.9 ± 0.3	8.1 ± 0.7	6.1 ± 0.9	2.6 ± 0.2	5.5 ± 0.2	2.7 ± 0.3	2.2 ± 0.2	5.5 ± 0.6	3.7 ± 0.4	3.8 ± 0.4
	49	39.2 ± 1.5	21.3 ± 0.2	8.0 ± 0.7	19.1 ± 5.8	33.5 ± 1.8	39.1 ± 2.0	44.5 ± 1.7	23.2 ± 0.6	54.5 ± 2.4	10.7 ± 4.1	17.2 ± 2.4	13.5 ± 4.8	23.9 ± 2.7	17.9 ± 0.4	7.2 ± 0.5	33.6 ± 2.5	28.4 ± 3.0	12.5 ± 0.6	47.1 ± 7.0	17.3 ± 3.0	16.3 ± 0.7
	36^a^	ND	ND	ND	1.3 ± 0.4	ND	ND	ND	ND	ND	ND	ND	ND	ND	ND	ND	ND	ND	ND	ND	ND	ND
	45^a^	1.3 ± 0.4	ND	ND	1.1 ± 0.2	ND	1.0 ± 0.2	ND	ND	8.7 ± 1.2	ND	ND	ND	ND	ND	ND	ND	0.3 ± 0.0	0.4 ± 0.0	1.0 ± 0.2	0.8 ± 0.3	ND
	69^a^	2.5 ± 0.4	3.3 ± 0.0	2.0 ± 0.5	1.1 ± 0.0	4.4 ± 0.5	1.0 ± 0.2	2.6 ± 0.3	2.0 ± 0.5	2.1 ± 0.6	3.0 ± 0.2	2.8 ± 0.1	2.5 ± 0.5	2.5 ± 0.3	3.5 ± 0.4	3.6 ± 0.3	2.1 ± 0.5	2.7 ± 0.5	2.0 ± 0.2	3.3 ± 0.4	2.4 ± 0.7	4.1 ± 0.2
	70	44.4 ± 1.2	53.9 ± 0.4	36.4 ± 2.9	23.3 ± 1.7	76.5 ± 5.9	18.5 ± 1.2	56.1 ± 2.4	41.5 ± 2.9	40.1 ± 1.1	57.0 ± 4.3	50.3 ± 1.4	46.6 ± 2.4	41.6 ± 1.0	64.0 ± 1.5	68.0 ± 1.5	42.6 ± 2.6	51.9 ± 2.0	48.4 ± 0.3	66.7 ± 2.7	47.2 ± 2.3	75.2 ± 1.6
**Subtotal**		**92.0 ± 6.2**	**89.0 ± 0.4**	**51.1 ± 4.8**	**49.6 ± 3.5**	49.6 ± 3.5	**63.2 ± 2.2**	**108.2 ± 2.9**	**69.1 ± 4.0**	**109.6 ± 1.2**	**72.9 ± 2.6**	**71.3 ± 4.2**	**68.5 ± 6.9**	**76.1 ± 3.3**	**91.6 ± 2.8**	**81.3 ± 1.8**	**83.8 ± 4.3**	**86.6 ± 6.0**	**65.5 ± 8.7**	**123.5 ± 9.3**	**71.4 ± 2.4**	**99.3 ± 2.3**
***Tectorigenin derivatives (3)***																						
	81^a^	0.7 ± 0.1	1.4 ± 0.5	0.6 ± 0.1	0.7 ± 0.1	ND	ND	ND	0.4 ± 0.0	ND	ND	ND	0.4 ± 0.0	0.4 ± 0.0	ND	ND	ND	ND	ND	ND	0.9 ± 0.0	ND
	56^a^	ND	ND	ND	2.6 ± 0.1	ND	ND	ND	ND	ND	ND	ND	ND	ND	ND	0.7 ± 0.1	1.0 ± 0.2	ND	ND	2.6 ± 0.1	ND	ND
	71^a^	10.2 ± 0.1	2.2 ± 0.1	5.4 ± 0.6	8.7 ± 0.5	3.4 ± 0.3	0.5 ± 0.1	2.3 ± 0.5	1.8 ± 0.2	ND	6.4 ± 0.6	2.3 ± 0.1	5.4 ± 0.5	2.9 ± 0.3	2.5 ± 0.2	8.2 ± 0.2	4.6 ± 0.6	4.3 ± 0.2	5.6 ± 0.4	11.2 ± 0.5	5.1 ± 0.1	2.4 ± 0.2
**Subtotal**		**10.9 ± 0.1**	**3.6 ± 0.5**	**6.0 ± 0.7**	**12.0 ± 0.6**	**3.4 ± 0.3**	**0.5 ± 0.1**	**2.3 ± 0.5**	**2.2 ± 0.4**	**ND**	**6.4 ± 0.6**	**2.3 ± 0.1**	**5.8 ± 0.6**	**3.3 ± 0.3**	**2.5 ± 0.2**	**8.9 ± 0.3**	**5.6 ± 0.7**	**4.3 ± 0.2**	**5.6 ± 0.4**	**13.9 ± 0.6**	**6.0 ± 0.1**	**2.4 ± 0.2**
***Afromosin derivatives (3)***																						
	83	ND	8.4 ± 0.1	ND	ND	ND	ND	1.0 ± 0.1	ND	ND	ND	ND	1.8 ± 0.1	ND	0.5 ± 0.1	ND	0.3 ± 0.1	ND	ND	2.4 ± 0.1	ND	ND
	72	ND	ND	ND	ND	ND	0.9 ± 0.3	0.2 ± 0.1	ND	1.3 ± 0.2	ND	ND	ND	ND	ND	ND	ND	ND	ND	ND	ND	ND
	78^a^	0.7 ± 0.2	1.9 ± 0.2	1.4 ± 0.4	ND	ND	1.1 ± 0.2	2.5 ± 0.2	1.0 ± 0.2	1.6 ± 0.4	0.2 ± 0.1	2.5 ± 0.3	1.7 ± 0.1	1.2 ± 0.3	1.7 ± 0.2	1.5 ± 0.3	0.8 ± 0.2	1.8 ± 0.3	0.5 ± 0.1	4.2 ± 0.3	2.5 ± 0.3	2.2 ± 0.4
**Subtotal**		**0.7 ± 0.2**	**10.4 ± 0.3**	**1.4 ± 0.4**	**ND**	**ND**	**2.1 ± 0.3**	**3.8 ± 0.4**	**1.0 ± 0.2**	**2.9 ± 0.5**	**0.2 ± 0.1**	**2.5 ± 0.3**	**3.5 ± 0.1**	**1.2 ± 0.3**	**2.2 ± 0.2**	**1.5 ± 0.3**	**1.1 ± 0.2**	**1.8 ± 0.3**	**0.5 ± 0.1**	**6.6 ± 0.2**	**2.5 ± 0.5**	**2.2 ± 0.4**
***Formononetin derivatives (2)***																						
	82	0.4 ± 0.0	1.9 ± 0.2	ND	ND	ND	ND	0.6 ± 0.1	ND	ND	ND	ND	1.2 ± 0.1	ND	1.0 ± 0.1	ND	2.4 ± 0.0	ND	ND	1.1 ± 0.1	ND	ND
	77	1.3 ± 0.2	5.3 ± 0.1	ND	ND	2.1 ± 0.5	1.0 ± 0.4	0.9 ± 0.1	1.4 ± 0.2	1.4 ± 0.0	2.4 ± 0.7	0.5 ± 0.1	1.5 ± 0.5	0.4 ± 0.2	3.0 ± 0.4	1.3 ± 0.2	2.7 ± 0.1	1.3 ± 0.3	3.5 ± 0.1	1.6 ± 0.3	0.6 ± 0.2	2.1 ± 0.2
**Subtotal**		**1.6 ± 0.2**	**7.2 ± 0.2**	**ND**	**ND**	**2.1 ± 0.5**	**1.0 ± 0.4**	**1.4 ± 0.1**	**1.4 ± 0.2**	**1.4 ± 0.0**	**2.4 ± 0.7**	**0.5 ± 0.1**	**2.7 ± 0.5**	**0.4 ± 0.2**	**4.0 ± 0.3**	**1.3 ± 0.2**	**5.0 ± 0.2**	**1.3 ± 0.3**	**3.5 ± 0.1**	**2.7 ± 0.2**	**0.6 ± 0.2**	**2.1 ± 0.2**
**Isoflavones**		**112.4 ± 3.9**	**130.6 ± 0.8**	**61.2 ± 8.0**	**63.7 ± 3.5**	**135.2 ± 5.1**	**70.6 ± 1.8**	**121.4 ± 2.7**	**77.3 ± 4.2**	**121.3 ± 1.9**	**90.7 ± 1.1**	**92.5 ± 0.5**	**87.8 ± 5.5**	**85.6 ± 1.9**	**104.5 ± 1.9**	**95.9 ± 1.5**	**105.1 ± 8.1**	**98.4 ± 7.9**	**15.1 ± 0.5**	**154.0 ± 5.6**	**84.1 ± 4.1**	**109.5 ± 1.3**
**Total**		**532.8 ± 18.6**	**542.8 ± 16.3**	**342.5 ± 17.2**	**427.0 ± 15.7**	**951.1 ± 33.4**	**849.1 ± 43.4**	**747.0 ± 18.2**	**842.5 ± 56.1**	**992.7 ± 35.2**	**671.7 ± 30.6**	**874.2 ± 31.0**	**742.5 ± 9.3**	**578.2 ± 11.7**	**613.6 ± 30.0**	**640.9 ± 42.3**	**576.3 ± 26.0**	**882.7 ± 29.4**	**408.4 ± 1.9**	**776.1 ± 34.1**	**769.9 ± 25.3**	**619.9 ± 12.6**

Each value calculated as mean values ± SD (*n* = 3) using internal standards (6-methoxyluteolin and 6-methoxyflavone for flavonol-flavone and isoflavone, respectively). SL, soybean leaf; ND, not detected.

a, new flavonoid in soybean leaves; b, newly named. Compound names are presented according to peak numbers in Table 1.

Significant values are in [bold].

**rham**^**1**^**(glu**^**(1)**^**)-glu**^**2(6)**^ (peaks **5**, **17** and **15**) varied from that of other Korean cultivars based on differences in the collected origins. The **SL**18 (GNU-2007-14502, Korean landrace; **TF**s 378.8) also provided a specific profile of higher **mono**-glycosides (48.6%; mainly 3-*O*-glucose and 3-*O*-galactose) as presented in Fig. 2C and Table 2.

Among **I**-rich **SL**s 1, 4, 11, 13, 16, 17 and 19, exceptionally, **SL**11 (Geomjeongkong-5, Korean landrace; **IG**s 31.4%, **di**- 92.7%) with higher **TF**s (773.5) contained predominantly **di**-glycosides of **glu**^**1**^**-gal**^**2**^ (peaks **8**, **26** and **31**), **glu**^**1**^**-glu**^**2**^ (**sop**, peaks **11**, **28** and **32**) and **rham**^**1**^**-gal**^**2**^ (peaks **24**, **37** and **47**) including I 3-*O*-(2″-*O*-glu)gal (peak **31**, 103.8), I 3-*O*-(2″-*O*-glu)glu (peak **32**, 65.3) and I 3-*O*-(2″-*O*-rham)gal (peak **47**, 54.6), which were reported as low level in previous studies^14–17,24^. Besides, the new **tri**-**IG**s (178.7; **soyanin**s **I**–**V**, peaks **22**, **23**, **33**, **34** and **42**) closely related to **di**-**IG**s in above **SL**11 included I 3-*O*-(2″-*O*-glu-6″-*O*-rham)glu (70.5; **soyanin II**, peaks **23**), I 3-*O*-(2″,6″-di-*O*-rham)gal (19.1; **soyanin III**, peaks **33**) and I 3-*O*-(4″,6″-di-*O*-rham)gal (45.9; **soyanin IV**, peaks **34**) as major **IG**s in **SL**19 (Junyeorikong, Korean landrace; **TF**s 611.8, **IG**s 39.8%, **tri**- 74.8%). It is considered that these **IG**s results play an important role on prediction of flavonol biosynthesis as well as determination of their precise structures (based on NMR) and contents from the **SL**s in further research.

**Figure 3:**
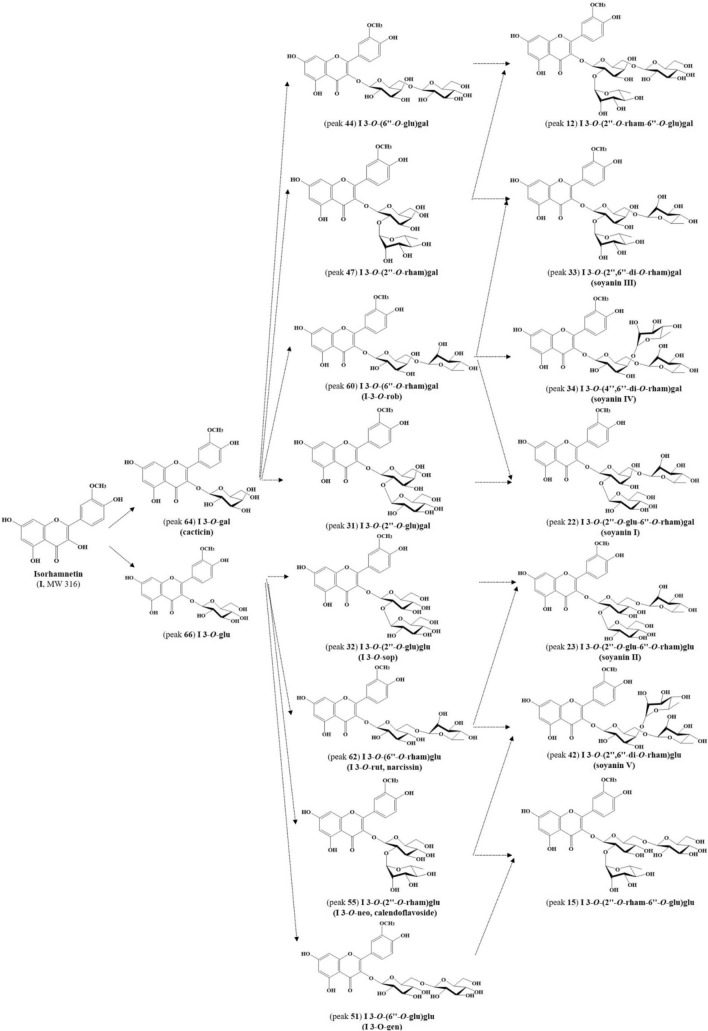
Proposed biosynthetic pathway of 17 isorhamnetin (**I**) glycosides (**mono**-, peaks **64** and **66**; **di**-, peaks **31**, **32**, **44**, **47**, **51**, **55**, **60** and **62**; **tri**-, peaks **12**, **15**, **22**, **23**, **33**, **34** and **42**) identified from young leaves of soybean cultivars (**SL**s 1, 4, 16 and 19). Compound names are presented according to peak numbers in Table 1. gal, galactose; glu, glucose; rham, rhamnose; gen, gentiobiose; rob, robinobiose; rut, rutinose; neo, neohesperidose; sop, sophorose.

In Fig. 3 and Supplementary Fig. S3, the flavonol biosynthetic pathways could be predicted through the present 52 glycosides (6 **mono**-, 24 **di**- and 22 **tri**-) according to aglycones (**K**, **Q**, and **I**) found from young leaves of 21 core-collected soybean cultivars. In general, the cyanidin 3-*O*-glucoside and peonidin 3-*O*-glucoside have been reported as major anthocyanins from black soybean seeds^41–43^. The rich **QG**s and **IG**s characterized only in black coated cultivars of this study^14,16^ suggest that they are closely related to the corresponding cyanidin and peonidin based-structures. Thus, it is considered significant that the rich **SL**s flavonol profiles can contribute to enhanced overproduction of seed anthocyanins through regulation of specific genes at the growth stage^23,44,45^ as well as select superior varieties which are expected to have higher biological activities. Thus, the present study summarized the relationship between cultivars and individual flavonoids content according to their aglycones and glycosides, and further described that the **SL**s from yellow-coated seed mostly composed of **KG**s, whereas, the **SL**s from black-coated seed presented as **QG**s and **IG**s rich sources (Fig. 2 and Table 2). In the future, it is also necessary to perform metabolomics approach to how these **SL**s flavonols change during the leaf growth and its fermentation, and investigate the correlation between **SL**s flavonoids and their biological activities in addition to agronomic characteristics.

## Materials and methods

### Plant materials

Among 23,199 soybean germplasms provided by the Gene Bank of National Agrobiodiversity Center (NAC, Korea), 1,000 core collected accessions with superior agronomic and functional traits were chosen. Finally, a total of twenty-one soybean cultivars (varieties, landraces and breeding lines) with a specific introduction (IT) number including three Korean representative varieties (Shinpaldalkong2ho, Daewon kong and Cheongja 2) were selected considering their genetic diversity and flavonoid profiles (Table 3). The seeds of these cultivars were sown on experimental field (5 June 2019, in rows at a spacing of 15 cm) located at the center (latitude/longitude: 35$^\circ$ 4938.37 N/127$^\circ$ 0907.78 E), and cultivated under similar conditions during the country’s cropping season (June-November 2019) and their leaves (randomly taken with 5–10 cm) were harvested after 4 weeks. The **SL**s were lyophilized and finely grounded with a sample mill for their use as analytical samples. Additionally, the seed coat color matured were further grouped as yellow, black and green-black when approximately 95% of their pods reached ‘mature color46’ in a maturity index. Experimental research and field studies on plant materials of this study complies with relevant institutional, national, and international guidelines and legislation.

**Table 3:** Characteristics of core collected soybean cultivars used in the present study.

No	Code	Accession number	Name	Seed coat color	Origins	Cultivars
1	SL1	IT263155	Shinpaldalkong2ho	Yellow	Korea	Variety
2	SL2	IT212859	Daewon kong	Yellow	Korea	Variety
3	SL3	IT213192	Cheongja 2	Black	Korea	Variety
4	SL4	IT021665	PI 90763	Black	China	Landrace
5	SL5	IT024099	YJ208-1	Yellow	Korea	Landrace
6	SL6	IT104690	Kongnamulkong	Yellow	Korea	Landrace
7	SL7	IT113218	Kongnamulkong	Yellow	Korea	Landrace
8	SL8	IT154724	KAS651-21	Green-Black	Korea	Landrace
9	SL9	IT155963	Nongrim 51	Yellow	Japan	Breeding line
10	SL10	IT161904	PI 84578	Black	Korea	Landrace
11	SL11	IT177573	Geomjeongkong-5	Black	Korea	Landrace
12	SL12	IT186183	Kongnamulkong	Black	Korea	Landrace
13	SL13	IT194560	Geomjeongkong	Black	Korea	Landrace
14	SL14	IT231360	Kongnamulkong	Yellow	Korea	Landrace
15	SL15	IT239896	Jwineorikong	Black	Korea	Landrace
16	SL16	IT252768	326	Black	Korea	Landrace
17	SL17	IT269617	CS 02028	Black	Korea	Landrace
18	SL18	IT274515	GNU-2007-14502	Black	Korea	Landrace
19	SL19	IT308619	Junyeorikong	Black	Korea	Landrace
20	SL20	K137773	Heukseong	Black	Korea	Breeding line
21	SL21	IT156272	Himeyudaga	Yellow	Japan	Breeding line

### Chemical reagents

Reference standards of apigenin, daidzein, daidzin, formononetin, genistein and genistin were obtained from Sigma-Aldrich Co. (St Louis, MO, USA); astragalin, calendoflavoside, cosmosiin, cynaroside, glycitin, hyperoside, isoquercitrin, isorhamnetin 3-*O*-glucoside, luteolin, narcissin, nicotiflorin and rutin as well as 6-methoxyluteolin and 6-methoxyflavone as internal standards were purchased from Extrasynthese (Genay, France); 6-*O*-malonyldaidzin and 6-*O*-malonylgenistin from Synthose Inc. (Ontario, Canada); kaempferol 3-*O*-gentiobioside, quercetin 3-*O*-gentiobioside and quercetin 3-*O*-sophoroside from PhytoLab GmbH & Co. (Vestenbergsgreuth, Germany); Cacticin and trifolin from MedChemExpress (Monmouth Junction, USA). LC–MS grade methanol, acetonitrile and water were supplied from Thermo Fisher Scientific Inc. (Waltham, MA, USA). Besides, formic acid (Junsei Chemical, Tokyo, Japan) was used as eluent additive in extraction and chromatographic separation of flavonoid derivatives.

### Extraction of flavonoid derivatives

The powdered samples (1.0 g) were extracted with mixed solvents (10 mL, methanol:water:formic acid, 50:45:5, v/v/v) for 30 min at 200 rpm using an orbital shaker, and then centrifuged at 2016 × *g* and 4℃ for 15 min (LABOGENE 1580R, LABOGENE, Korea). Each supernatant was filtered through a PVDF syringe filter (0.2 µm, Thermo Fisher Scientific Inc., Waltham, MA, USA). The filtrates (0.5 mL) and internal standards (**IS**, 0.5 mL) were further diluted with distilled water to 7 mL (final volume), respectively. The **IS** solution (50 µg/mL) was composed of 6-methoxyluteolin (for flavonol and flavone) and 6-methoxyflavone (for isoflavone) to quantify the identified flavonoid derivatives. In order to obtain the crude flavonoids, a solid phase extraction (SPE) method was performed with a Hypersep C_18_ SPE cartridge (Thermo Fisher Scientific Inc., Waltham, MA, USA). Briefly, the initial cartridge activation was proceeded through washing with methanol (3 mL), followed by conditioning with distilled water (5 mL). Then, the previously diluted solutions of extracts and **IS** were sequentially loaded on activated cartridge and washed with distilled water (5 mL) to remove impurities. Finally, a loaded sample was eluted from the cartridge by 5 mL of methanol (with 1% formic acid). The semi-purified flavonoid eluate was concentrated using N_2_ gas and re-dissolved in 0.5 mL of extraction solvent prior to UPLC-DAD-QToF/MS analysis. All analyzes were carried out in triplicates.

### UPLC-DAD-QTOF/MS analysis

An analytical system of UPLC-DAD (ACQUITY UPLC™ system, Waters Co., Miliford, MA, USA) and QToF/MS (Xevo G2-S QToF, Waters MS Technologies, Manchester, UK) equipped with CORTECS T3 C18 column (2.1 × 150 mm, 1.6 μm, Waters Co.) were operated to identify and quantify numerous flavonoid derivatives from young leaves of soybean cultivars. According to our previous reports^46,47^, chromatographic conditions used were: flow rate (0.3 mL/min), column oven temperature (30 °C), sample injection volume (1 μL). UV spectra was multi-scanned in the region of 210–400 nm (representative wavelengths; 254 nm for isoflavones, 350 nm for flavonols and flavones). The gradient profile was set followed as: initial 5% B; 20 min, 25% B; 25 min, 50% B; 30–32 min, 90%, 35–40 min, 5% B with 0.5% formic acid in water for eluent A and 0.5% formic acid in acetonitrile for eluent B used as mobile phases. Mass spectra were simultaneously measured with the range of *m/z* 100–1,200 in positive ionized mode using an electrospray ionization (+ ESI) probe, and their parameters used were: capillary voltage 3.5 kV, sampling cone voltage 40 V, source temperature 120 °C, desolvation temperature 500 °C, desolvation N_2_ gas flow 1020 L/h. To maintain mass accuracy, 0.5 mM sodium formate solution was used externally for the mass calibration, and also, leucine-enkephalin (2 ng/μL) was monitored internally as a reference standard (*m/z* 556.2766) in real time and introduced using the LockSpray interface at 10 μL/min.

### Identification and quantification of flavonoid derivatives

The LC–MS library (from ‘RDA DB 1.0—Flavonoids’ completed in 2016)^47^ was constructed to carry out more clear and efficient identification of flavonoid derivatives from the **SL**s based on literature’s analytical data with structural evidences elucidated by NMR and MS spectroscopies, and composed of 53 flavonoids information including positive and negative ion fragmentations (Supplementary Table S1). The purposed flavonoids were tentatively determined by considering the positive fragmentation (reported and proposed), UV spectra (λ_max_, data not shown) and elution order presented in the constructed library^48^ (Table 2). Additionally, some derivatives of them were further confirmed through comparison with 25 types of reference standards provided in Table 2. However, since it is not complete to obtain all available standards consistent with the identified derivatives, the quantification for each peak (based on UV detection) was calculated as 1:1 without considering the relative response factor for **IS**, and expressed as mean ± standard deviation of their triplicated results (Table 3). Especially, in order to select and maintain a stable **IS**, it was verified that the pre-inserted **IS** did not overlap with sample peaks, and its recovery was repeatedly validated to correct errors that may occur during the SPE process.

## Conclusions

In this study, a total of 83 flavonoid derivatives were comprehensively identified and quantified from young leaves of 21 core-collected soybean cultivars based on high-resolution UPLC-DAD-QToF/MS analysis with constructed LC–MS library previously reported. Among flavonoid derivatives, the abundant flavonols contained mainly as di- and tri-glycosidic forms from the **SL**s were distributed in the order of **K**, **Q**, and **I** according to their aglycone types, and had different predominant aglycones under affected by the cultivar’s characteristics. The **SL**s from yellow-coated seed mostly composed of **KG**s, whereas, the **SL**s from black-coated seed presented as **QG**s and **IG**s rich sources. From identified 83 flavonoid derivatives, the flavonol biosynthetic pathways were proposed according to the aglycones (**K**, **Q**, and **I**), so it is considered that the pathways can play a key role in determining their structures precisely and predicting flavonol biosynthesis. Thus, the **SL**s flavonoid profiles can contribute to breed superior varieties with excellent biological activities and perform metabolomics approach to investigate the changes of these flavonols of **SL**s during the leaf growth and fermentation in further study.

## Supplementary Information

Supplementary Information 1.

Supplementary Information 2.

Supplementary Information 3.

Supplementary Information 4.

## Data Availability

The flavonoids data presented in this study was cited from a ‘Flavonoid Database Search’ (http://koreanfood.rda.go.kr/eng/fctFoodSrchEng/main) belong to Korean Food Composition Database provided in National Institute of Agricultural Sciences, Rural Development Administration (RDA).
