# Supplementary material for: Comprehensive characterization of flavonoid derivatives in young leaves of core-collected soybean (Glycine max L.) cultivars based on high-resolution mass spectrometry
Source: Sci Rep. 2022 Aug 29;12:14678. doi: 10.1038/s41598-022-18226-4 (PMC9424525; doi:10.1038/s41598-022-18226-4)
Supplement: Supplementary file 1 — Supplementary Information 1. [file 41598_2022_18226_MOESM1_ESM.pdf]

Supplementary Figure S1. UPLC-DAD chromatograms of flavonol / flavone (wavelength at 350 nm) and isoflavone (wavelength at 254 nm) derivatives from young leaves of 21 soybean cultivars. Code of each cultivars (SLs 1-21) correspond with Table 1.

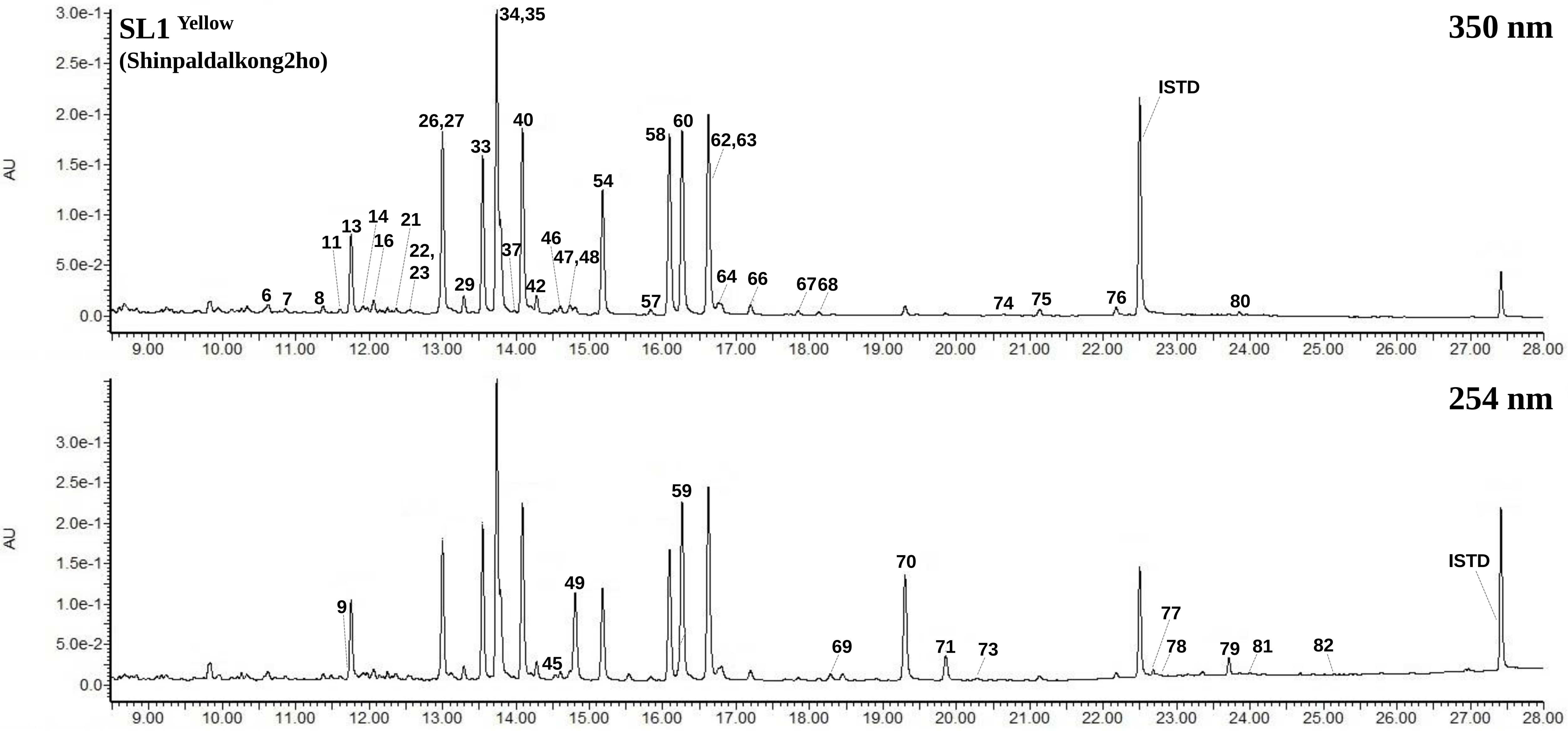
SL1 Yellow
(Shinpaldalkong2ho)
350 nm
254 nm
AU
ISTD
34,35
26,27
33
40
58
60
62,63
54
13
14
21
11
16
22,
23
37
46
47,48
29
42
6
7
8
57
64
66
67
68
74
75
76
80
59
70
49
9
45
69
71
73
77
78
79
81
82
3.0e-1
2.5e-1
2.0e-1
1.5e-1
1.0e-1
5.0e-2
0.0
9.00
10.00
11.00
12.00
13.00
14.00
15.00
16.00
17.00
18.00
19.00
20.00
21.00
22.00
23.00
24.00
25.00
26.00
27.00
28.00

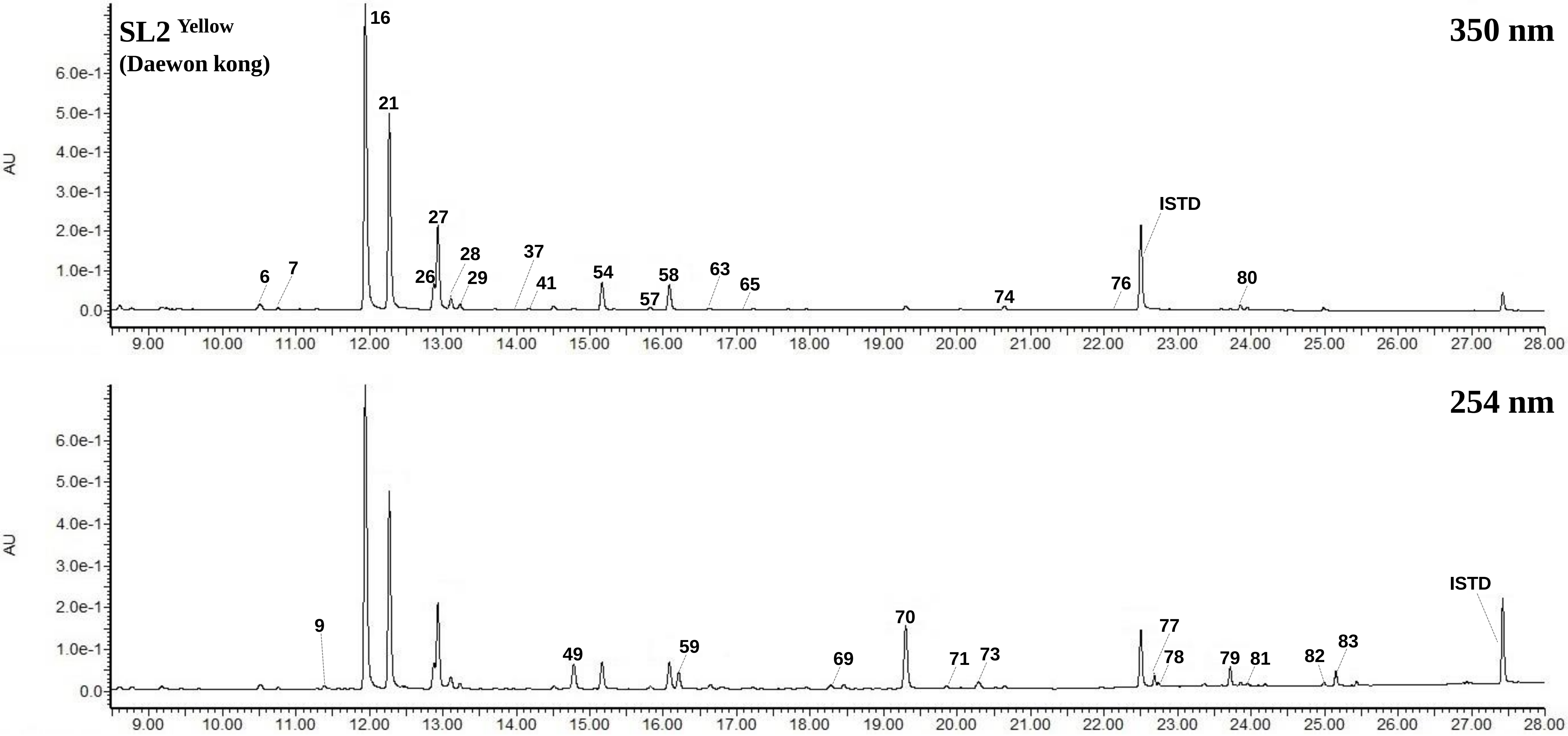
SL2 Yellow
(Daewon kong)
350 nm
254 nm
AU
ISTD
16
21
27
28
29
26
37
41
54
57
58
63
65
74
76
80
6
7
9
49
59
69
70
71
73
77
78
79
81
82
83

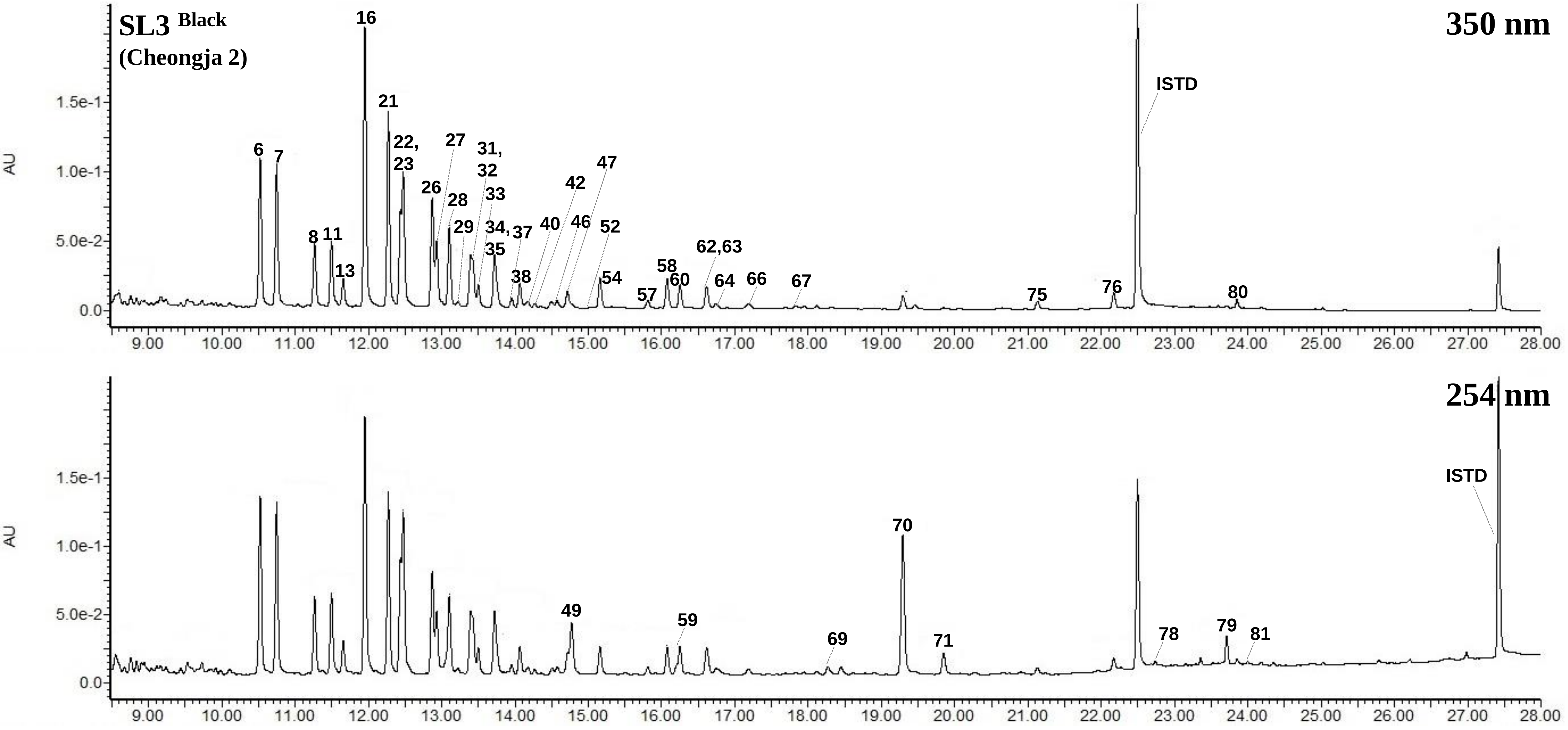
SL3 Black
(Cheongja 2)
350 nm
254 nm
AU
ISTD
6
7
8
11
13
16
21
22,
23
26
27
28
29
31,
32
33
34,
35
37
38
40
42
46
47
49
52
54
57
58
59
60
62,63
64
66
67
69
70
71
75
76
78
79
80
81
1.5e-1
1.0e-1
5.0e-2
0.0
9.00
10.00
11.00
12.00
13.00
14.00
15.00
16.00
17.00
18.00
19.00
20.00
21.00
22.00
23.00
24.00
25.00
26.00
27.00
28.00

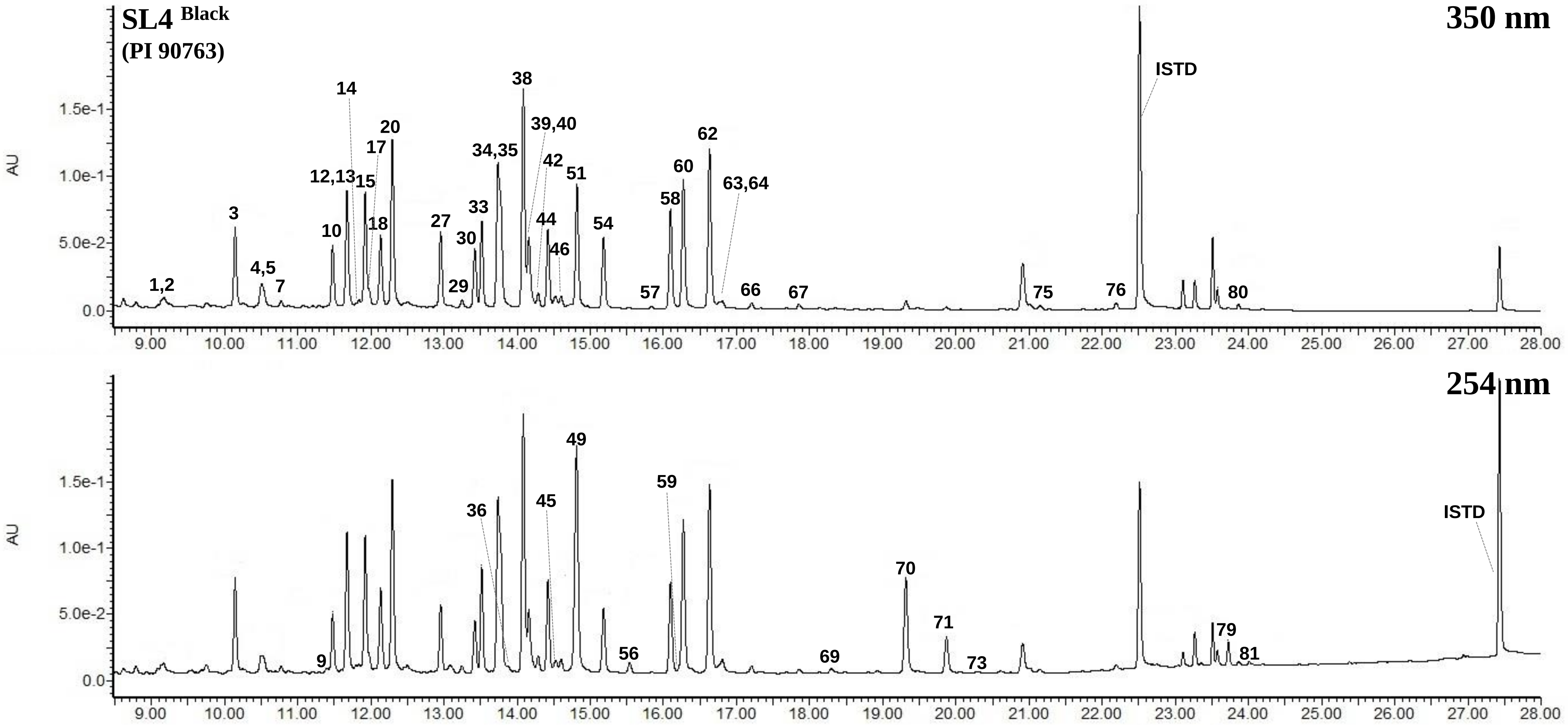

SL4 Black
(PI 90763)
350 nm
254 nm
AU
ISTD
1,2
3
4,5
7
9
10
12,13
14
15
17
18
20
27
29
30
33
34,35
36
38
39,40
42
44
45
46
49
51
54
56
57
58
59
60
62
63,64
66
67
69
70
71
73
75
76
79
80
81

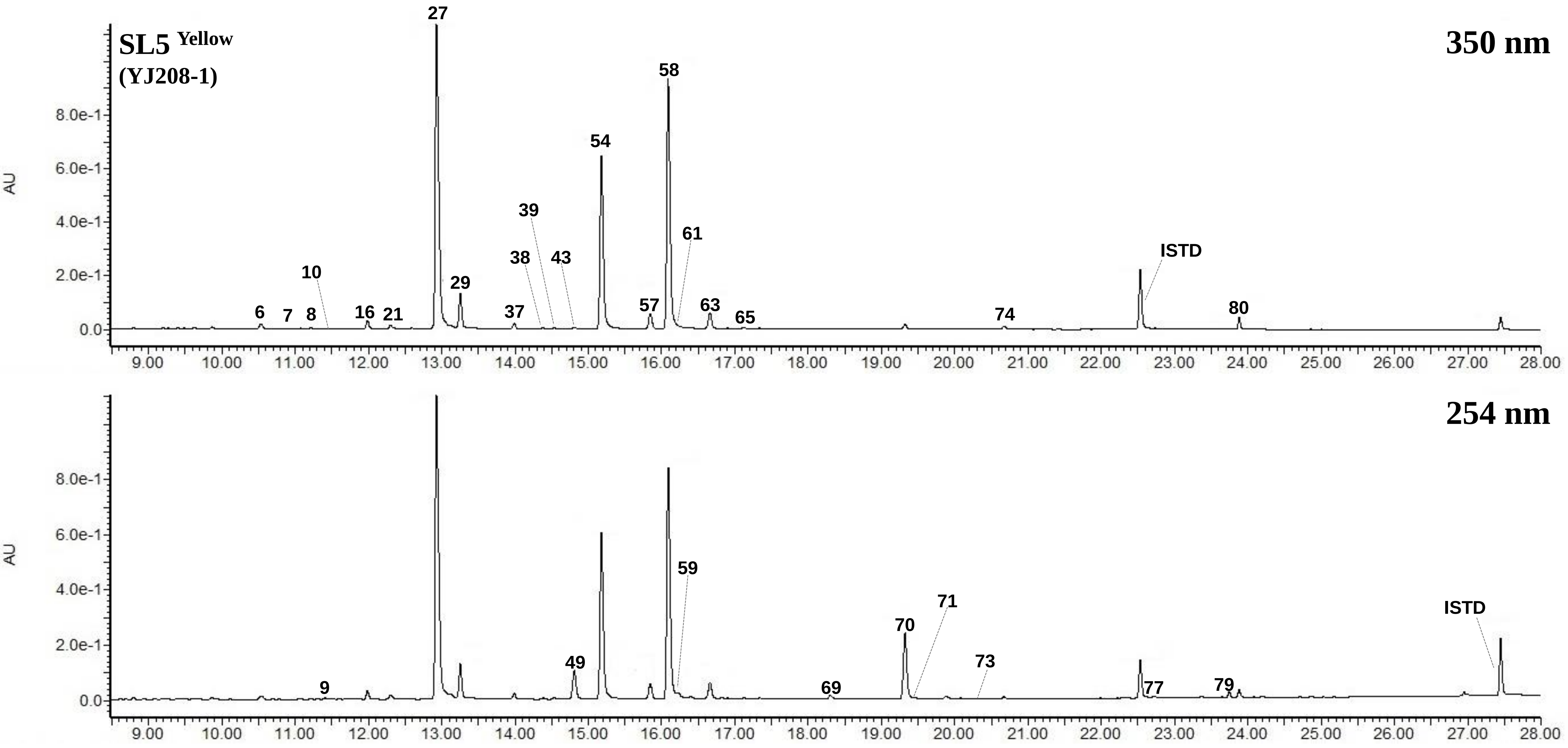

SL5 Yellow
(YJ208-1)
350 nm
254 nm
AU
8.0e-1
6.0e-1
4.0e-1
2.0e-1
0.0
9.00
10.00
11.00
12.00
13.00
14.00
15.00
16.00
17.00
18.00
19.00
20.00
21.00
22.00
23.00
24.00
25.00
26.00
27.00
28.00
27
58
54
39
61
38
43
ISTD
10
29
6
7
8
16
21
37
57
63
65
74
80
59
71
70
73
49
9
69
77
79

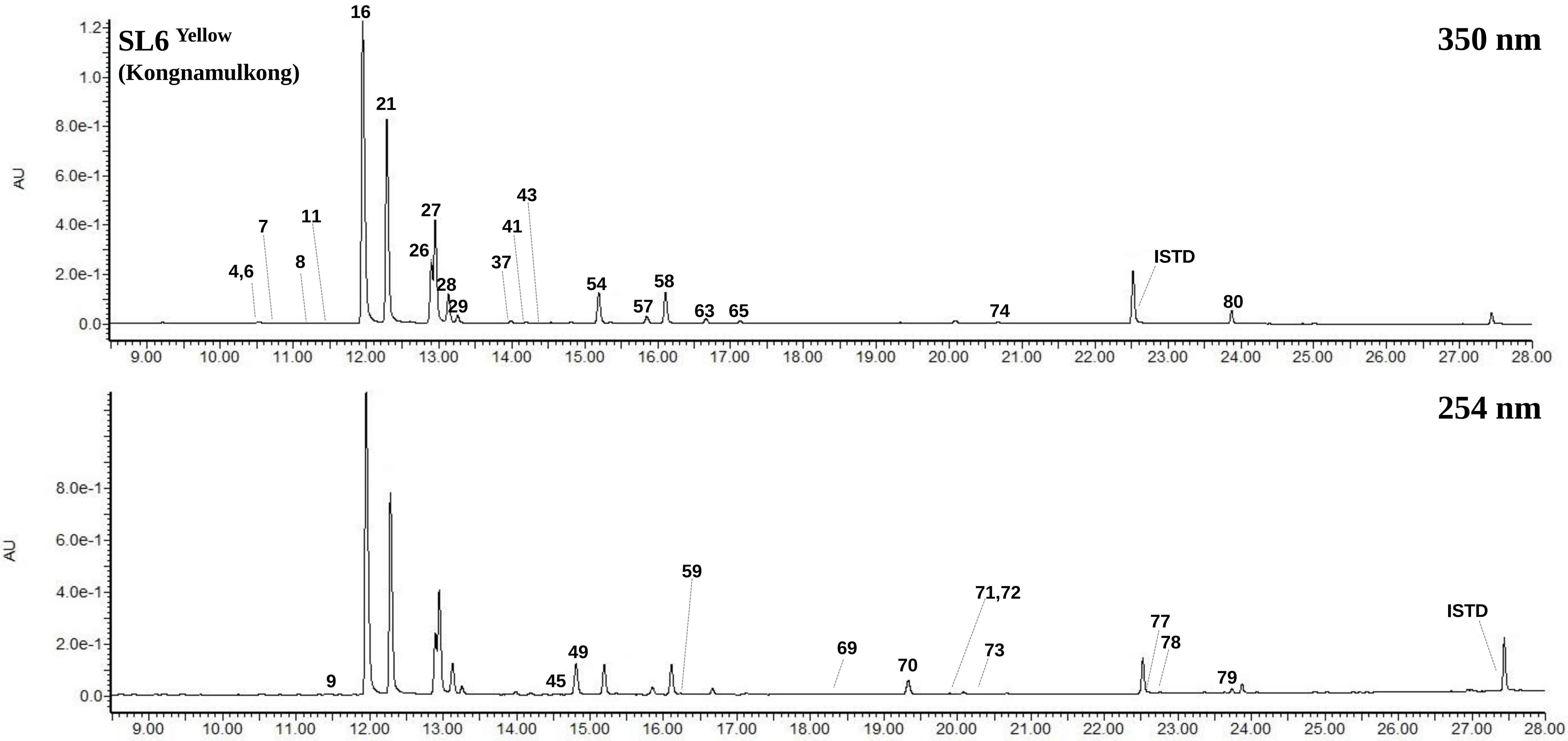
SL6 Yellow
(Kongnamulkong)
350 nm
254 nm
AU
16
21
27
26
28
29
43
41
37
11
8
7
4,6
54
57
58
63
65
74
ISTD
80
9
45
49
59
69
70
71,72
73
77
78
79
ISTD

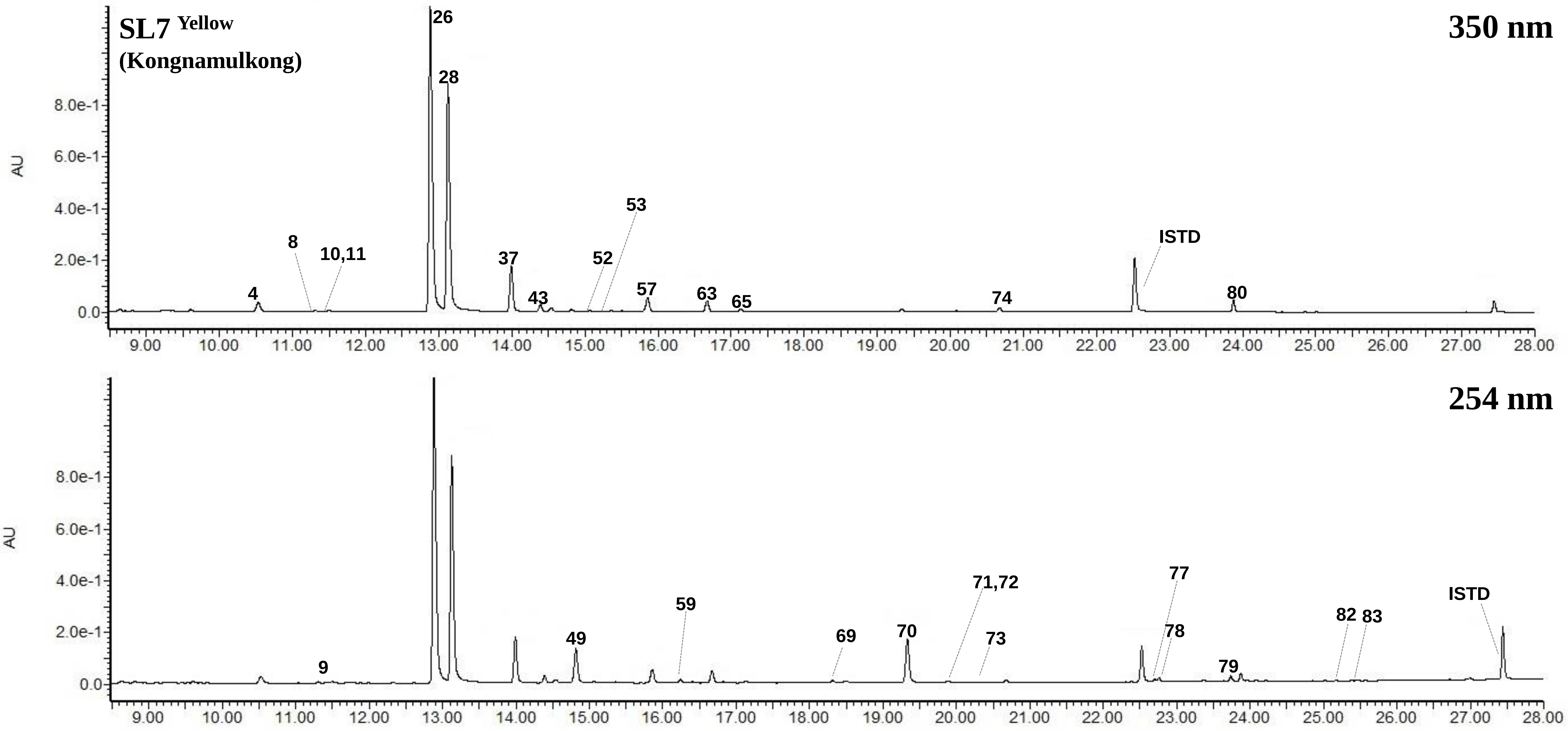

SL7 Yellow
(Kongnamulkong)
350 nm
254 nm
AU
8.0e-1
6.0e-1
4.0e-1
2.0e-1
0.0
26
28
4
8
10,11
37
43
52
53
57
63
65
74
ISTD
80
9
49
59
69
70
71,72
73
77
78
79
82
83
9.00
10.00
11.00
12.00
13.00
14.00
15.00
16.00
17.00
18.00
19.00
20.00
21.00
22.00
23.00
24.00
25.00
26.00
27.00
28.00

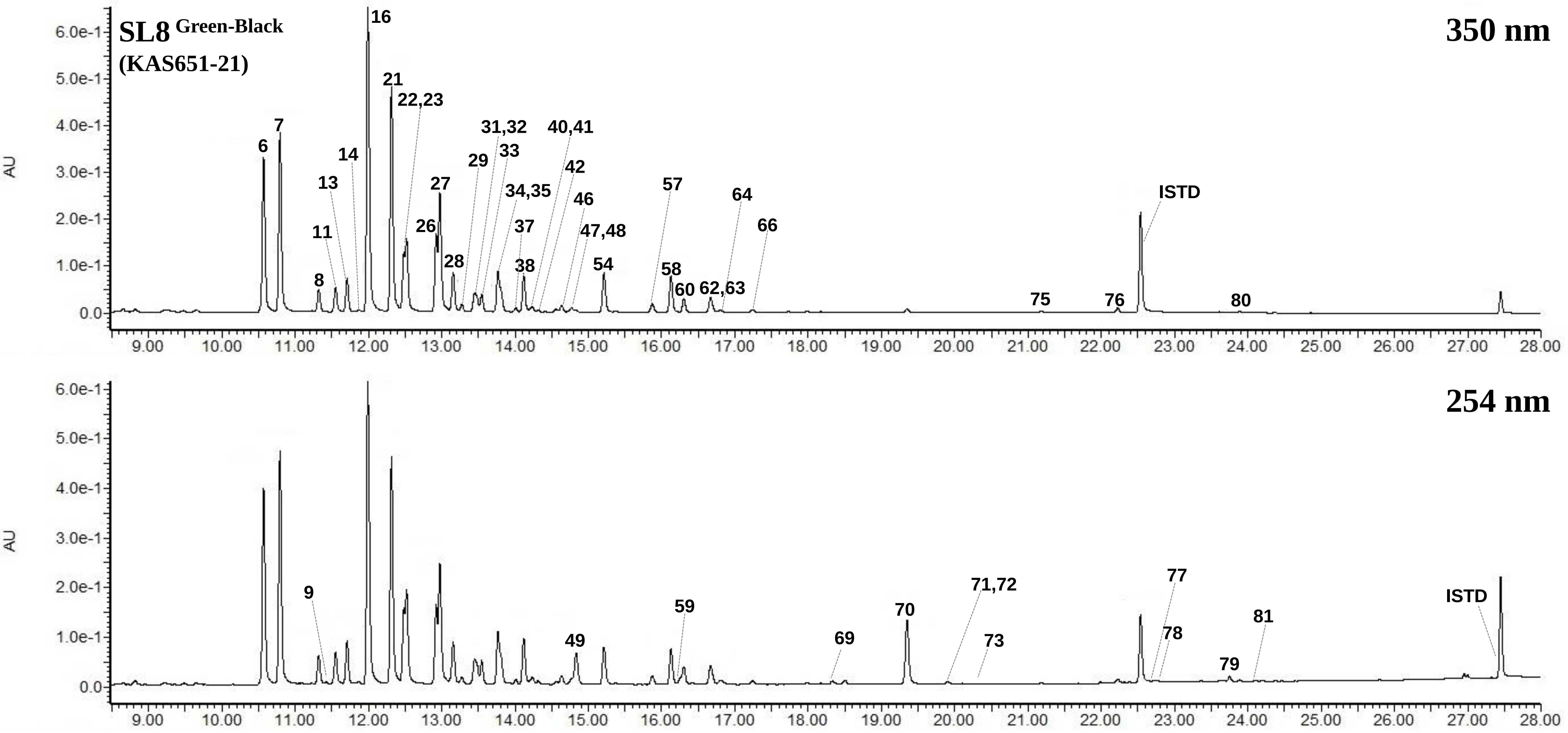
SL8 Green-Black
(KAS651-21)
350 nm
254 nm
AU
6.0e-1
5.0e-1
4.0e-1
3.0e-1
2.0e-1
1.0e-1
0.0
9.00
10.00
11.00
12.00
13.00
14.00
15.00
16.00
17.00
18.00
19.00
20.00
21.00
22.00
23.00
24.00
25.00
26.00
27.00
28.00
6
7
8
11
13
14
16
21
22,23
26
27
28
29
31,32
33
34,35
37
38
40,41
42
46
47,48
54
57
58
60
62,63
64
66
75
76
ISTD
80
9
49
59
69
70
71,72
73
77
78
79
81
ISTD

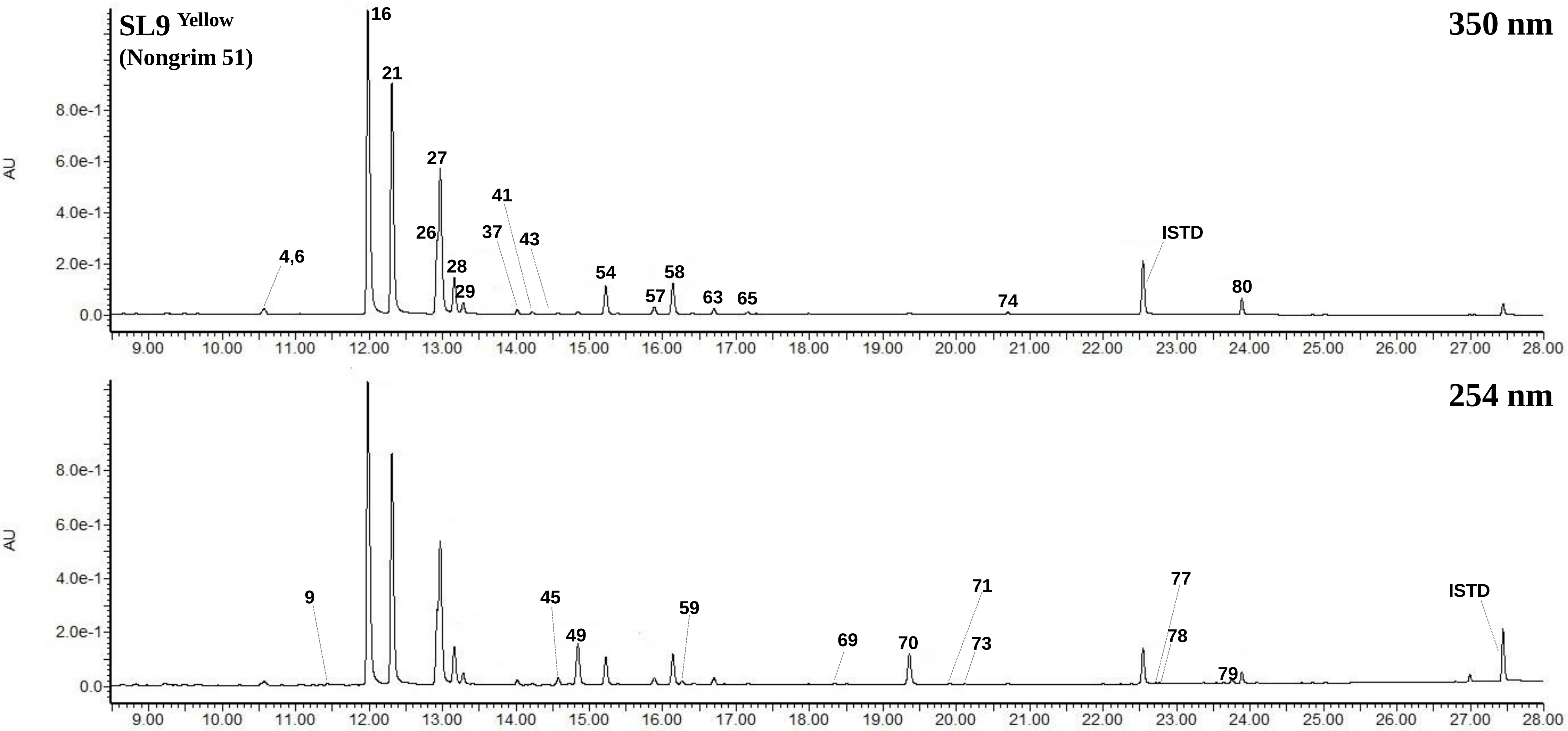
SL9 Yellow
(Nongrim 51)
350 nm
254 nm
AU
8.0e-1
6.0e-1
4.0e-1
2.0e-1
0.0
9.00
10.00
11.00
12.00
13.00
14.00
15.00
16.00
17.00
18.00
19.00
20.00
21.00
22.00
23.00
24.00
25.00
26.00
27.00
28.00
16
21
27
41
26
37
43
4,6
28
29
54
57
58
63
65
74
ISTD
80
9
45
49
59
69
70
71
73
77
78
79
ISTD

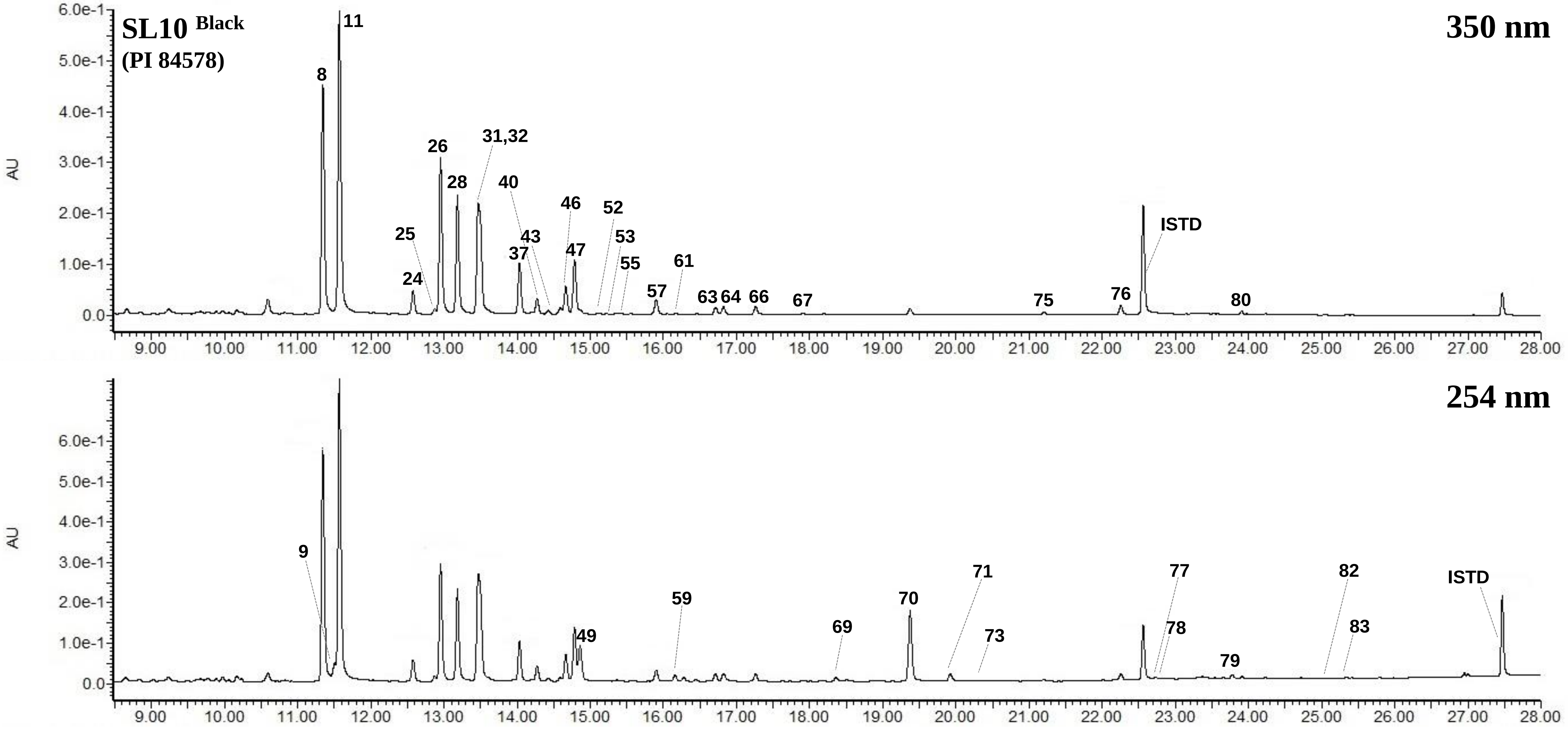
SL10 Black
(PI 84578)
350 nm
254 nm
AU
ISTD
8
9
11
24
25
26
28
31,32
37
40
43
46
47
49
52
53
55
57
59
61
63
64
66
67
69
70
71
73
75
76
77
78
79
80
82
83
6.0e-1
5.0e-1
4.0e-1
3.0e-1
2.0e-1
1.0e-1
0.0
9.00
10.00
11.00
12.00
13.00
14.00
15.00
16.00
17.00
18.00
19.00
20.00
21.00
22.00
23.00
24.00
25.00
26.00
27.00
28.00

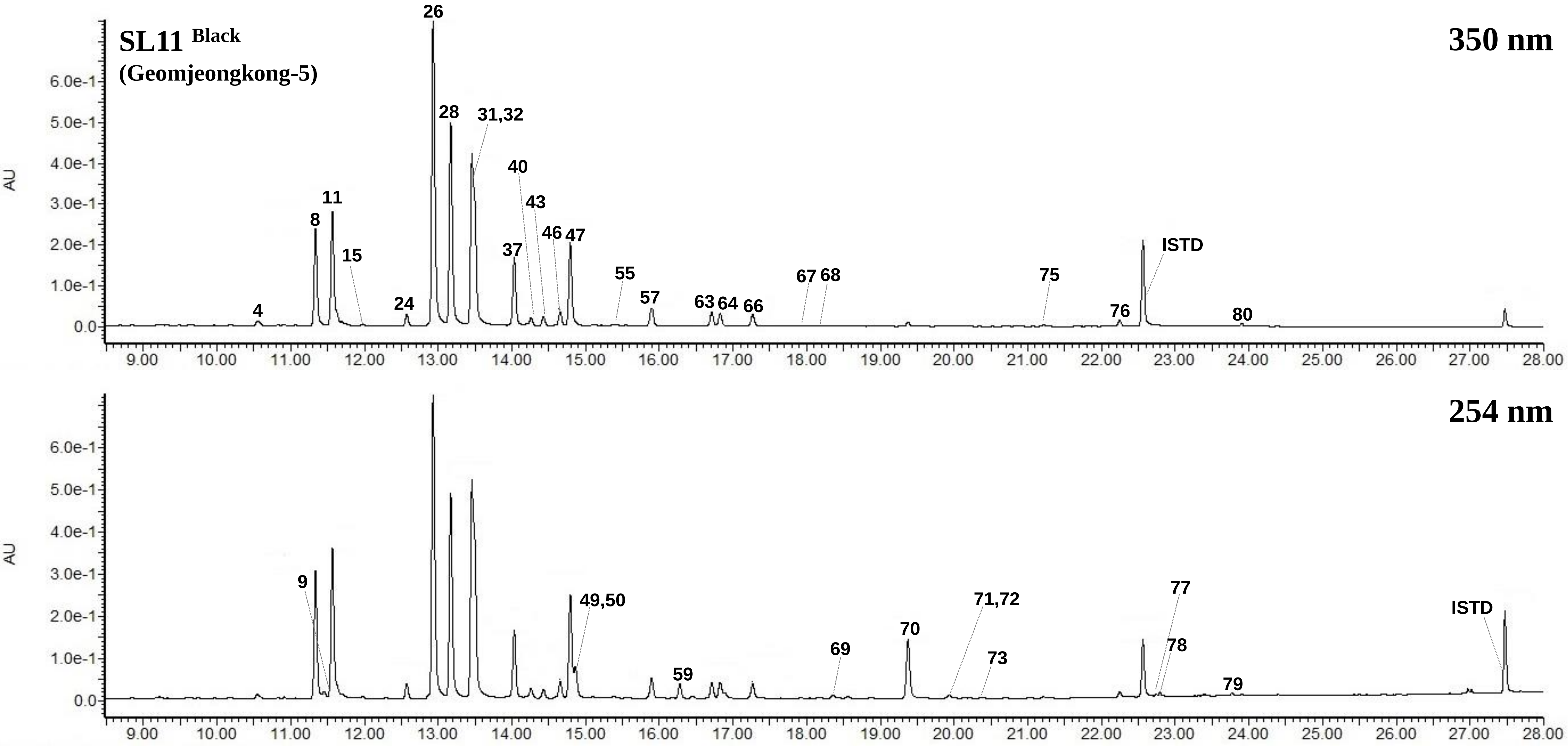
SL11 Black
(Geomjeongkong-5)
350 nm
254 nm
AU
0.0
1.0e-1
2.0e-1
3.0e-1
4.0e-1
5.0e-1
6.0e-1
9.00
10.00
11.00
12.00
13.00
14.00
15.00
16.00
17.00
18.00
19.00
20.00
21.00
22.00
23.00
24.00
25.00
26.00
27.00
28.00
4
8
11
15
24
26
28
31,32
37
40
43
46
47
55
57
63
64
66
67
68
75
76
ISTD
80
9
49,50
59
69
70
71,72
73
77
78
79
ISTD

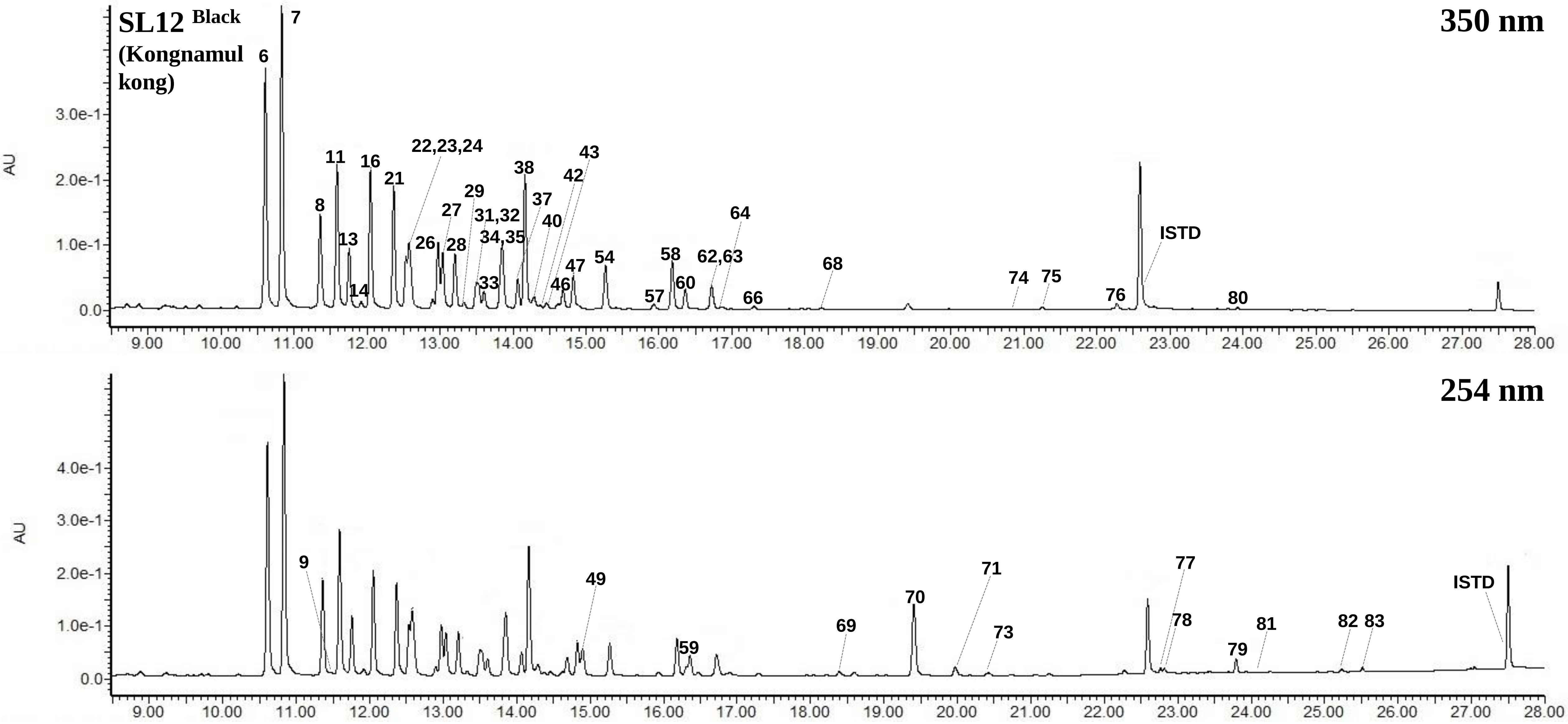
SL12 Black
(Kongnamul kong)
350 nm
254 nm
AU
6
7
8
9
11
13
14
16
21
22,23,24
26
27
28
29
31,32
33
34,35
37
38
40
42
43
46
47
49
54
57
58
59
60
62,63
64
66
68
69
70
71
73
74
75
76
77
78
79
80
81
82
83
ISTD
ISTD

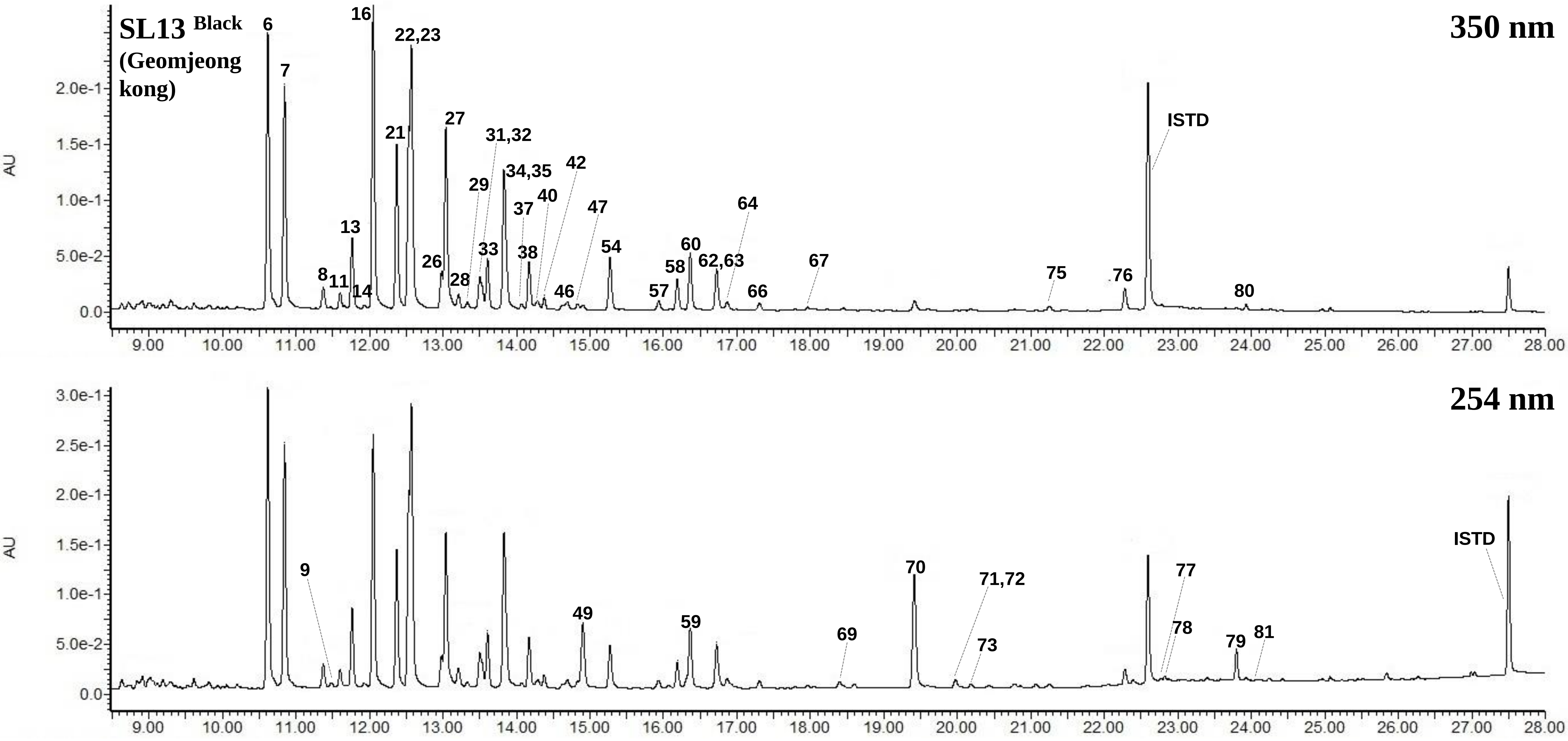

SL13 Black
(Geomjeong kong)
350 nm
254 nm
AU
ISTD
0.0
5.0e-2
1.0e-1
1.5e-1
2.0e-1
2.5e-1
3.0e-1
9.00
10.00
11.00
12.00
13.00
14.00
15.00
16.00
17.00
18.00
19.00
20.00
21.00
22.00
23.00
24.00
25.00
26.00
27.00
28.00
6
7
8
11
13
14
16
21
22,23
26
27
28
29
31,32
33
34,35
37
38
40
42
46
47
54
57
58
60
62,63
64
66
67
75
76
80
9
49
59
69
70
71,72
73
77
78
79
81

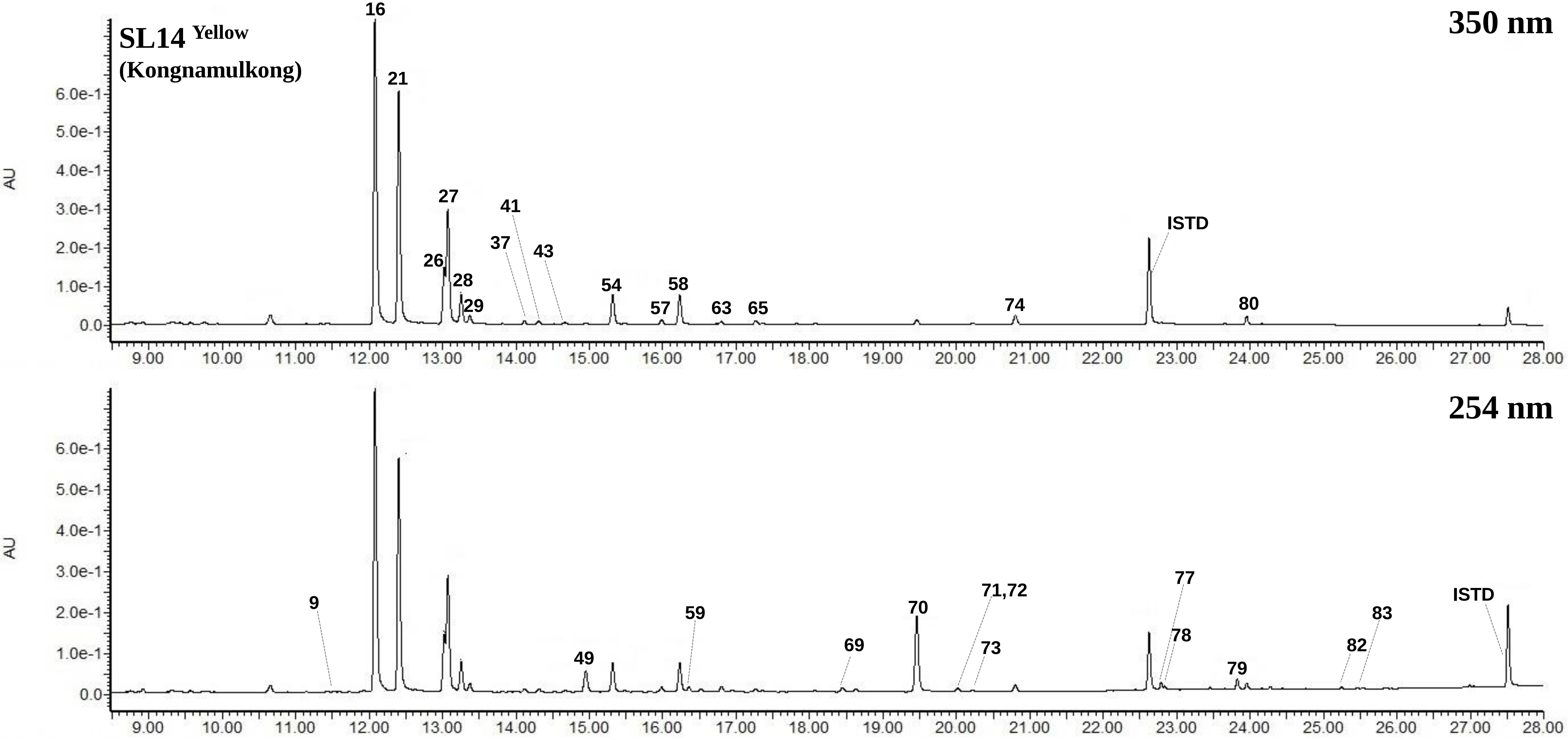
SL14 Yellow
(Kongnamulkong)
350 nm
254 nm
AU
ISTD
16
21
26
27
28
29
37
41
43
54
57
58
63
65
74
80
9
49
59
69
70
71,72
73
77
78
79
82
83

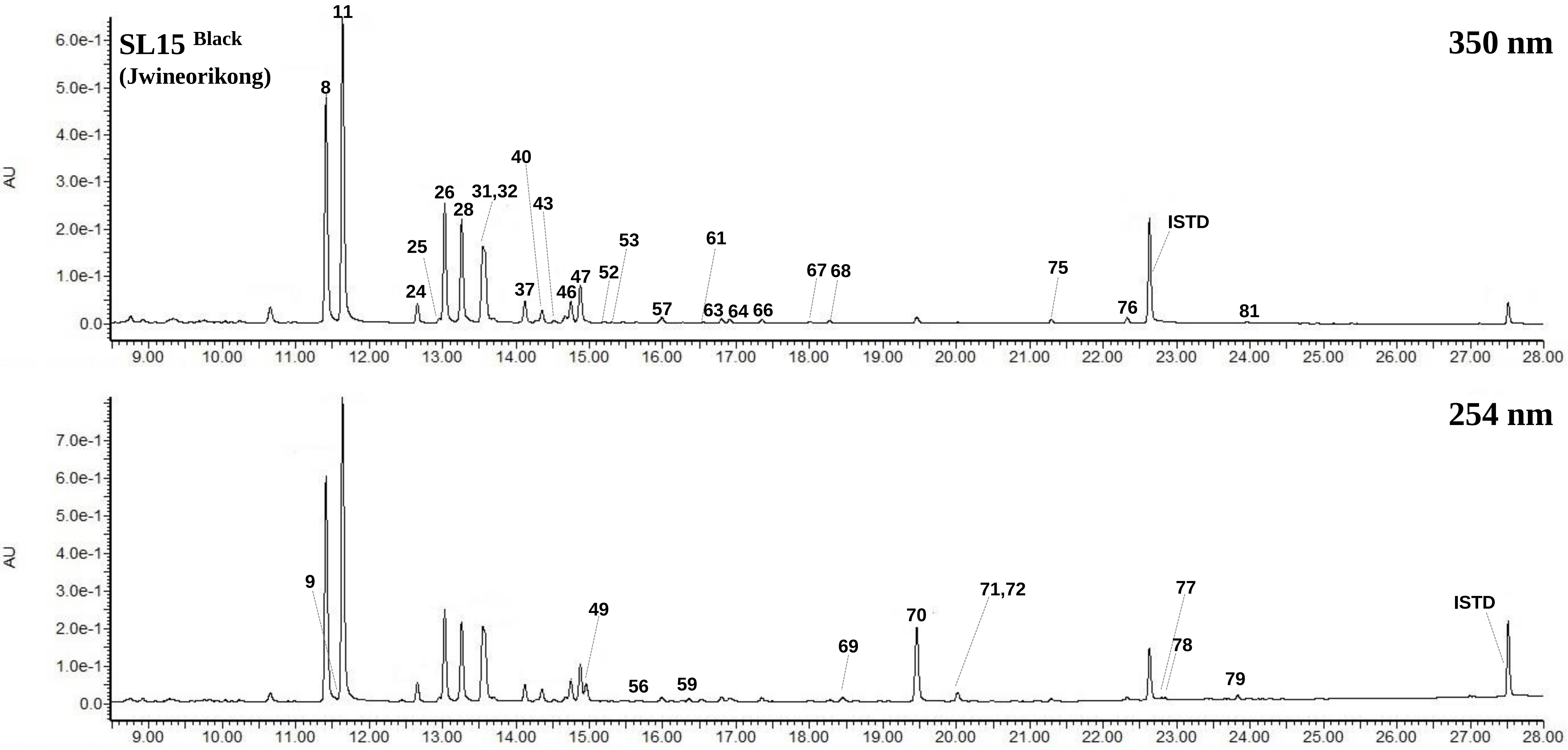
SL15 Black
(Jwineorikong)
350 nm
254 nm
AU
6.0e-1
5.0e-1
4.0e-1
3.0e-1
2.0e-1
1.0e-1
0.0
7.0e-1
9.00
10.00
11.00
12.00
13.00
14.00
15.00
16.00
17.00
18.00
19.00
20.00
21.00
22.00
23.00
24.00
25.00
26.00
27.00
28.00
11
8
24
25
26
28
31,32
37
40
43
46
47
52
53
57
61
63
64
66
67
68
75
76
ISTD
81
9
49
56
59
69
70
71,72
77
78
79

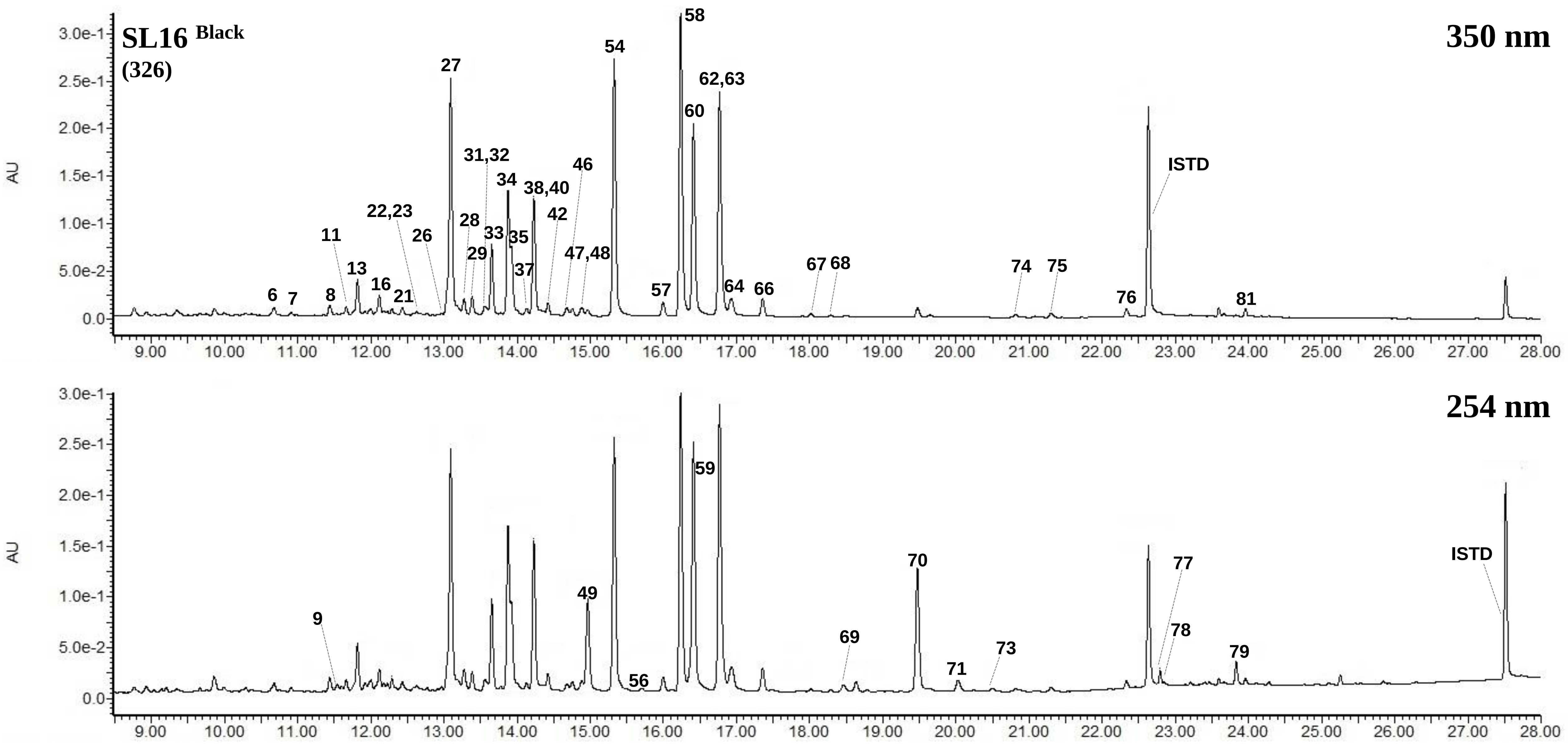

SL16 Black
(326)
350 nm
254 nm
AU
ISTD
3.0e-1
2.5e-1
2.0e-1
1.5e-1
1.0e-1
5.0e-2
0.0
9.00
10.00
11.00
12.00
13.00
14.00
15.00
16.00
17.00
18.00
19.00
20.00
21.00
22.00
23.00
24.00
25.00
26.00
27.00
28.00
6
7
8
11
13
16
21
22,23
26
27
28
29
31,32
33
34
35
37
38,40
42
46
47,48
54
57
58
60
62,63
64
66
67
68
74
75
76
81
9
49
56
59
69
70
71
73
77
78
79

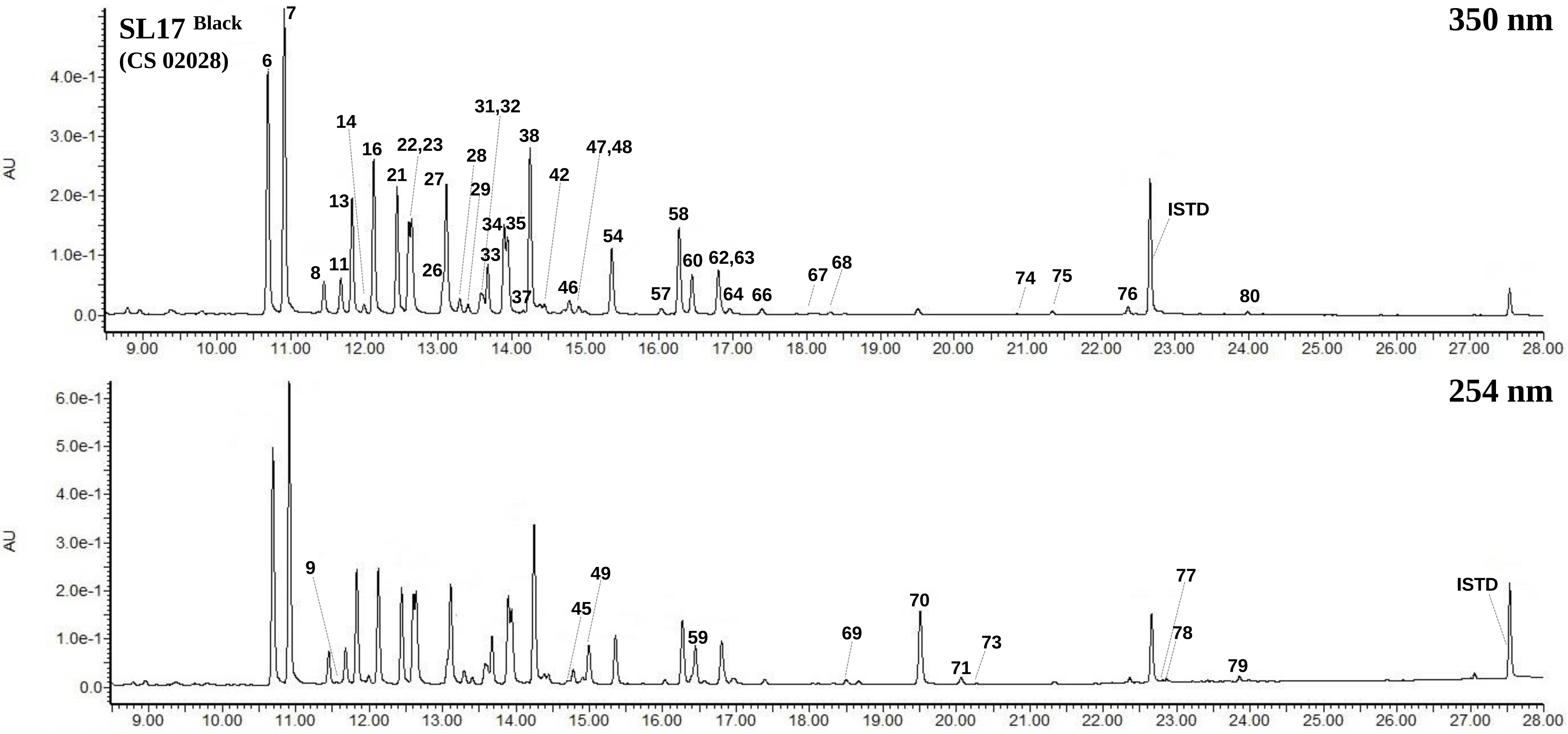
SL17 Black
(CS 02028)
350 nm
254 nm
AU
ISTD
6
7
8
9
11
13
14
16
21
22,23
26
27
28
29
31,32
33
34
35
37
38
42
45
46
47,48
49
54
57
58
59
60
62,63
64
66
67
68
69
70
71
73
74
75
76
77
78
79
80

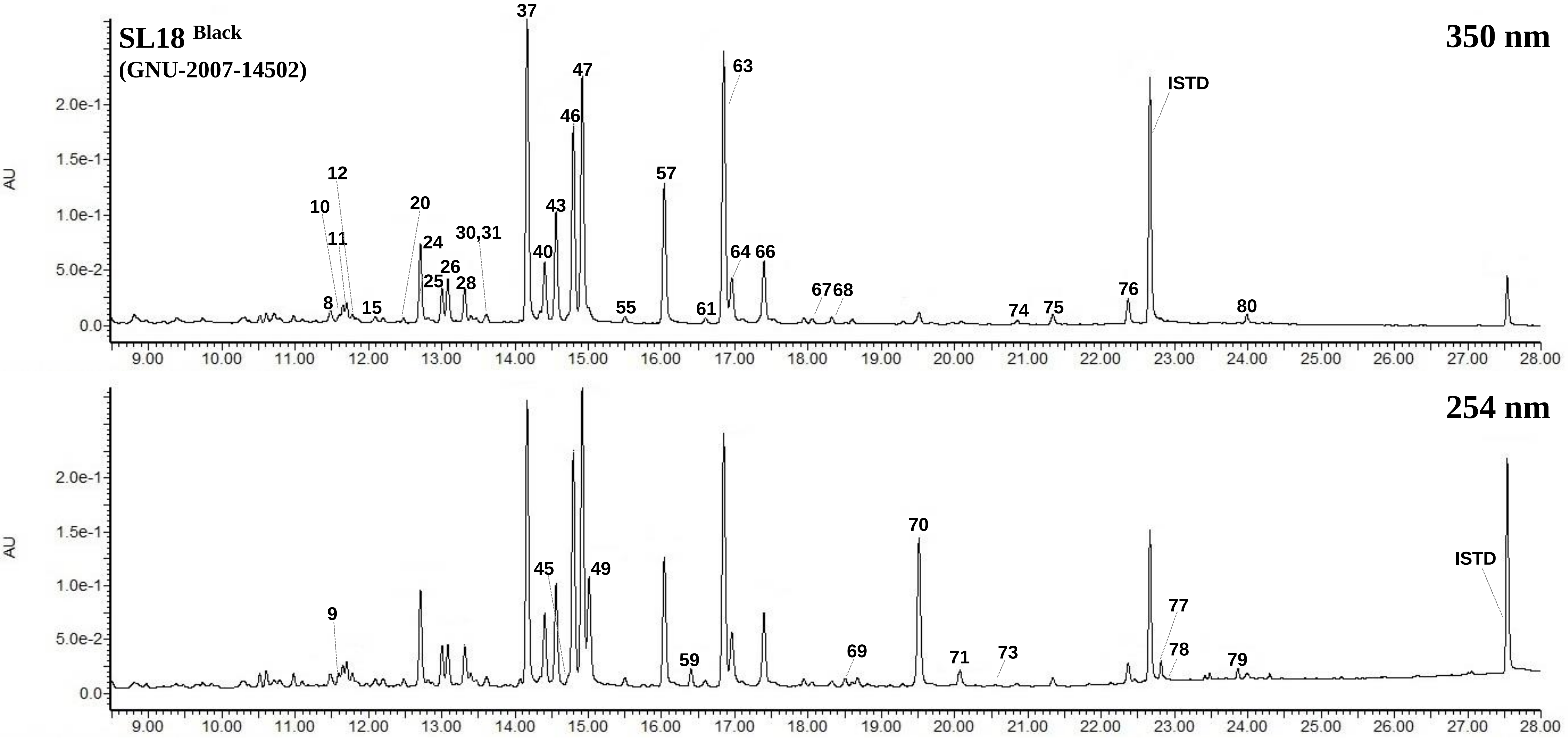
SL18 Black
(GNU-2007-14502)
350 nm
254 nm
AU
ISTD
2.0e-1
1.5e-1
1.0e-1
5.0e-2
0.0
9.00
10.00
11.00
12.00
13.00
14.00
15.00
16.00
17.00
18.00
19.00
20.00
21.00
22.00
23.00
24.00
25.00
26.00
27.00
28.00
8
9
10
11
12
15
20
24
25
26
28
30,31
37
40
43
45
46
47
49
55
57
59
61
63
64
66
67
68
69
70
71
73
74
75
76
77
78
79
80

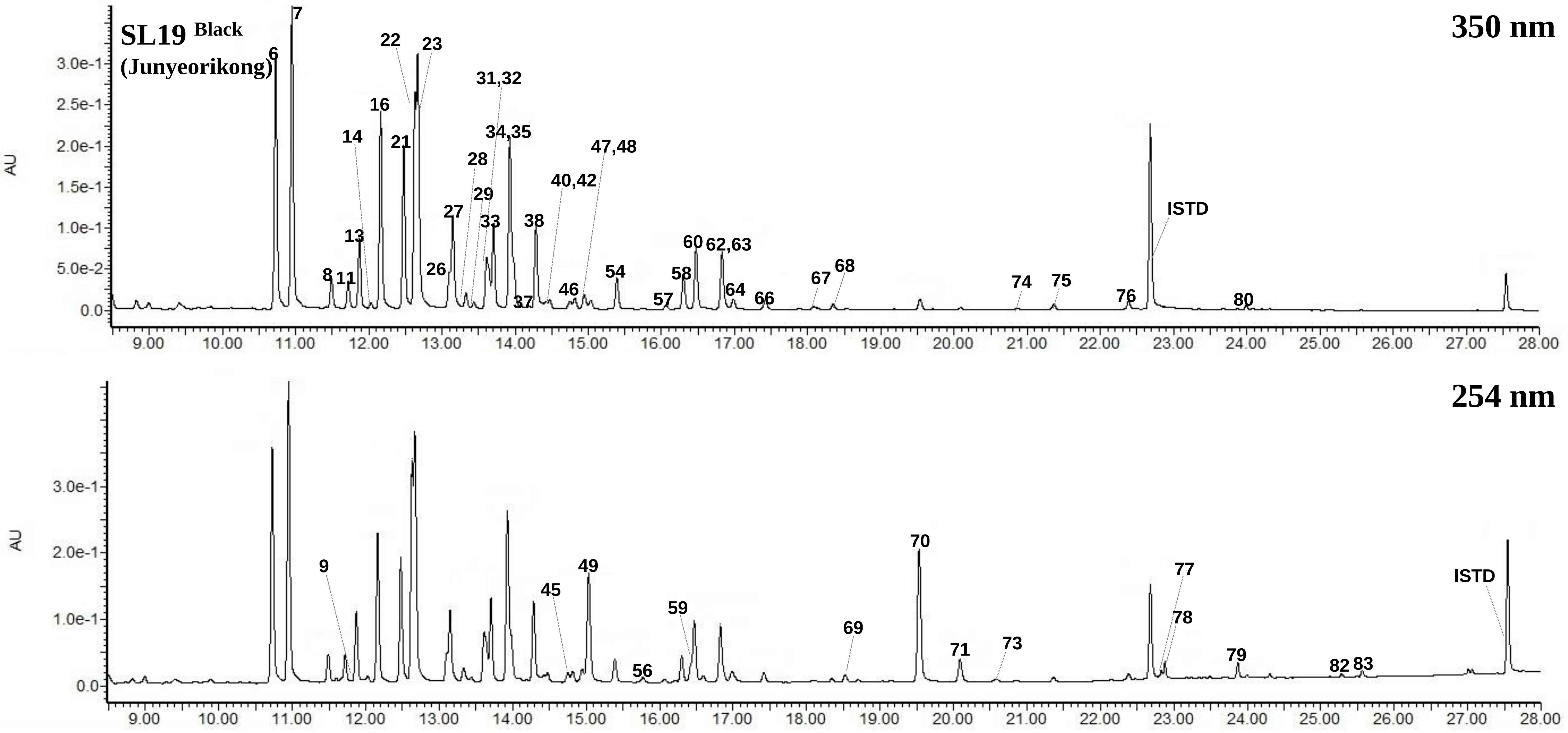

SL19 Black
(Junyeorikong)
350 nm
254 nm
AU
3.0e-1
2.5e-1
2.0e-1
1.5e-1
1.0e-1
5.0e-2
0.0
9.00 10.00 11.00 12.00 13.00 14.00 15.00 16.00 17.00 18.00 19.00 20.00 21.00 22.00 23.00 24.00 25.00 26.00 27.00 28.00
6 7 8 11 13 14 16 21 22 23 26 27 28 29 31,32 33 34,35 37 38 40,42 46 47,48 54 57 58 60 62,63 64 66 67 68 74 75 76 ISTD 80
9 45 49 56 59 69 70 71 73 77 78 79 82 83 ISTD

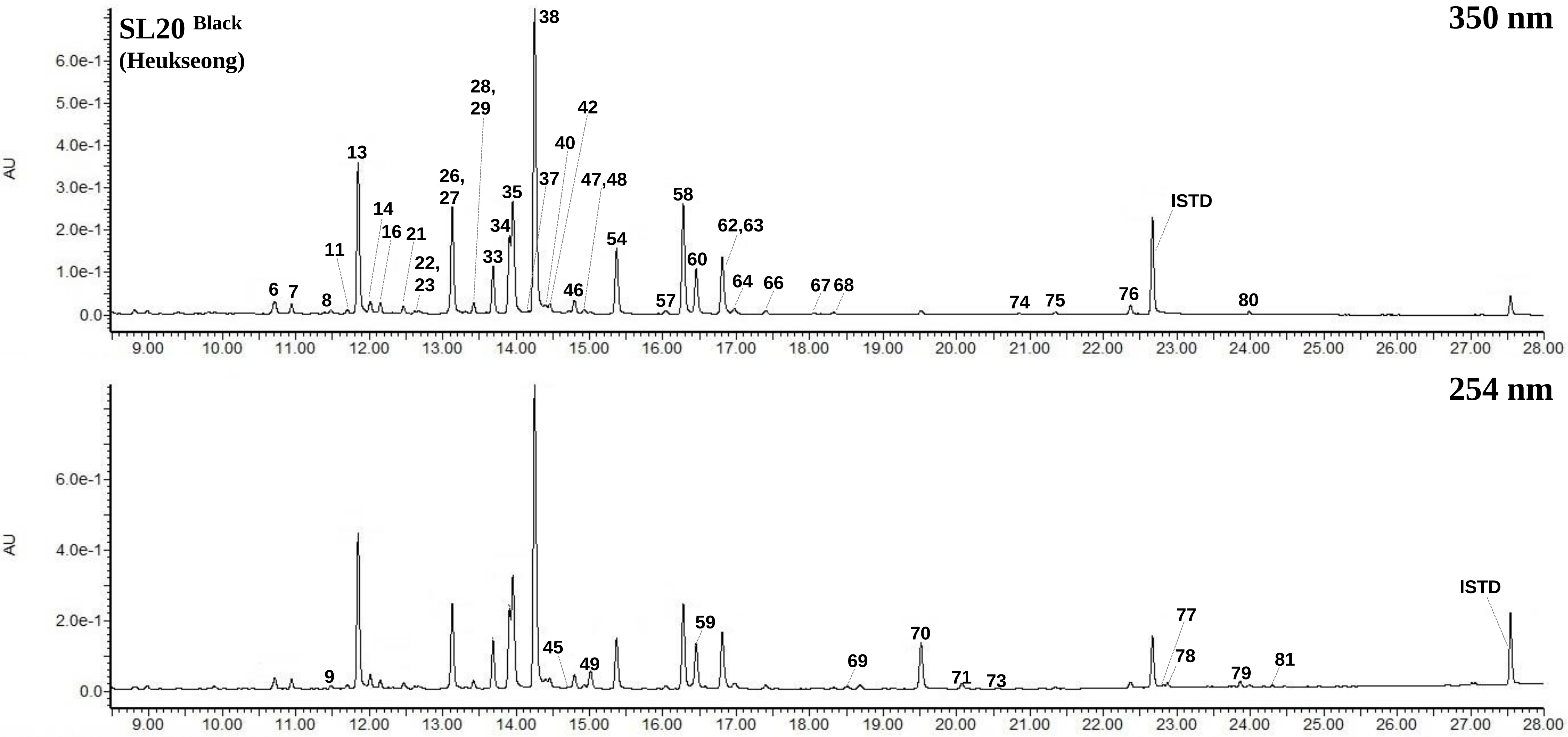
SL20 Black
(Heukseong)
350 nm
254 nm
AU
6.0e-1
5.0e-1
4.0e-1
3.0e-1
2.0e-1
1.0e-1
0.0
9.00
10.00
11.00
12.00
13.00
14.00
15.00
16.00
17.00
18.00
19.00
20.00
21.00
22.00
23.00
24.00
25.00
26.00
27.00
28.00
6
7
8
11
13
14
16
21
22, 23
26, 27
28, 29
33
34
35
37
38
40
42
46
47,48
54
57
58
60
62,63
64
66
67
68
74
75
76
ISTD
80
9
45
49
59
69
70
71
73
77
78
79
81

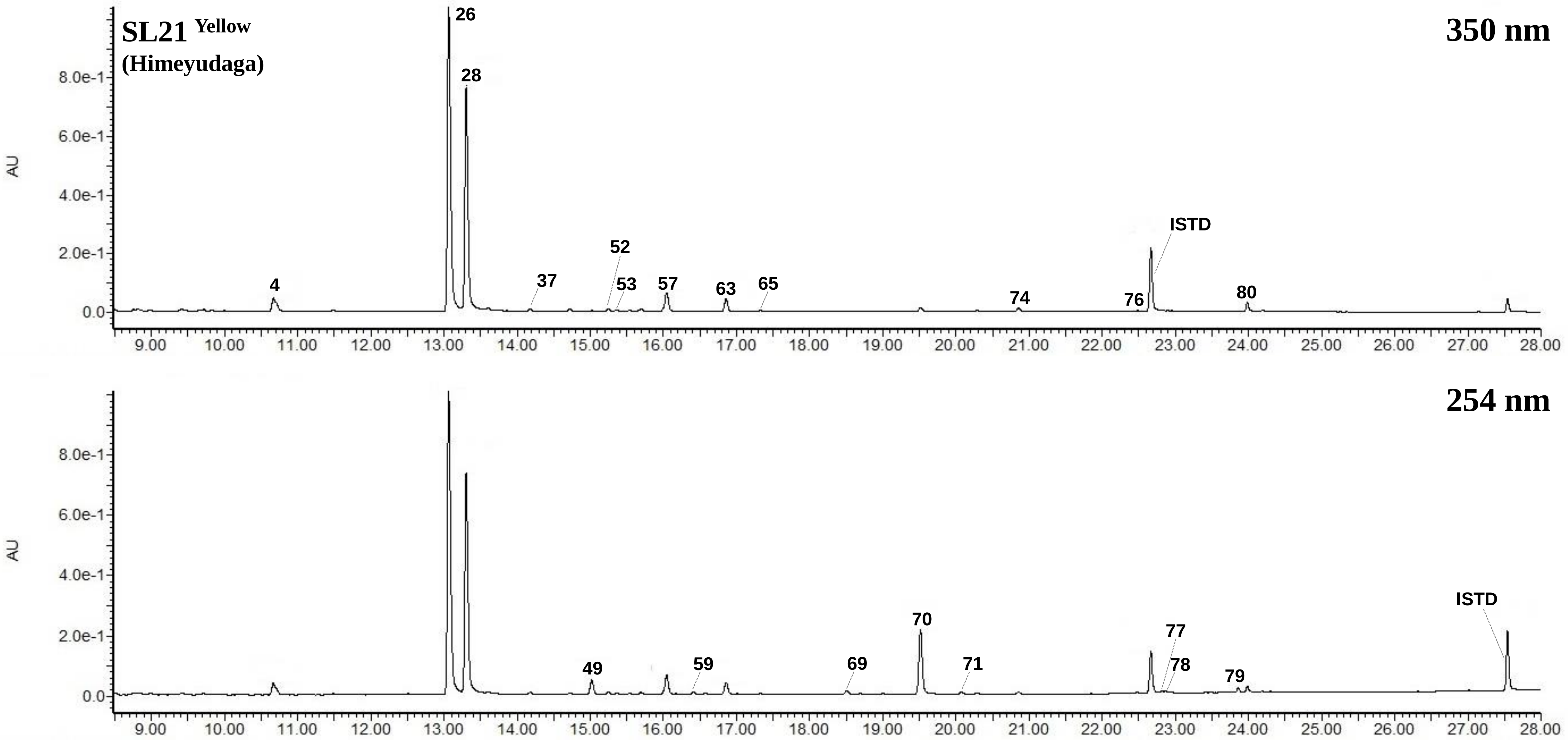

SL21 Yellow
(Himeyudaga)
350 nm
254 nm
AU
26
28
4
37
52
53
57
63
65
74
76
ISTD
80
49
59
69
70
71
77
78
79
ISTD
