# Supplementary material for: Comprehensive characterization of flavonoid derivatives in young leaves of core-collected soybean (Glycine max L.) cultivars based on high-resolution mass spectrometry
Source: Sci Rep. 2022 Aug 29;12:14678. doi: 10.1038/s41598-022-18226-4 (PMC9424525; doi:10.1038/s41598-022-18226-4)

Supplementary Figure S2. Positive fragmentation of the 55 flavonol derivatives identified from young leaves of 21 soybean cultivars. **K**, kaempferol; **Q**, quercetin; **I**, isorhamnetin; gal, galactoside (galactosyl); glu, glucoside (glucosyl); rham, rhamnoside (rhamnosyl); gen, gentiobiose; neo, neohesperidose; rob, robinobiose; rut, rutinose; sop, sophorose. Compound names of each peak are presented in Table 2.

Peak **1**_Q 3-*O*-(2,6-di-*O*-glu)gal (SL4)

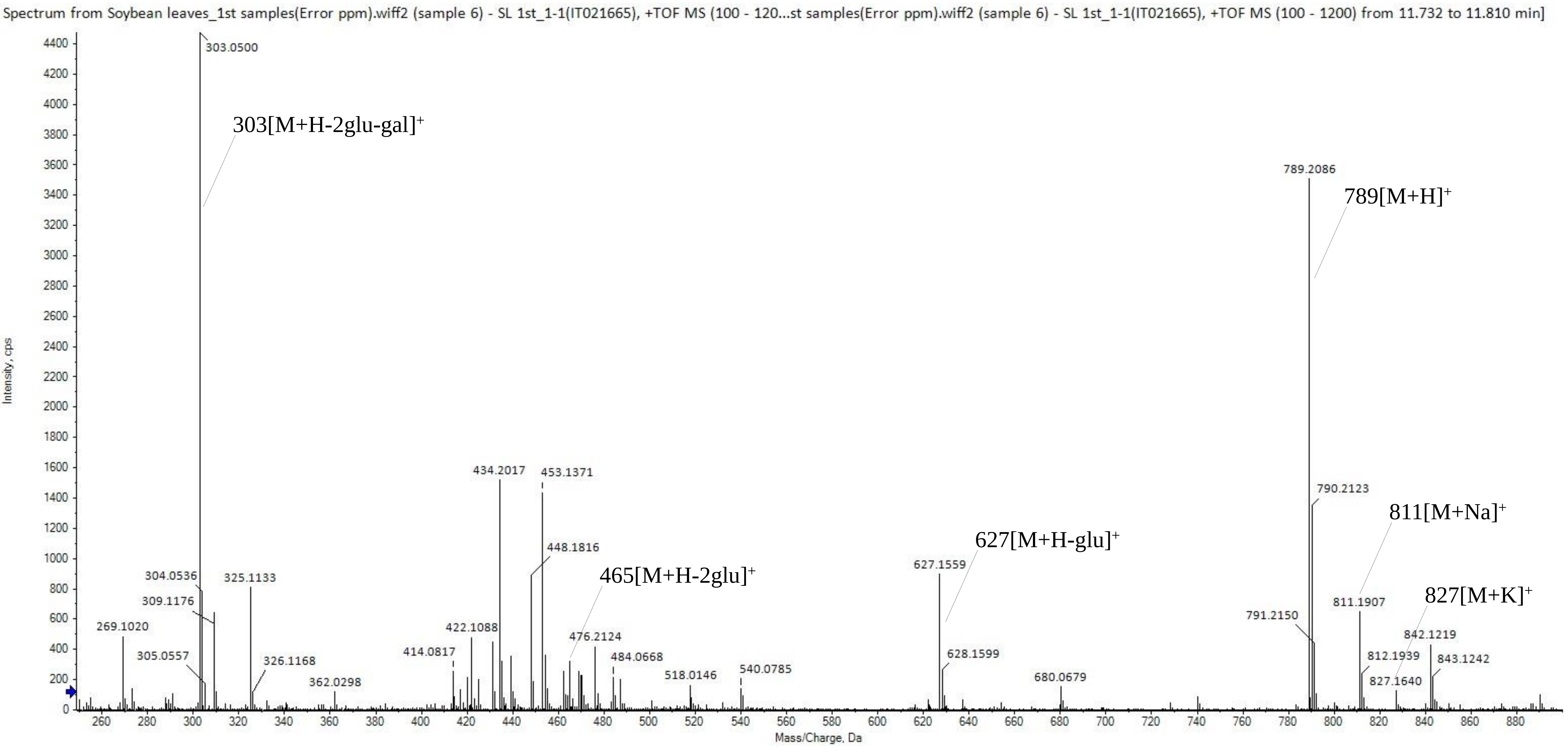

Peak **2**_Q 3-*O*-(2,6-di-*O*-glu)glu (SL4)

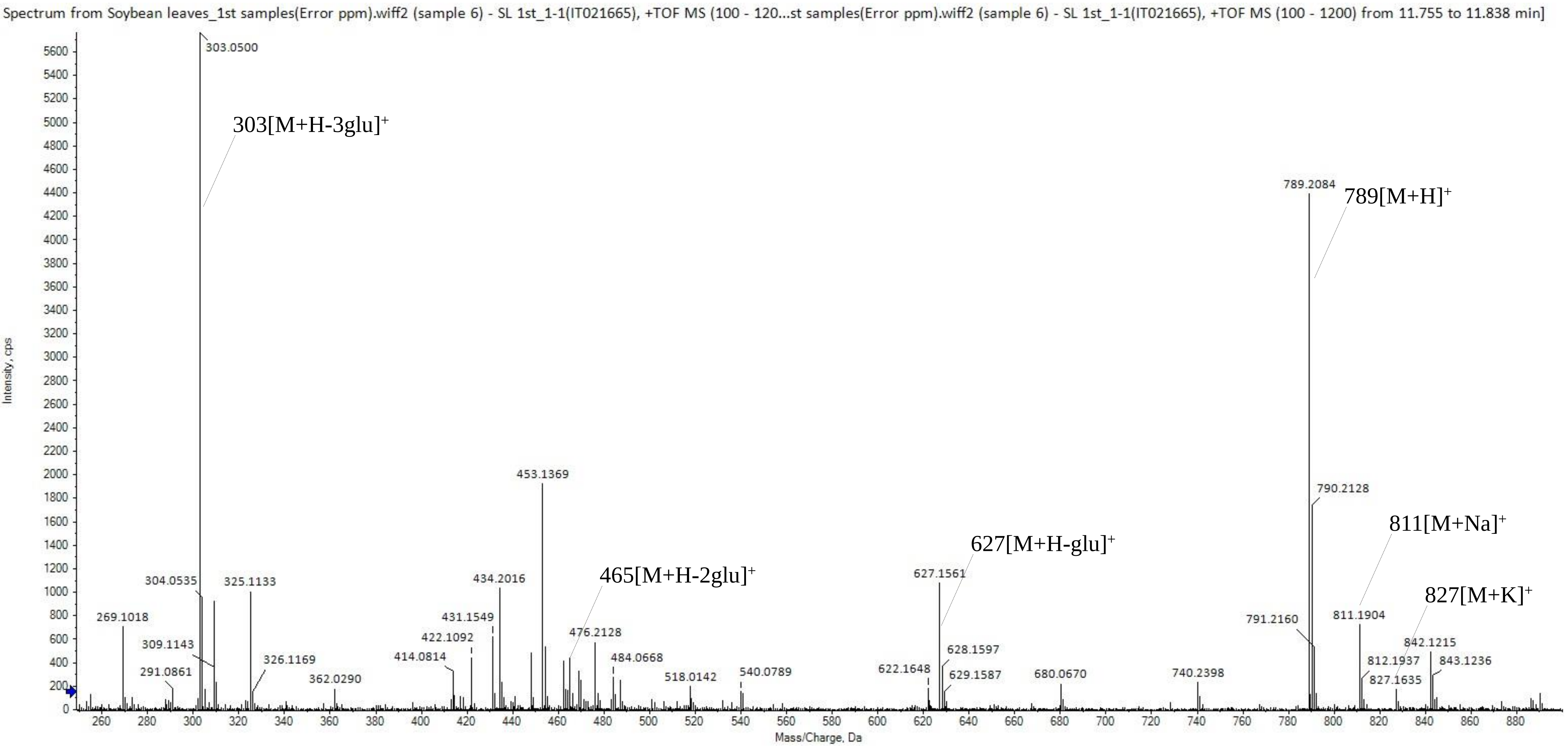

Peak **3**_Q 3-*O*-(2-*O*-rham-6-*O*-glu)gal (SL4)

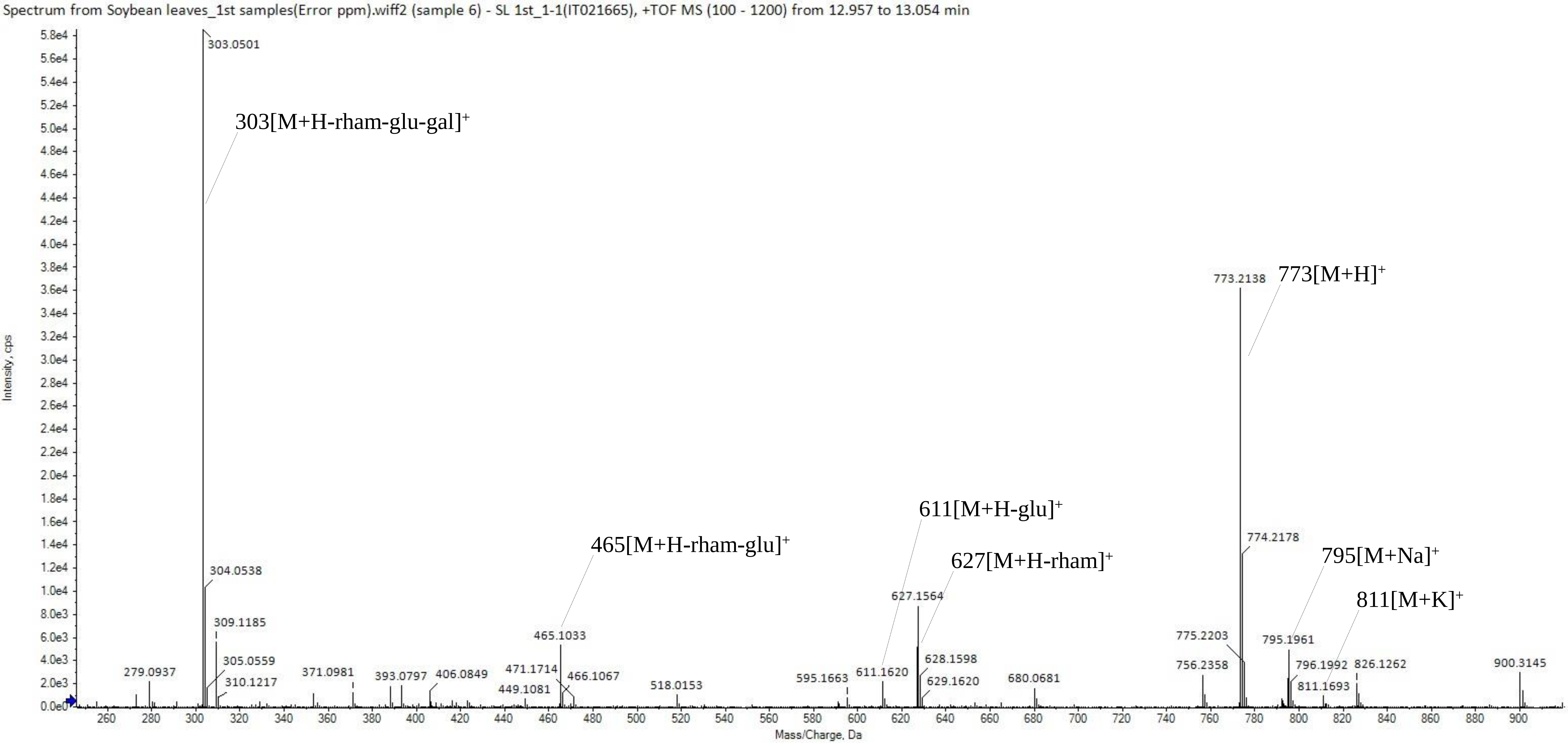

Peak **4**_K 3-*O*-(2,6-di-*O*-glu)glu (SL4)

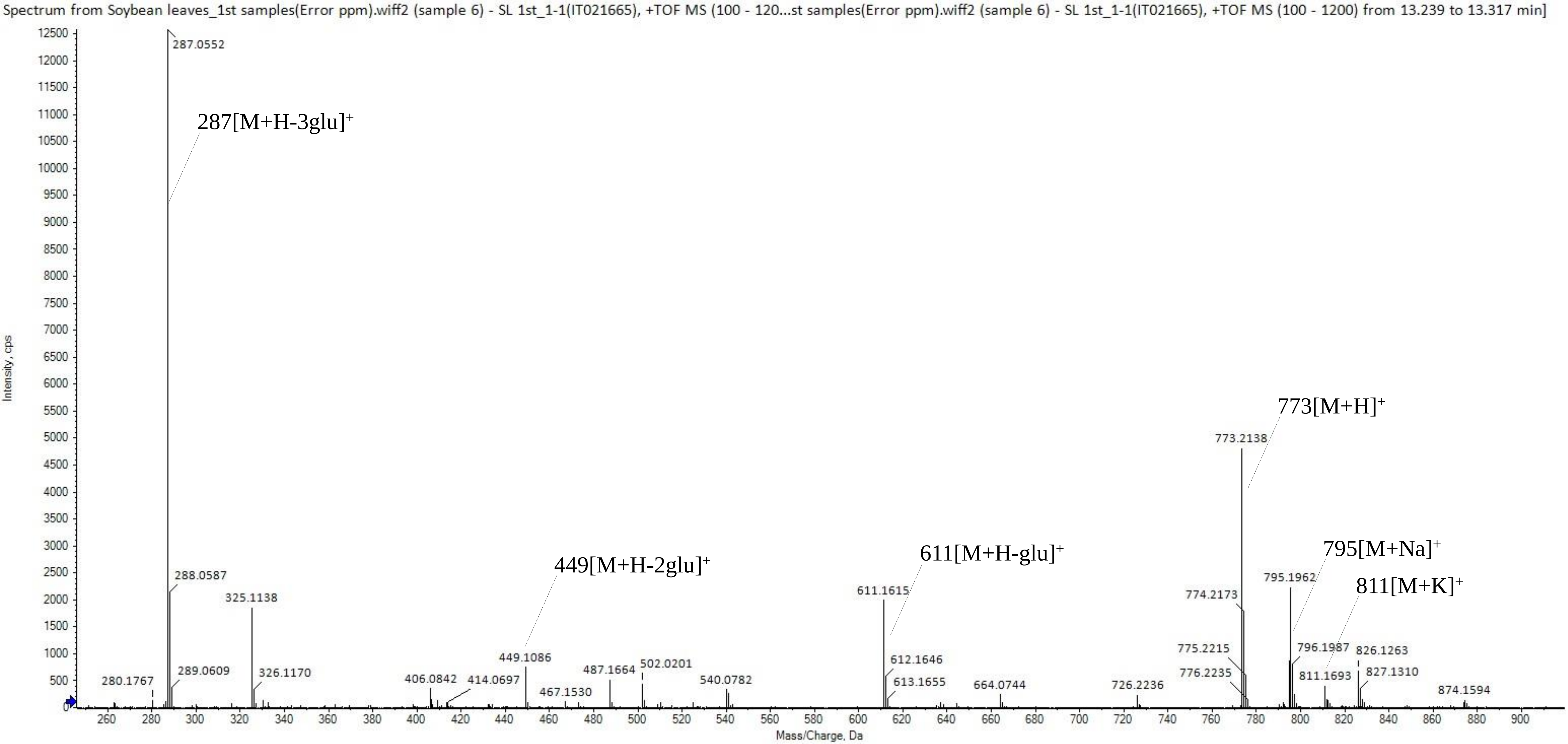

Peak **5**_Q 3-*O*-(2-*O*-rham-6-*O*-glu)glu (SL4)

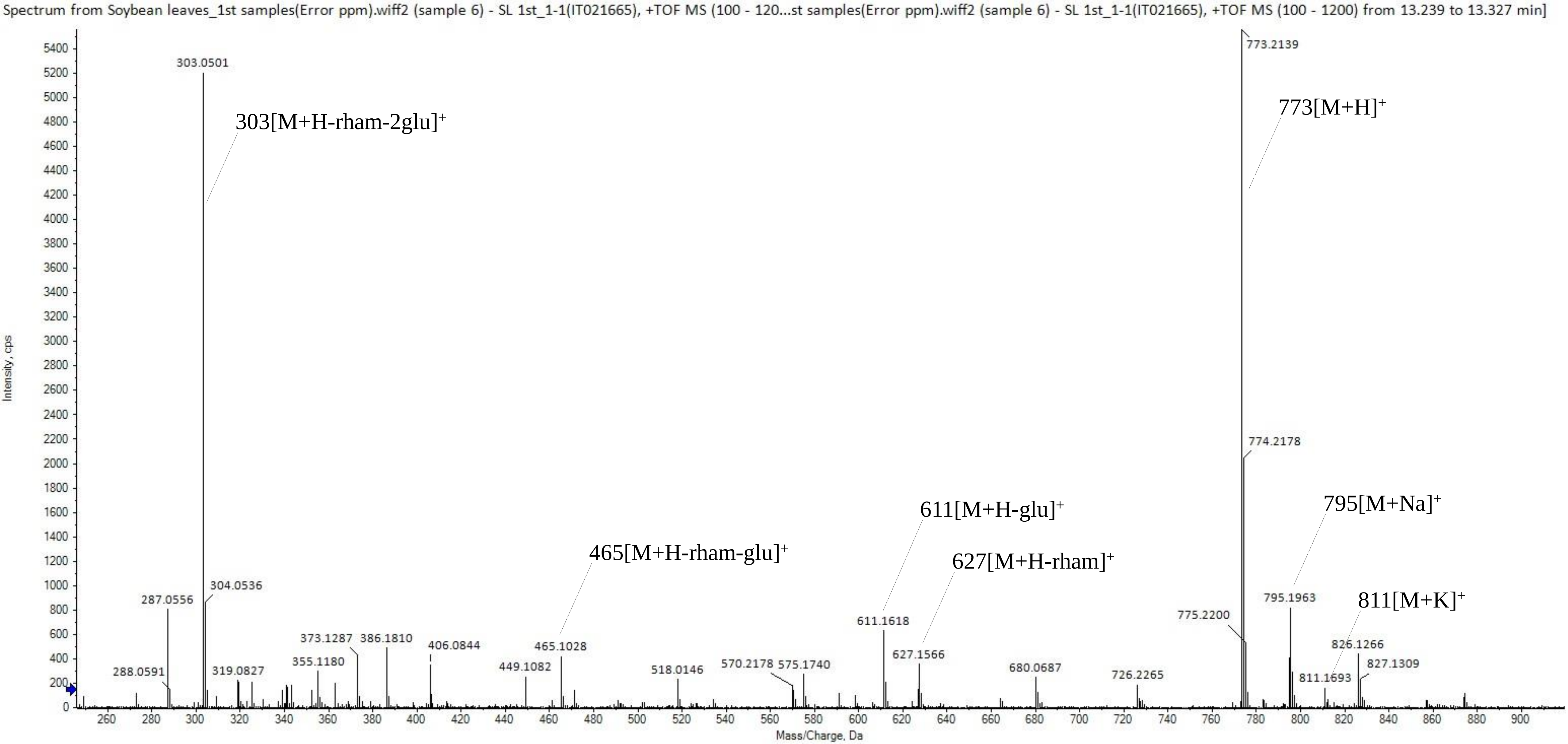

Peak **6**_Q 3-*O*-(2-*O*-glu-6-*O*-rham)gal (SL3)

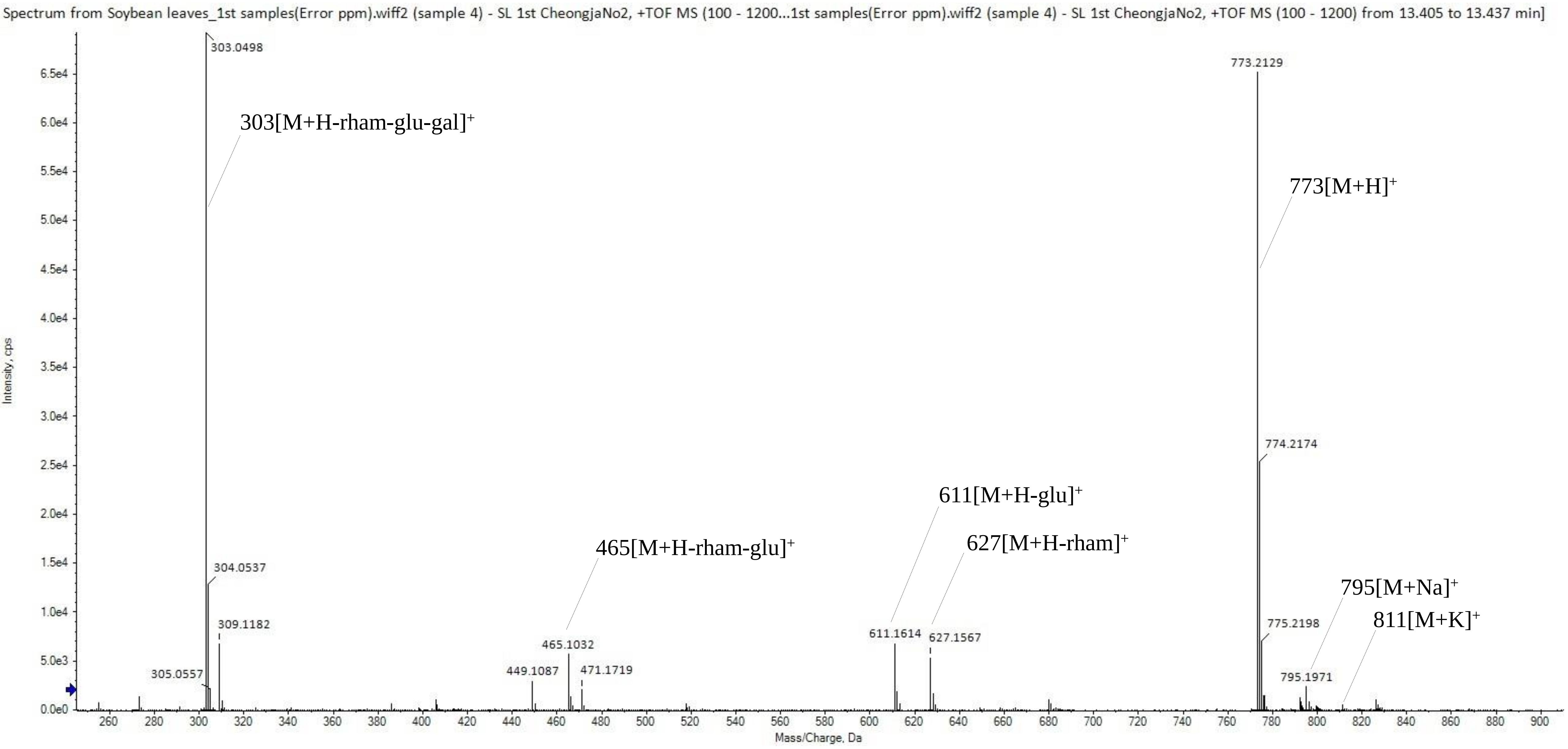

Peak **7**_Q 3-*O*-(2-*O*-glu-6-*O*-rham)glu (SL3)

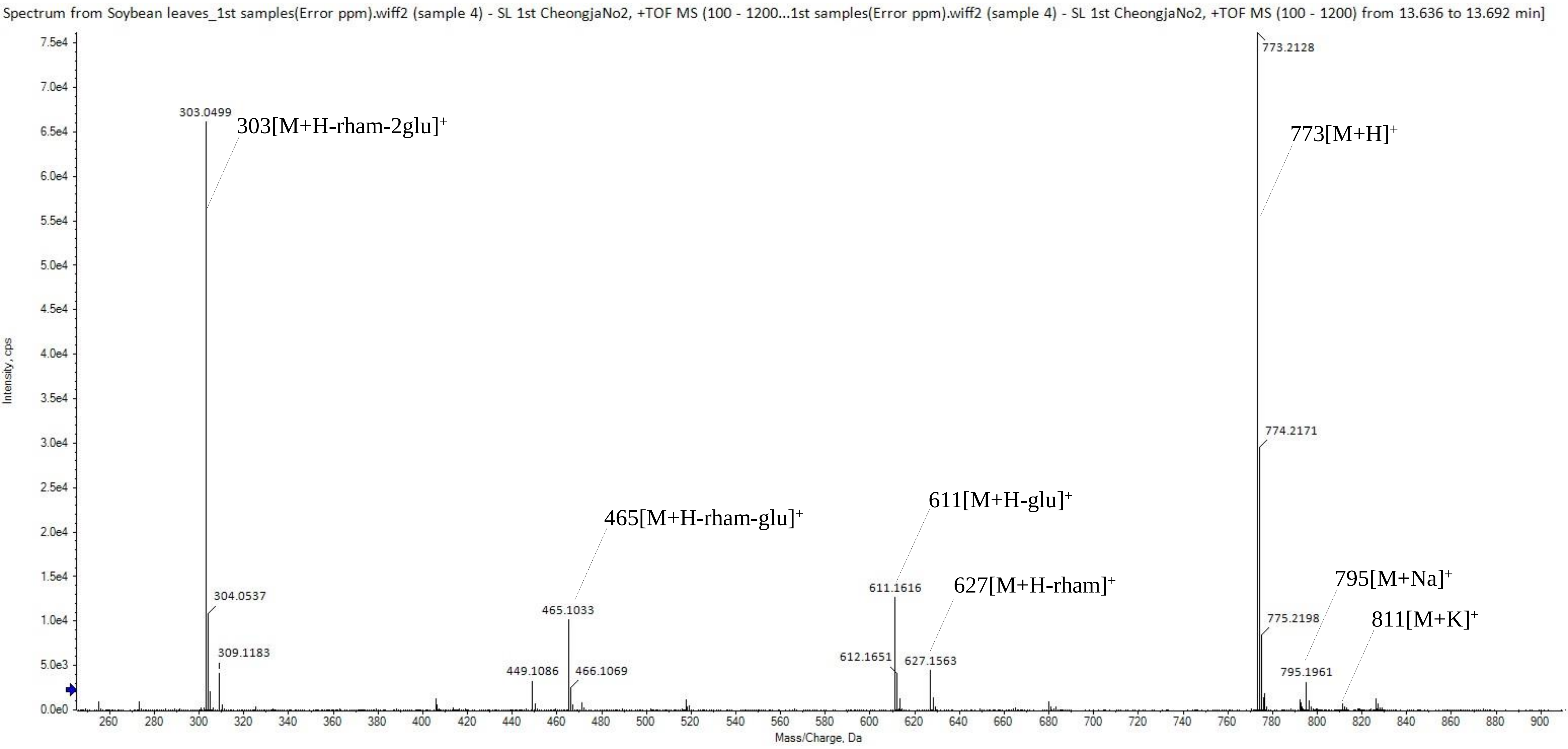

Peak **8**_Q 3-*O*-(2-*O*-glu)gal (SL3)

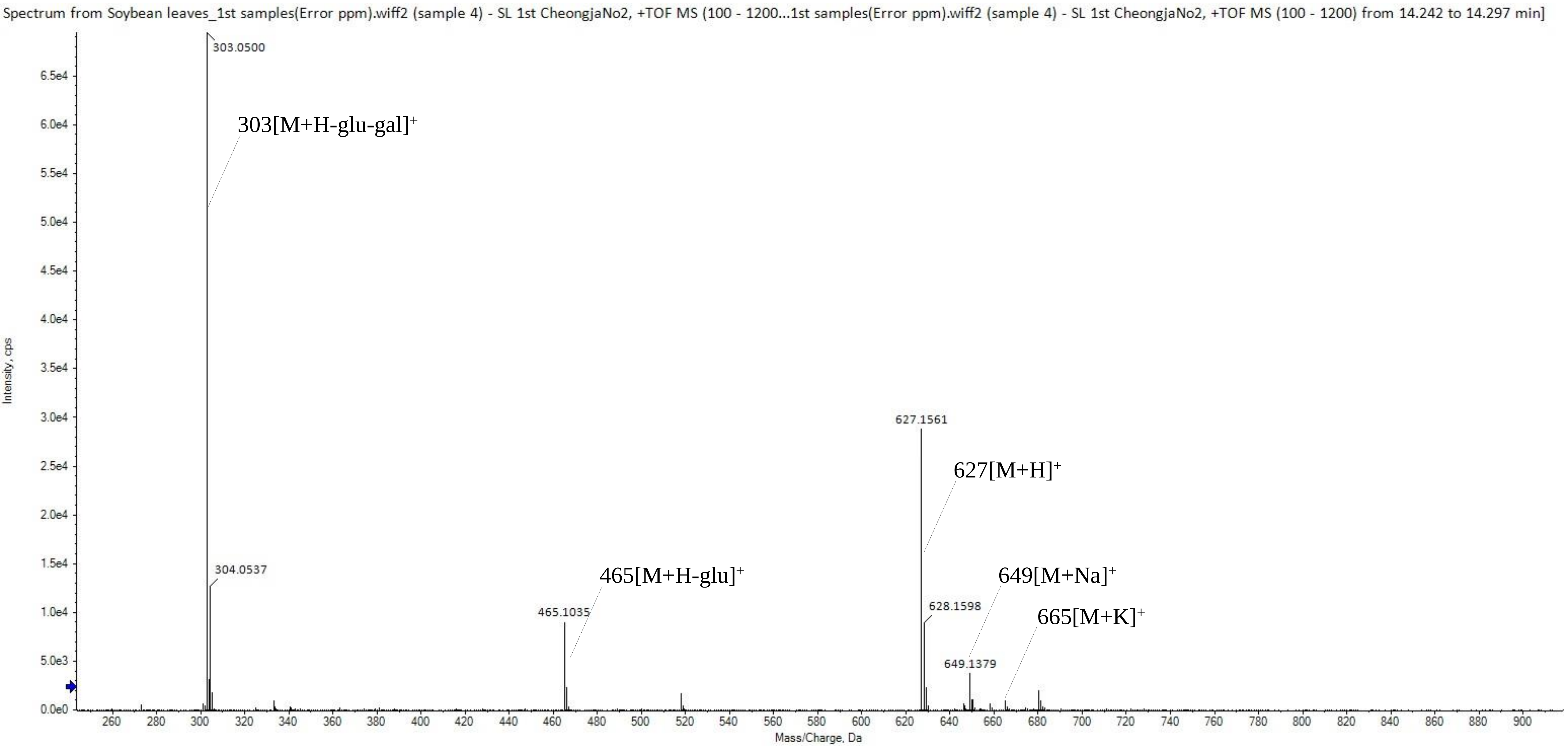

Peak **10**_K 3-*O*-(2-*O*-rham-6-*O*-glu)gal (SL4)

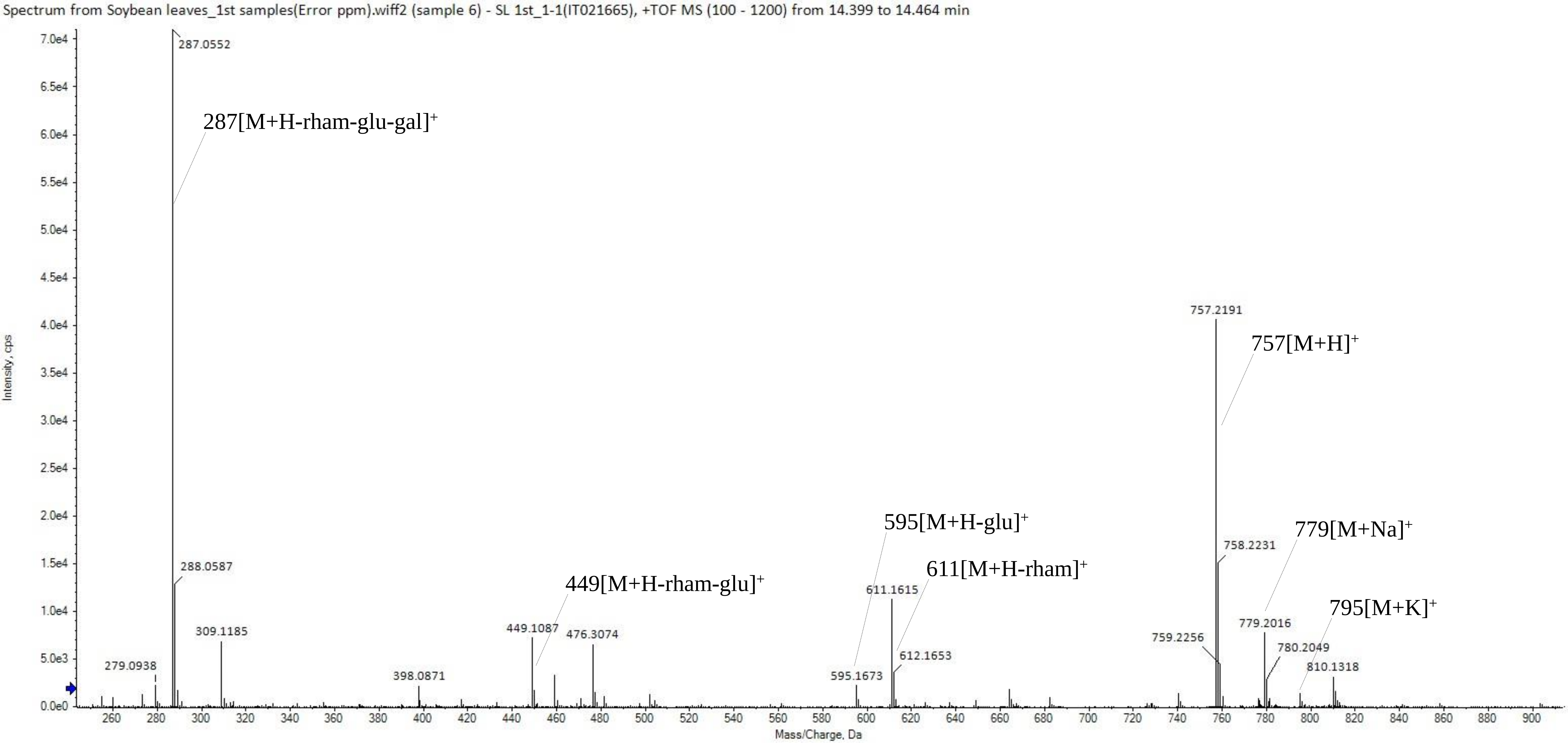

Peak **11**_Q 3-*O*-(2-*O*-glu)glu (Q 3-*O*-sop) (SL3)

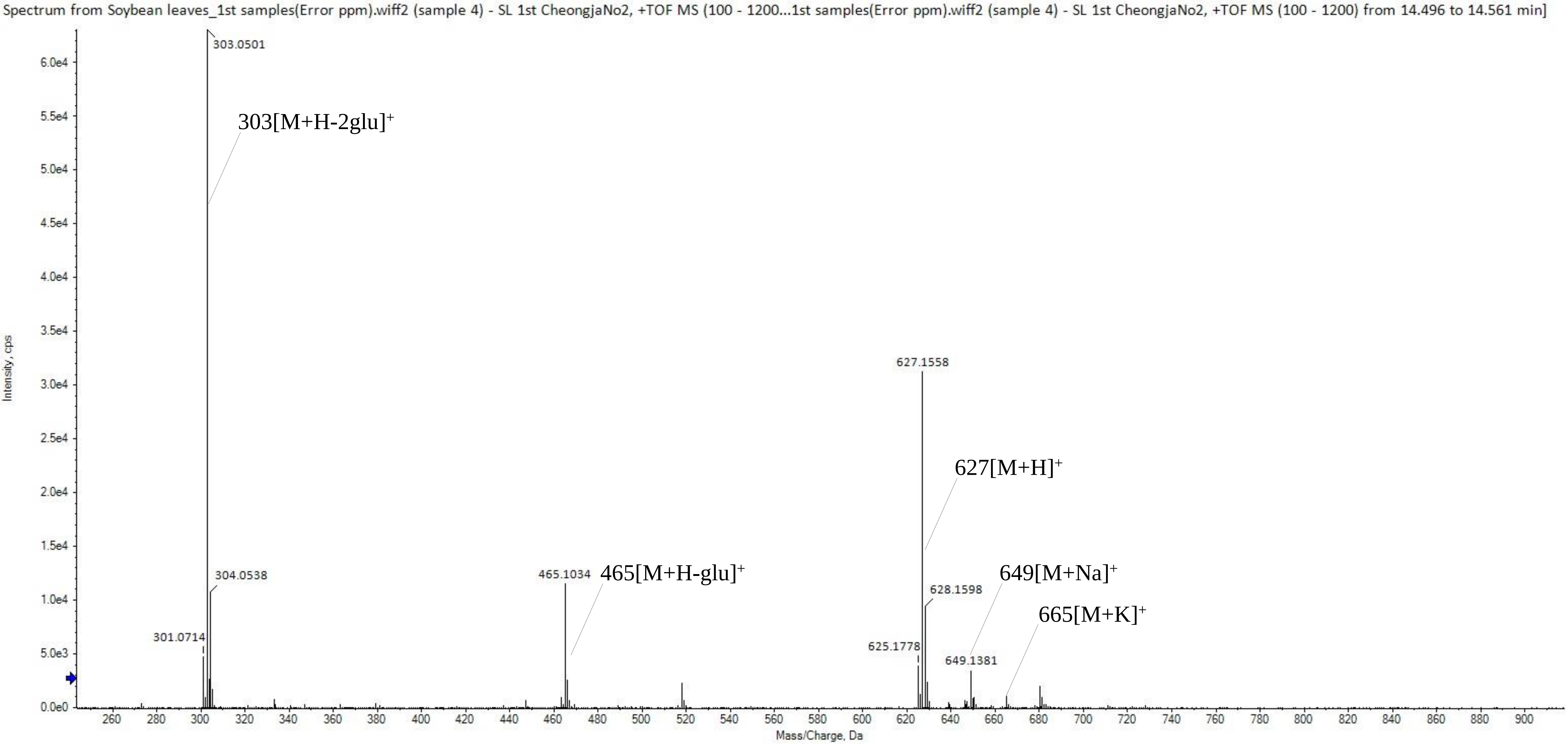

Peak **12**_I 3-*O*-(2-*O*-rham-6-*O*-glu)gal (SL4)

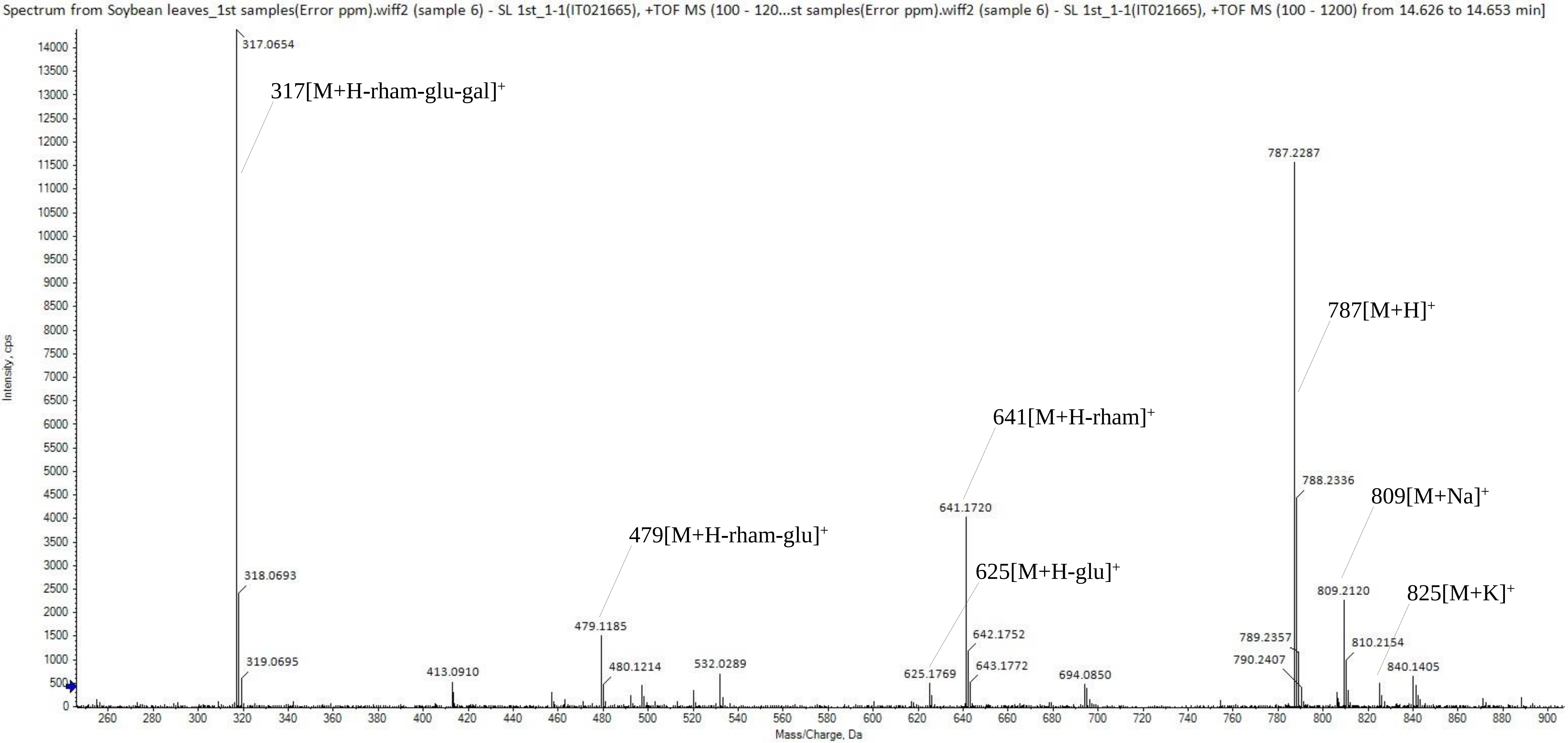

Peak **13**_Q 3-*O*-(2,6-di-*O*-rham)gal (SL1)

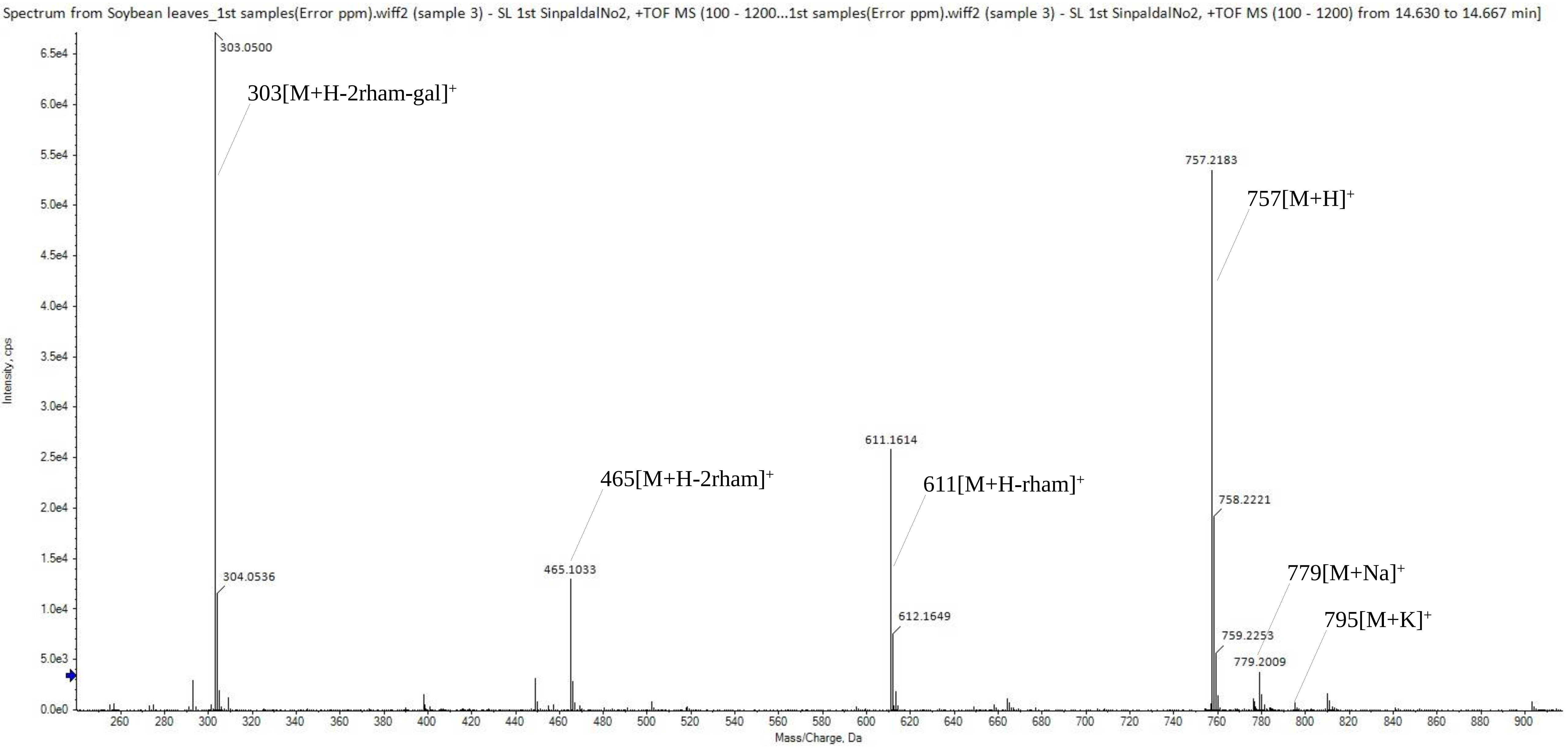

Peak **14**_Q 3-*O*-(4,6-di-*O*-rham)gal (SL20)

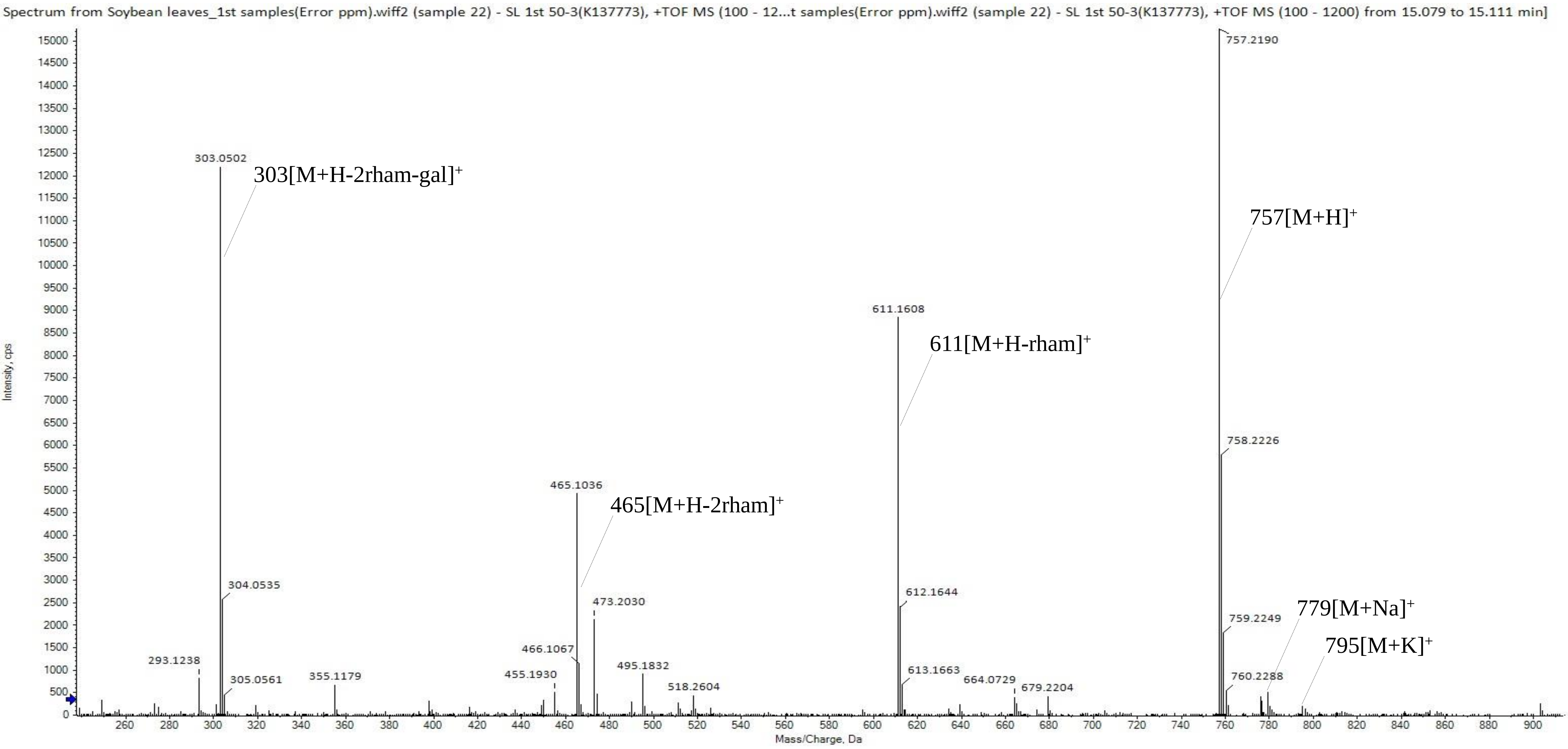

Peak **15**_I 3-*O*-(2-*O*-rham-6-*O*-glu)glu (SL4)

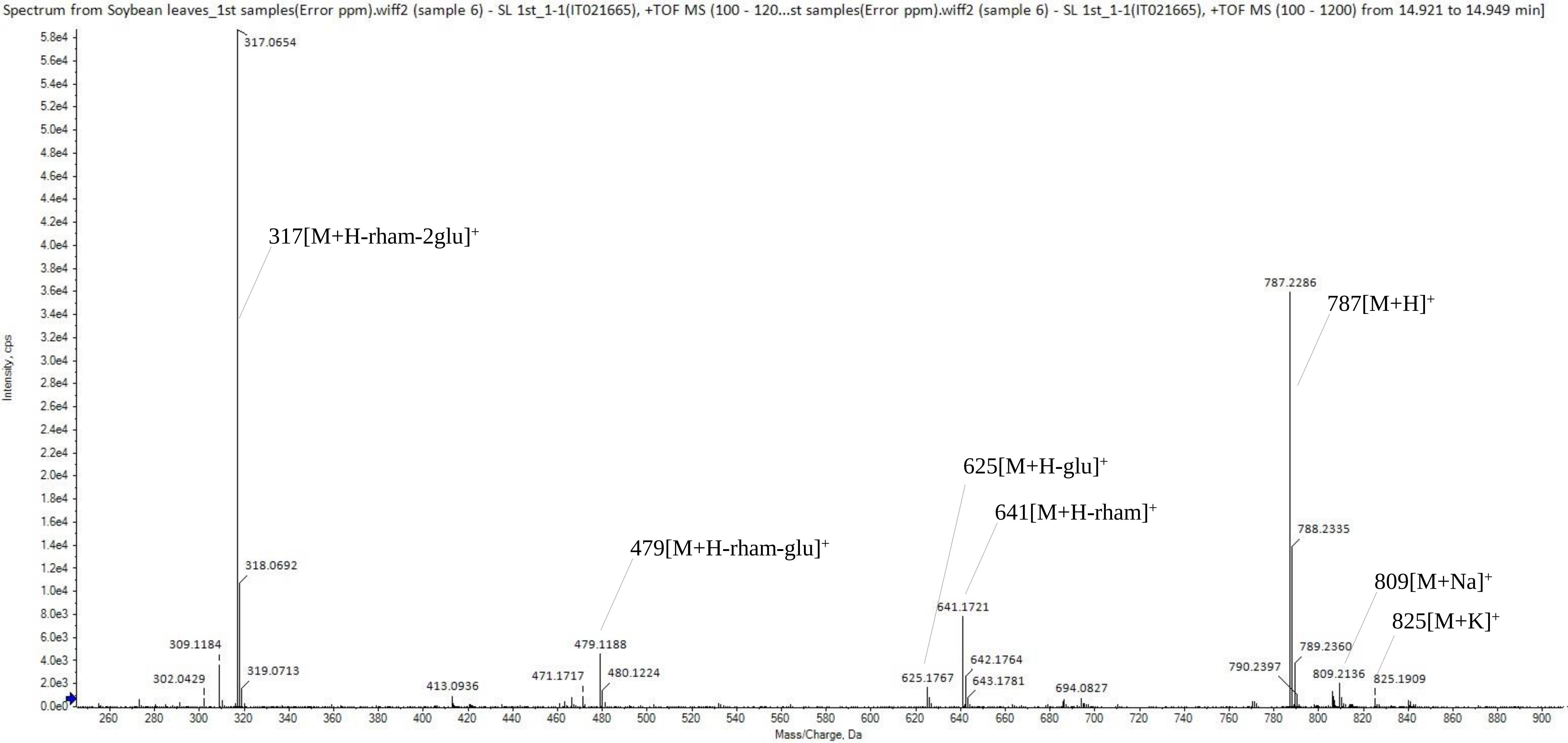

Peak **16**_K 3-*O*-(2-*O*-glu-6-*O*-rham)gal (SL3)

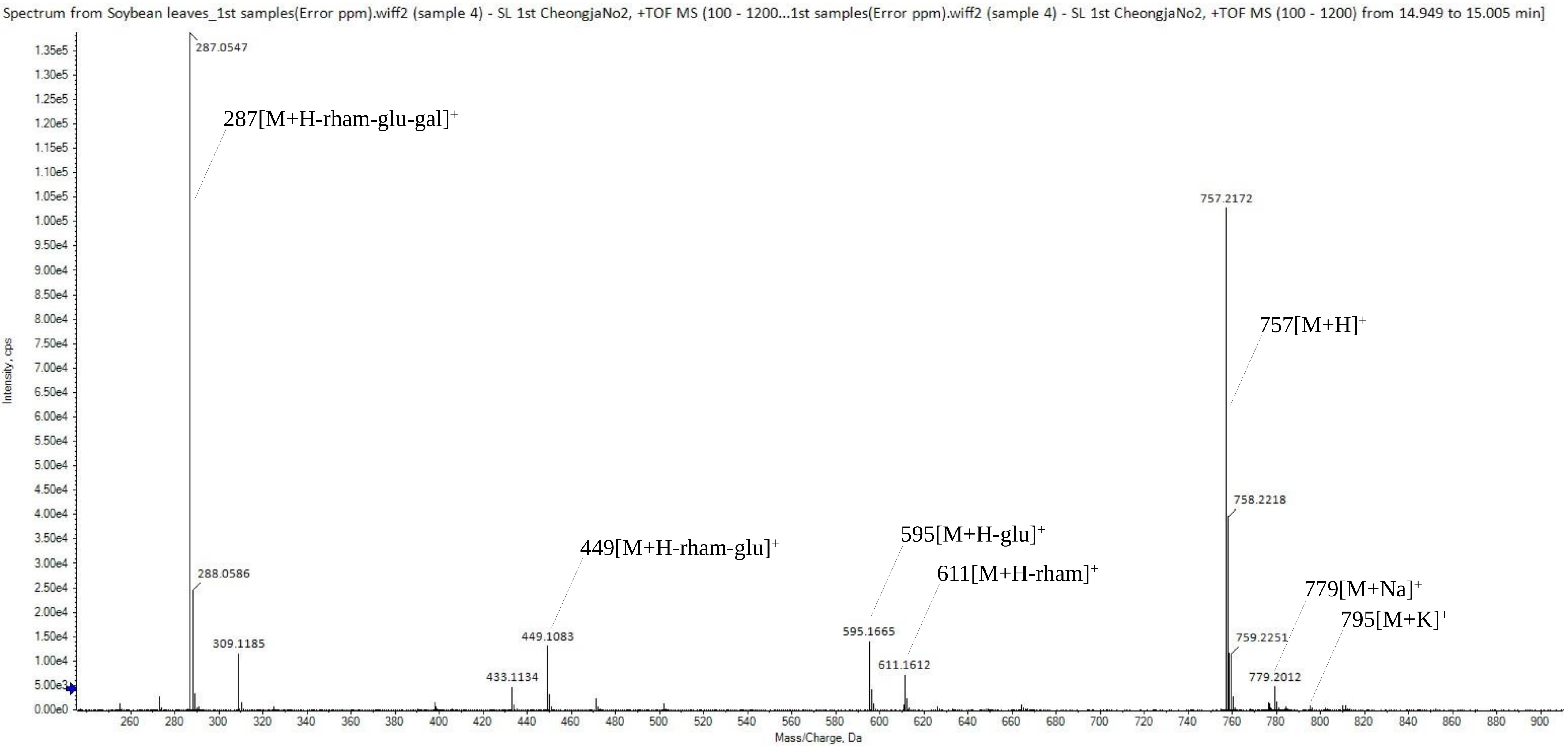

Peak **17**_K 3-*O*-(2-*O*-rham-6-*O*-glu)glu (SL4)

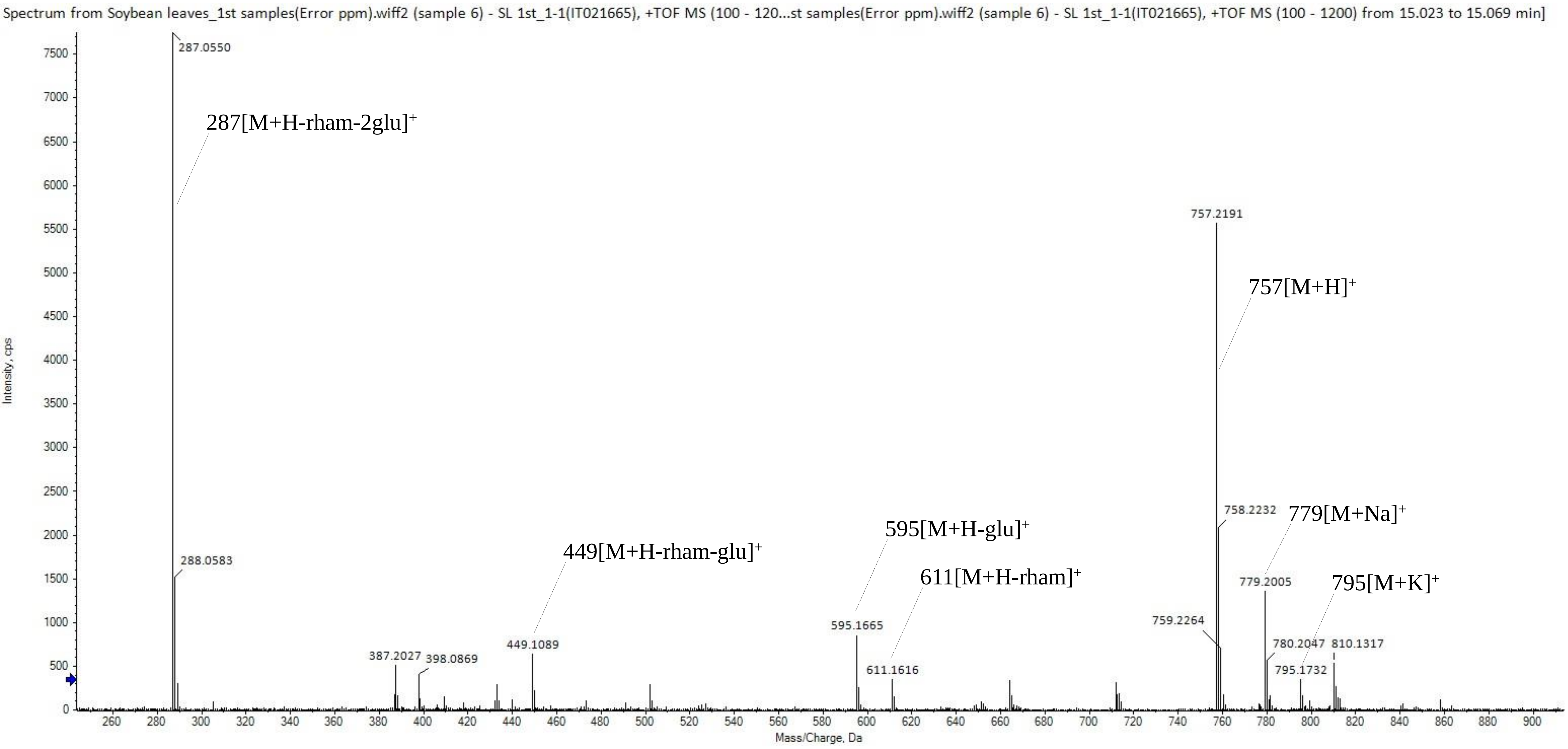

Peak **18**_Q 3-*O*-(6-*O*-glu)gal (SL4)

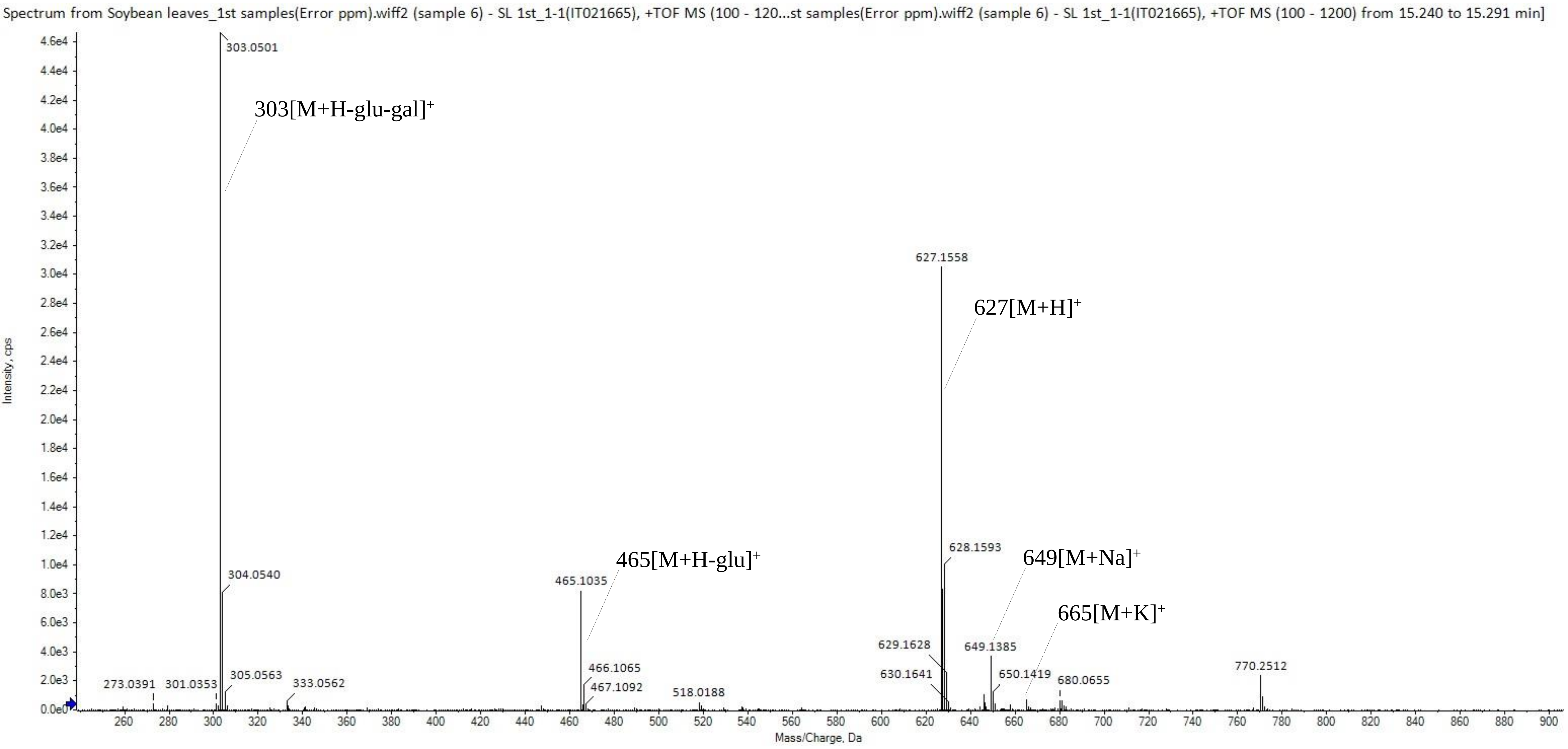

Peak **20**_Q 3-*O*-(6-*O*-glu)glu (Q 3-*O*-gen) (SL4)

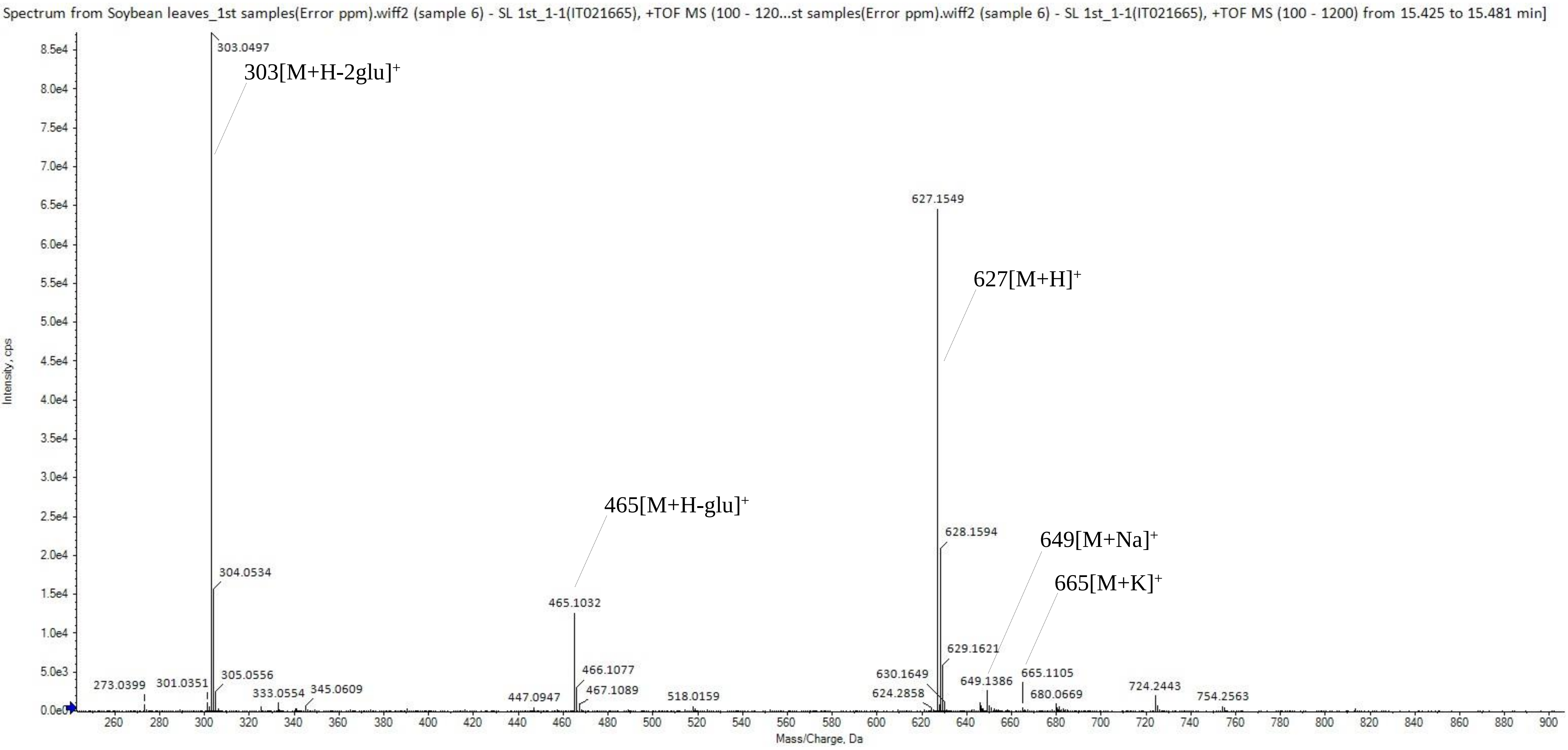

Peak **21**_K 3-*O*-(2-*O*-glu-6-*O*-rham)glu (SL3)

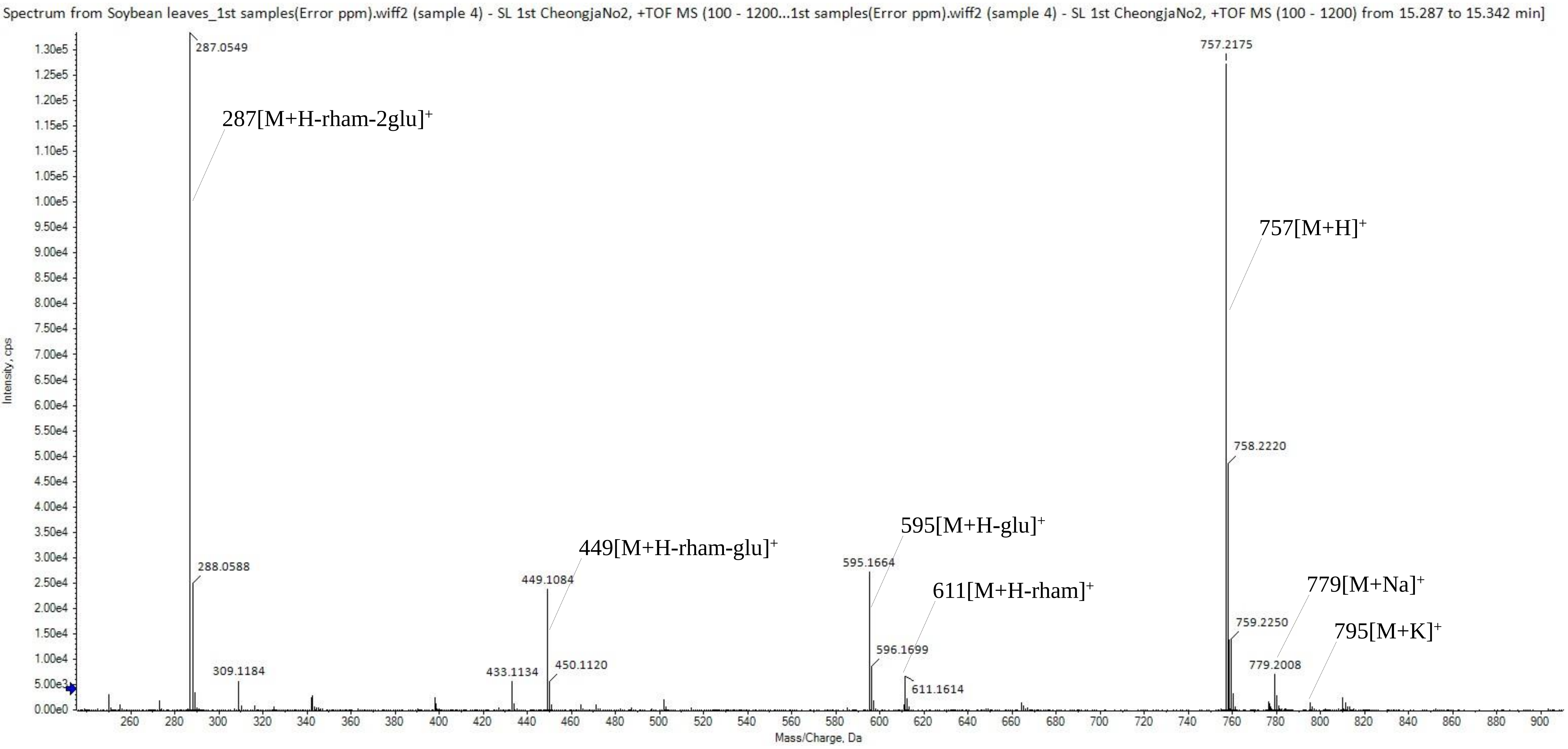

Peak **22**_I 3-*O*-(2-*O*-glu-6-*O*-rham)gal (soyanin I) (SL3)

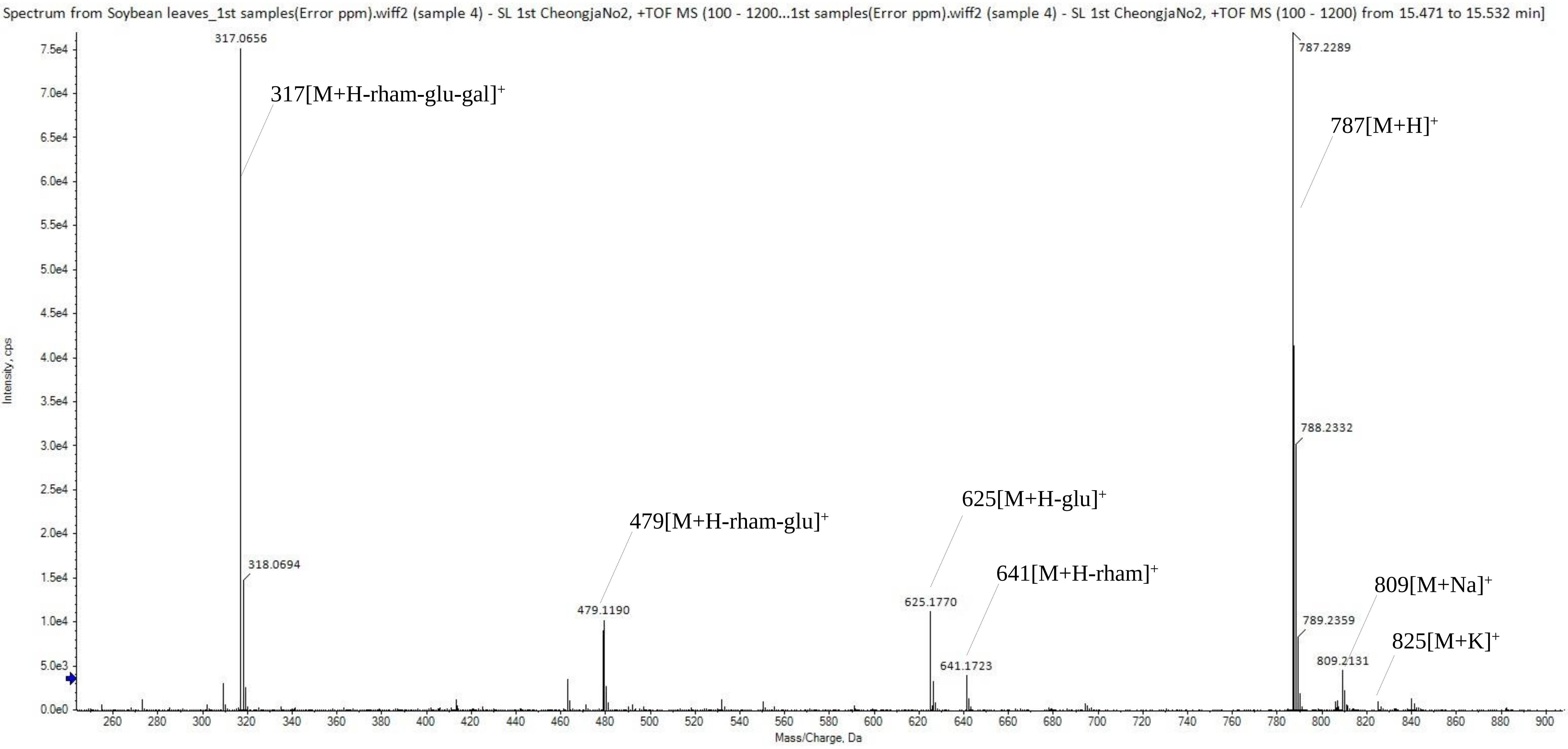

Peak **23**_I 3-*O*-(2-O-glu-6-O-rham)glu (soyanin II) (SL3)

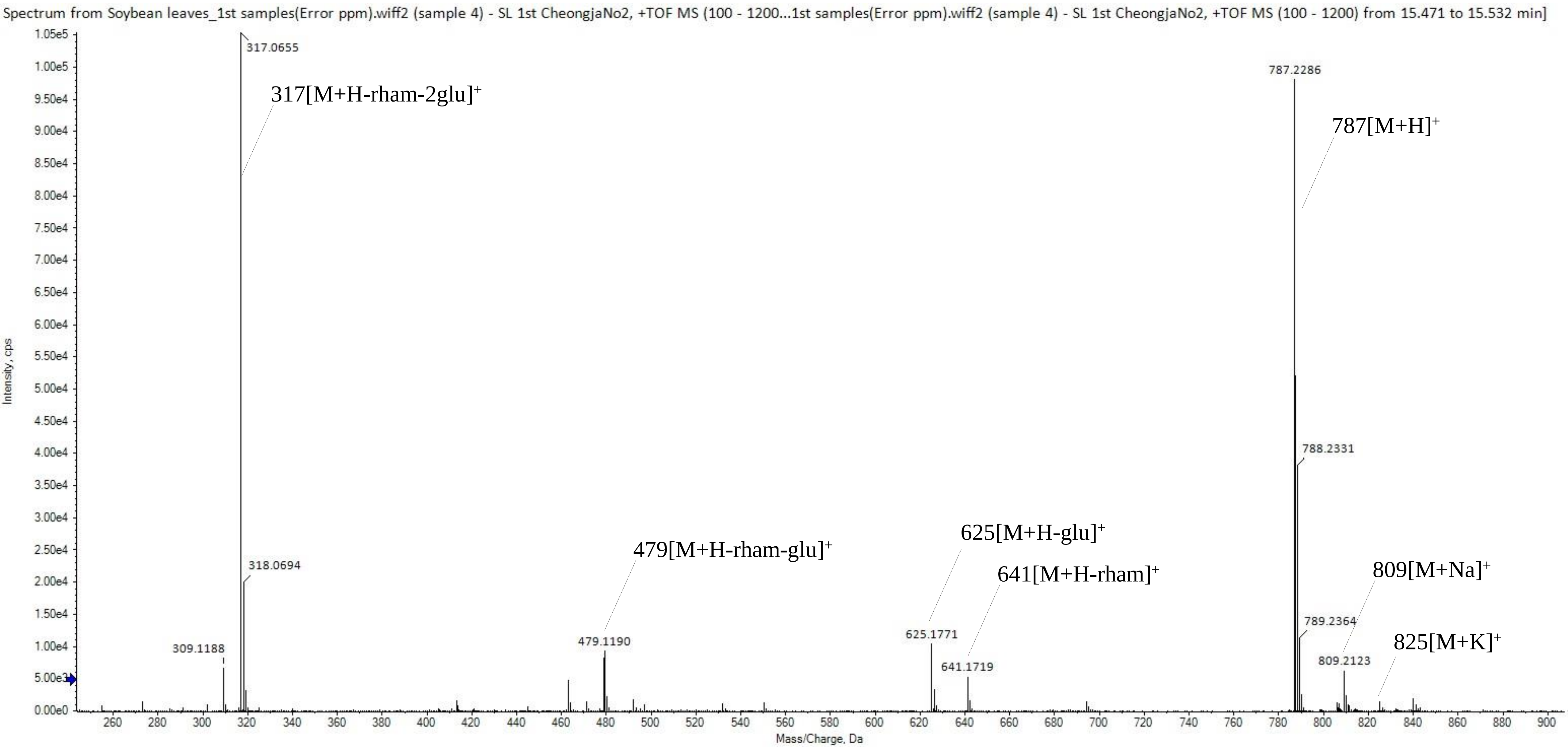

Peak **24**_Q 3-*O*-(2-*O*-rham)gal (SL10)

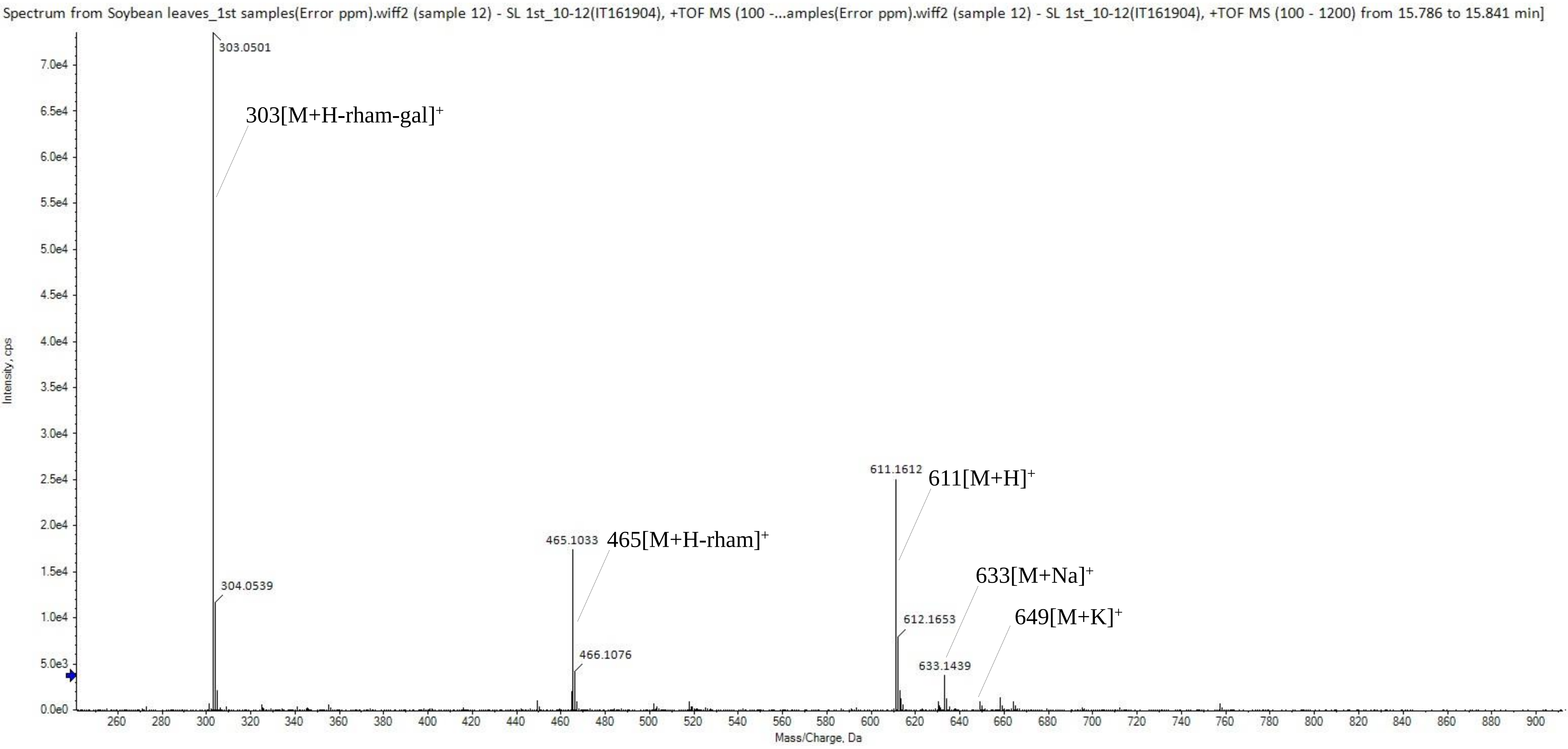

Peak **25**_Q 3-*O*-(2-*O*-rham)glu (Q 3-*O*-neo) (SL18)

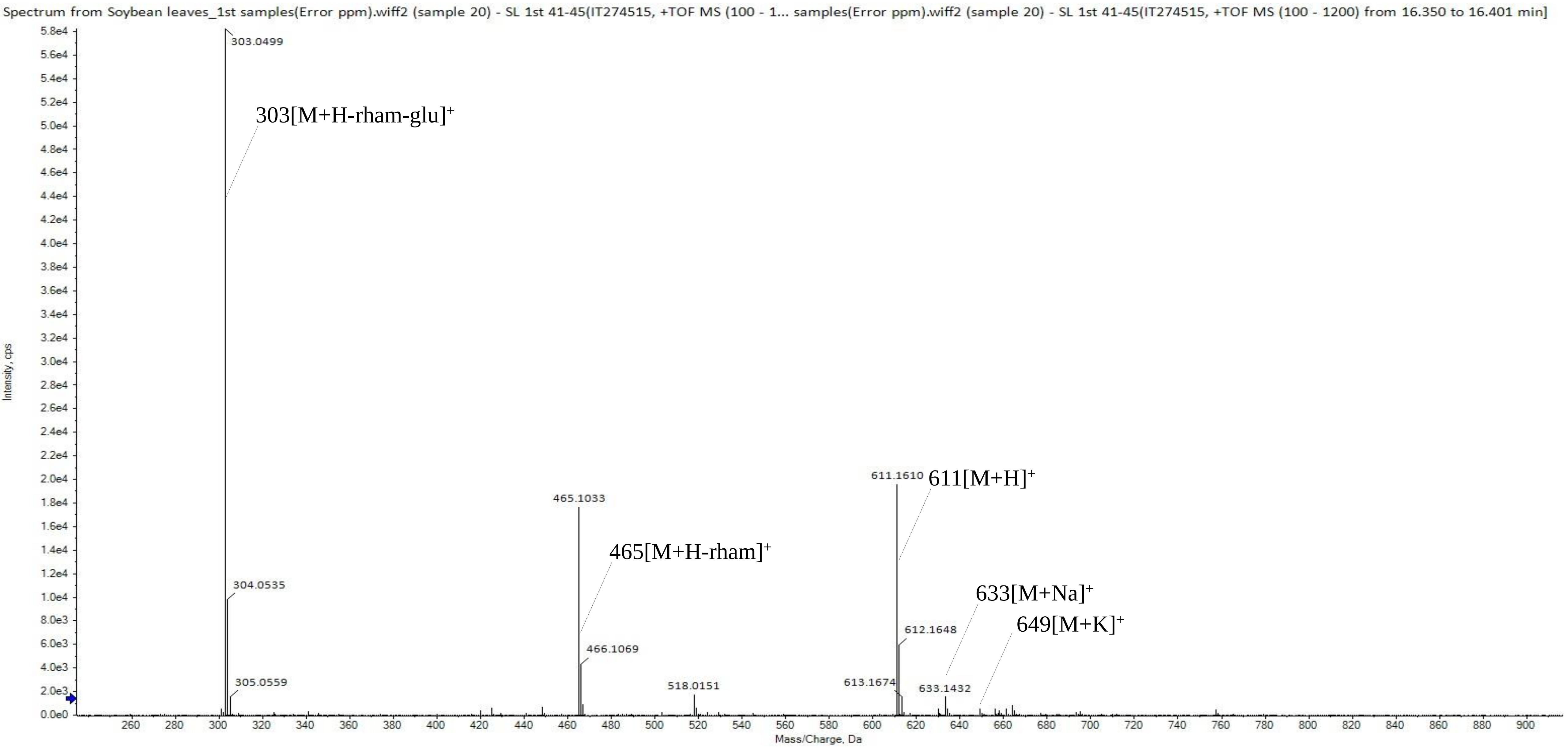

Peak **26**_K 3-*O*-(2-*O*-glu)gal (SL3)

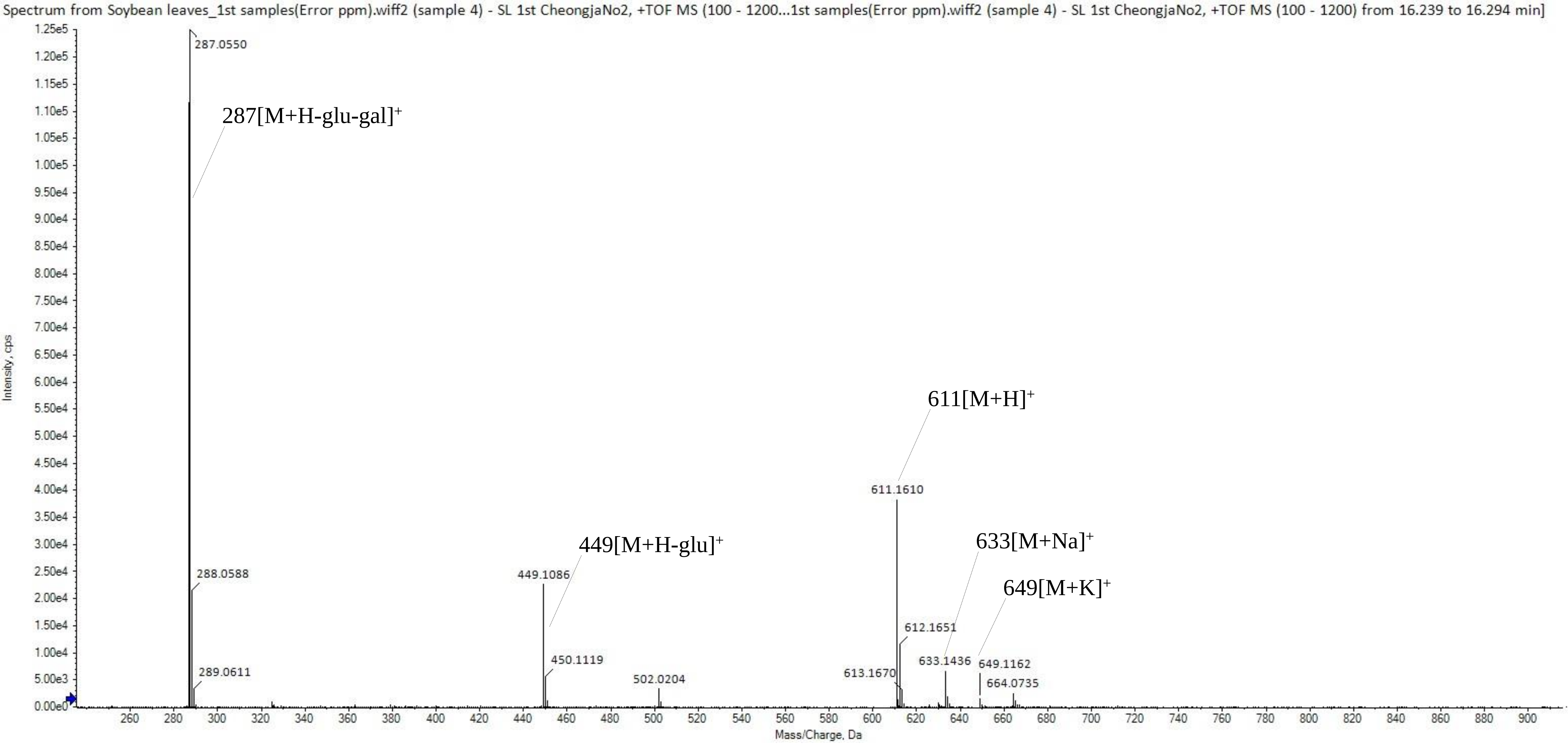

Peak **27**_K 3-*O*-(2,6-di-*O*-rham)gal (SL4)

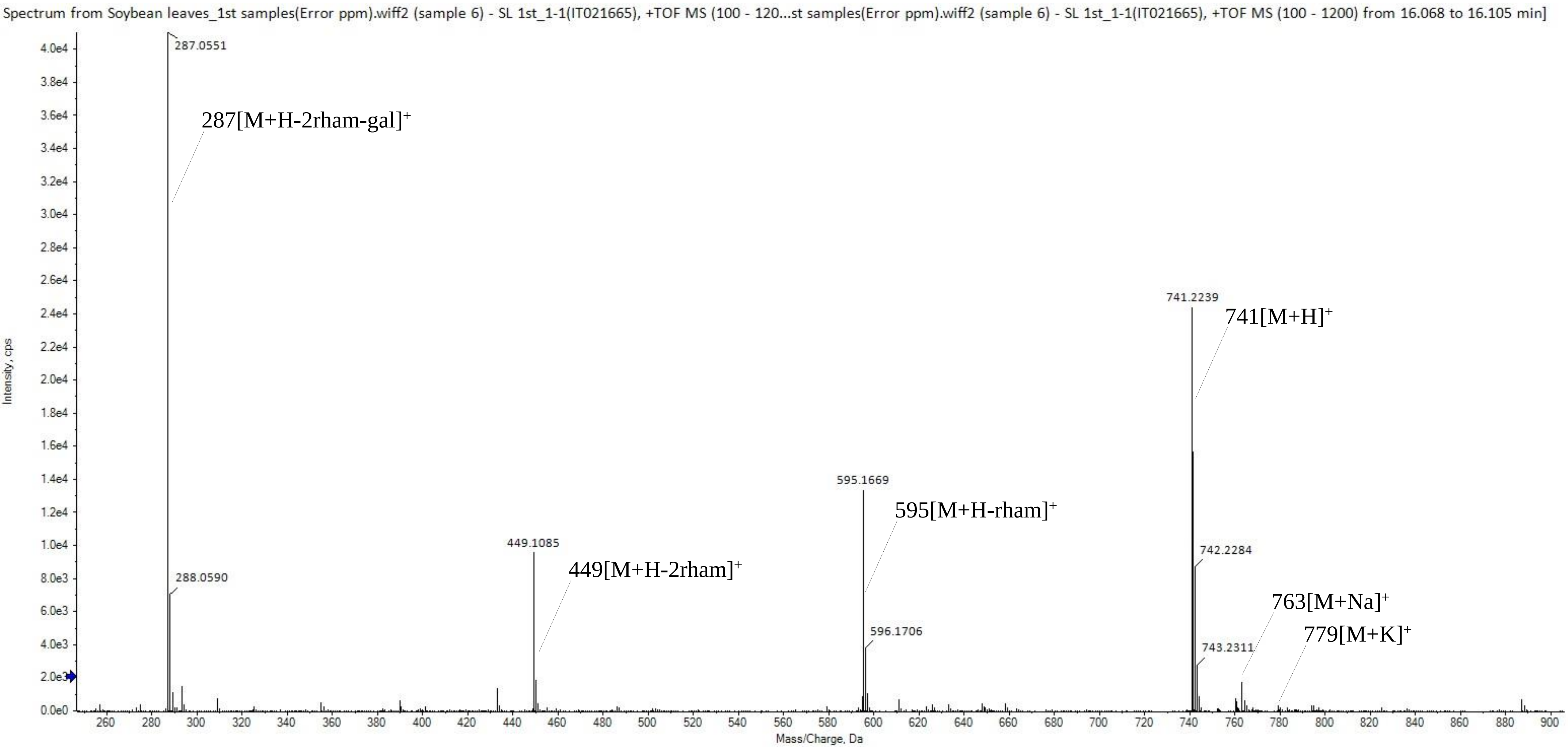

Peak **28**_K 3-*O*-(2-*O*-glu)glu (K 3-*O*-sop) (SL3)

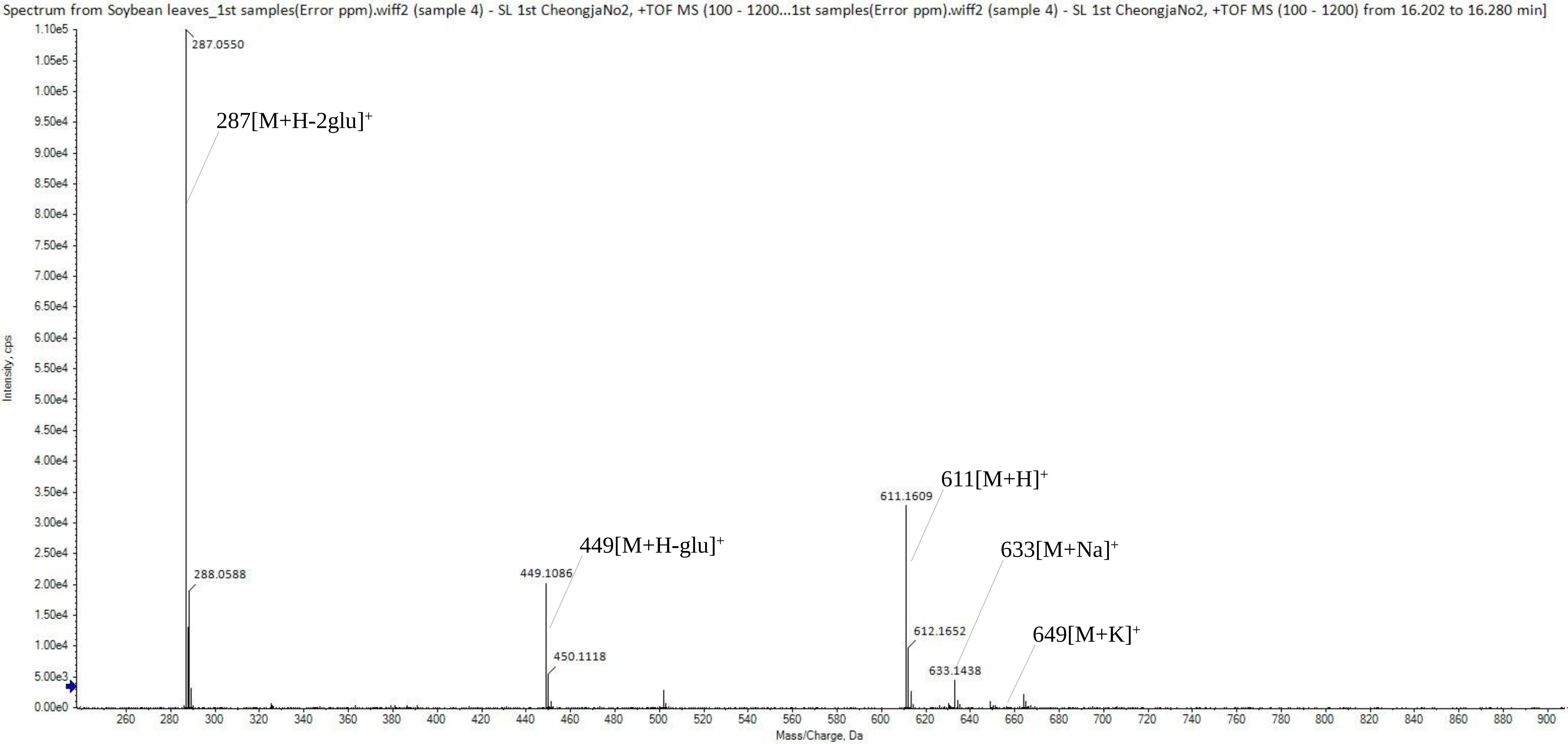

Peak **29**_K 3-*O*-(4,6-di-*O*-rham)gal (SL5)

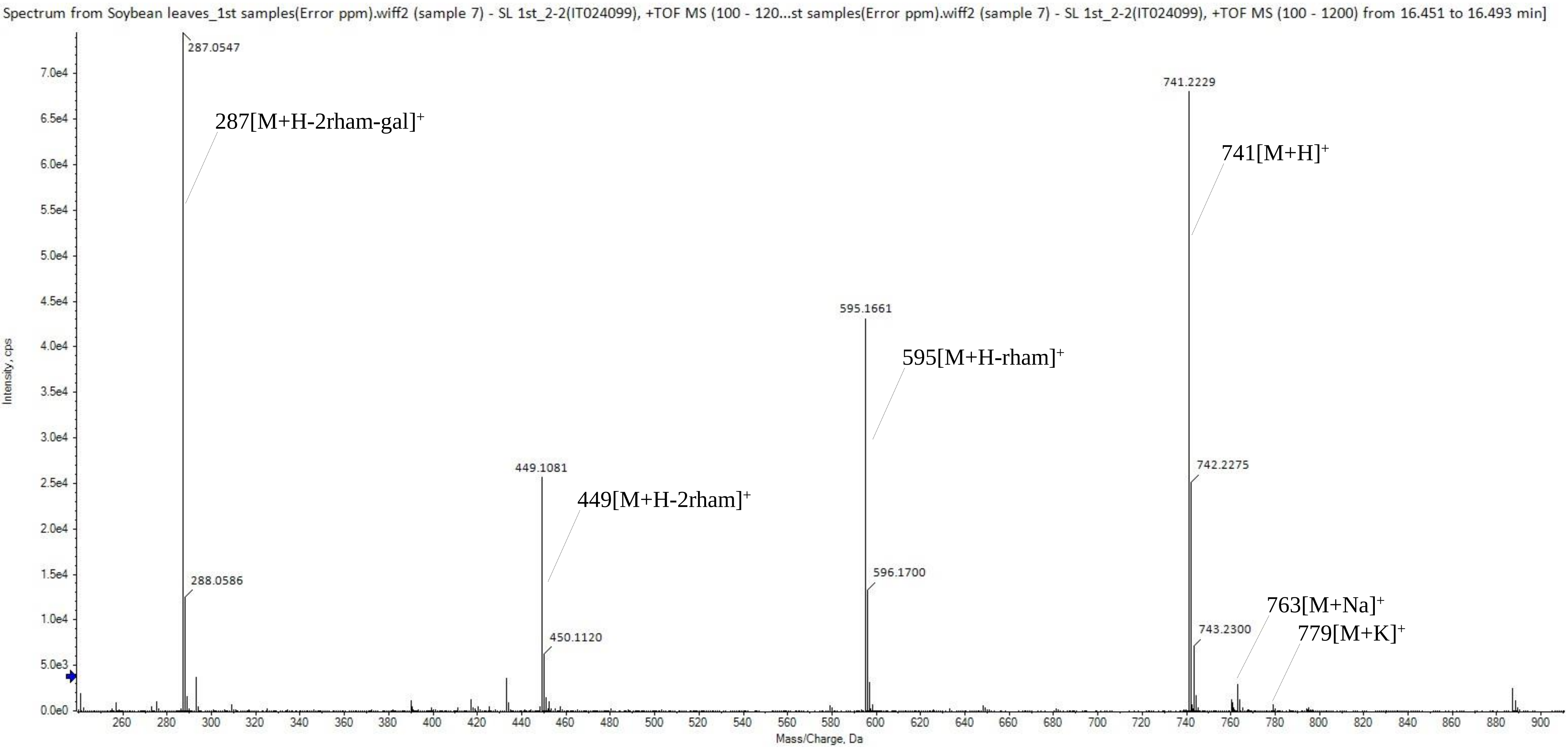

Peak **30**_K 3-*O*-(6-*O*-glu)gal (SL4)

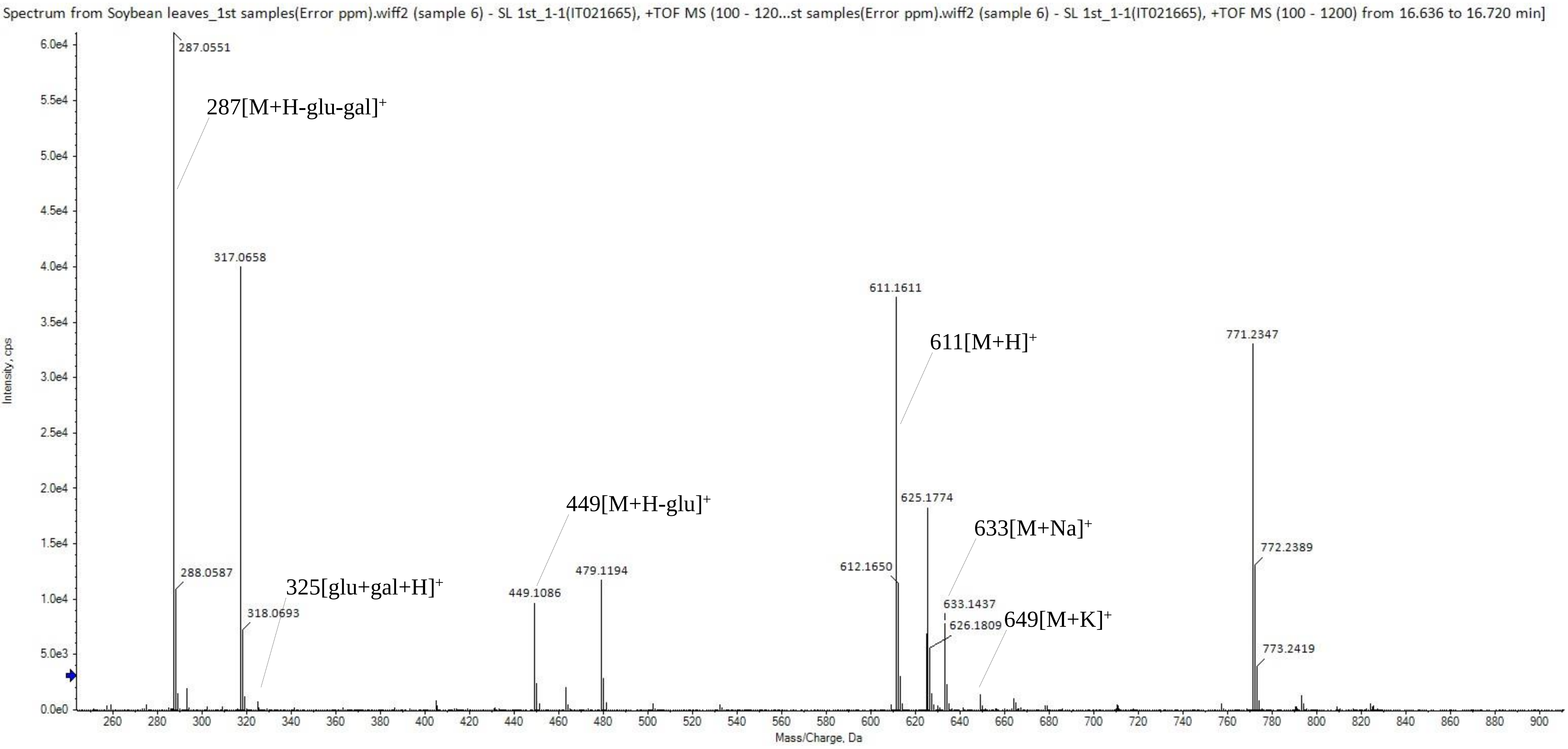

Peak **31**_I 3-*O*-(2-*O*-glu)gal (SL10)

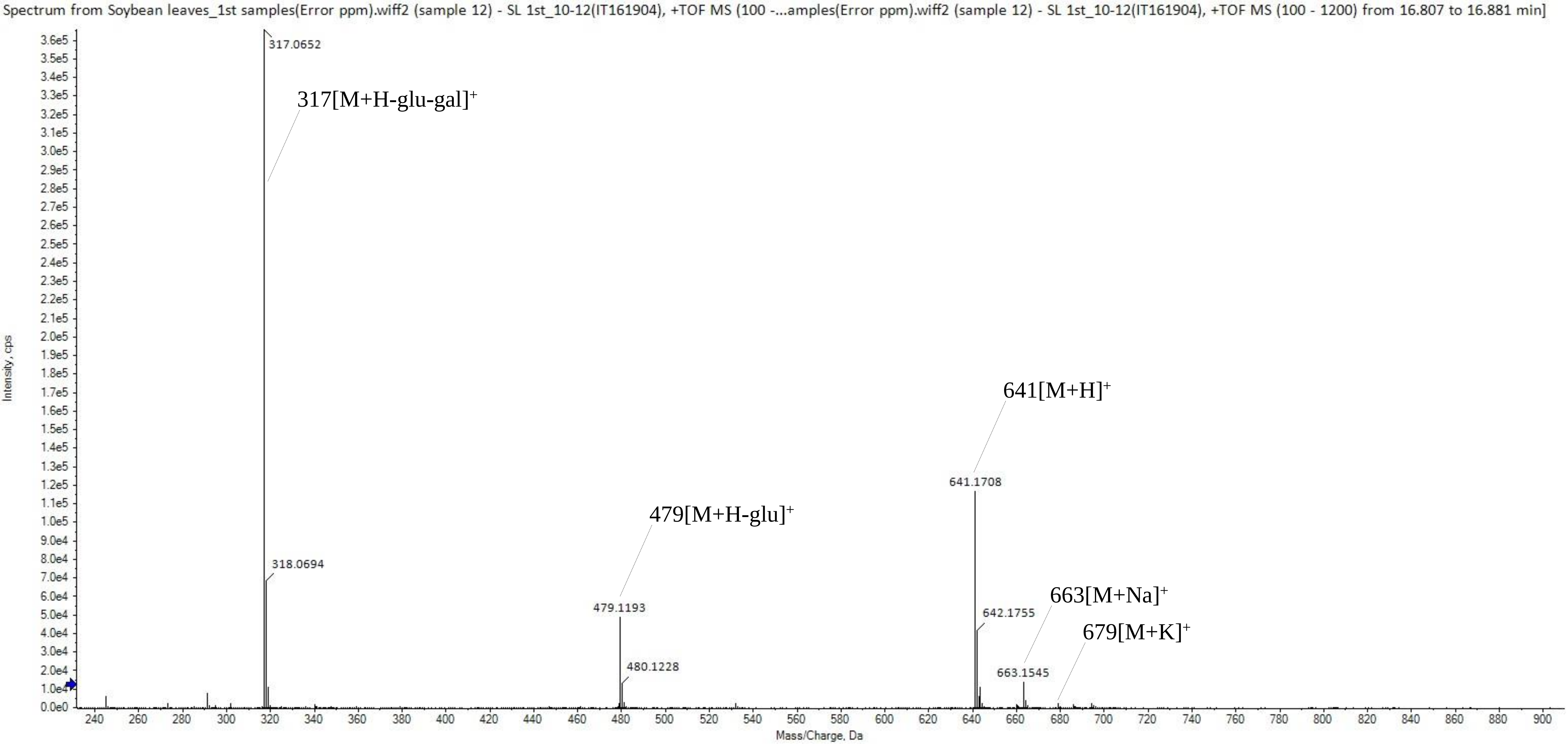

Peak **32**_I 3-*O*-(2-*O*-glu)glu (I 3-*O*-sop) (SL10)

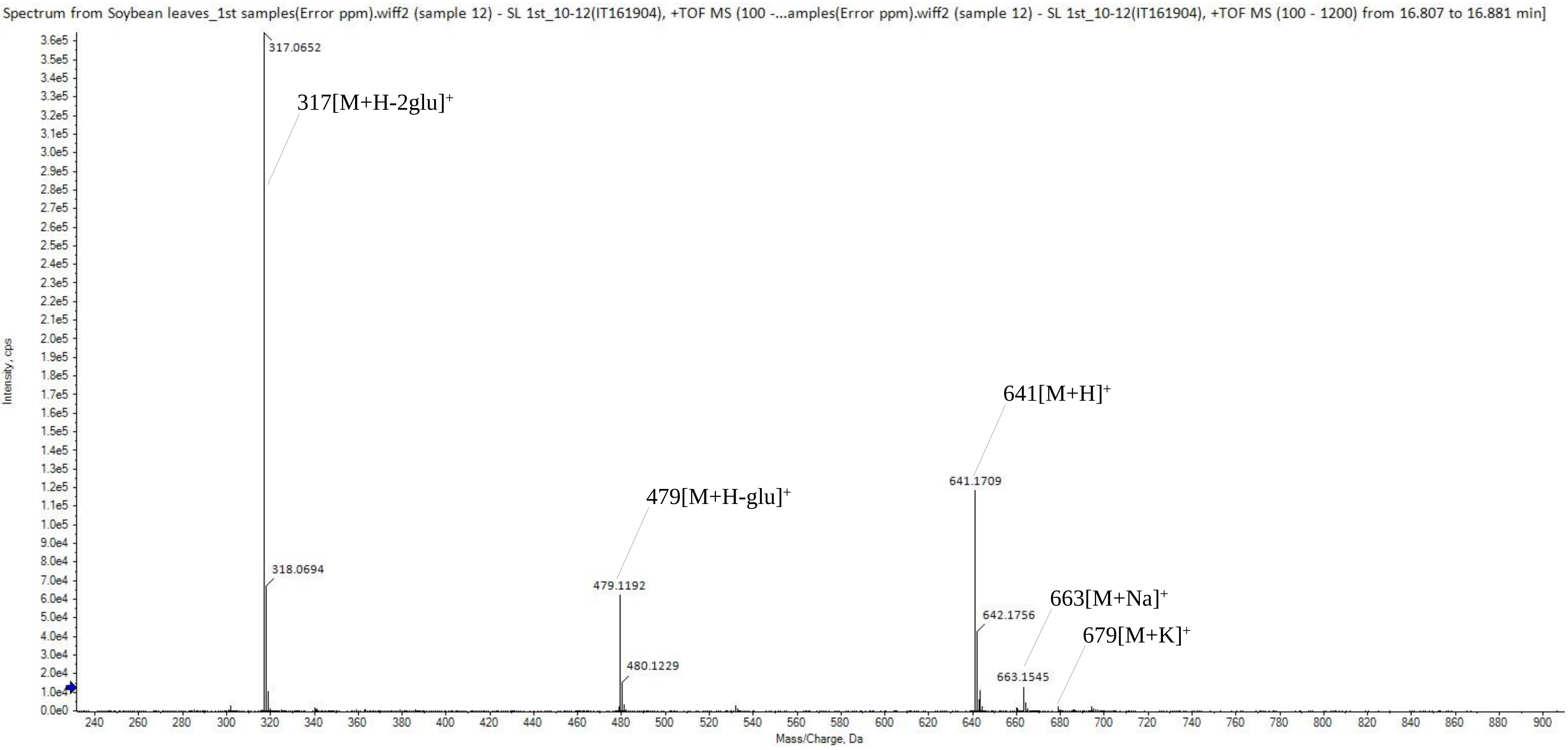

Peak **33**_I 3-*O*-(2,6-di-*O*-rham)gal (soyanin III) (SL4)

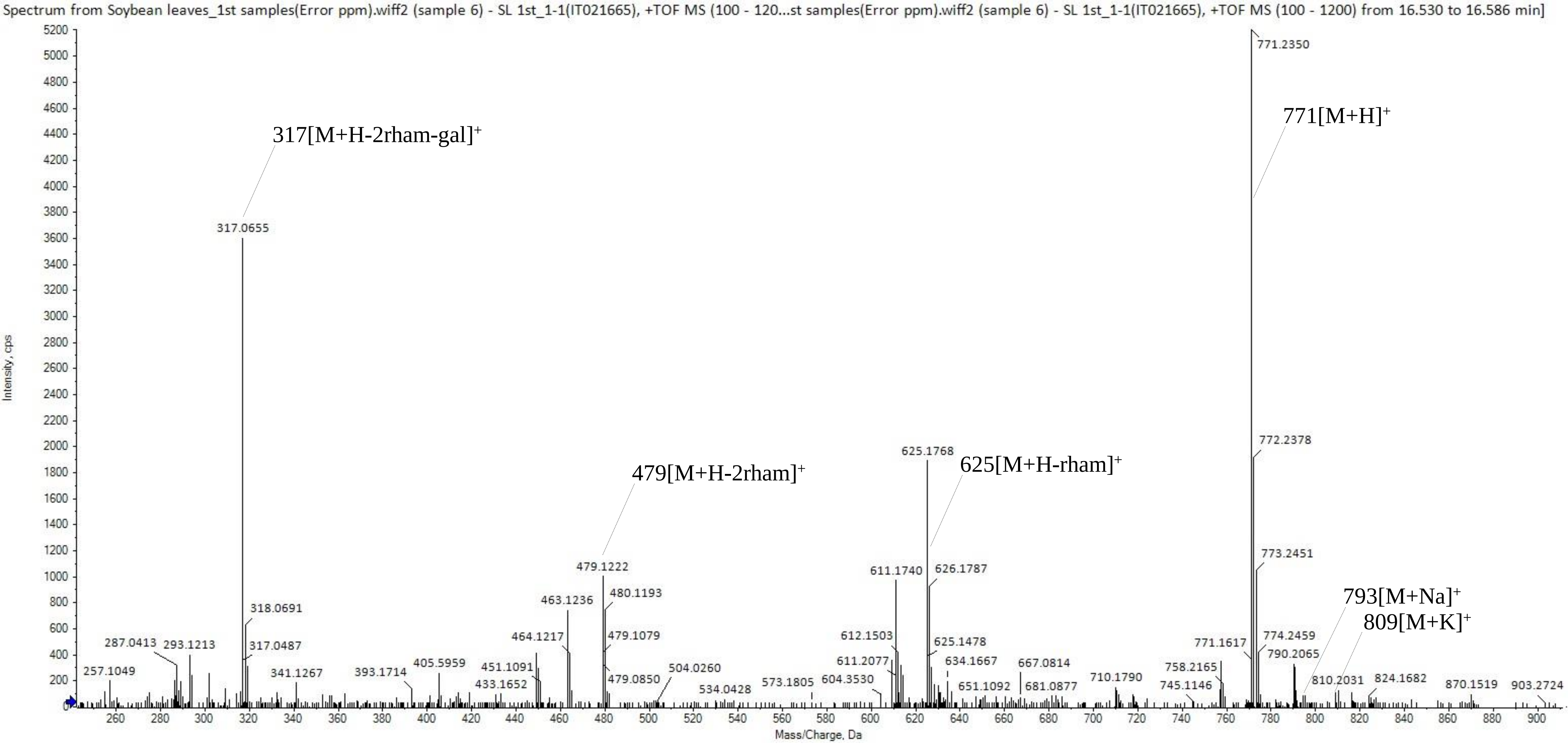

Peak **34**_I 3-*O*-(4,6-di-*O*-rham)gal (soyanin IV) (SL4)

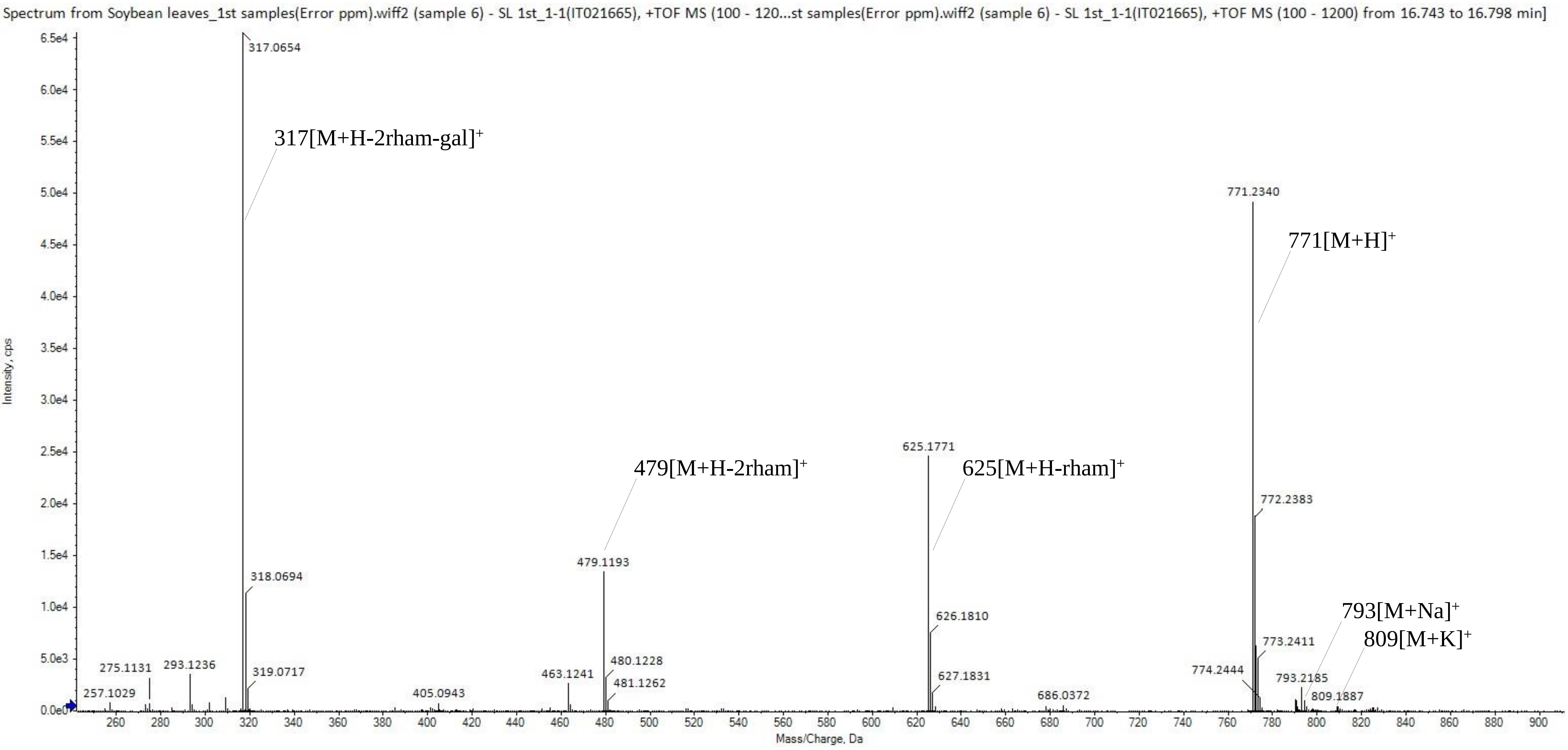

Peak **35**_Q 3-*O*-(6-*O*-rham)gal (Q 3-*O*-rob) (SL4)

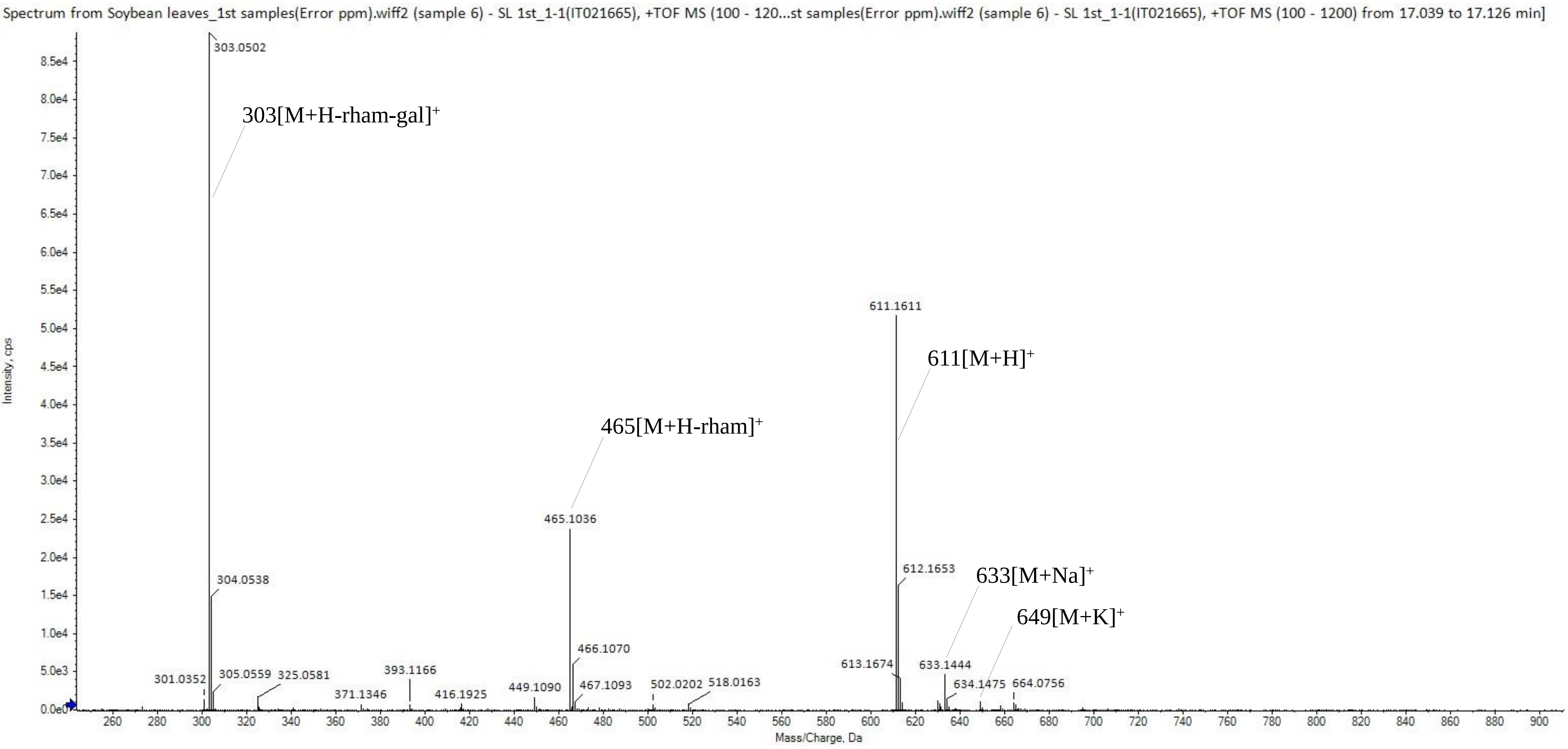

Peak **37**_K 3-*O*-(2-*O*-rham)gal (SL10)

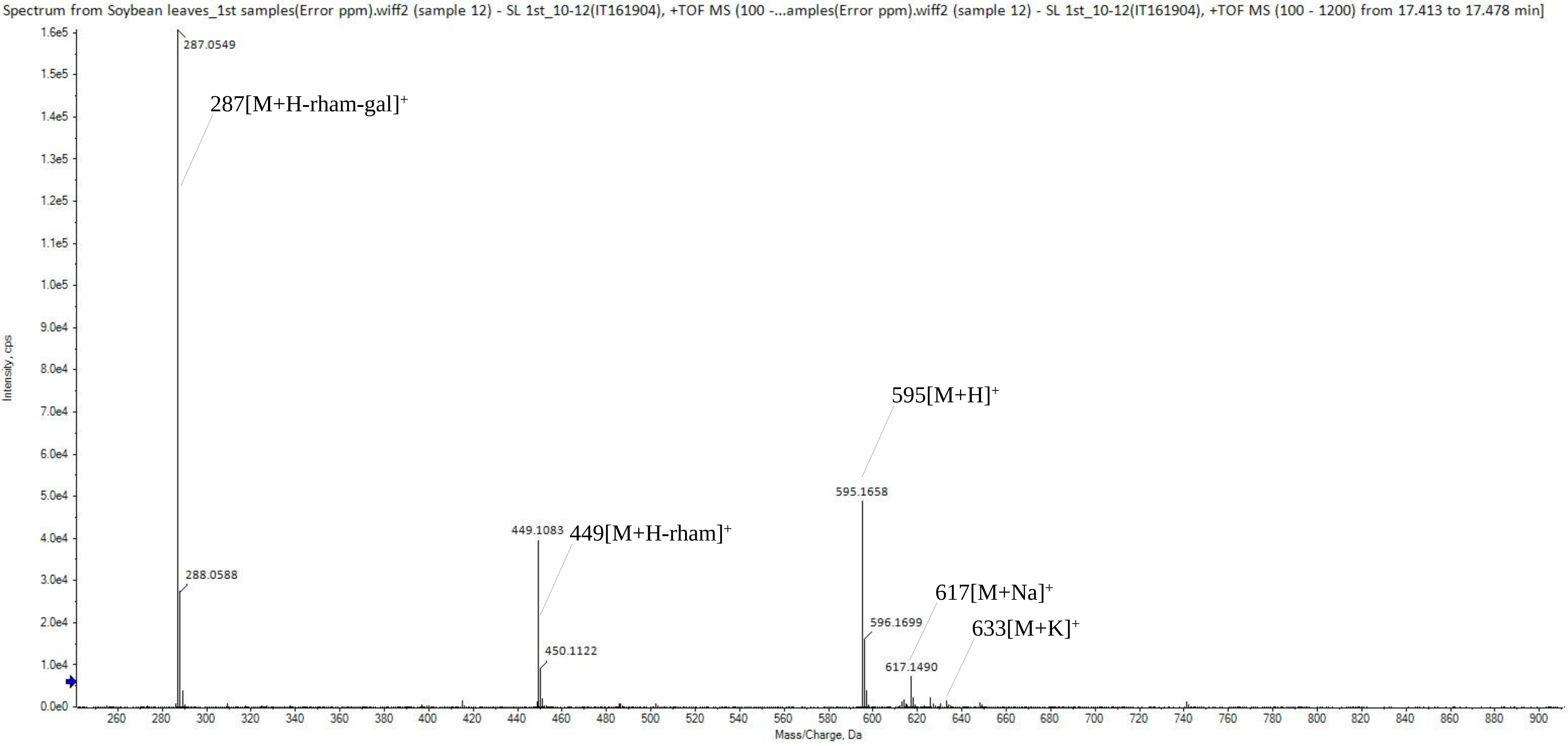

Peak **38**_Q 3-*O*-(6-*O*-rham)glu (Q 3-*O*-rut, rutin) (SL4)

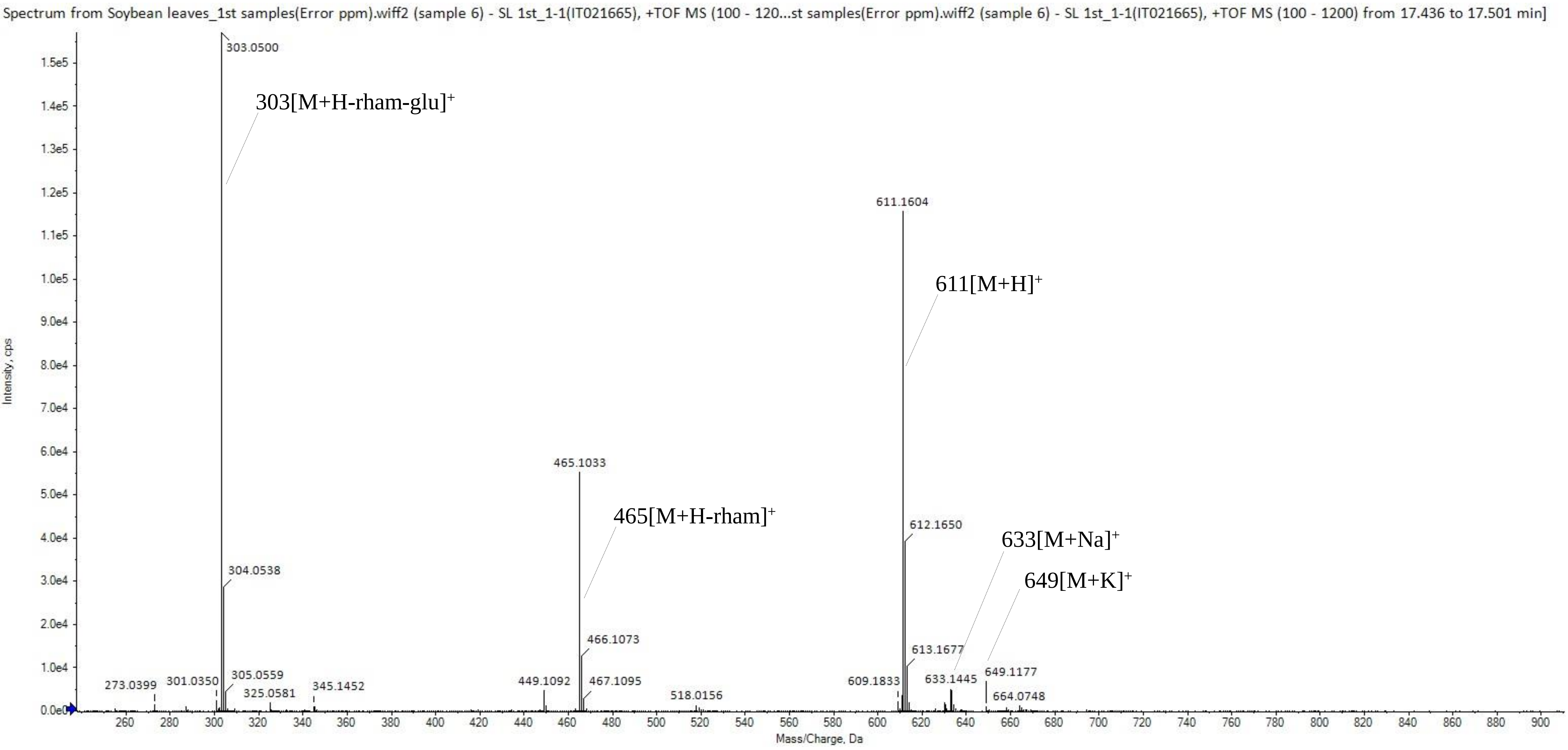

Peak **39**_K 3-*O*-(6-*O*-glu)glu (K 3-*O*-gen) (SL4)

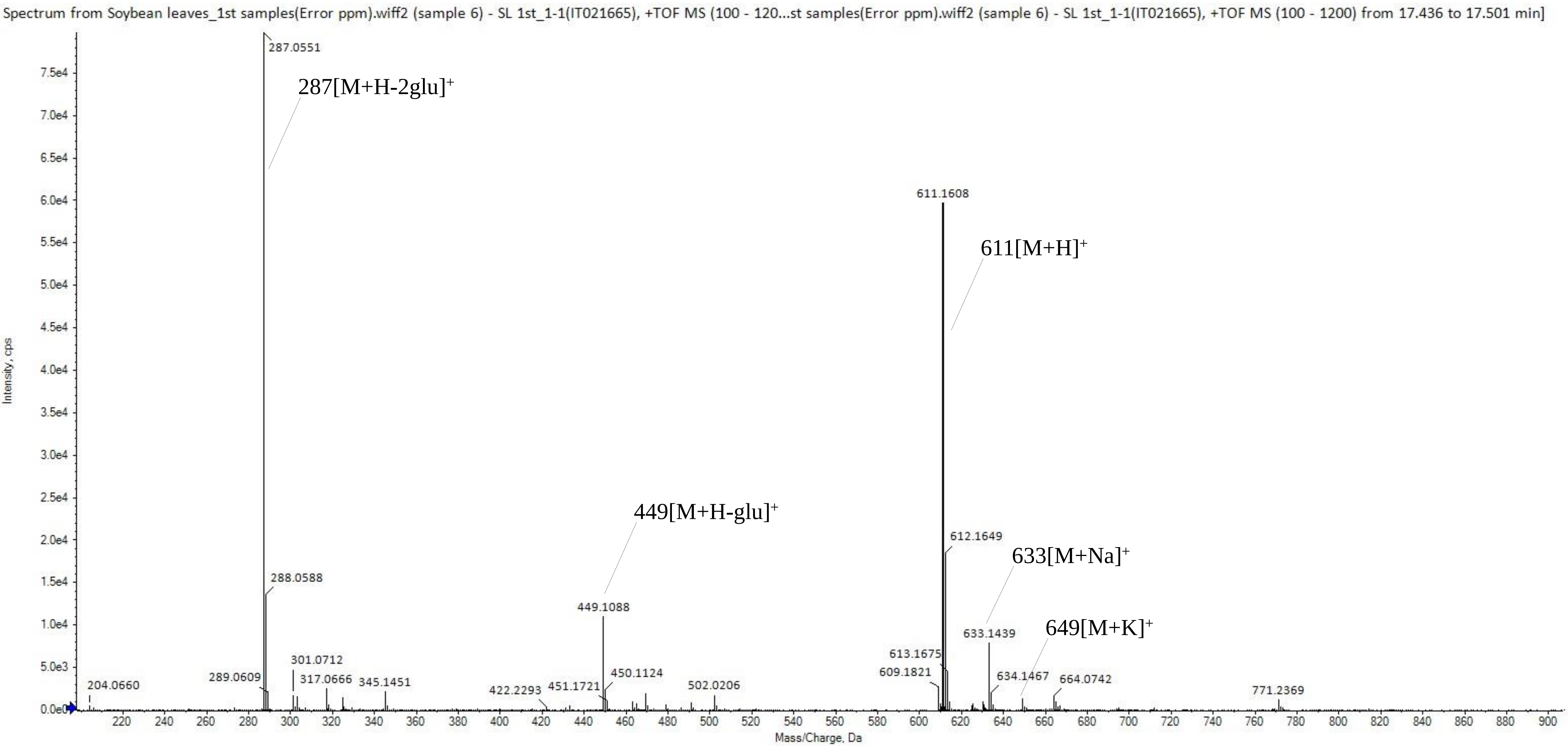

Peak **40**_Q 3-*O*-gal (hyperoside) (SL18)

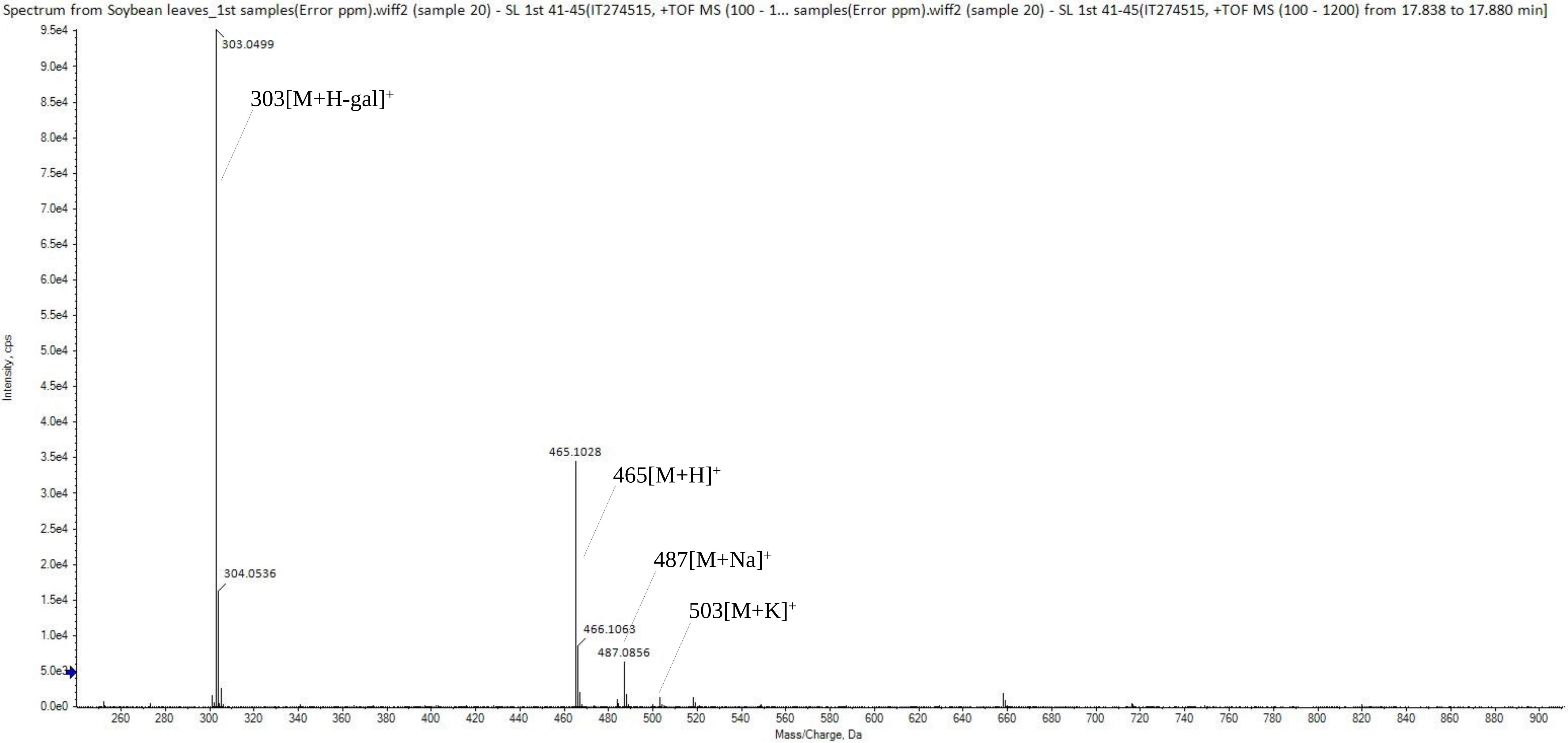

Peak **41**_K 3-*O*-(2-*O*-glu-6-*O*-rham)gal DME (SL9)

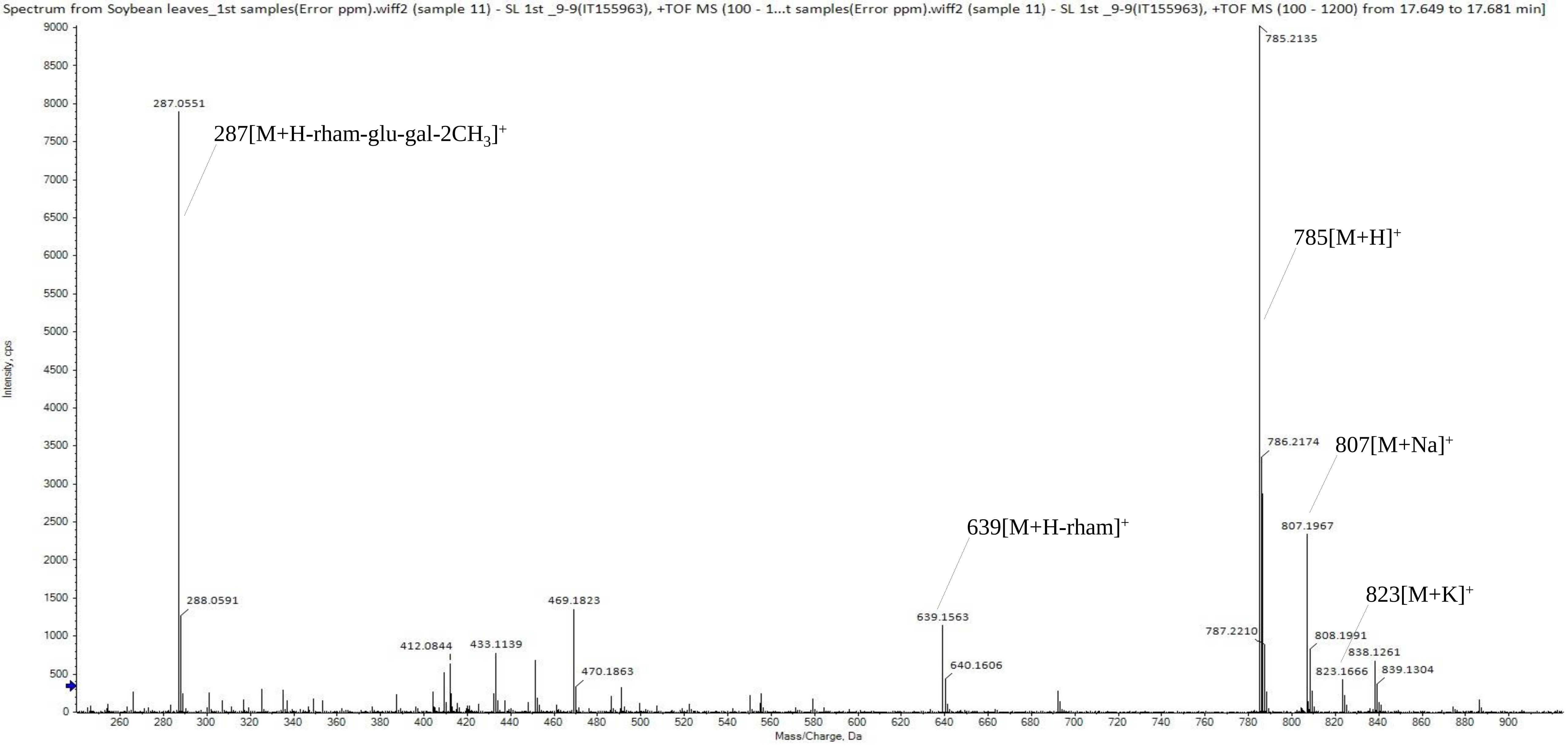

Peak **42**_I 3-*O*-(2,6-di-*O*-rham)glu (soyanin V) (SL4)

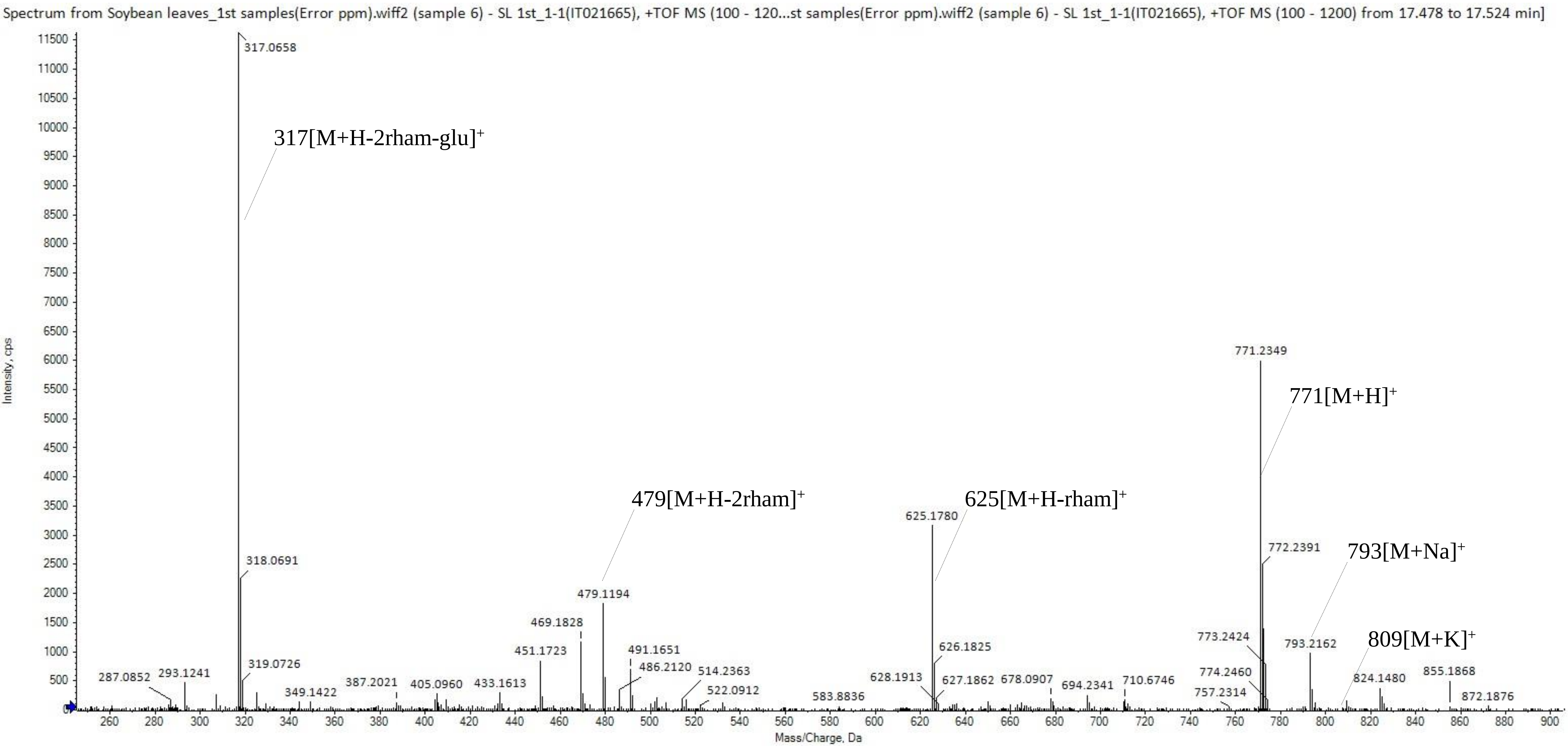

Peak **43**_K 3-*O*-(2-*O*-rham)glu (SL18)

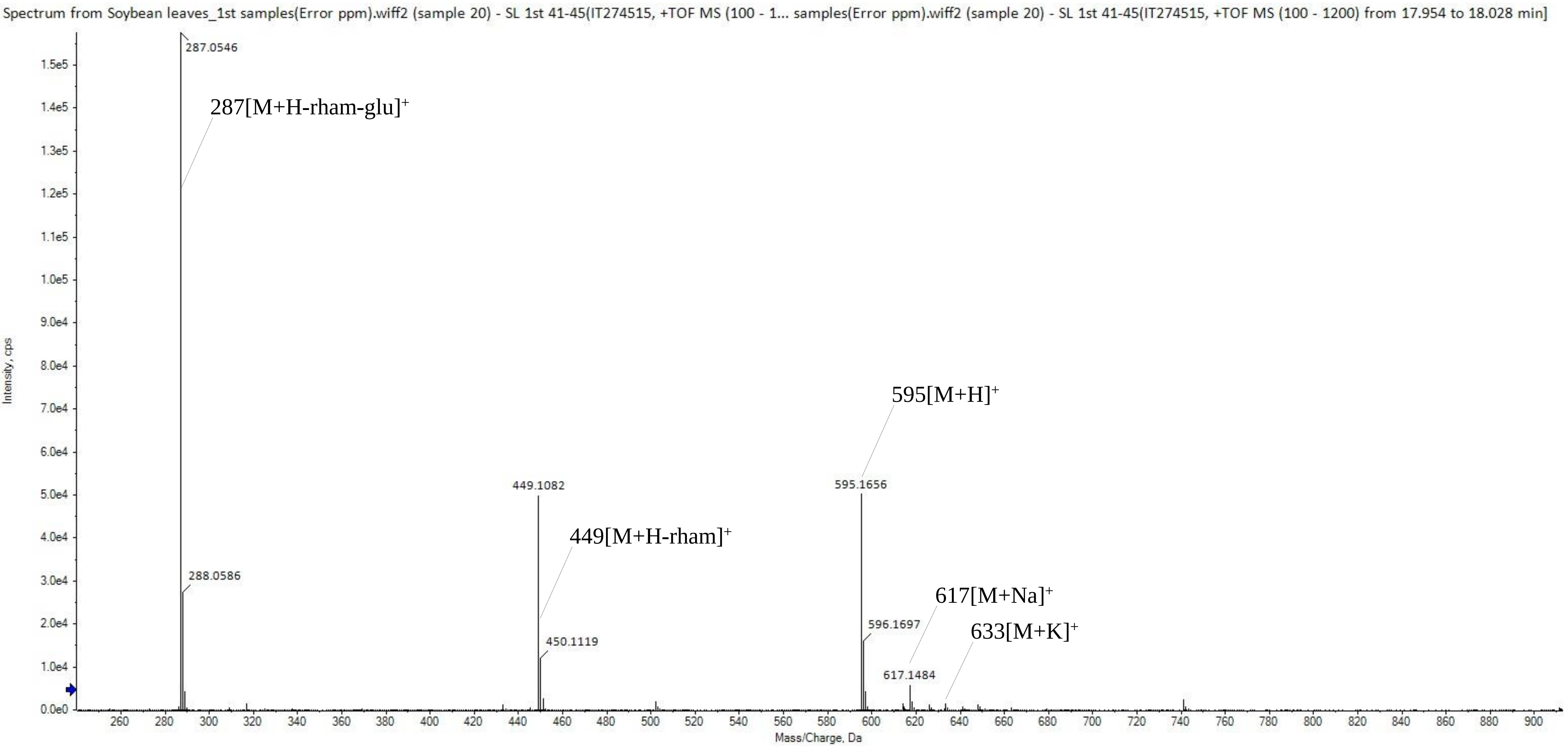

Peak **44**_I 3-*O*-(6-*O*-glu)gal (SL4)

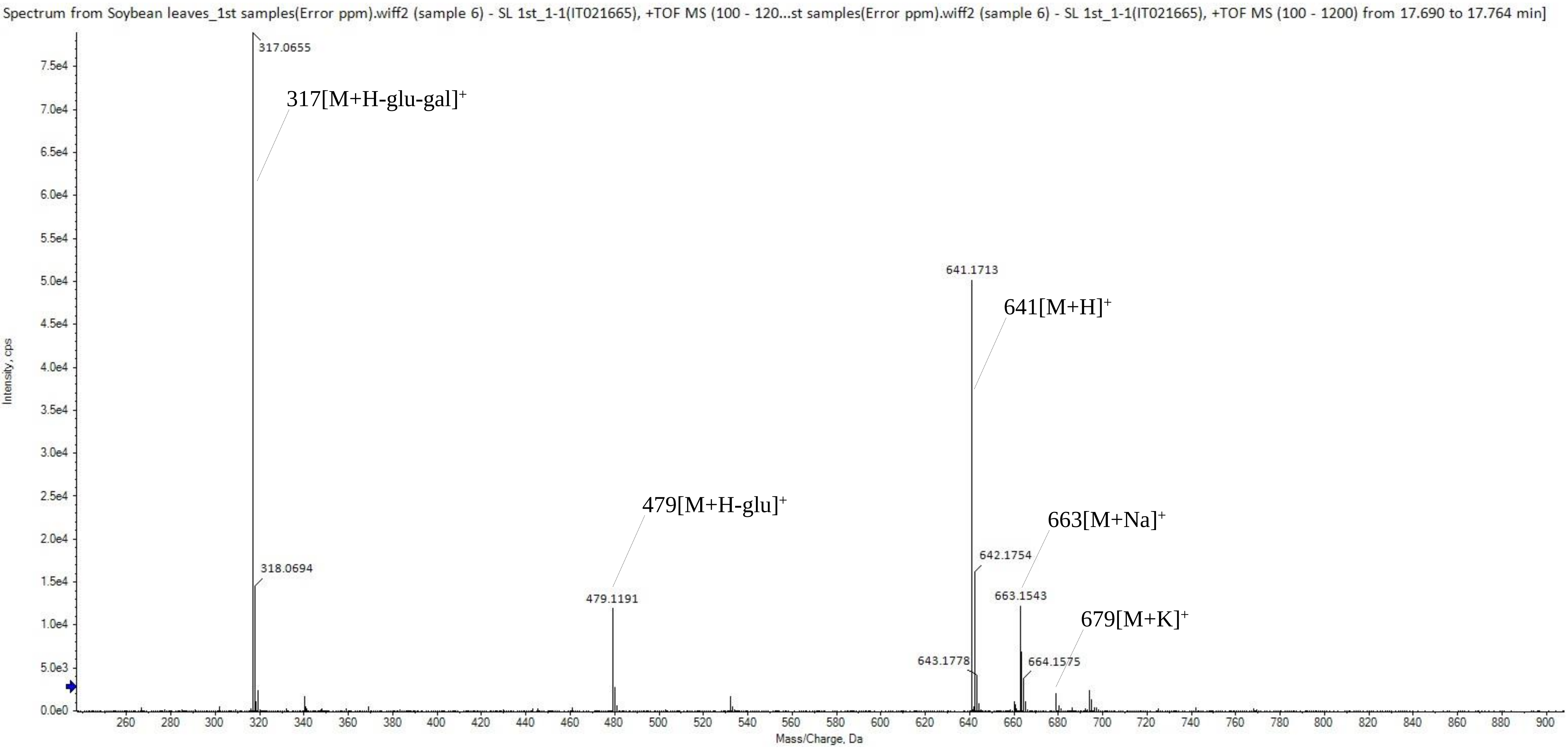

Peak **46**_Q 3-*O*-glu (isoquercitrin) (SL18)

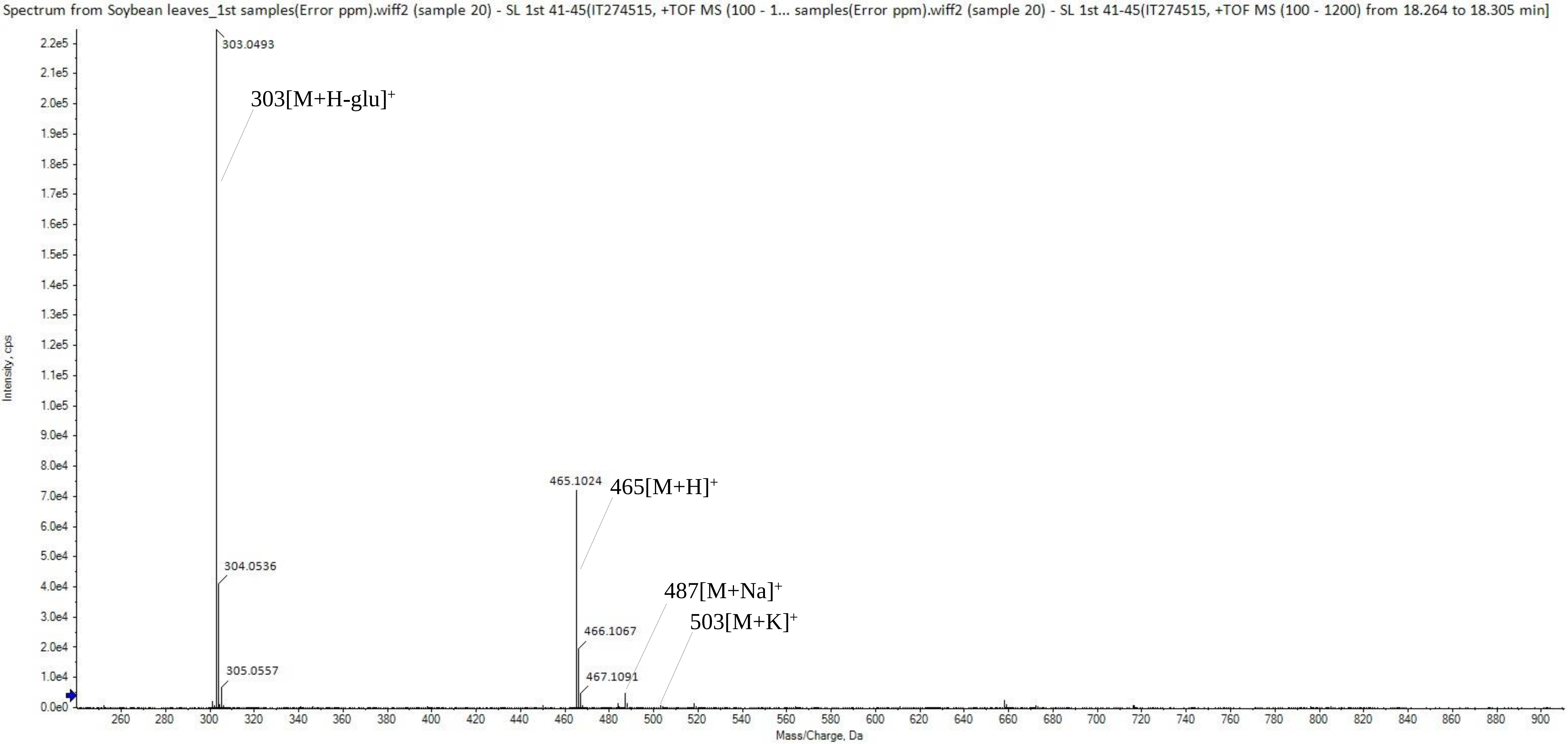

Peak **47**_I 3-*O*-(2-*O*-rham)gal (SL18)

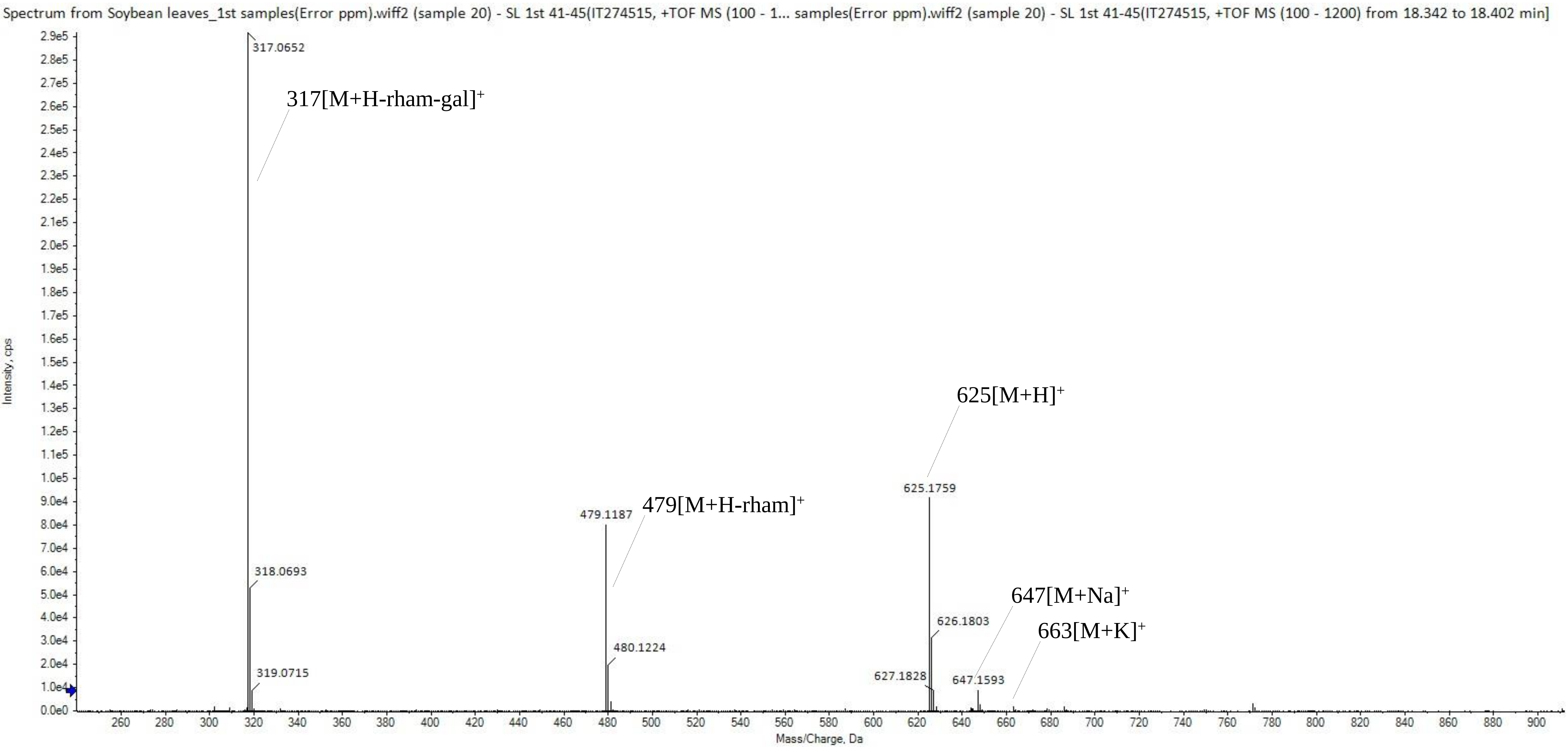

Peak **51**_I 3-*O*-(6-*O*-glu)glu (I 3-*O*-gen) (SL4)

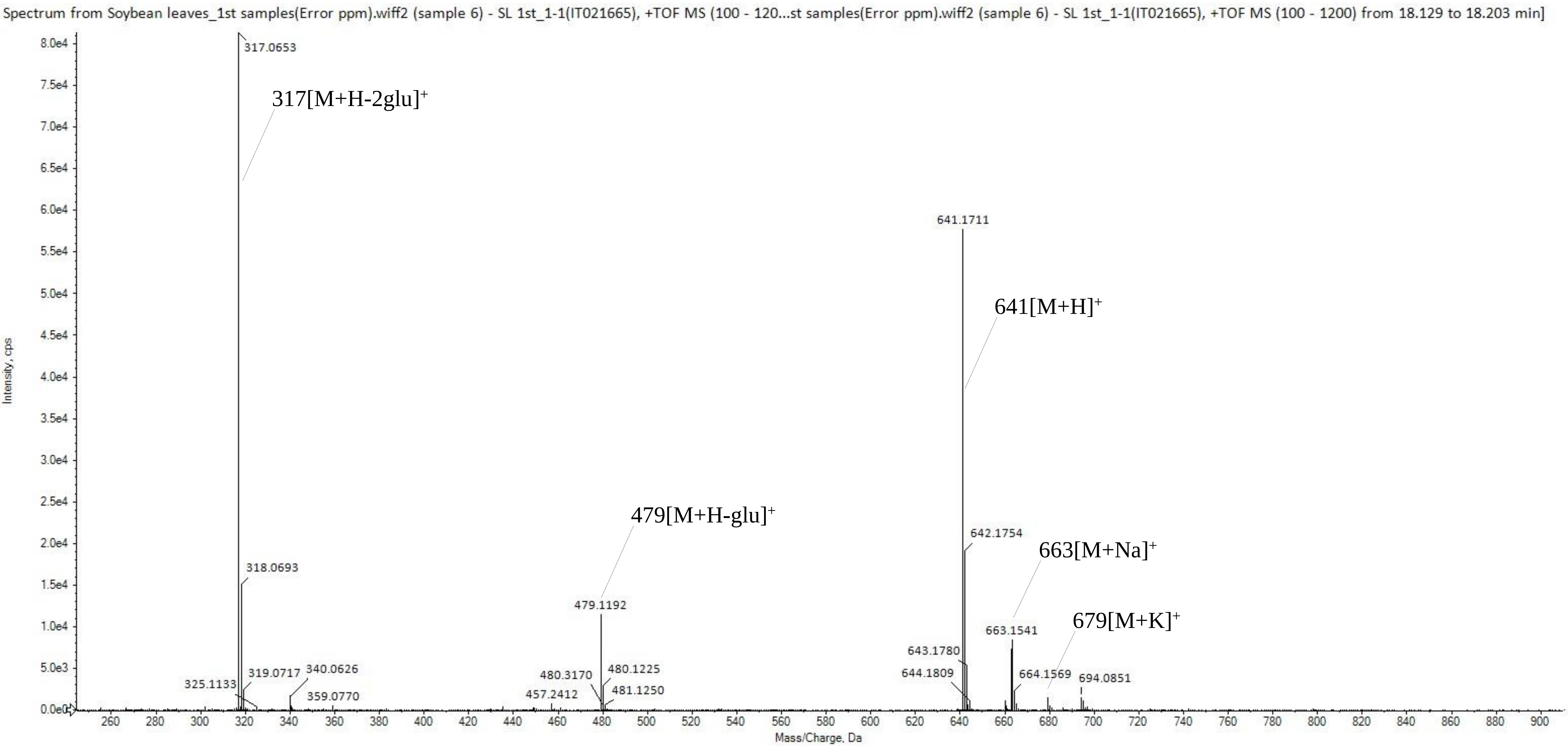

Peak **52**_K 3-*O*-(2-*O*-glu)gal DME (SL21)

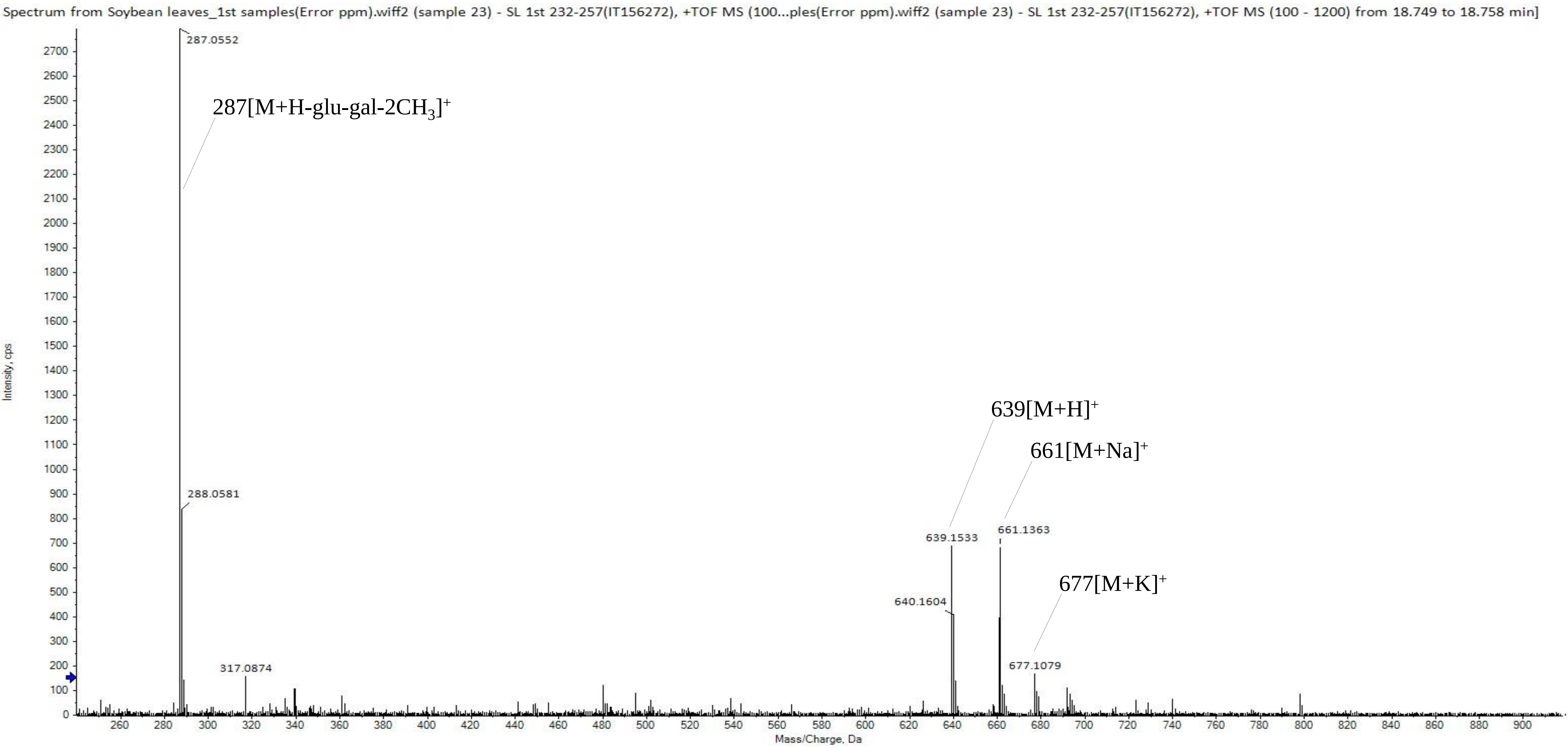

Peak **53**_K 3-*O*-(2-*O*-glu)glu DME (SL21)

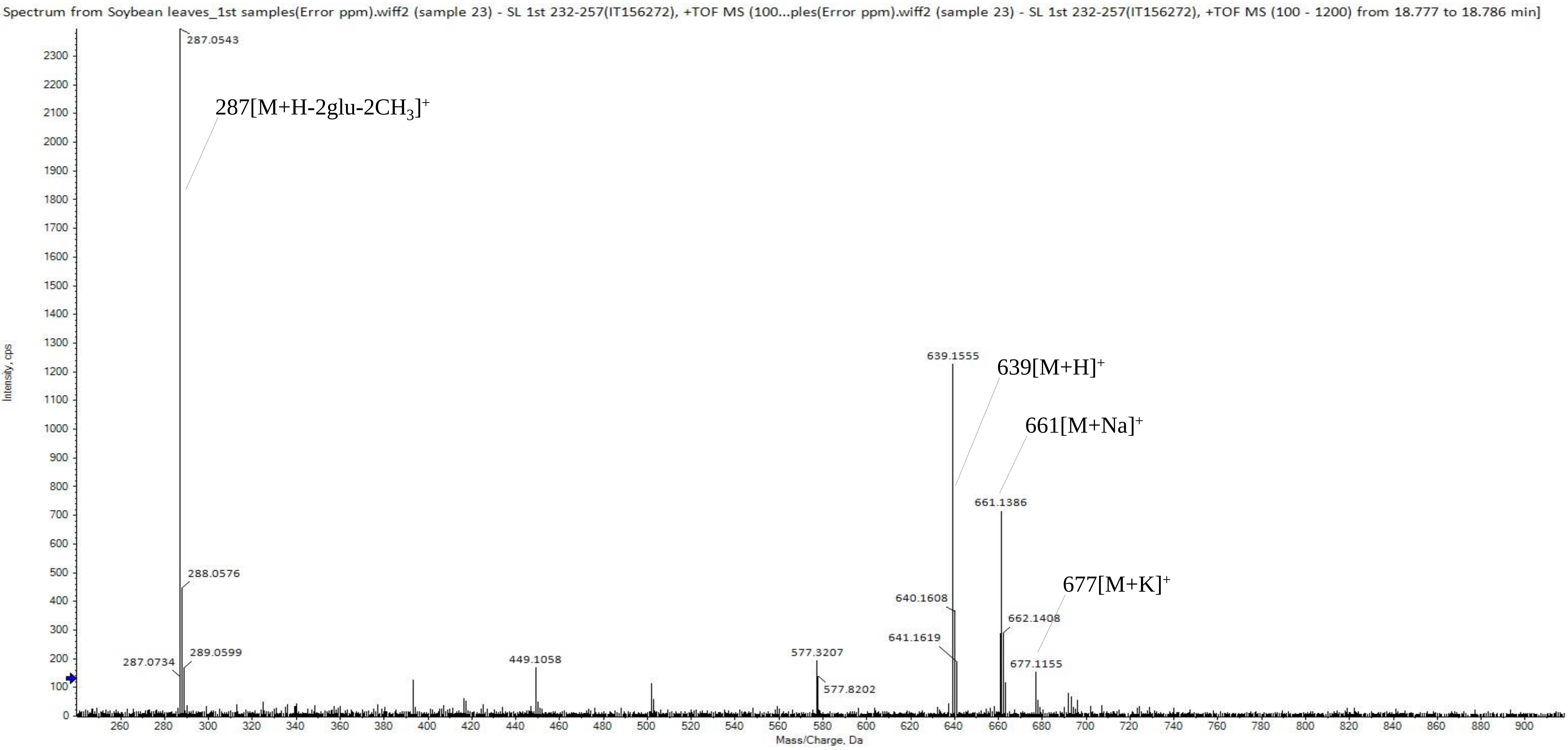

Peak **54**_K 3-*O*-(6-*O*-rham)gal (K 3-*O*-rob, biorobin) (SL4)

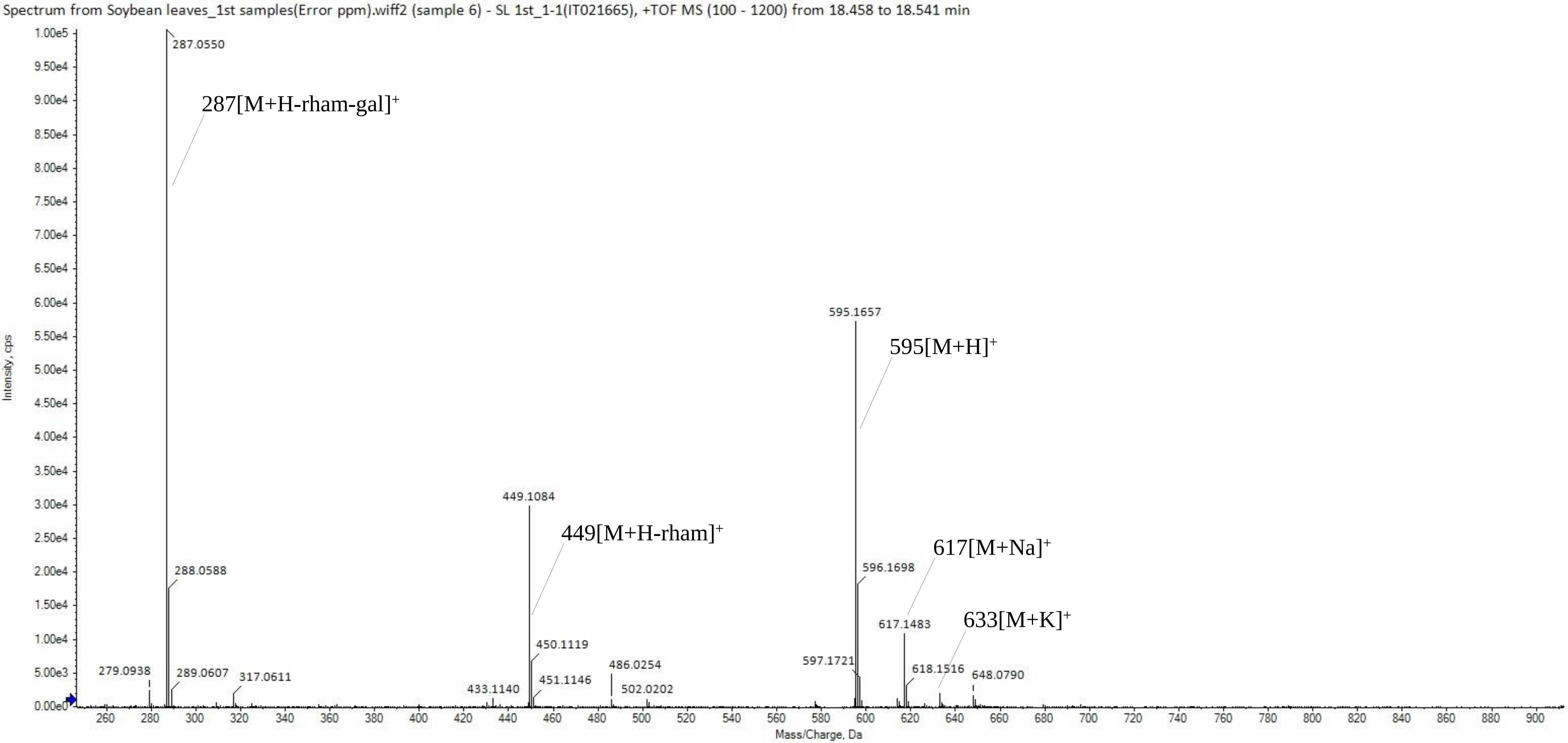

Peak **55**_I 3-*O*-(2-*O*-rham)glu (I 3-*O*-neo, calendoflavoside) (SL18)

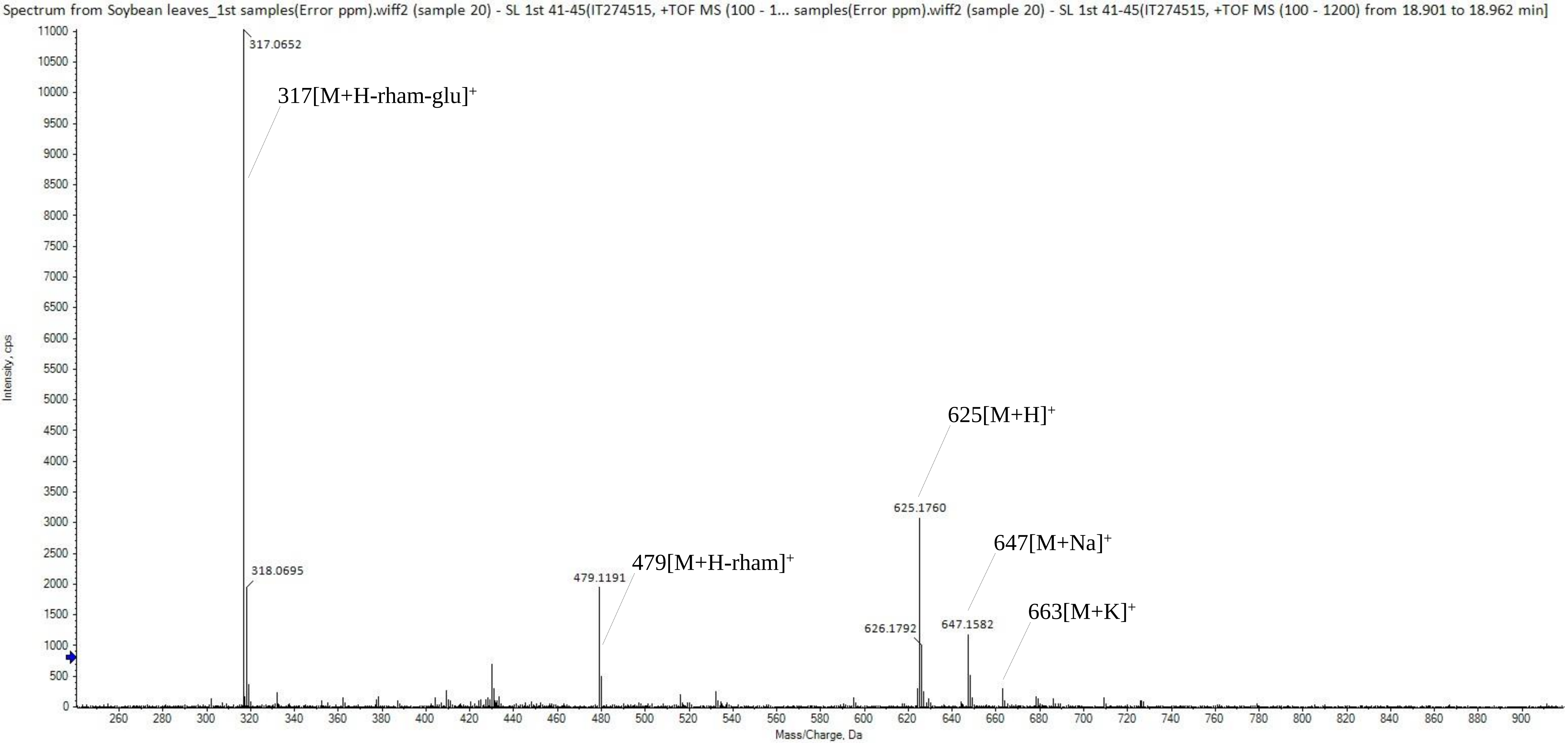

Peak **57**_K 3-*O*-gal (trifolin) (SL4)

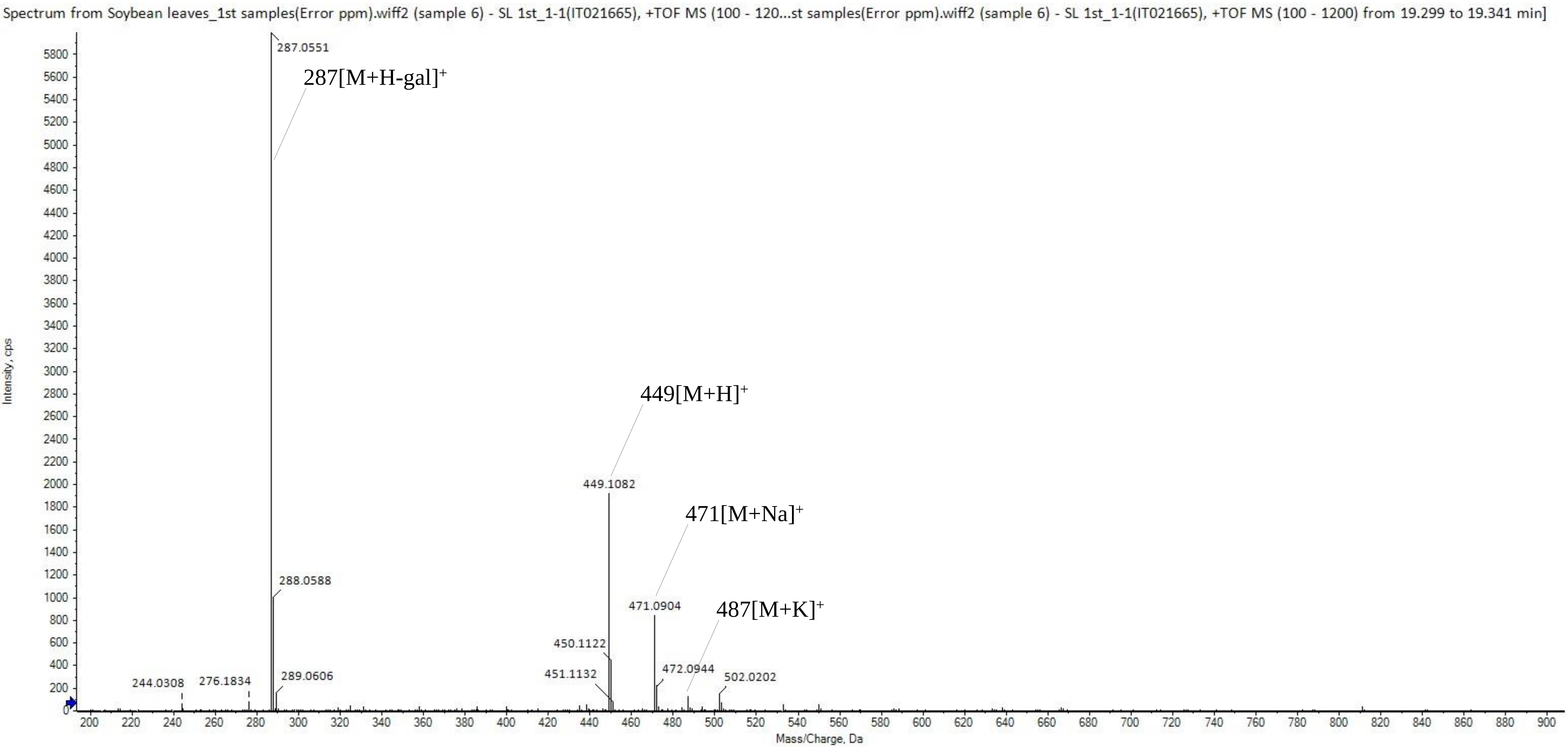

Peak **58**_K 3-*O*-(6-*O*-rham)glu (K 3-*O*-rut, nicotiflorin) (SL4)

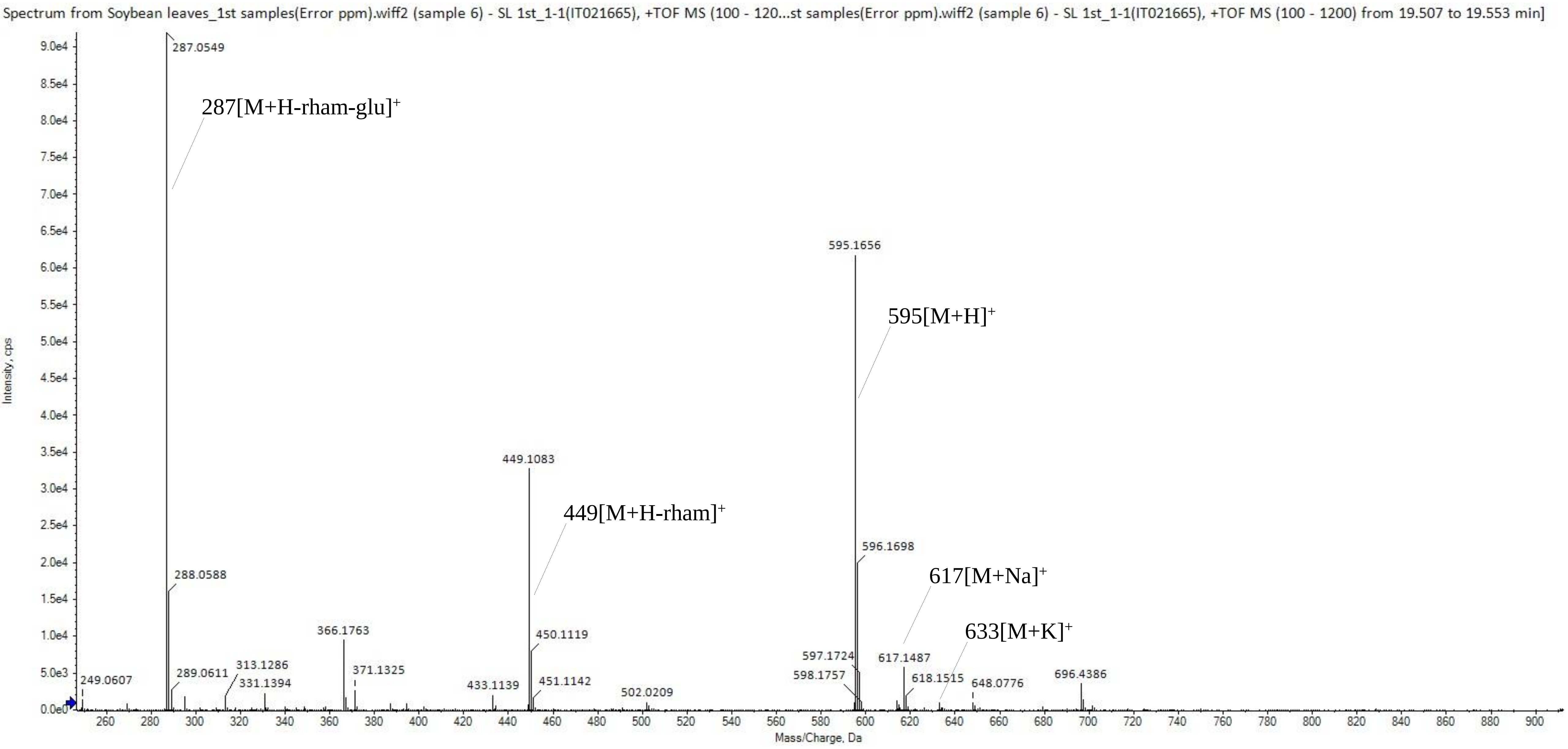

Peak **60**_I 3-*O*-(6-*O*-rham)gal (I 3-*O*-rob) (SL4)

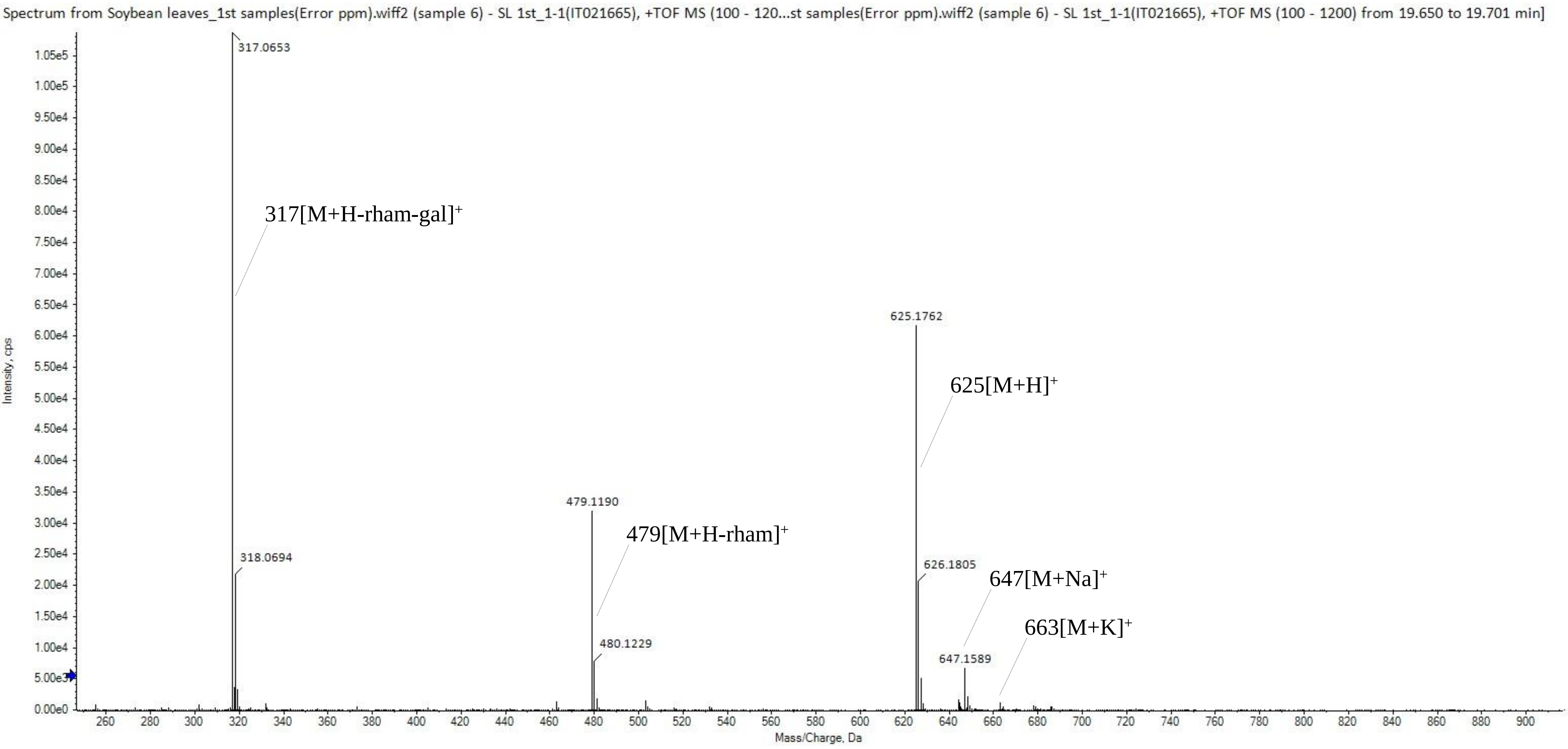

Peak **62**_I 3-*O*-(6-*O*-rham)glu (I 3-*O*-rut, narcissin) (SL4)

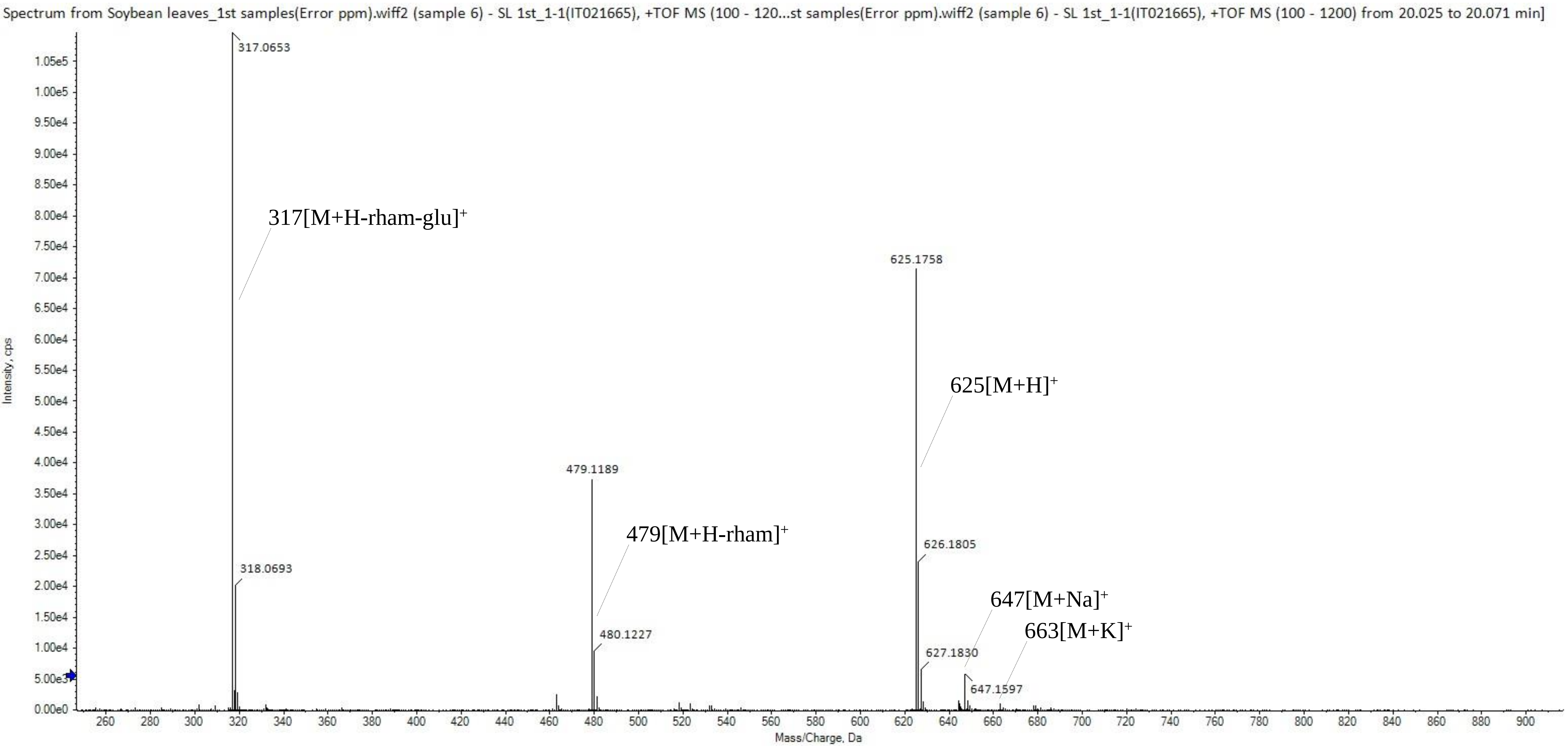

Peak **63**_K 3-*O*-glu (astragalin) (SL4)

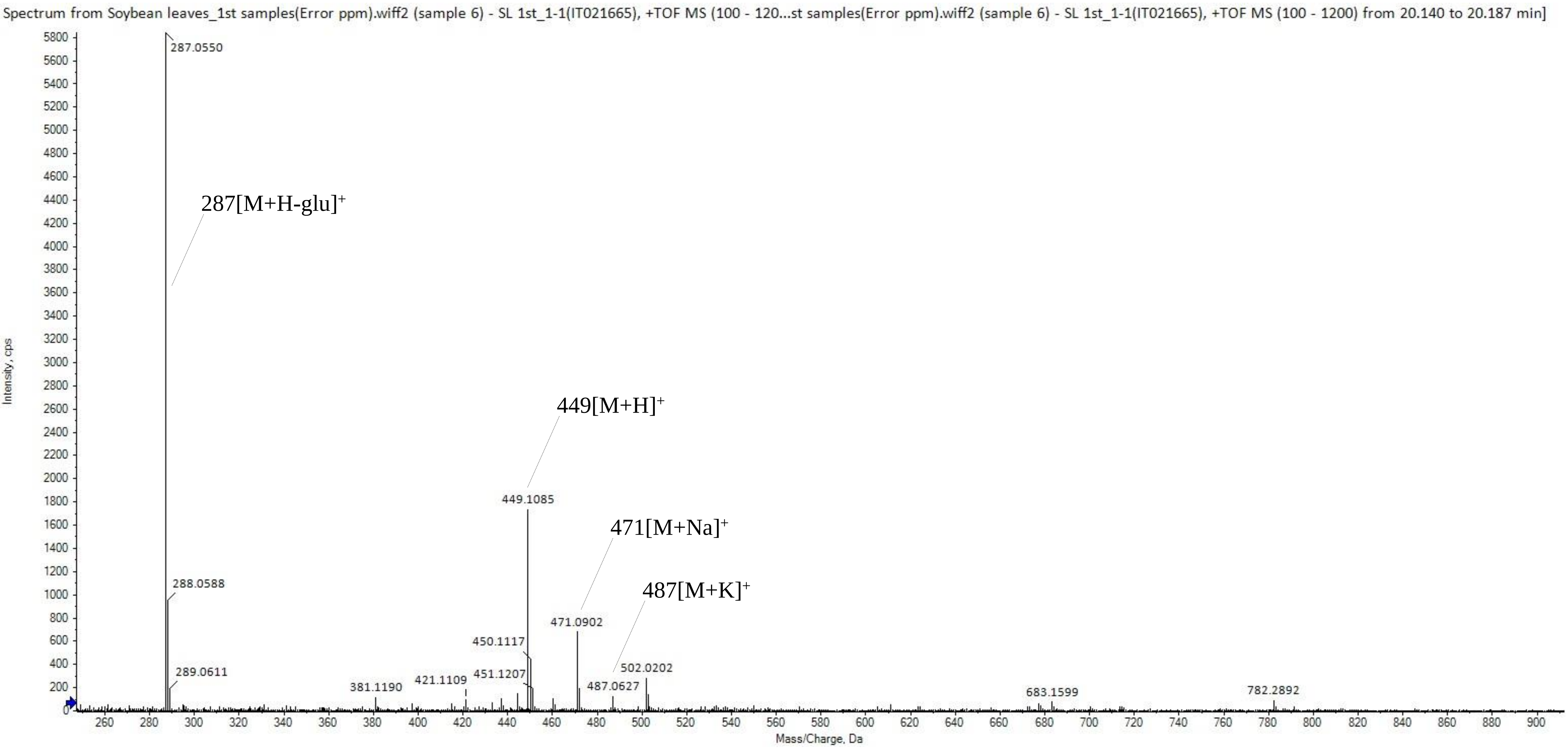

Peak **64**_I 3-*O*-gal (cacticin) (SL4)

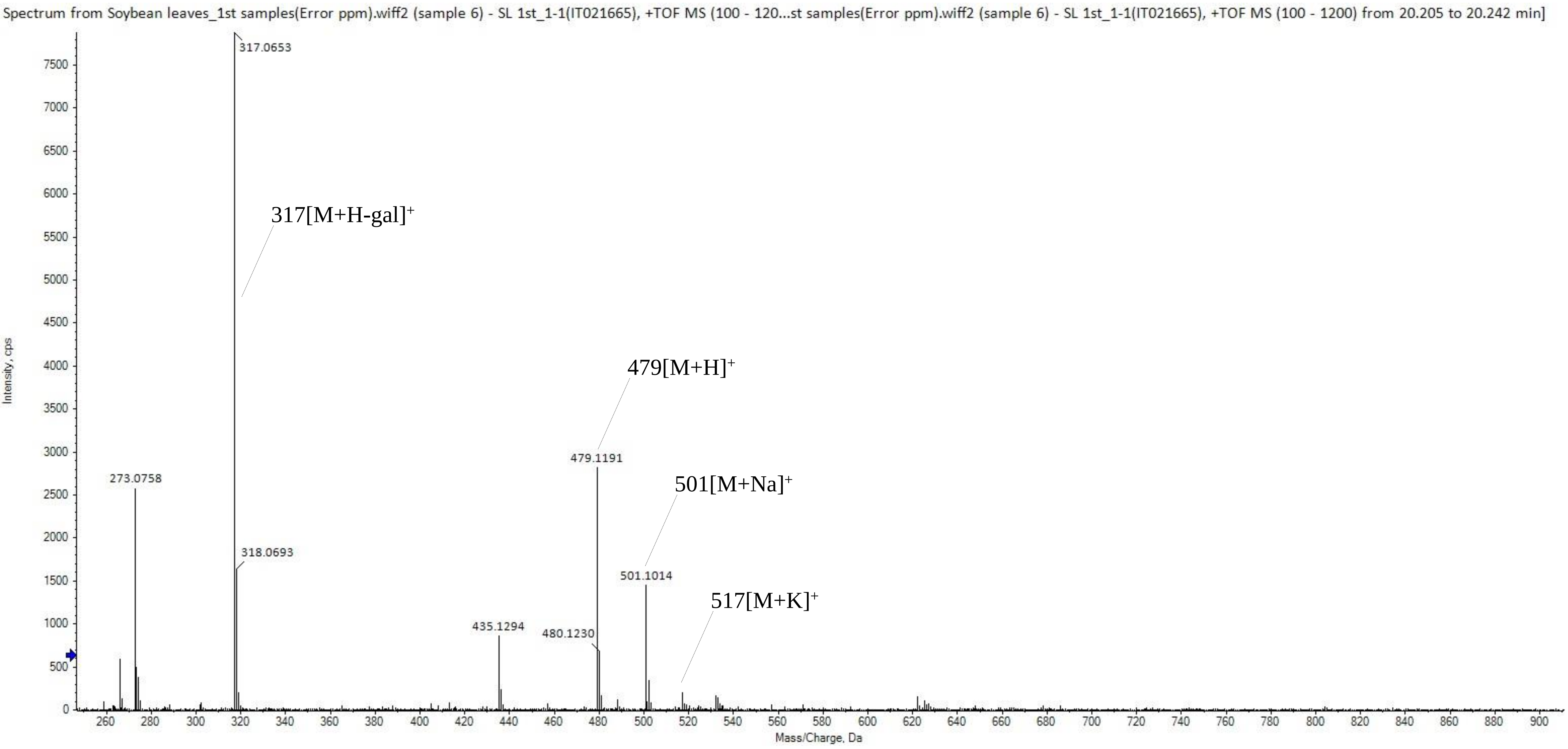

Peak **66**_I 3-*O*-glu (SL18)

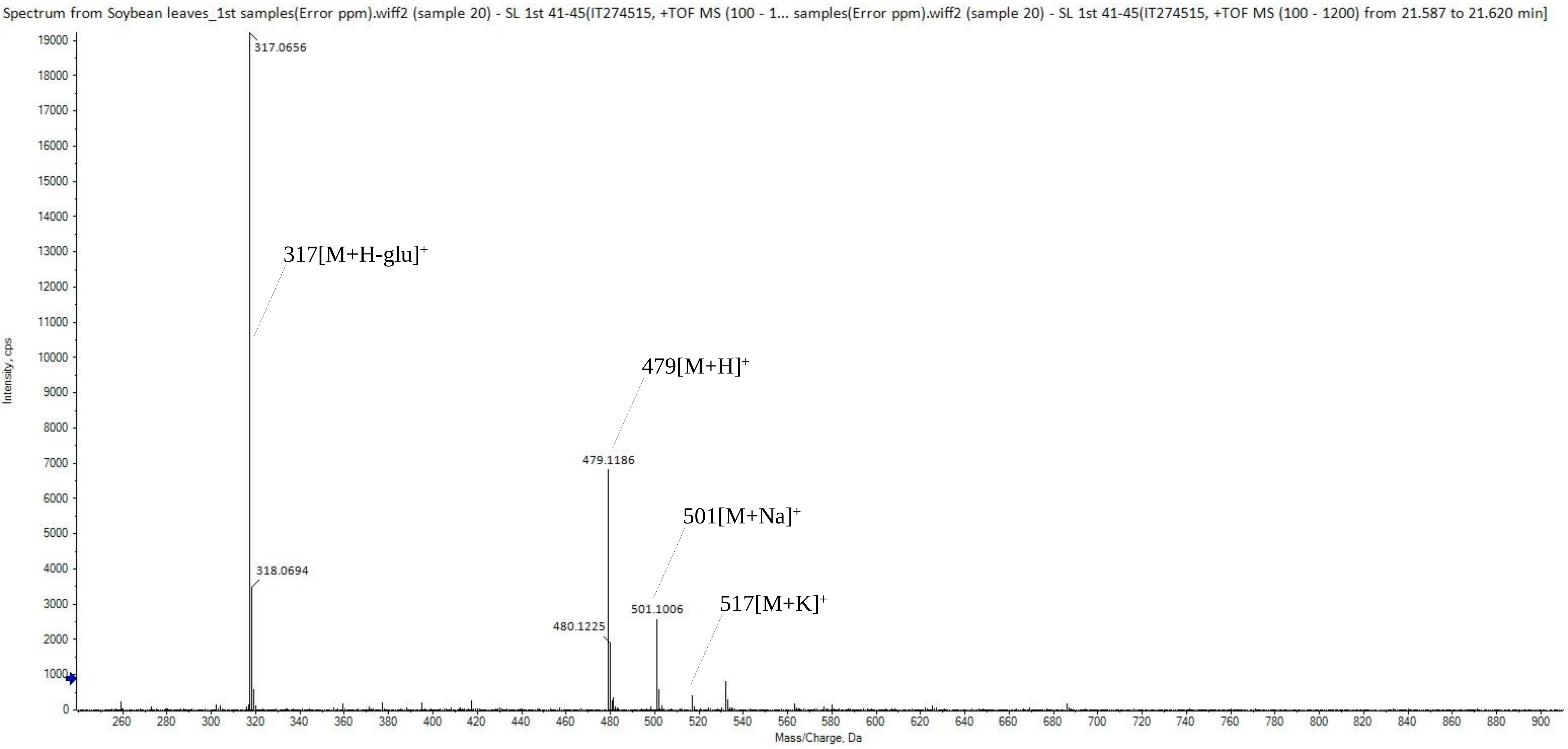

Supplement: Supplementary file 2 — Supplementary Information 2. [file 41598_2022_18226_MOESM2_ESM.pdf]
