# Supplementary material for: Comprehensive characterization of flavonoid derivatives in young leaves of core-collected soybean (Glycine max L.) cultivars based on high-resolution mass spectrometry
Source: Sci Rep. 2022 Aug 29;12:14678. doi: 10.1038/s41598-022-18226-4 (PMC9424525; doi:10.1038/s41598-022-18226-4)
Supplement: Supplementary file 3 — Supplementary Information 3. [file 41598_2022_18226_MOESM3_ESM.pdf]

Supplementary Figure S3. Proposed biosynthetic pathways of **A**) 17 kaempferol (**K**) and **B**) 18 quercetin (**Q**) glycosides. gal, galactoside (galactosyl); glu, glucoside (glucosyl); rham, rhamnoside(rhamnosyl); gen, gentiobiose; neo, neohesperidose; rob, robinobiose; rut, rutinose; sop, sophorose. Compound names of each peak are presented in Table 2.

A

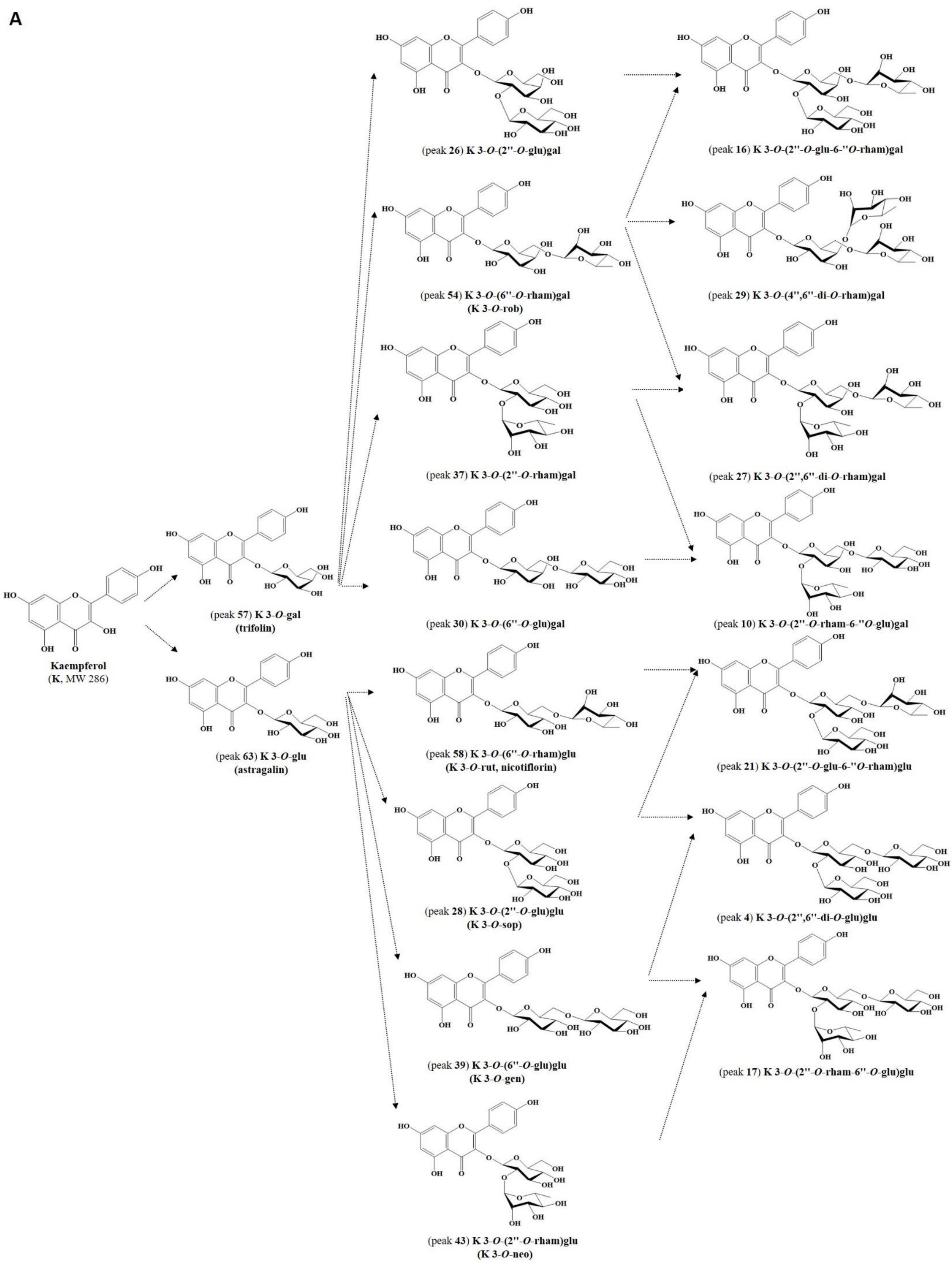
Kaempferol
(K, MW 286)
(peak 57) K 3-O-gal
(trifolin)
(peak 63) K 3-O-glu
(astragalin)
(peak 26) K 3-O-(2''-O-glu)gal
(peak 54) K 3-O-(6''-O-rham)gal
(K 3-O-rob)
(peak 37) K 3-O-(2''-O-rham)gal
(peak 30) K 3-O-(6''-O-glu)gal
(peak 58) K 3-O-(6''-O-rham)glu
(K 3-O-rut, nicotiflorin)
(peak 28) K 3-O-(2''-O-glu)glu
(K 3-O-sop)
(peak 39) K 3-O-(6''-O-glu)glu
(K 3-O-gen)
(peak 43) K 3-O-(2''-O-rham)glu
(K 3-O-neo)
(peak 16) K 3-O-(2''-O-glu-6-''O-rham)gal
(peak 29) K 3-O-(4'',6''-di-O-rham)gal
(peak 27) K 3-O-(2'',6''-di-O-rham)gal
(peak 10) K 3-O-(2''-O-rham-6-''O-glu)gal
(peak 21) K 3-O-(2''-O-glu-6-''O-rham)glu
(peak 4) K 3-O-(2'',6''-di-O-glu)glu
(peak 17) K 3-O-(2''-O-rham-6''-O-glu)glu

B

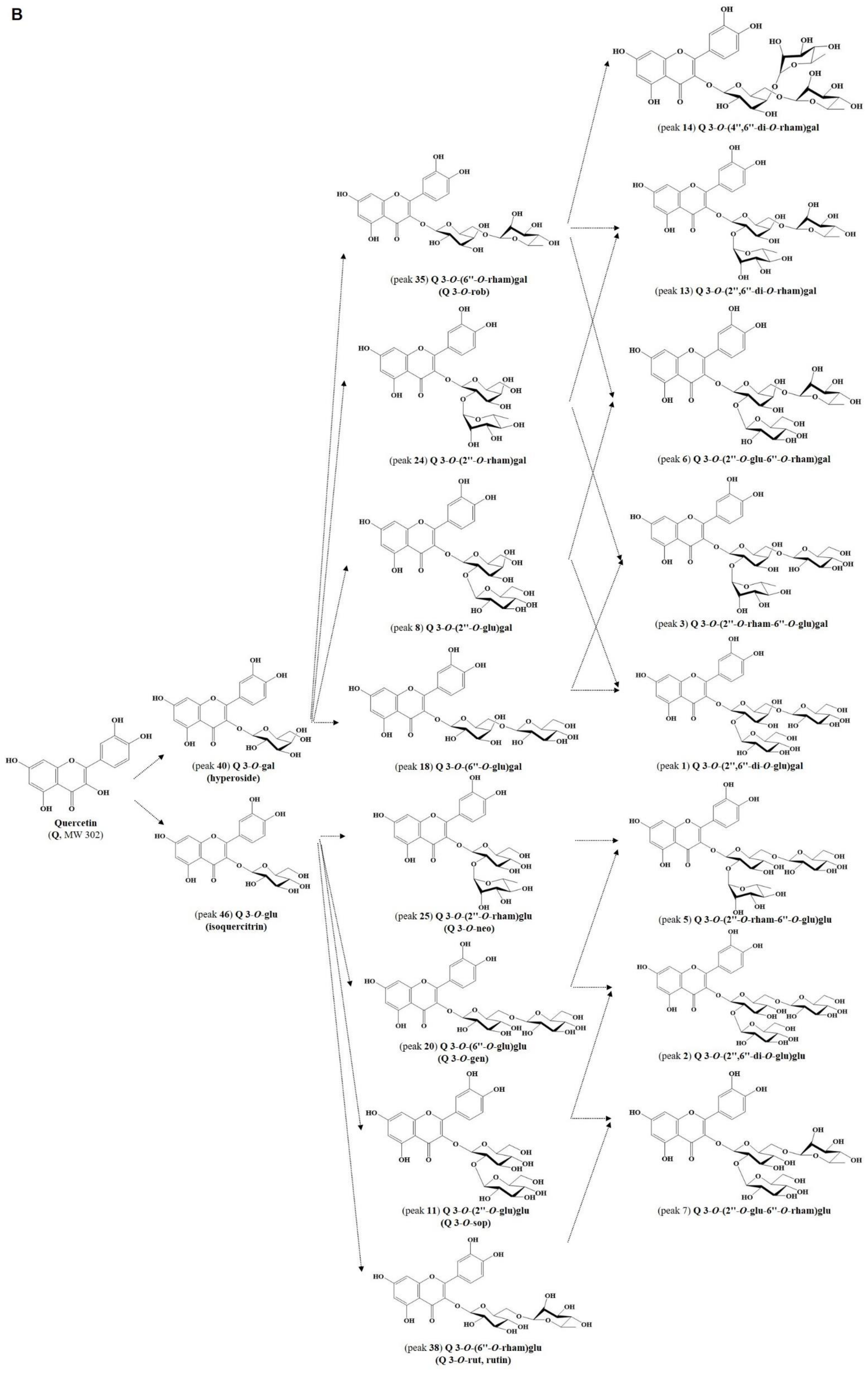
Quercetin
(Q, MW 302)
(peak 40) Q 3-O-gal
(hyperoside)
(peak 46) Q 3-O-glu
(isoquercitrin)
(peak 35) Q 3-O-(6''-O-rham)gal
(Q 3-O-rob)
(peak 24) Q 3-O-(2''-O-rham)gal
(peak 8) Q 3-O-(2''-O-glu)gal
(peak 18) Q 3-O-(6''-O-glu)gal
(peak 25) Q 3-O-(2''-O-rham)glu
(Q 3-O-neo)
(peak 20) Q 3-O-(6''-O-glu)glu
(Q 3-O-gen)
(peak 11) Q 3-O-(2''-O-glu)glu
(Q 3-O-sop)
(peak 38) Q 3-O-(6''-O-rham)glu
(Q 3-O-rut, rutin)
(peak 14) Q 3-O-(4'',6''-di-O-rham)gal
(peak 13) Q 3-O-(2'',6''-di-O-rham)gal
(peak 6) Q 3-O-(2''-O-glu-6''-O-rham)gal
(peak 3) Q 3-O-(2''-O-rham-6''-O-glu)gal
(peak 1) Q 3-O-(2'',6''-di-O-glu)gal
(peak 5) Q 3-O-(2''-O-rham-6''-O-glu)glu
(peak 2) Q 3-O-(2'',6''-di-O-glu)glu
(peak 7) Q 3-O-(2''-O-glu-6''-O-rham)glu
