# Supplementary material for: Comprehensive characterization of flavonoid derivatives in young leaves of core-collected soybean (Glycine max L.) cultivars based on high-resolution mass spectrometry
Source: Sci Rep. 2022 Aug 29;12:14678. doi: 10.1038/s41598-022-18226-4 (PMC9424525; doi:10.1038/s41598-022-18226-4)
Supplement: Supplementary file 4 — Supplementary Information 4. [file 41598_2022_18226_MOESM4_ESM.pdf]

Supplementary Table S1. The LC-MS library of 53 flavonoids from soybean (*Glycine max* L.) leaves based on the literature sources.

No.	Class	Compound names	Molecular weight	Fragmentations (*m/z*)		Literatures[3)]
				Positive[1)]	Negative[2)]	
1		3, 4',5,7-tetrahydroxyflavonol (kaempferol)	286	325, 309, 287		3, 4, 10
2		3,3',4',5,7-pentahydroxyflavonol (quercetin)	302	341, 325, 303		3
3		kaempferol 3-*O*-glucoside (astragalin)	448	487, 471, **449**, **287**	447	2, 11
4		quercetin 3-O-galactosdie (hyperoside)	464	503, 487, 465, **303**		18
5		quercetin 3-O-glucoside (isoquercitrin)	464	503, 487, 465, **303**		18
6		kaempferol 3-*O*-(2"-*O*-rhamnosyl)galactoside	594	633, 617, **595**, 449, 287		3, 6
7		kaempferol 3-*O*-(6"-*O*-rhamnosyl)galactoside (kaempferol 3-*O*-robinobioside, biorobin)	594	633, 617, **595**, 449, 287	593	1, 3, 7, 11, 15, 18
8		kaempferol 3-*O*-(6"-*O*-rhamnosyl)glucoside (kaempferol 3-O-rutinoside, nicotiflorin)-	594	633, 617, **595**, 449, 287		1, 3, 7, 18
9		kaempferol 3-*O*-(2"-*O*-glucosyl)galactoside	610	649, 633, 611, 449, 287	609, 285	3, 5, 13
10		kaempferol 3-*O*-(6"-*O*-galactosyl)galactoside	610	649, 633, **611**, **449**, **287**	609	1, 3, 11, 15
11		kaempferol 3-*O*-(2"-*O*-glucosyl)glucoside (kaempferol 3-*O*-sophoroside)	610	649, 633, 611, 449, 287		2, 3, 5
12		kaempferol 3-*O*-(6"-*O*-glucosyl)glucoside (kaemperol 3-*O*-gentiobioside)	610	649, 633, **611**, **449**, **287**	609	1, 3, 11, 15
13		quercetin 3-*O*-(6"-*O*-rhamnosyl)galactoside (quercetin 3-*O*-robinobioside)	610	649, 633, 611, 465, 303		7, 18
14		quercetin 3-*O*-(6"-*O*-rhamnosyl)glucoside (quercetin 3-*O*-rutinoside, rutin)	610	649, 633, 611, 465, 303		7, 18
15		isorhamnetin 3-*O*-(2"-*O*-rhamnosyl)galactoside	624	663, 647, 625, 479, 317		3, 6
16	Flavonol	isorhamnetin 3-*O*-(6"-*O*-rhamnosyl)galactoside (isorhamnetin 3-*O*-robinobioside)	624	663, 647, 625, 479, 317		7, 18
17		isorhamnetin 3-*O*-(6"-*O*-rhamnosyl)glucoside (isorhamnetin 3-*O*-rutinoside, narcissin)	624	663, 647, 625, 479, 317		3, 7, 18
18		quercetin 3-*O*-(2"-*O*-rhamnosyl)galactoside	626	665, 649, 627, 465, 303		3, 6
19		quercetin 3-*O*-(2"-*O*-glucosyl)galactoside	626	665, 649, 627, 465, 303		3, 6
20		quercetin 3-*O*-(2"-*O*-glucosyl)glucoside (quercetin 3-*O*-sophorosde)	626	665, 649, 627, 465, 303		3, 6
21		isorhamnetin 3-*O*-(2"-*O*-glucosyl)galactoside	640	679, 663, 641, 479, 317		3, 6
22		kaempferol 3-*O*-(2",6"-di-*O*-rhamnosyl)galactoside	740	779, 763, **741**, **595**, **449**, **287**	739, 285	1, 3, 5, 6, 11, 13, 15
23		kaempferol 3-*O*-(4",6"-di-*O*-rhamnosyl)galactoside	740	779, **763**, 741, **595**, **449**, **287**	739	3, 7, 8, 18
24		kaempferol 3-*O*-(2"-*O*-rhamnosyl-6"-*O*-glucosyl)galactoside	756	795, 779, **757**, **611**, 595, **449**, **287**		1, 3
25		kaempferol 3-*O*-(4"-*O*-rhamnosyl-6"-*O*-glucosyl)galactoside	756	795, **779**, 757, 611, 595, **449**, **287**	755	3, 8
26		kaempferol 3-*O*-(2"-*O*-glucosyl-6"-*O*-rhamnosyl)galactoside	756	795, **779**, 757, 611, 595, **449**, **287**	755, 285	3, 5, 8, 11, 13, 15
27		kaempferol 3-*O*-(2"-*O*-glucosyl-6"-*O*-rhamnosyl)glucoside	756	795, **779**, 757, 611, 595, **449**, **287**	755, 285	3, 5, 8, 11, 13, 15
28		quercetin 3-*O*-(4",6"-di-*O*-rhamnosyl)galactoside	756	795, 779, **757**, **611**, **465**, **303**	755	7, 18
29		quercetin 3-*O*-(2"-*O*-glucosyl-6"-*O*-rhamnosyl)galactoside	772	811, **795**, 773, 627, 611, **465**, **303**	771	3, 6, 8
30		quercetin 3-*O*-(2"-*O*-glucosyl-6"-*O*-rhamnosyl)glucoside	772	811, **795**, **773**, 627, 611, **465**, **303**		3, 6, 8
31		4',7 -dihydroxyflavone	254	287, 271, **255**		9
32		5,7-dihydroxyflavone (chrysin)	254	287, 271, 255	253	11
33	Flavone	4',5,7 -trihydroxyflavone (apigenin)	270	309, 293, 271		2, 3, 4
34		3',4',5,7-tetrahydroxyflavone (luteolin)	286	325, 309, 287		3, 4
35		apigenin 7-*O*-glucoside (cosmosiin)	432	471, 455, 433, 271	431	11
36		luteolin 7-*O*-glucoside (cynaroside)	448	487, 471, 449, 287		3, 4, 18

37		chrysoeriol 7-*O*-glucoside (thermopsoside)	462	485, 463, 301, 286		18
38		4',7-dihydroxyisoflavone (daidzein)	254	293, 277, **255**	253	3, 4, 9, 10, 11, 16, 17
39		4'-methoxy-7-hydroxyisoflavone (formononetin)	268	307, 291, **269**		3, 9, 12
40		4'-hydroxy-7-methoxyisoflavone (isoformononetin)	268	307, 291, **269**		3, 12, 14
41		4',5,7-trihydroxyisoflavone (genistein)	270	309, 293, 271	269	3, 4, 10, 11, 16, 17
42		4',7-dihydroxy-6-methoxyisoflavone (glycitein)	284	323, 307, 285	283	3, 4, 11, 17
43		4',6-dimethoxy-7-hydroxyisoflavone (afromosin)	298	337, 321, **299**		3, 12, 16
44		daidzein 7-*O*-glucoside (daidzin)	416	461, 455, 439, **417**, 255	415,253	3, 4, 9, 10, 11, 15, 16, 17, 18
45	Isoflavone	formononetin 7-*O*-glucoside (ononin)	430	468, 452, **431**, 269		9
46		genistein 7-*O*-glucoside (genistin)	432	477, 471, 455, **433**, 271	431, 269	1, 2, 3, 4, 10, 11, 15, 16, 17, 18
47		glyctein 7-*O*-glucoside (glycitin)	446	491, 469, 447, 285	445	15, 16, 17
48		afromosin 7-*O*-glucoside	460	505, 461, 299		16
49		daidzein 7-*O*-(6"-*O*-malonyl)glucoside (6"-*O*-malonyldaidzin)	502	541, 525, **503**, 255	253	3, 6, 9, 10, 15, 16, 17
50		formononetin 7-*O*-(6"-*O*-malonyl)glucoside (6"-*O*-malonylononin)	516	555, 539, **517**, 269		9
51		afromosin 7-*O*-malonyl glucoside	546	547, 299		16
52		glyctein 7-*O*-(6"-*O*-malonyl)glucoside (6"-*O*-malonylglycitin)	532	555, 533, 285	283	16
53		genistein 7-*O*-(6"-*O*-malonyl)glucoside (6"-*O*-malonylgenistin)	518	557, 541, **519**, 271	517, 269	1, 3, 6, 10, 11, 15, 16

1) Positive fragmentations under (+) ESI-ionization mode, bold font indicates fragment ions reported from literature; the remaining's are fragment ions proposed in this study (*m/z*, $[M+H]^+$, including adduct ions of $[M+Na]^+$ and $[M+K]^+$).

2) Negative fragmentations under (-) ESI-ionization mode from literature (*m/z*, $[M-H]^-$).

3) **1**, Ho et al., 2002; **2**, Hur et al., 2001; **3**, Jeong et al., 2019; **4**, Lee et al., 2008; **5**, Li et al, 2015; **6**, Li et al., 2019; **7**, Murai et al., 2019; **8**, Murai et al., 2013; **9**, Murakami et al., 2014; **10**, Romani et al., 2003; **11**, Song et al., 2014; **12**, Yuk et al., 2011; **13**, Zang et al., 2011; **14**, Ingham et al., 1981; **15**, Yuk et al., 2016; **16**, Veremeichik et al., 2021; **17**, Veremeichik et al., 2019; **18**, Iwashina et al., 2018.

References for library

1. Ho, H. M. *et al*. Difference in flavonoid and isoflavone profile between soybean and soy leaf. *Biomed and Pharmacother*. **56**, 289-295 (2002).
2. Hur, J. M., Park, S. J., Park, J. G., Hwang, Y. H. & Park, J. C. Flavonoids from the leaves of *Glycine max* showing anti-lipid peroxidative effect. *Nat Prod Sci*. **7**, 49-52 (2001).
3. Jeong, T. S. *et al*. Composition for prevention or treatment of metabolic syndrome or for antioxidation containing black bean leaf extracts and flavonol glycosides isolated therefrom as active ingredients. *Patent Application Publication, United States.* US2019/0075823A1 (2019).
4. Lee, J. H. *et al*. Phytochemical constituents from the leaves of soybean [*Glycine max* (L.) Merr.]. *Food Sci Biotechnol.* **17**, 578-586 (2008).
5. Li, H. *et al*. Soy leaf extract containing kaempferol glycosides and pheophorbides improves glucose homeostasis by enhancing pancreatic *β*-cell function and suppressing hepatic lipid accumulation in *db/db* mice. *J Agri Food Chem.* **63**, 7198-7210 (2015).
6. Li, H. *et al*. Suppression of hyperglycemia and hepatic steatosis by black-soybean-leaf extract via enhanced adiponectin-receptor signaling and AMPK activation. *J Agri Food Chem.* **67**, 90-101 (2019).
7. Murai, Y., Takahashi, R., Kitajima, J. & Iwashina, T. New quercetin triglycoside from the leaves of soybean cultivar 'Clark'. *Nat Prod Commun*. **14**(5), 1934578X19843614 (2019).
8. Murai, Y., Takahashi, R., Rodas, F. R., Kitajima, J. & Iwashina, T. New flavonol triglycosides from the leaves of soybean cultivars. *Nat Prod Commun*. **8**(4), 453-456 (2013).
9. Murakami, S.; Nakata, R.; Aboshi, T.; Yoshinaga, N.; Teraishi, M.; Okumoto, Y.; Ishihara, A.; Morisaka, H.; Huffaker, A.; Schmelz, E. A. & Mori, N.; Insect-induced daidzein,

formononetin and their conjugates in soybean leaves. *Metabolites*. *4*, 532-546 (2014).

10. Romani, A.; Vignolini, P.; Galardi, C.; Aroldi, C.; Vazzana, C. & Heimler, D.; Polyphenolic content in different plant parts of soy cultivars grown under natural conditions. *J Agri Food Chem*. ***51***, 5301-5306 (2013).
11. Song, H-H. *et al*. Metabolomics investigation of flavonoid synthesis in soybean leaves depending on the growth stage. *Metabolomics*. **10**, 833-841 (2014).
12. Yuk, H. J. *et al*. The most abundant polyphenol of soy leaves, coumestrol, displays potent α-glucosidase inhibitory activity. *Food Chem*. **126**, 1057-1063 (2011).
13. Zang, Y., Sato, H. & Igarashi, K. Anti-diabetic effects of a kaempferol glycoside-rich fraction from unripe soybean (Edamame, *Glycine max* L. Merrill. 'Jindai') leaves on KK-A^y mice. *Biosci Biotechnol Biochem.* **75**, 1677-1684 (2011).
14. Ingham, J. L., Keen, N. T., Mulheirn, L. J. & Lyne, R. L. Inducibly-formed isoflavonoids from leaves of soybean. *Phytochem*. **20**, 795-798 (1981).
15. Yuk, H. J. *et al*. Ethylene induced a high accumulation of dietary isoflavones and expression of isoflavonoid biosynthetic genes in soybean (*Glycine max*) leaves. *J Agri Food Chem*. **64**, 7315-7324 (2016).
16. Veremeichik, G. N. *et al*. Isoflavonoid biosynthesis in cultivated and wild soybeans grown in the field under adverse climate conditions. *Food Chem*. **342**, 128292 (2021).
17. Veremeichik, G. N. *et al*. Increase in isoflavonoid content in *Glycine max* cells transformed by the constitutively active Ca^{2+} independent form of the *AtCPK1* gene. *Phytochem*. **157**, 111-120 (2019).
18. Iwashina, T. *et al*. Flavonoids from three Wild Glycine species in Japan and Taiwan. *Nat Prod Commun*. **13**(12), 1641-1644 (2018).
